# Mechanisms of aortic dissection: From pathological changes to experimental and *in silico* models

**DOI:** 10.1016/j.pmatsci.2024.101363

**Published:** 2024-09-12

**Authors:** Malte Rolf-Pissarczyk, Richard Schussnig, Thomas-Peter Fries, Dominik Fleischmann, John A. Elefteriades, Jay D. Humphrey, Gerhard A. Holzapfel

**Affiliations:** a Institute of Biomechanics, Graz University of Technology, Austria; b High-Performance Scientific Computing, University of Augsburg, Germany; c Institute of Structural Analysis, Graz University of Technology, Austria; d 3D and Quantitative Imaging Laboratory, Department of Radiology, Stanford University, USA; e Aortic Institute, Yale University School of Medicine, New Haven, USA; f Department of Biomedical Engineering, Yale University, New Haven, USA; g Department of Structural Engineering, Norwegian University of Science and Technology (NTNU), Trondheim, Norway

**Keywords:** Aortic dissection, Microstructure, Morphology, Material characterization, Computational modeling, Computational mechanics, Fluid-structure interaction, Damage mechanics, Hemodynamics, Computational fluid dynamics, Patient-specific simulation

## Abstract

Aortic dissection continues to be responsible for significant morbidity and mortality, although recent advances in medical data assimilation and in experimental and *in silico* models have improved our understanding of the initiation and progression of the accumulation of blood within the aortic wall. Hence, there remains a pressing necessity for innovative and enhanced models to more accurately characterize the associated pathological changes. Early on, experimental models were employed to uncover mechanisms in aortic dissection, such as hemodynamic changes and alterations in wall microstructure, and to assess the efficacy of medical implants. While experimental models were once the only option available, more recently they are also being used to validate *in silico* models. Based on an improved understanding of the deteriorated microstructure of the aortic wall, numerous multiscale material models have been proposed in recent decades to study the state of stress in dissected aortas, including the changes associated with damage and failure. Furthermore, when integrated with accessible patient-derived medical data, *in silico* models prove to be an invaluable tool for identifying correlations between hemodynamics, wall stresses, or thrombus formation in the deteriorated aortic wall. They are also advantageous for model-guided design of medical implants with the aim of evaluating the deployment and migration of implants in patients. Nonetheless, the utility of *in silico* models depends largely on patient-derived medical data, such as chosen boundary conditions or tissue properties. In this review article, our objective is to provide a thorough summary of medical data elucidating the pathological alterations associated with this disease. Concurrently, we aim to assess experimental models, as well as multiscale material and patient data-informed *in silico* models, that investigate various aspects of aortic dissection. In conclusion, we present a discourse on future perspectives, encompassing aspects of disease modeling, numerical challenges, and clinical applications, with a particular focus on aortic dissection. The aspiration is to inspire future studies, deepen our comprehension of the disease, and ultimately shape clinical care and treatment decisions.

## 1. Introduction

Patients often describe the onset of aortic dissection as the most severe pain a person can feel. Notably, this pain is frequently characterized by anatomically precise terms such as ‘tearing’ and ‘ripping’. Medical textbooks have taught us the ‘classic’ nature of the constellation of symptoms and signs of acute aortic dissection. A classic case might be a 72-year-old male patient who has a long history of high blood pressure and suddenly experiences the onset of intense, sharp pain between his shoulder blades. Upon arrival at the hospital, the patient appears sweaty and agitated, his blood pressure is 195mmHg/110mmHg. Although many patients report symptoms such as chest, back, or abdominal pain, there is significant overlap with other acute syndromes and signs, making a precise diagnosis particularly difficult. In fact, most patients with aortic dissection do not have these classic symptoms, but only some of the well-known indicators. This has led to aortic dissection being referred to as ‘the great masquerader’ [1,2].

Aortic dissection is relatively uncommon, with incidences ranging from approximately three to eight cases per 100,000 individuals, yet responsible for significant morbidity and mortality [3–5]. This condition is characterized by delamination of the aortic wall, followed by blood inflow into the medial layer creating a new, false lumen, and eventually resulting in backflow into the actual, true lumen downstream. When left untreated, the mortality rate of acute ascending aortic dissection increases by 1 to 2% per hour within the first 24 hours of arriving at the hospital. As a result, mortality rates of up to 50 to 74% have been observed within the first two weeks [3,4]. Estimates suggest that up to 40% of cases are fatal prior to arrival at the hospital [6]. In contrast, an acute descending aortic dissection is generally less severe, with survival rates of up to 89% in medically treated patients after one month and 80% after five years [6].

Traditionally, aortic dissections involving the ascending aorta were treated with open surgery, while dissections beyond the aortic arch were managed through medical treatment [7,8]. However, over the past two decades, this approach has undergone a paradigm shift due to significant advances in stent-graft technology, a better understanding of the initiation and propagation of the disease, including hemodynamics in the true and false lumina, and improvements in medical treatment, resulting in fewer long-term dissection-related complications [9]. These advances are not solely due to new medical data but are also associated with the progression of *in silico* medicine, which describes the application of computational modeling and simulations to study, diagnose, treat, or prevent diseases, such as aortic dissection. Novel numerical tools enable detailed analyses of specific phenomena in healthy and dissected aortas without the need for costly and time-consuming experiments or invasive measurements. Moreover, these tools have demonstrated their ability to accelerate the development of new medical devices and enable treatment comparisons or studies on virtual cohorts.

Over the last decade, several review articles on aortic dissection have been published, providing various perspectives on what is arguably one of the most challenging and complex vascular diseases. An early review article on this topic was given by Humphrey [10]. Subsequently, Rajagopal et al. [11] published a review article to better understand the mechanics behind aortic dissection. Tsamis et al. [12] characterized the microstructure of the aorta and its relationship to aging and disease, with a particular emphasis on alterations in fibrous components. Sherifova and Holzapfel [13] examined pathological structural changes in aortic dissection, focusing on possible genetic causes. Peterss et al. [14] highlighted the differences in the acute and chronic phases of the disease, discussed changes related to morphology, and provided a comprehensive overview of the current state of research on these changes. Similarly, Spinelli et al. [15] provided an overview of current evidence on predicting dilatation and growth in aortic dissections, while Zilbert et al. [16] discussed the relationship between false lumen hemodynamics and aortic remodeling. In addition, several review articles summarized mechanical experiments and structural investigations on dissected tissue [17–19], as well as experimental models both *in vivo* and *ex vivo* [20], or solely *in vitro* [21]. These experiments and models are critical for the development of multiscale material and *in silico* models for the various pathological phenomena observed in aortic dissection. Finally, several review articles have discussed multiscale material and *in silico* models [19,22–25]. Some have even considered aortic dissection from both clinical and biomechanical perspectives [22,23,26–31]. Moreover, several other review articles focus on imaging techniques and their challenges [32–35], as well as the diagnosis and treatment of aortic dissections [36], among others, which lie beyond the scope of this work.

A common limitation of current studies is their narrow focus. The studies available today often focus on specific pathological aspects, experimental models, or modeling approaches. These may include the microstructure or mechanical behavior of the pathological aortic wall or hemodynamics in patient-specific studies. The limited scope of these studies prevents a more comprehensive understanding of aortic dissection, which is crucial for comprehending the initiation and progression of aortic dissection and for improving treatment and management strategies. Our objective in this review article is to provide a detailed summary of two main aspects. First, this review article provides a detailed presentation of the available medical data describing the pathological changes in aortic dissection, including remodeled anatomy and vessel walls, changed hemodynamics, and potential thrombus formation. These medical data are fundamental for the development and validation of physics-based computational or *in silico* models. It is important to note that the accuracy of *in silico* models heavily depends on the available medical data, such as patient-specific geometries, blood inflow and outflow conditions, and information about mechanical behavior and aortic microstructure. Without such data, the obtained results may be subject to significant uncertainty, affecting their reliability and applicability for clinical decision-making. Therefore, advances in disease understanding and the development of *in silico* models would be unthinkable without the significant impact of modern medical imaging technology and associated data. Second, this review article summarizes experimental, multiscale material, and patient data-informed *in silico* models that have advanced our understanding of the mechanisms underlying aortic dissection, as well as patient management. These models also underscore the application of the medical and experimental data utilized in their development. Specifically, it is important to emphasize the methods used, applications, and key findings to help the reader understand the available literature and the current state of the art. In conclusion, we offer a discussion on future perspectives, particularly focusing on aortic dissection. This includes considerations of disease-related modeling aspects, numerical challenges, and clinical applications, all aimed at motivating targeted future research in biomedical engineering and healthcare. Ultimately, these efforts are intended to enhance clinical decision-making and advance personalized medicine.

The structure of this review article is as follows. Section 2 provides background information about aortic dissection, followed by an overview of pathological changes in Section 3, including remodeled anatomy, remodeled wall microstructure and mechanics, changed hemodynamics, and the role of thrombus formation. Section 4 then discusses the available experimental models, followed by an examination of multiscale material models, with emphasis on the damage and failure of the dissected aorta in Section 5. The next three sections discuss *in silico* models that explore various phenomena related to aortic dissection using patient-derived medical data. This encompasses *in silico* hemodynamic models in Section 6, coupled *in silico* models on the interaction between fluid and solid in Section 7, and, subsequently, *in silico* models on selected topics such as wall stress analysis in patient-specific studies, stent-graft deployment and migration, and thrombus formation in Section 8. Finally, Section 9 provides a summary and discusses future perspectives.

## 2. A basic introduction to aortic dissection

Frank Nicholls is believed to have been the first person to describe type A dissection more than 250 years ago, in 1760 [37]. As the personal physician to George II, King of England, he witnessed the King’s death due to sustained fatal cardiac tamponade caused by an acute type A dissection that had ruptured into the pericardial sac. In 1819, about 60 years later, René Laennec was probably the first to introduce the term ‘dissecting aneurysm’. Laennec did not know at the time that this term would unfortunately lead to ongoing confusion about aortic dissection and thoracic aortic aneurysm. This term is now considered incorrect because dissection without the presence of an aneurysm tends to be more common. The more accurate term ‘aortic dissection’, proposed by Jean Pierre Maunoir in 1802, has been largely overlooked due to Laennec’s greater prominence [38].

A first milestone was achieved in 1954 with the successful surgical resection of a dissecting thoracic aortic aneurysm by the renowned surgeons Michael E. DeBakey, Denton A. Cooley, and Oscar Creech [39]. Subsequent advances in cardiopulmonary bypass and adjunctive techniques for circulatory management opened the door to surgical treatment of ascending aortic dissection. This led to several further milestones in the early and mid-1960s: Spencer and his colleagues at New York University first reported surgical treatment of chronic ascending aortic dissection with aortic insufficiency in 1962 [40]; and Morris and his colleagues in Houston reported the first successful treatment of acute aortic dissection with aortic insufficiency in 1963 [41,42]. Finally, pharmacological treatment of descending aortic dissection was introduced by Wheat and his colleagues in 1965 [43], based on control of systemic arterial pressure and ‘global’ rate of the left ventricular ejection d*p*/d*t*. As early as 1980, DeBakey and his colleagues demonstrated significant clinical and surgical expertise in the management of aortic dissection patients, as evidenced by their 20-year follow-up study of 527 patients who had received surgical treatment [44]. Remarkably, DeBakey himself underwent and survived open surgery for a type A dissection at age 97 [45].

Decades later, our modern understanding and treatment of aortic dissection have been shaped by two significant developments. The first was the establishment of the International Registry of Acute Aortic Dissection (IRAD) in 1996, whose numerous contributions have significantly advanced our knowledge and understanding of aortic dissection over the last decades. The second pivotal event was the publication of two groundbreaking papers in the 1999 issue of the New England Journal of Medicine, which for the first time detailed endovascular stent-graft intervention for acute type B dissection [7,46]. These articles marked the beginning of the endovascular era in the management of aortic dissections.

This fascinating early history of aortic dissection was partly extracted from the documentation by Criado [47] and by Rajagopal et al. [11]. The following section provides a basic introduction to the biomechanics of the normal aorta. This is followed by a concise overview of current guidelines on classification and definition, patient treatment and imaging, and recognized associated aortopathies.

### 2.1. Normal aortic biomechanics

#### Anatomy.

**Fig. 1.**
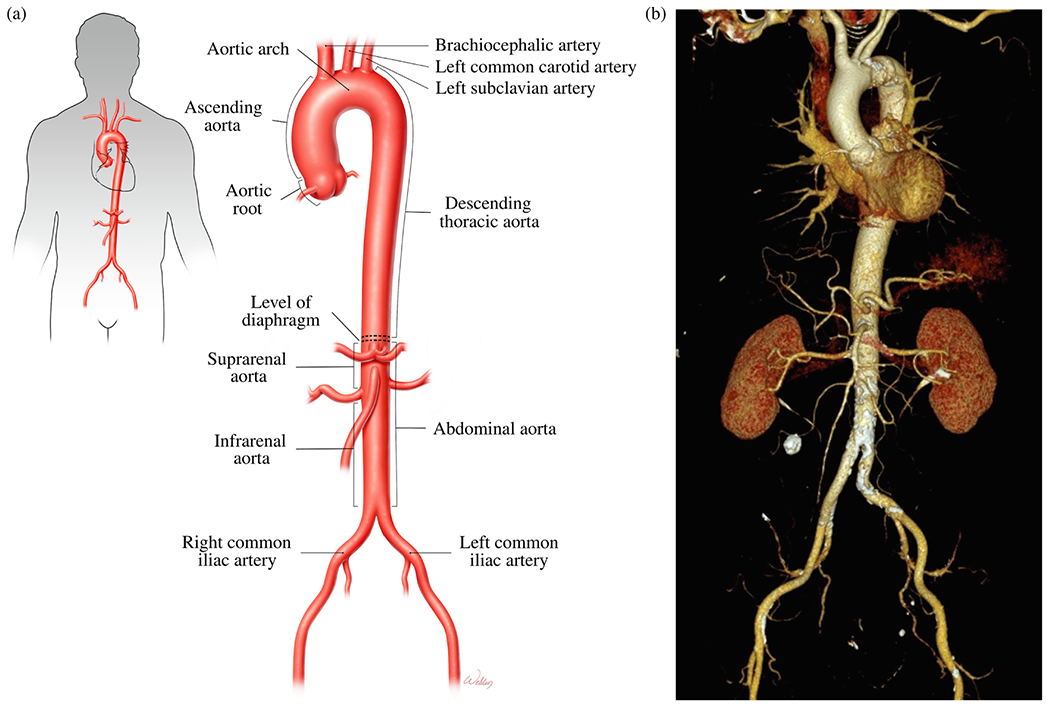
(a) Schematic representation of the aortic anatomy and its main branch vessels. (b) Computed tomography angiography images of the aorta and peripheral vasculature. Figure (a) is adapted from Shen et al. [49] with permission from Elsevier, while (b) is from Patel et al. [50] (licensed under CC-BY 4.0).

The aorta, the largest and most important artery in the human body, is responsible for transporting oxygen-rich blood from the heart to the distal vessels that distribute it throughout the body and for returning the blood to the heart muscle [48]. As illustrated in Figs. 1(a) and (b), this crucial vessel can be divided into five main anatomical segments, following the most common anatomical description among the numerous segments available.

The first section of the aorta, called the aortic root, arises in the left ventricle, includes the aortic valve and the sinuses of Valsalva, and extends to the sinotubular junction. The ascending thoracic aorta, the second section, is approximately 5cm long and extends from the sinotubular junction to the innominate or brachiocephalic artery. It then forms the third section, the aortic arch, also referred to as the transverse aorta. The arch stretches from the innominate to the left subclavian artery and is approximately 4 to 5cm long. First, it ascends diagonally to the left over the anterior portion of the trachea, then descends to the left of the fourth thoracic vertebral body, continuing as the descending segment of the thoracic aorta. There are numerous variations in the anatomy of the thoracic aortic arch, typically involving branching patterns of the large vessels and the location of the descending thoracic aorta in relation to the spine [51]. The most common configuration is the left aortic arch, from which the brachiocephalic artery, the left common carotid artery, and the left subclavian artery branch off. The brachiocephalic artery is further subdivided into the right common carotid artery and the subclavian artery.

Following the arch, the aorta proceeds as a descending thoracic aorta, the fourth section, which extends from the left subclavian artery to the diaphragm and is about 20cm long. It arises at the level of the intervertebral disc between the fourth and fifth thoracic vertebrae, penetrates the diaphragm, and ends anteriorly between the twelfth thoracic and first lumbar vertebrae. The descending thoracic aorta is initially located on the left side of the spine; however, at the level of the seventh thoracic vertebra, it shifts slightly to the right, anterior to the vertebral bodies. It is worth noting a small anatomical feature located on the proximal descending aorta, just distal to the left subclavian artery: the ligamentum arteriosum. This remnant is a leftover from the ductus arteriosus, a structure that connects the fetal pulmonary circulation to the aorta. The descending thoracic aorta gives rise to several larger vessels as well as a number of smaller but clinically important arteries. These vessels supply the bronchi, esophagus, intercostal muscles, and spinal cord. The intercostal arteries arise from the posterolateral aspect of the thoracic aorta and extend from the level of the third to the twelfth vertebral bodies.

Finally, the abdominal aorta extends from the diaphragm to the aortic bifurcation and branches into numerous smaller arteries that supply blood to the abdominal organs and lower limbs. Sometimes it is divided into the superior and inferior segments, which are delineated from the aorta by the branching of the renal arteries. From its origin at the aortic valve to the aortic bifurcation in the abdomen, the size of the aorta decreases [52,53]. Directly above the coronary sinuses, the diameter of the ascending aorta is typically between 2.5 and 3.5cm. The arch and descending thoracic aorta are often only slightly narrower than the ascending aorta. This tapering is particularly important for the so-called ‘Windkessel effect’.

#### Windkessel effect.

The heart continuously pumps oxygen and nutrient-rich blood throughout the body to sustain life. To achieve this, the heart generates pressure and flow waves in the arteries. As the heart contracts, the pressure in the ventricle rises rapidly at the beginning of systole and soon exceeds the pressure in the aorta, causing the aortic valve to open, blood to be ejected, and aortic pressure to rise. While ventricular pressure falls rapidly as the heart muscle relaxes, aortic pressure falls more slowly due to the elastic properties of the aorta. The aorta and some proximal large vessels act as an elastic buffer chamber behind the heart, as explained by the Windkessel theory [54–56]. In this way, they serve as a reservoir for the stroke volume ejected by the left ventricle during systole. During diastole, the elastic properties of the aortic wall propel this volume forward into the peripheral arteries. This systolic-diastolic interaction, which represents the Windkessel effect, not only aids perfusion of peripheral arteries, but also benefits the heart by reducing left ventricular afterload and improving coronary blood flow and ventricular relaxation.

**Fig. 2.**
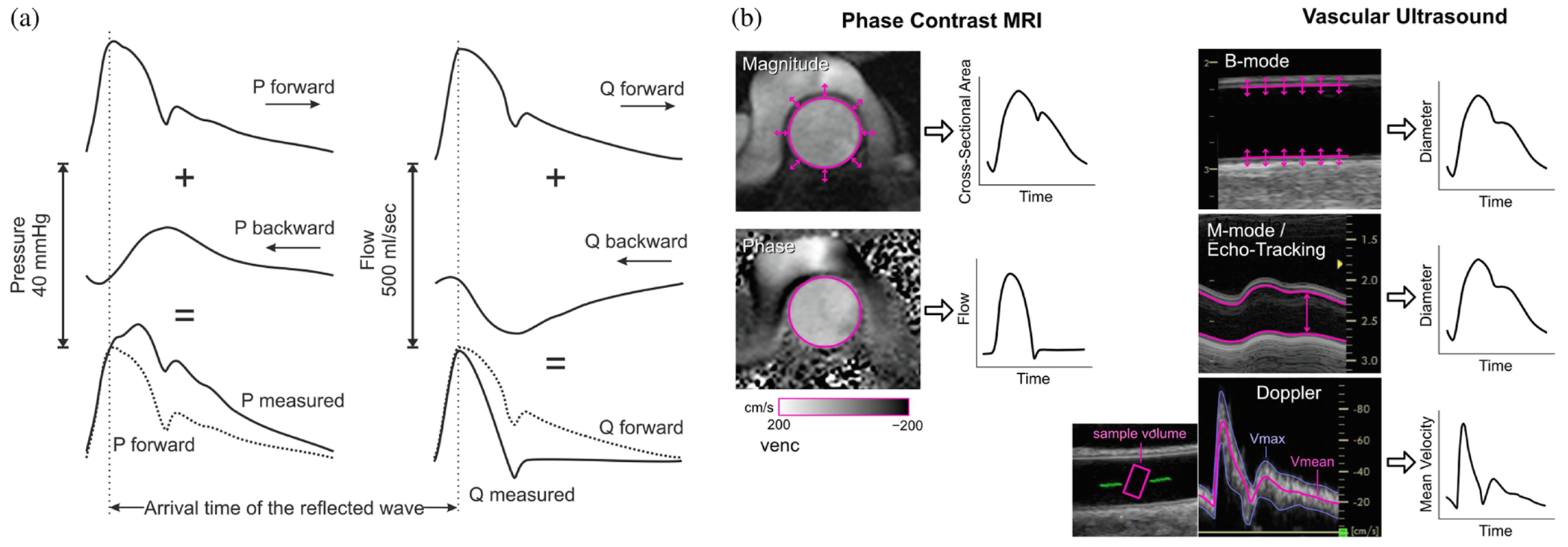
(a) Representative curves illustrating the principles of arterial wave reflection theory and the effects on pressure P and flow Q waveforms caused by reflected waves. (b) Selected non-invasive medical imaging methods (phase contrast-magnetic resonance imaging (MRI) and ultrasound imaging) to obtain cross-sectional area or diameter waveforms and flow or mean velocity waveforms of a patient’s artery. Figure (a) is adapted from Pagoulatou et al. [57] (licensed under CC-BY 4.0), while (b) is from Mynard et al. [58] (licensed under CC-BY 4.0).

As one moves away from the aortic valve, the pressure wave changes, in particular there is a sharpening and an increase in amplitude pressure, while the so-called dicrotic notch gradually decreases. This elevation in systolic pressure as it travels through the circulatory system is primarily due to wave reflections, see Fig. 2(a). Wave reflections occur in the arterial system at locations where there is a discontinuity in artery size, elasticity, or a mismatch in vascular impedance. Common reflection sites include branching points, areas where diameter and elasticity vary, and high-resistance arterioles, which are significant reflection points. Conversely, for a constant flow in a rigid tube, the pressure would decrease along the flow direction unless there is deceleration. Although the amplitude of the pressure between systole and diastole nearly doubles due to reflections, resulting in a reduced pressure load on the left ventricle, the average pressure over the cardiac cycle decreases with increasing distance.

The speed at which arterial pressure propagates through the circulatory system is called pulse wave velocity and is due to vascular compliance, i.e. the ability of the vessel to expand under the forces exerted by blood pressure. It is typically calculated by dividing the distance between two points in the recording arteries by the pressure wave transit time. This measurement is a standard indicator of central arterial stiffness [59–63]. Higher pulse wave velocity values indicate stiffer arteries, which may be associated with an increased risk of cardiovascular events such as heart attack and stroke. Conversely, lower pulse wave velocity values suggest more elastic and healthier arteries. In addition, increased arterial stiffness due to aging or pathophysiological conditions can lead to decreased distensibility of the aorta and other vessels, resulting in elevated intraluminal pressure.

These characteristic pressure and wave waveforms can be measured using both invasive and non-invasive techniques. While the gold standard for acquiring the blood pressure waveform is invasive measurement, such as with a micromanometer-tipped catheter, various surrogates are often used [64]. These surrogates measure distension (arterial diameter, area, or volume change) to approximate the pressure waveform. However, it is important to note that the relationship between pressure and distension is not strictly linear and exhibits hysteresis due to the viscoelastic properties of the vessel walls. Despite this, and due to their limited spatial and temporal resolution, ultrasound imaging and magnetic resonance imaging (MRI) are widely available and provide acceptable accuracy [58]. Fig. 2(b) shows representative results of MRI, illustrating the changes in cross-sectional area and flow information as well as edge tracking of the artery wall in B-mode and M-mode, and Doppler ultrasound for mean velocity measurements. An introduction to the main imaging modalities used can be found in Section 2.3.1.

#### Wall microstructure.

**Fig. 3.**
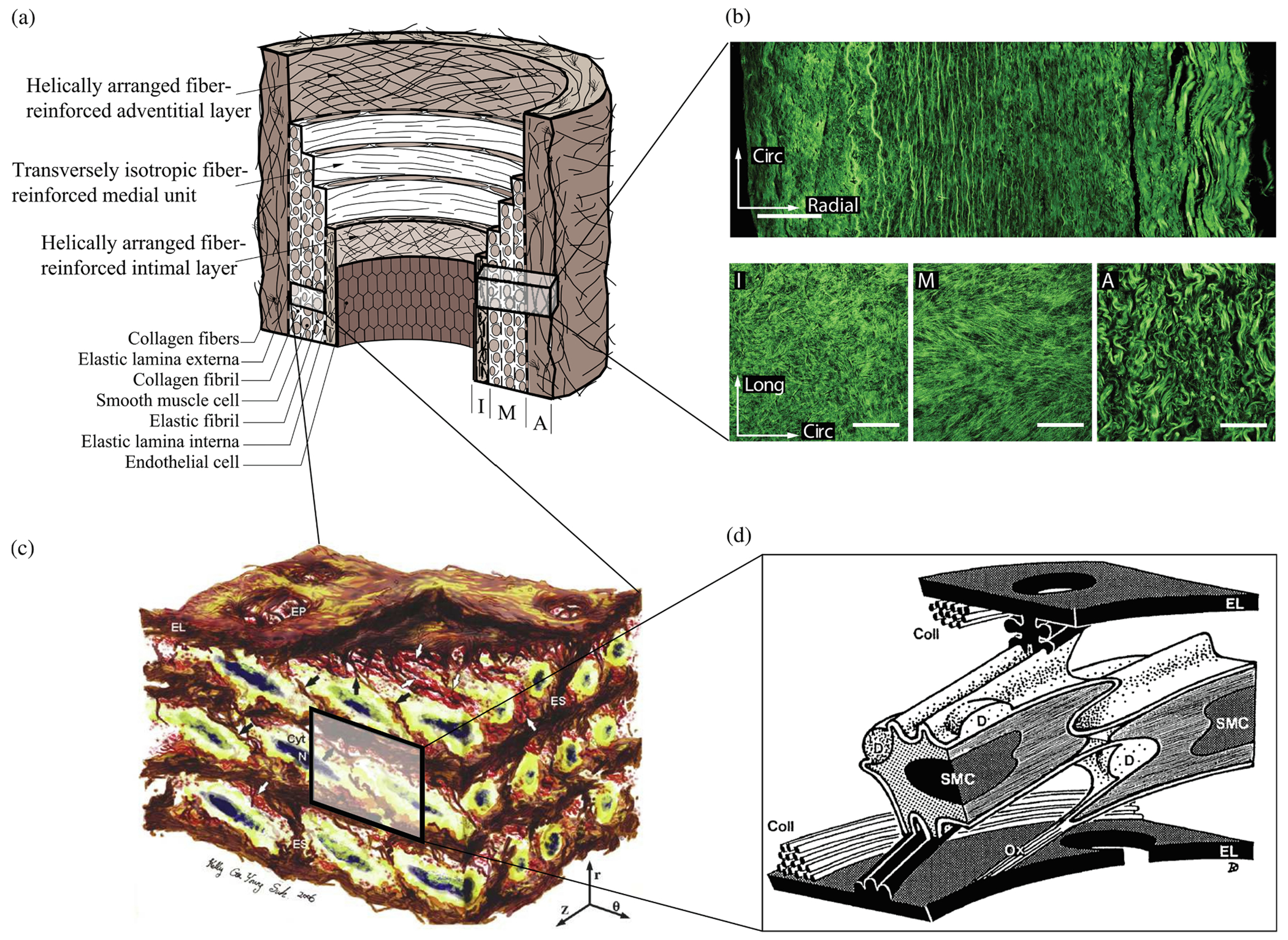
Illustration of the microstructure of the aortic wall showing (a) the composition of a healthy yet aged aortic wall exhibiting non-atherosclerotic intimal thickening. This structure consists of three layers, namely the intima (‘I’), media (‘M’), and adventitia (‘A’). (b) Layered collagen architecture of a healthy, aged abdominal aorta, where the top image shows an out-of-plane structure in the circumferential-radial plane. The three lower images present in-plane sections of the intima (‘I’), media (‘M’), and adventitia (‘A’), with white scale bars corresponding to 100 μm. (c) 3D microstructure of an aortic media composed of multiple lamellar units. These include circumferentially-oriented, radially-tilted smooth muscle cells (‘SMCs’) featuring elliptical nuclei (‘N’), located between elastic lamellae (‘EL’). These are surrounded by a dense network of interlamellar elastin fibers (‘IEFs’, indicated by black arrows), elastin struts (‘ES’), and reinforced elastin pores (‘EP’). (d) Schematic depiction of two SMCs and two fenestrated elastic lamellae with their interconnections. Specifically, collagen fibers (‘Coll’) are closely associated with elastic lamellae. The left SMC’s surface ridges are connected to both elastic lamellae via elastin protrusions, while the right SMC is connected to the lower elastic lamellar via an oxytalan fiber (*O*x). Larger deposits (‘D’) containing collagen and heparan sulfate proteoglycan can be found at indentations in the cell surface. Figure is reprinted from Sherifova and Holzapfel [18] with permission from Elsevier.

**Fig. 4.**
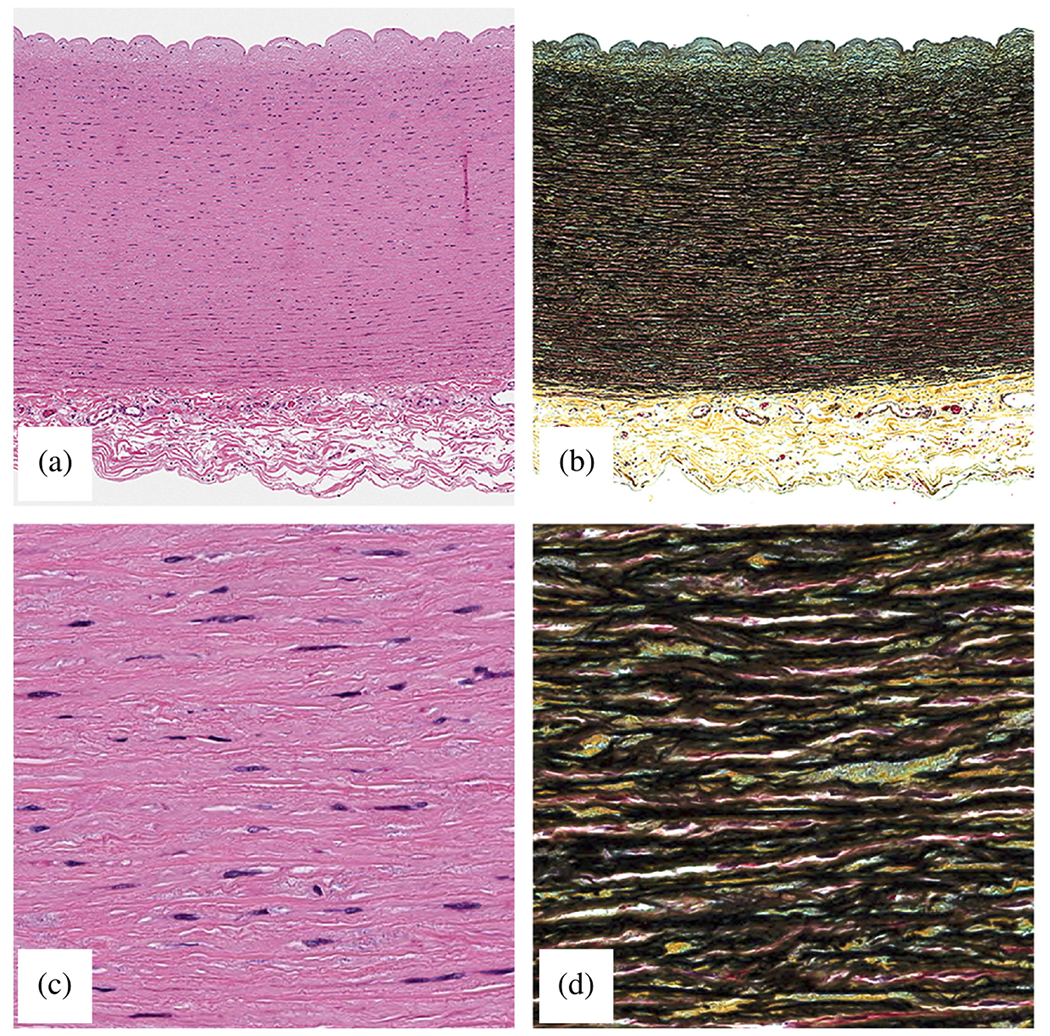
Histological images of the normal aortic wall of a young adult showing (a) a transverse the three layers of an aorta: intima, media, and adventitia from top to bottom (50x, H&E stain). (b) Image of the elastic fibers of the intima, a distinctly paler layer than the media, and the numerous lamellar units of the media delineated by black elastic laminae. There is a marked change at the media-adventitia boundary, with the adventitia comprised mostly of loose fibrous tissue (yellow). The normally thick vasa vasorum can be clearly seen (50x, Movat’s pentachrome stain). (c) Higher magnification of the lamellar units of the media with pronounced eosinophilic, refringent elastic laminae (500x, H&E stain). (d) Close-up view of the lamellar units. A single lamellar unit is defined as the components sandwiched between two elastic laminae: (i) elastic lamina, (ii) extracellular matrix (fibrous tissue (yellow) and mucopolysaccharides (green/blue)), (iii) SMCs (red cytoplasm and dark blue/burgundy nuclei), (iv) extracellular matrix, and (v) another elastic lamina (500x, Movat’s pentachrome stain). Figure is reprinted from Halushka et al. [66] with permission from Elsevier.

The normal aortic wall is characterized by three primary layers: intima, media, and adventitia, as depicted in the schematic image in Fig. 3(a) and the histological images in Figs. 4(a) and (c) [65]. Each layer exhibits a unique structure and composition required for its particular purpose and function. Apart from that, the intima and media are separated by the internal elastic lamina, while the external elastic lamina separates the media from the adventitia.

The intimal layer consists of a single layer of endothelial cells [65]. For many years, this cell layer was considered relatively inert, a mere physical barrier between the circulating blood and the underlying tissue. However, it is now known that endothelial cells are metabolically active [67,68]. They form a semi-permeable barrier that controls the passage of materials and white blood cells into and out of the bloodstream, while also allowing the selective transfer of nutrients, hormones, and waste products between the bloodstream and tissues. They play a crucial role in vascular homeostasis, which involves the regulation of blood flow and vascular tone through the release of various vasoactive factors that cause vasodilation or vasoconstriction. These may include vasodilatory factors such as nitric oxide (NO), an endothelium-dependent vasodilator of the underlying smooth muscle. In addition, they balance blood clotting, mediate inflammation and immune responses, contribute to the metabolism of lipids and other substances, and maintain vascular integrity by remodeling. Typically, endothelial cells are flat and elongated in the direction of blood flow and are subject to shear stresses caused by blood flow, commonly referred to as wall shear stresses (WSSs). WSSs are tangential or frictional forces exerted by blood flow on the inner surfaces of blood vessels. Endothelial cells can convert these mechanical stimuli into intracellular signals that result into the production of multiple biomolecules, some of which diffuse and affect smooth muscle cells (SMCs) located in the media, impairing their function [52].

Adjacent to this monolayer is a thin basal lamina with a thickness of about 80nm [52], which consists of net-like type IV collagen and adhesion molecules such as laminin. The endothelium not only senses shear stresses, it also plays a key role in hemostasis and also regulates the transport of substances such as nutrients, leukocytes, or lipids into the wall, thereby playing a critical role in the proper functioning of the aorta. In fact, endothelial dysfunction is often associated with the initiation and progression of cardiovascular diseases, including aortic dissection. Moreover, the layer only becomes mechanically significant with age-related changes, when collagen fibers are deposited, resulting in non-atherosclerotic intimal thickening [69].

In contrast, the media consists of many lamellar units, also known as musculo-elastic fascicles [70], with layers of elastic fibers separated by SMCs, and collagen fibers (primarily types III and V), as depicted in Fig. 3 and Fig. 4. These components are all embedded in the ground substance, which primarily consists of water and glycosaminoglycans (GAGs)/proteoglycans (PGs) (mainly versican). This complex network is commonly referred to as the extracellular matrix (ECM), as shown in Fig. 3. Interestingly, the thickness of a single lamellar unit remains the same across mammalian species [71,72], with an average thickness of 13.9±1.2μm [73]. Consequently, the thickness of the thoracic aortic media increases proportionally with the number of lamellar units. Overall, the media has 53 to 78 lamellar units in the proximal human aorta, with fewer units observed distally as the aortic diameter tapers [52]. The lamellae appear wavy in most collapsed specimens, but almost straight in distended specimens.

The primary component of the lamellar unit is elastic fibers, which consist mainly of elastin (~90%) and multiple elastin-associated glycoproteins (~10%) such as the fibrillins and fibulins. In contrast to collagen fibers, which undergo constant turnover in the arterial wall [74], elastic fibers are mainly deposited during the perinatal period and, due to their extended half-life, endure millions of loading cycles over the course of an individual’s life [75]. These elastic fibers exist in three forms: lamellae, interlamellar fibers, and inter-lamellar struts. Lamellae are thick, continuous sheets with a fibrous surface, numerous rounded fenestrations and a thickness of 2.1±0.6μm [73,76]. Gross irregularities are also observed, with the lamellae splitting and branching [77]. The intralamellar space contains dense, intricately organized interlamellar elastic fibers, which are cords or wisps of elastin and elastin-associated microfibrils that protrude obliquely from the top to the bottom of lamellar surfaces. They can form a cage-like structure surrounding and abutting SMCs, and are important for mechanosensing by the SMCs [78]. In contrast to less dense interlamellar elastic fibers, interlamellar struts are more substantial and form connections between adjacent lamellae and separate SMCs. Due to their considerable thickness and mainly radial orientation, they can play a critical role in supporting radial loads, including those originating from Gibbs-Donnan swelling pressures emanating from the aggregating medial PGs [79], which likely contributes to SMC mechanosensation.

Elliptically shaped SMCs are arranged between lamellae with their long axis aligned nearly circumferentially with a radial tilt of 19±3° [73], the details of which remain controversial. SMCs are also indirectly connected to the lamellae via conspicuous oxytalan and elaunin fibers to sense mechanical stress. Cyclic physiological tensile stresses tend to promote what is known as a contractile phenotype of SMCs, which under normal conditions maintains the normal ECM based on actomyosin-mediated mechanosensing and slow turnover (synthesis and degradation). In contrast, non-physiological stresses can lead to a synthetic SMC phenotype characterized by increased production of matrix metalloproteinase (MMP) and increased ECM synthesis. Depending on the relative intensity of these two processes, fibrosis or atrophy may ultimately occur. Finally, SMC apoptosis tends to increase when the medial layer is stress-shielded, and SMC anoikis occurs when the cells lose their connection to the ECM (a cell survival signal; [80]).

Collagen fibers, densely packed in the media and typically of type III, appear as interspersed fibers between elastic lamellae, either as fiber bundles, thin bundles, or individual fibers. These fiber bundles run parallel within each layer, implying greater packing efficiency and allowing more fibers to be packed into a smaller volume, which is advantageous for high loading conditions. Additionally, these fibers are helically woven around the vessel but are primarily circumferentially oriented [70,81], similar to other primary mural constituents, such as interlamellar elastic fibers or SMCs. Because of this alignment, they do not exhibit any radial tilt, which remains controversial. Collagen bundles are independent of elastin and SMCs and have no direct connections. However, fiber bundles within each layer tightly envelop the SMCs and may contribute to a pericellular coat. The fiber bundles often twist under mean physiological pressure. As pressure increases, more and more bundles are recruited and the fibers straighten. In addition to the elastic lamellae, collagen fibers serve as the mechanical basis of the media. More specifically, collagen fibers provide the aorta with high tensile strength, ensuring its structural integrity, while elastin gives the aortic wall its ability to expand and recoil in response to blood pressure, a phenomenon known as the Windkessel effect [82], as previously introduced. Due to the greater distensibility of elastin compared with collagen, this dual stress distribution is responsible for the characteristic nonlinear stress–strain behavior observed in soft tissues during *ex vivo* testing [83].

Finally, the adventitia consists mainly of a dense network of type I collagen fibers, along with scattered fibroblasts and tissue-resident macrophages, as well as sparse elastin, nerves, and vasa vasorum, which supply nutrients and oxygen to the aortic wall, as shown by the histological images in Figs. 4(a) and (c). The process of collagen I fibrillogenesis is supported by type III collagen as well as the small leucin-rich PGs decorin and biglycan. The importance of these accessory molecules is highlighted well by biglycan deficiency, which is associated with aortic dissection [84]. Normal collagen fibers in the adventitia appear undulated at physiological pressure but straighten at higher pressure, leading to the hypothesis that they serve as a protective sheath. In other words, the adventitia functions to prevent over-distension and vascular rupture due to acute pressure increases [65]. In contrast to the collagen fibers in the media, the collagen fibers in the adventitia are oriented more randomly, with their mean direction coinciding with the axial direction of the vessel [85].

The primary constituents of the ground substance found in each of the three tissue layers are water and GAGs/PGs, with varying compositions and organizations across the tissue depth [86]. GAGs, negatively charged polysaccharides, comprise repeating disaccharide units and are classified into six families: hyaluronic acid, dermatan sulfate, keratan sulfate, chondroitin sulfate, heparan sulfate, and heparin [87]. With the exception of hyaluronic acid, GAGs are covalently linked to protein cores, forming larger structures, the PGs, which are categorized based on cellular location, protein homology, and protein modules within their cores [88]. The ECM harbors both small and large PGs, with small PGs encompassing decorin, biglycan, and fibromodulin, and large PGs incorporating aggregan, versican, neucoran, and brevican [89]. The aortic wall contains hyaluronan and three types of sulfated GAGs: chondroitin sulfate, heparan sulfate, and dermatan sulfate [90,91]. In the media and adventitia, varying proportions of these GAGs are found, exhibiting a non–homogeneous distribution across the wall, with higher concentrations toward the media and intima [92]. Twenty different PG types have been identified within the ECM of the human thoracic aorta [93]. Versican, a large PG, is the primary component, accounting for 53.5% of total PGs and predominantly residing in the intima [94,95]. In contrast, small PGs such as biglycan and decorin account for 40% of the total PGs in the wall [94,95]. Biglycan is localized in the intima, outer media, and adventitia, while decorin is restricted to the adventitia in normal aortas [96]. In general, most PGs are found in the media, where they can either exist within the interstitial space or associate with elastin and collagen [97]. Although GAGs/PGs account for merely 2 to 5% of the thoracic aortic wall [98], they may play a significant role in the mechanobiological functions of the aortic wall due to their swelling capacity [92,99]. As negatively charged molecules, GAGs maintain electroneutrality by drawing in cations, which results in water influx and the generation of osmotic pressure in GAG-containing regions, a phenomenon known as Gibbs-Donnan swelling [100,101]. Swelling distribution across aortic thickness can vary [99], and establishing a correlation between transmural GAGs/PGs and swelling distributions is essential for better understanding residual stresses. Uniform swelling may put tension on the wall network [102], while localized swelling can produce elevated pressures, potentially leading to delamination and dissection in the aortic wall [103].

Residual stresses in the aorta are defined by the internal, self-equilibrating stresses that exist in the absence of external forces or pressure [86]. These stresses likely arise during vascular development, as revealed by changes in the intricate structure and composition of the aortic wall [104]. Residual stresses play a critical role in preserving the integrity and adaptability of the wall to changes in blood pressure and other physiological and pathological influences and contribute to load distribution across the aortic wall. This load distribution appears to be mechanobiologically favorable; it also minimizes stress concentrations and helps prevent damage. Residual stresses can be accounted for computationally via multiple approaches [52], including constrained mixture-based material models [105] and classic continuum mechanics models [106].

In conclusion, the aortic wall has an impressive microstructure of various interwoven constituents, resulting in an extremely complex architecture. Even minor deviations from this delicate structure can cause significant alterations in integrity and mechanical behavior, potentially leading to the onset of diseases such as aortic dissection. Although intense research efforts have already uncovered some peculiarities of arterial tissue and its composition, many questions remain unanswered to date.

#### Modeling aortic mechanics.

Aortic tissues pose significant challenges for modelers because they are complex mixtures of fluids, such as water; fibers such as collagen and elastin; cells; and a myriad of connective tissue proteins [107]. Over the course of the cardiac cycle, the aortic wall undergoes motion and deformation in response to various applied forces or loads, which are independent variables, while motions and deformations are dependent variables. Blood pressure acts perpendicularly and into the aortic intima, while blood flow along the aorta induces shear forces parallel to the intimal surface. These forces can be described, for instance, by the Cauchy stress tensor, which describes the state of stress at a given point within a material in the deformed configuration. This mathematical object has three normal components and six shear components. In fact, due to the symmetry of the stress tensor, only three of these shear components are independent. Additional complexity arises from the unique geometry of the aorta and its movement due to heart contractions, resulting in axial deformation and torsion. In contrast to intimal surface forces, aortic tethering to adjunct organs results in adventitial surface forces. Taken together, these factors culminate in a fully 3D stress state in the aortic wall.

To establish a relationship between the occurring stresses and deformations, constitutive relations can be formulated, typically based on continuity equations, although aortic tissue may not adhere to these assumptions due to its multiphasic and multicomponent nature. The most common approaches include classic continuum models that act on the organ scale and homogenize the aortic microstructure, multiscale models that incorporate detailed microscale knowledge by what is known as a representative volume element and allow for correlations between micro and macro behavior, and constrained mixture models that interpret biological tissue as a mixture of multiple constituents. For more details, we refer readers to [108–110]. While all constitutive relations have their justification, they always require a detailed understanding of the material composition. As previously demonstrated, this proves to be difficult because the aortic wall is a complex, layered structure composed of a heterogeneous mixture of various solid and fluid components. Additionally, the aortic wall is anisotropic, with varying degrees of anisotropy depending on location, and demonstrates a location-dependent viscoelastic response that has a positive effect on smoothing the pulsatile nature of flow, which is beneficial for perfusion [111–113]. While it is generally assumed to be incompressible, some studies define it as slightly compressible [114]. Most of its constituents exhibit nonlinear responses, and it is also essential to consider both the active and the passive mechanical behavior of the aorta. These material properties of aortic tissue are typically obtained by biopsying small pieces of tissue and measuring their characteristics. These measurements are then extrapolated to represent the entire aorta. Microstructural information can be obtained using a range of techniques, including histology, optical imaging methods such as second harmonic generation microscopy [115], X-ray transmission imaging methods such as micro-computed tomography (CT) [116], and non-invasive medical imaging techniques such as diffusion MRI [117,118], among others. Remarkably, only a few studies take into account the heterogeneous nature of the aortic wall – that is, the fact that each material point behaves differently – despite its critical importance.

In addition to that, a particular challenge lies in what is known as the initial stress or strain within the aorta. This is especially relevant for blood vessels like the aorta, as it is not *a priori* clear what the stress-free or strain-free reference configuration is – a baseline from which to measure deformation. In the past, and mainly based on Fung and his colleagues [119,120], researchers have assumed that the stress-free state of the aorta could be determined by making repeated incisions in the tissue. However, there is no certainty that all stresses have been completely relieved after a finite number of cuts. Additionally, while such incisions may lead to a global configuration, they do not guarantee that every local point in the body under study is stress free. Although this approach is a good approximation, the *in vivo* situation might be more complex.

The complexity of understanding aortic behavior lies not only in the aortic tissue itself but also in the intricate nature of blood, which presents its own unique challenges. Blood is a composite fluid made of gel-like cellular matter – including erythrocytes, leukocytes, and platelets – suspended in a plasma solution in which numerous proteins and various ions are dissolved in water as a solvent, adding another level of complexity. While the behavior of plasma can often be approximated using the Navier–Stokes constitutive theory, whole blood resists such simple modeling. For example, its behavior in the vasa vasorum or other small arteries cannot be captured by Navier–Stokes equations because blood shears significantly [121,122] and stress relaxes [123]. This shear-thinning property, resulting from the disassembly of erythrocyte rouleaux aggregates at high shear rates, should be accounted for in any comprehensive model.

Similar to other arteries, accurate mathematical modeling of the aorta presents particular challenges and becomes even more complex in the event of disease. It becomes clear that, with some exceptions, constitutive relationships that treat the arterial wall as a purely elastic body and are based on Navier–Stokes fluid models for blood are generally insufficient to provide reliable modeling results. To conclude, mathematical models that are as simple as possible without compromising accuracy and precision should be used. The underlying mathematical and physical theories must be conceptually rigorous, while accurately modeling real-world phenomena [124]. Although this is easy to state, the biomechanical complexity of the aorta in both health and disease makes modeling challenging, and it is often still unclear how many and which simplifications are still valid.

### 2.2. Definition and classification systems

Aortic dissection is a complex and challenging medical emergency that presents a wide range of clinical features. Over the past several decades, experts from various disciplines have sought to define and classify this condition to enhance communication and understanding based on anatomy, acuity, and those with prognostic impact [125].

#### Definition.

**Fig. 5.**
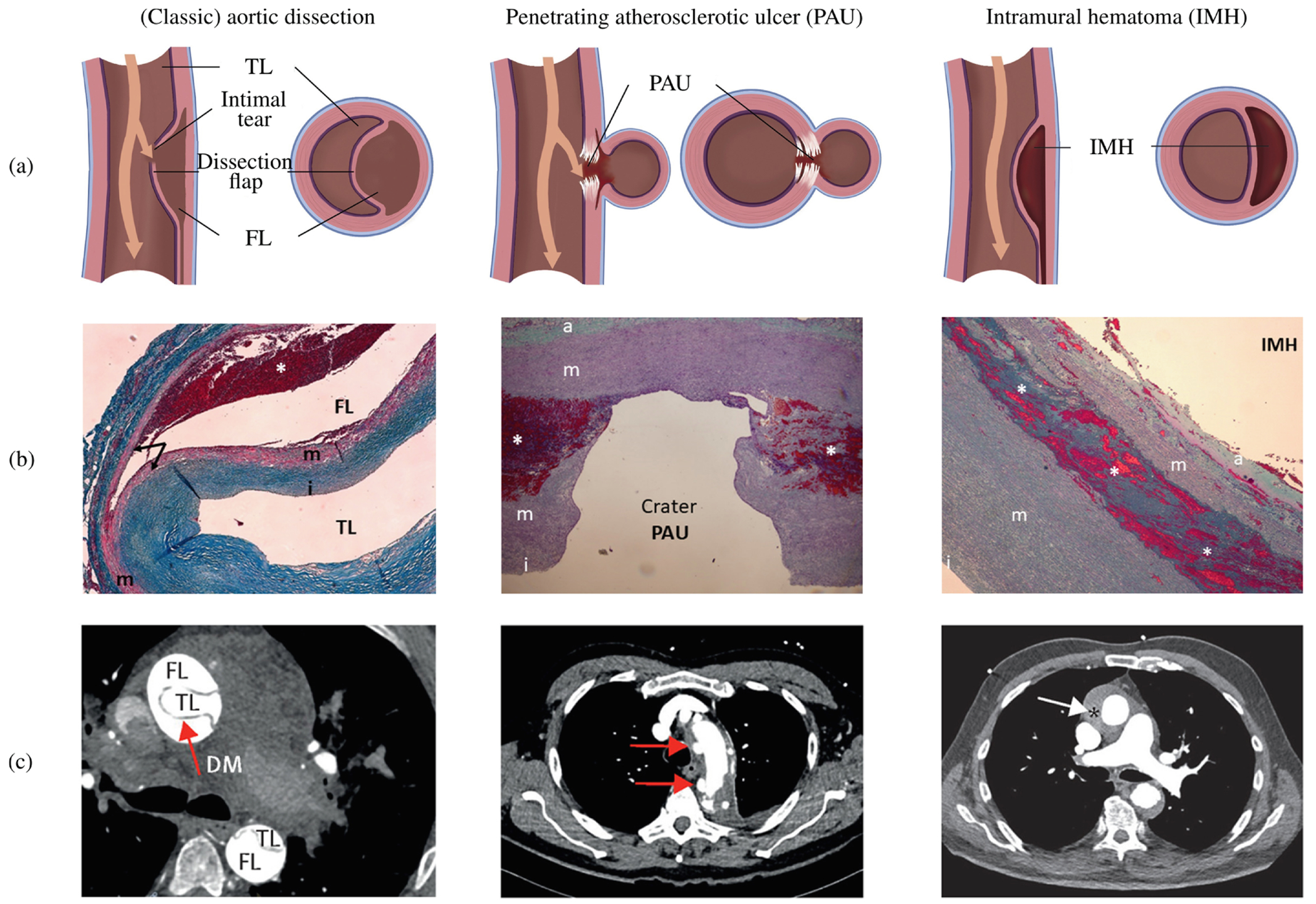
(a) Schematic overview of acute aortic syndromes and their main morphological characteristics, including (i) (classic) aortic dissection with true lumen (‘TL’) and false lumen (‘FL’), (ii) penetrating atherosclerotic ulcer (‘PAU’), and (iii) intramural hematoma (‘IMH’). (b) Histological sections of the aorta (Masson’s trichrome stain) showing (i) the ascending aorta in a (classic) aortic dissection with visible intima (blue, ‘i’), media (red, ‘m’), and false lumen (‘FL’), where arrows indicate the separation of the media forming the dissection flap and a thrombus in the false lumen (asterisk); (ii) the descending aorta in the case of penetrating atherosclerotic ulcer (‘PAU’) features an ulcerated crater, which penetrates the media and is characterized by adventitia (‘a’), intima (‘i’), and media (‘m’), with intramedial hemorrhage also detected (asterisk); and (iii) an intramural hematoma (‘IMH’) with the dissection plane within the outer aortic media (asterisks) showing layers including adventitia (‘a’), intima (‘i’), and media (‘m’). (c) Computed tomography images depict (i) an acute type A dissection with an intimal rupture and a dissection flap (here: dissecting membrane, ‘DM’), creating a true lumen (‘TL’) and a false lumen (‘FL’); (ii) a penetrating atherosclerotic ulcer in the descending aorta (red arrows) with a transmural lesion and a localized subadventitial hematoma; and (iii) an intramural hematoma in the ascending aorta (asterisk) with no visible lesion in the inner aortic layers. Figures (a) and (b) are adapted from Vilacosta et al. [128], while (c) is from Carrel et al. [129], with permission from Elsevier, respectively.

In addition to intramural hematoma and penetrating atherosclerotic ulcers, aortic dissection is the most common form of acute aortic syndrome [126]. These life-threatening conditions compromise the integrity of the aortic wall. Intramural hematoma is a contained aortic wall hematoma with bleeding within the media but without the development of an intimal tear. On the other hand, a penetrating atherosclerotic ulcer is an ulceration of an aortic atherosclerotic plaque that penetrates through the internal elastic lamina into the media. Interestingly, both intramural hematoma and aortic dissection primarily affect the media, while a penetrating atherosclerotic ulcer primarily affects the intima [127]. A schematic representation of these acute aortic syndromes, along with representative histology and CT images, is shown in Fig. 5.

According to the classic definition, an aortic dissection occurs when an intimal tear permits blood to flow through the tear and into the aortic media. This process splits the wall and forms a dissection flap that separates the true lumen from a newly created false lumen [130–132]. The dissection flap can progress antegrade or retrograde, potentially leading to several life-threatening complications. These complications may include acute aortic valve regurgitation, myocardial ischemia, acute stroke, malperfusion syndromes, or complete aortic rupture, which can ultimately lead to cardiac tamponade if the dissection occurs in the proximal aorta. Blood flowing through the false lumen may rupture back into the true lumen and create additional connections or fenestrations, the term ‘fenestra’ originating from the Latin word for ‘window’. If instead the blood in the false lumen ruptures through the outer media and adventitia, complete aortic rupture can occur.

In chronic aortic dissection, the development of an aortic pseudoaneurysm is not uncommon. This technical term refers to the dilatation of the false lumen. Because not all three layers of the aortic wall are affected, it is distinguished from a classic aneurysm. However, this terminology is rarely used clinically. Instead, ‘aneurysmal degeneration of the false lumen’ or simply ‘false lumen dilatation’ are more commonly employed for several primarily clinical reasons: typically only the entire aortic diameter is measured and used as a size criterion for surgical interventions. The clinical manifestation is more similar to that of a dissected aneurysmal aorta. Therefore, the term pseudoaneurysm alone might not be appropriate in many cases. To ensure consistency throughout this review article, we aim to use commonly accepted clinical terminology.

#### Anatomical classification.

**Fig. 6.**
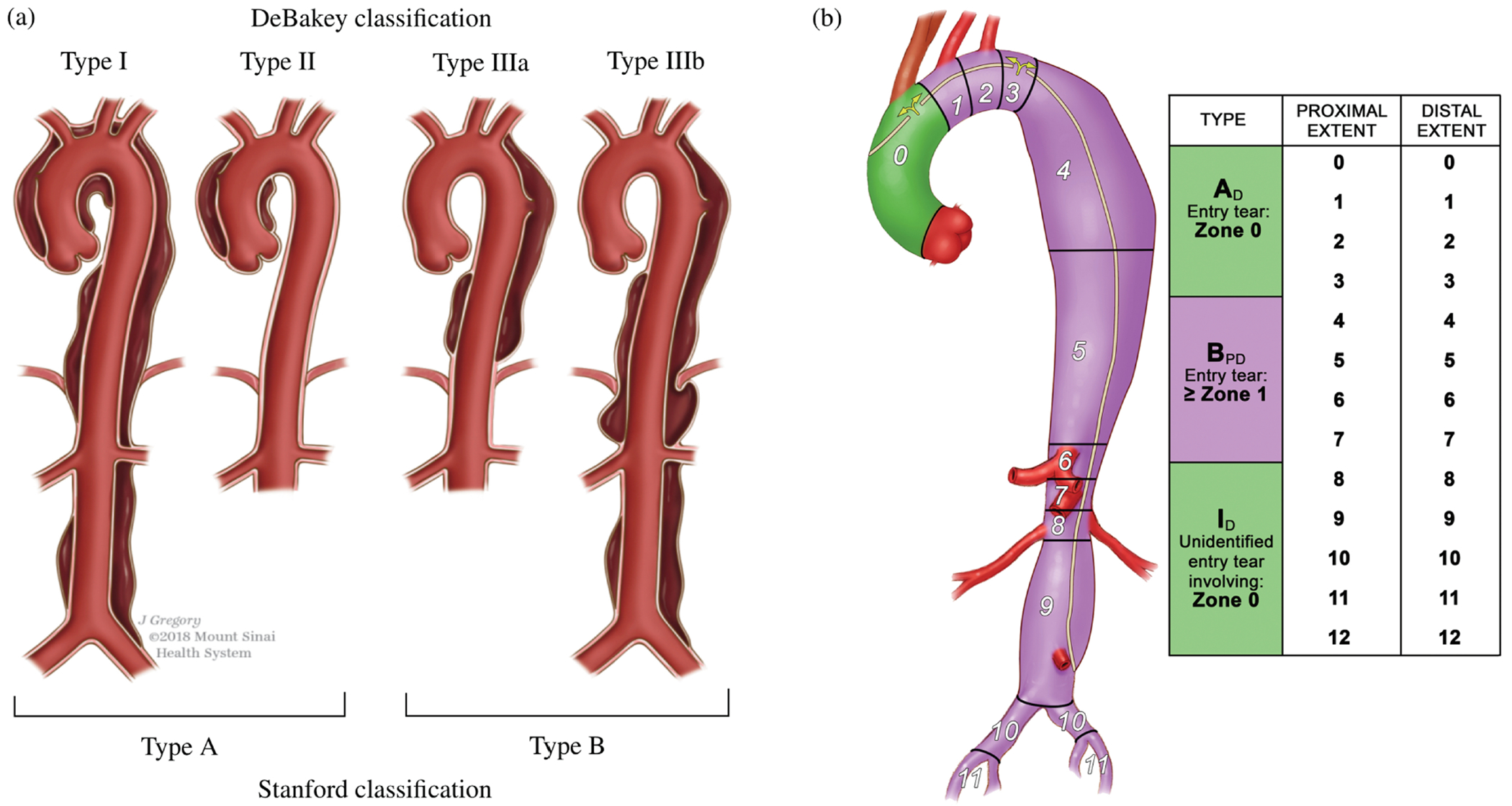
(a) Stanford and DeBakey classification systems for aortic dissection. The Stanford classification divides aortic dissections into two groups: type A and type B, while the DeBakey classification divides aortic dissections into three groups: type I (including the ascending and descending aorta), type II (limited to the ascending aorta), and type III (limited to the descending aorta). (b) Society for Vascular Surgery and Society of Thoracic Surgeons aortic dissection classification system of dissection subtype by zone location of primary entry tear [134]. In the example illustrated, the aortic dissection has a primary intimal tear in zone 0, with the dissection process extending distally to zone 9. Therefore, the dissection is fully classified as type A_9_. Figure (a) is adapted from Tadros et al. [135], while (b) is from Vacirca et al. [136], with permission from Elsevier, respectively.

Aortic dissections are traditionally classified according to the DeBakey and Stanford systems, as depicted in Fig. 6(a). The DeBakey classification system [133] consists of type I, type II, and type III, determined by the origin of the intimal tear and dissection extent. Type I arises in the ascending aorta, with the dissection propagating distally to encompass the aortic arch and usually the descending aorta. Type II is restricted to the ascending aorta, whereas type III originates distal to the subclavian artery in the descending aorta and typically extends distally. Type III dissections can be further subdivided into IIIa, which is limited to the descending thoracic aorta, and IIIb, which begins in the descending thoracic aorta and extends below the diaphragm.

The more common Stanford classification [137] simplifies the DeBakey system and divides aortic dissections into two categories based on ascending aorta involvement, regardless of the origin site. Type A includes all dissections involving the ascending aorta, irrespective of the location of the intimal tear. Type B encompasses dissections that exclude the ascending aorta, including even those that involve the aortic arch but exclude the ascending aorta.

In particular, no classification addresses aortic dissections that originate in the arch. In 2019, a third category to treat thoracic arch pathology termed ‘non-A non-B dissection’ was introduced for patients with arch involvement due to the most proximal tear or retrograde extension – in other words, the proximal dissection of the flap begins in the aortic arch [138]. Non-A non-B dissections are rare and among the most challenging cases for surgical or endovascular treatment [139].

In 2020, the Society for Vascular Surgery and the Society of Thoracic Surgeons introduced a comprehensive classification system that provides a more detailed perspective on aortic dissection anatomy [134], see Fig. 6(b). This approach characterizes dissections based on the location of primary intimal tears and the proximal and distal extent of the dissection. The updated classification scheme differentiates between type A and type B dissections based solely on the location of the primary intimal tear. This refinement was critical in accurately describing the involvement of the aortic arch in aortic dissection and in aligning with current treatment approaches. Use of this updated classification system enables clinicians to effectively envision the primary intimal tear location and the proximal and distal dissection extent through a single, concise descriptor.

There is also an alternative classification approach, still based on an anatomical system, from the European Society of Cardiology [4]. This approach comprises five classes and is derived from the initial description by Svensson et al. [140]. The classification system is based on the anatomic presentation of the dissection and includes: (i) classic aortic dissection with true and false lumina separated by a dissection flap, (ii) intramural hematoma or hemorrhage without tear or flap imaged, (iii) subtle or discrete dissection, (iv) penetrating atherosclerotic ulcers, and (v) iatrogenic or traumatic dissection. While little is known about subtle or discrete dissections due to difficulties in imaging detection, they have been described as a partial stellate or linear tear in the vessel wall without a dissection flap, eccentric bulge, or intramural hematoma.

#### Temporal classification.

Historically, aortic dissections have been classified into two groups based on the time of symptom onset: acute, defined as less than 14 days from symptom onset, and chronic, defined as more than 14 days from symptom onset. This 14-day cutoff was originally established due to the prevalence of dissection-related complications during this period and was derived in part from the work of Hirst et al. [141] in the late 1950s. Since then, however, there have been significant advances in imaging techniques and treatment options. The IRAD proposed a more nuanced classification system that includes four distinct time periods: hyperacute (symptom onset to 24 hours), acute (2 to 7 days), subacute (8 to 30 days), and chronic (over 30 days) [142]. This updated classification better reflects the different phases of acute aortic syndromes and associated mortality rates.

The most contemporary temporal classification system, proposed by the Society for Vascular Surgery and the Society of Thoracic Surgeons, similarly divides aortic dissection into four temporal types: the hyperacute phase (first 24 h) within the acute phase (up to 14 days after onset), followed by the subacute (15 to 90 days after onset) and the chronic phase (> 90 days after onset). This refined classification system helps improve prognosis and guide decision-making about the timing and types of potential interventions in aortic dissection patients [34,126,134,143]. From a prognostic perspective, acute aortic dissection is more likely to result in life-threatening complications than subacute or chronic dissection. Medical treatment of aortic dissection involving the ascending aorta typically has a poor prognosis. However, chronic dissections tend to have a relatively mild natural course compared with the acute phase [144].

#### Prognostic classification.

In contrast, prognostic classification systems offer clinicians a useful method for predicting the likely outcome or course of a condition. Perhaps the most common differentiation is to distinguish between complicated and uncomplicated dissections, irrespective of the time elapsed since the onset of dissection and the proximity of the dissected segments of the aorta. This is particularly useful for type B dissection [145]. Complicated type B dissections are characterized by the presence of a malperfusion syndrome, potentially leading to visceral, renal, or extremity ischemia. Other distinguishing characteristics include rupture, uncontrolled hypertension, persistent abdominal or chest pain, or signs of rapid expansion, which can be detected through medical imaging. The management of complicated acute type B dissections can be particularly challenging, as discussed later. In contrast, uncomplicated dissections are typically managed only through aggressive blood pressure control.

### 2.3. Imaging and treatment of patients

Imaging techniques and management strategies for aortic dissection patients play a crucial role in ensuring accurate diagnosis, appropriate risk stratification, and effective treatment. Advanced imaging techniques not only enhance our understanding of the remodeled anatomy and altered hemodynamics but also facilitate the development of *in silico* models, as we will demonstrate in Sections 6 to 8. A synopsis of the strengths and limitations of the subsequently discussed imaging modalities and their variants for clinical use and research is given in Table 1.

#### 2.3.1. Imaging modalities and variants for clinical use and research

A wide variety of imaging techniques are available for anatomic and functional assessment of the aorta, both for routine clinical use and for research applications [34,126]. The main diagnostic imaging modalities are CT imaging, MRI, and ultrasound techniques. It is important to be familiar with the basic principles of these technologies to understand their strengths, their limitations, and how the variety of modern imaging applications based on these technologies can be used to assess cardiovascular diseases, such as aortic dissection, and to inform computational models.

#### Angiography.

Classic angiography, a traditional invasive radiologic technique for imaging blood vessels, is based on the direct injection of an iodinated contrast agent into the bloodstream, either through a hollow needle or, nowadays, through a catheter inserted into the femoral artery in the groin. The contrast-opacified vessels become then visible in an X-ray image on a radiographic film, which is referred to as an angiogram. However, catheter-based angiography is no longer used for diagnostic purposes in patients with aortic disease and has been completely replaced by non-invasive imaging techniques, particularly CT angiography (CTA). Today, angiography is routinely used in catheter-based interventions such as minimally invasive procedures, percutaneous fenestrations, and other endovascular procedures.

#### Computed tomography imaging.

CT is an X-ray-based imaging technique commonly used for diagnostics, providing 24/7 availability [146–148]. The produced raw data is reconstructed into thin transverse cross-sectional slices, with these images typically measuring 512×512pixel and having a section thickness as thin as 0.5mm. A full-body CT scan, including the chest, abdomen, and pelvis, has between 900 and 1,500 slices, and its acquisition time is in the order of a few seconds. Disadvantages of CT include ionizing radiation and low contrast resolution for soft tissues. With a few exceptions, all cardiovascular CT applications require the use of an intravenously injected contrast medium to enhance the X-ray attenuation of the blood and enable visualization of the heart and blood vessels.

By far the most commonly used imaging technology to assess aortic disease is CTA, a CT scanning technique optimized for displaying vascular structures and reminiscent of conventional (catheter-based) angiography. CTA scanning combines optimal synchronization of the contrast medium injection with CT data acquisition to achieve particularly strong contrast medium opacification of the vascular structures. The resulting high-contrast, high-resolution CTA data set can be further reconstructed into specific angiographic representations, such as 3D representations of the vessels, using volume-rendering techniques.

Although modern CT scanners have impressively short ‘shutter open’ times between 65 and 150ms, these durations are still not sufficient to capture motion-free images of the beating heart or the thoracic aorta [149]. This results in images of the aortic root and ascending aorta that appear blurry, with ‘double contours’ due to the motion transmitted from the heart, potentially obscuring or mimicking disease and decreasing measurement accuracy. Consequently, CT and related techniques rely heavily on the synchronization of CT acquisition with the patient’s electrocardiogram (ECG) signal. This is commonly referred to as ECG-synchronized CT, gated CTA, gated chest, or cardiac CT, which all refer to the same imaging procedure. ECG gating is increasingly used for thoracic aortic imaging, but is only necessary when the aortic arch or descending thoracoabdominal aorta are of clinical interest.

Another related advanced imaging technique is 4D CT or 4D CTA, which typically requires prolonged exposure to radiation. This technique typically reconstructs ten sequential ‘frozen’ 3D data sets across the entire cardiac cycle, using the R-peaks of the patient’s ECG signal as a temporal reference. The ten data sets, reconstructed with time stamps between 0 and 90% (in 10% increments) of the R-R interval, can be viewed in a cine-loop. Note that the R-R interval is the time period between two consecutive R waves, typically the highest and most prominent upward deflection in an ECG representing the duration of a complete cardiac cycle. This ultimately leads to visualization of a beating heart, the motion transmitted to the aorta, as well as the cyclic diameter change (or ‘pulsation’) of the aorta and the cyclic motion and deformation of a dissection flap. 4D CTA data are clinically used in many academic or large medical centers and enable quantitative analysis of cardiac phase-dependent deformations with high spatial resolution. In addition, 4D CTA data can be used to measure the distensibility of large vessels, such as the aortic arch and the abdominal aorta, using ECG-gated CTA [150–152]. Distensibility is defined as the maximum change in cross-sectional area relative to the pressure change, scaled by the minimum area [153]. This method can also be applied to smaller vessels, like the carotid arteries [154]. However, measuring distensibility in smaller vessels becomes more challenging because the annotation error increases relative to the change in cross-sectional area.

Recently, photon-counting CT (PCCT) has emerged as an advanced alternative imaging technique and is becoming a standard clinical use in CT imaging [155,156]. Compared to conventional CT imaging, PCCT offers several advantages, including improved spatial and contrast resolution, reduced image noise and artifacts, and lower radiation exposure, resulting in an overall improvement in quantitative imaging. In addition, the high resolution could enable future investigations into the structure of the aortic wall and measurement of wall thickness along its length, which has not been possible previously.

#### Magnetic resonance imaging.

MRI is an accepted alternative to CT, but has the disadvantage of a longer acquisition time, lower spatial resolution, and typically limited availability in the immediate vicinity of an emergency room [32,157]. The advantage is that it does not require ionizing radiation and is therefore well suited for monitoring the size of the aorta, especially in young patients and in surveillance imaging. The resulting images typically have a thickness of 1 to 3mm and a pixel size of only 1 to 2mm. An MRI study protocol typically comprises several pulse sequences, with each sequence lasting from seconds to minutes, and is optimized for specific purposes. Total study time is generally between 30 and 60 minutes, but may be significantly longer in a research setting. A pulse sequence in MRI involves certain settings and instructions for the MRI device that affect the appearance of the image. It regulates the timing, strength and type of pulses used and can highlight different tissues or disease processes.

Magnetic resonance angiography (MRA) imaging, much like CTA, is most commonly performed by injecting a gadolinium-based agent. It uses pulse sequences to generate optimized vascular images. MRA data sets can be ECG-gated, but usually are not. Since the 3D data acquisition averages over many heartbeats, this results in mild blurriness of the ascending aorta, which is acceptable for clinical use except for dedicated aortic root imaging. In addition, fast MRA techniques allow multiphasic imaging, resulting in a series of MRA images every few seconds, allowing observations of vascular or tissue enhancement patterns over several seconds to a minute. However, this requires the use of specialized pulse sequences and data acquisition methods.

Cine MRI is the basic cardiac MRI technique used to assess both morphology and function. It is based on a steady-state free precession pulse sequence, which allows for the generation of time-resolved images, typically consisting of 20 frames per heartbeat, to assess the beating heart. This imaging technique is considered the gold standard for determining functional parameters such as the ejection fraction of the left ventricle. In addition to cardiac imaging, cine MRI can also be used to detect motions of the dissection flap, flow disturbance at entry and exit tears, and to distinguish flowing blood from thrombus at selected slice locations.

Phase contrast (PC)-MRI, which is based on the phase difference in moving protons, is a standard magnetic resonance sequence available on all cardiac MRI-capable scanners. The PC pulse sequence can be used to visualize and quantify blood flow in the cardiovascular system in 2D [158]. Usually, anatomical images are then overlaid with a color-coded map. PC imaging can also use the patient’s ECG signal to map the velocity measurements to the phase of the cardiac cycle, a technique called cine PC imaging. This allows the visualization and measurement of blood flow over time, i.e. over the cardiac cycle. Time-resolved (cine) 2D PC images are typically reviewed and analyzed in a cine loop of 20 frames over the cardiac cycle. This allows quantification of maximum, minimum, and average blood flow velocities, and many other flow parameters used in cardiac and vascular imaging. It can be used to measure blood flow velocities in the true and false lumina.

An extension of cine 2D PC-MRI to a 3D volume is time-resolved 3D PC-MRI, also known as 4D-flow MRI or 4D PC-MRI. With the latest acceleration techniques, such as parallel imaging, a 4D-flow MRI data set can be acquired in about 10 minutes. 4D-flow MRI, predominantly used in academic centers, is a promising technique with prognostic value for patients with aortic dissection [159–162]. It facilitates direct, non-invasive blood flow measurements within an entire anatomical volume, and contains velocities encoded in the three dimensions of space. For example, it enables the measurement of blood flow in both the true and the false lumen, blood flow across the fenestrations, and also facilitates the derivation of 4D-flow-based WSSs. However, it is important to note that the assessment of WSS is highly dependent on the accuracy of the surface reconstruction. Due to the limited spatial resolution of 4D-flow MRI, this accuracy is often quite low, leading to frequent errors [163–165]. Moreover, capturing the wide range of velocities present in aortic dissection can pose a challenge with 4D-flow MRI, as a velocity encoding (VENC) must be preselected based on the expected velocity of the target vessel. If the velocity encoding (VENC) is set too high or too low, this can lead to inaccurate measurements. One recently described potential solution is dual-VENC 4D-flow MRI, which enables more accurate measurements of a wider range of velocities.

#### Ultrasound imaging.

In ultrasound imaging, high-frequency sound waves are emitted, and the ‘echo’ reflected by tissue is received by the same ultrasound transducer. The temporal resolution of ultrasound is high, and gray-scale anatomic images are generated in ‘real time’ while the echo probe is moved over the tissue of interest. Ultrasound is a widely used technique to image the heart, commonly referred to as echocardiography (ECG) or, in short, echo. Ultrasound imaging is completely non-invasive and used for many other soft tissue imaging applications, as well as vascular imaging. The frequency of ultrasound waves is also altered when reflected by moving blood (the Doppler effect), which can be exploited to measure blood flow velocities, a technique called Doppler ultrasound. When the Doppler signal is displayed in color, for example, blue for velocities toward the probe and red for velocities away from the probe, and overlaid on a simultaneously acquired gray-scale anatomic image, the technique is referred to as color Doppler or duplex ultrasound. When the ultrasound probe is placed on a patient’s thorax to examine the heart, it is called transthoracic ECG (TTE). TTE also allows visualization of the aortic root and parts of the ascending aorta. In contrast, transesophageal ECG (TEE) provides a closer look at the heart and heart valves at higher spatial resolution. TEE is considered an invasive procedure that requires sedation or anesthesia to introduce an ultrasound probe into a patient’s esophagus. It is also used intraoperatively during procedures such as open heart or aortic surgery. Additionally, it facilitates the visualization of the aortic root and provides limited views of the ascending aorta and the aortic arch [166]. In selected patients, TTE and TEE may also be useful to evaluate the dissection flap [167], to demonstrate communication between the true and false lumina, and to assess blood flow versus thrombus in the false lumen. Sophisticated variations of the insonation pulses and signal analysis allow the generation of 3D Doppler ultrasound images, mostly used in the setting of ECG. Miniaturized ultrasound probes mounted on a catheter tip can be used to generate ultrasound images and even Doppler signal from inside a blood vessel to examine the vessel wall, known as intravascular ultrasound. This is an invasive procedure and is always used in conjunction with another catheter-based diagnostic (angiography) or interventional procedure, for example, to guide endovascular procedures such as intravascular ultrasound-guided fenestration of the dissection flap.

#### 2.3.2. Current treatment strategies

All patients with acute or chronic dissection are medically managed, with the primary goal to control blood pressure. In addition to medical management, open surgical intervention involving the replacement of a segment of the dissected aorta with a synthetic graft, or endovascular surgery with the placement of a stent-graft, also called an endograft, into the diseased aorta may be necessary. The current indications are based on the 2022 ACC/AHA guidelines [126], which differ slightly from the 2014 ESC guidelines [168]; some of which are summarized here [169].

#### Medical (conservative) management.

Non-invasive therapy of aortic dissection consists of pharmacologic blood pressure control, typically with beta-receptor blockers. The rationale for reducing blood pressure in patients with acute dissection is halting disease propagation, and reducing the risk of aortic rupture. Pain medication is often required in the acute phase as well. In the chronic phase, lowering blood pressure reduces wall tension and may thus reduce risk and delay the formation of a false lumen dilatation and delayed rupture.

#### Open surgical repair.

Open surgical repair may be necessary for patients in various situations. Classic type A repair is necessary when patients with acute type A dissection require urgent surgery to replace the dissected ascending aorta with a synthetic graft to prevent rupture. The procedure requires a sternotomy and placing the patient on cardiopulmonary bypass, similar to other open heart surgeries such as a coronary artery bypass graft. In addition to the ascending aorta replacement, the procedure may require concomitant repair or replacement of the aortic root, which includes the aortic valve and the coronary artery ostia. An ascending replacement may also include a distal extension along the undersurface of the aortic arch, a procedure known as a hemi-arch procedure. Open surgical repair may also be necessary when a residual dissection remains after type A repair, meaning when an aortic dissection persists following initial treatment. In the majority of patients with type A dissection (~90%), the dissection extends beyond the ascending aorta into the arch and descending aorta. This residual dissection distal to the ascending aortic graft is typically managed conservatively, and requires lifelong surveillance of the dissected aorta. Finally, descending aortic repair, or open surgical repair of the descending thoracic aorta, remains a treatment option for patients with complicated type B dissection when the anatomy is not suitable for endovascular repair. Also, in patients with the Marfan syndrome or other connective tissue disorders, open surgical repair is the preferred option to avoid anchoring of an endograft in potentially fragile aortic tissue.

#### Endovascular surgery.

The principle of endovascular therapy for aortic diseases consists in the placement of a stent-graft into the aorta through a small groin incision and through sheath and catheter introduction into the common femoral artery. A stent-graft consists of graft material, typically a fabric like polyester, mounted on a metal frame, known as stent, which can be crimped down enough to fit through the delivery system and then expand to its desired diameter within the aorta. A stent-graft is placed in an angiography suite, or in a hybrid operating room under fluoroscopic and angiographic guidance. Thoracic endovascular aortic repair (TEVAR) is the first-line therapy for patients with complicated type B dissection. The technical goal is to anchor the endograft in a non-diseased segment of the aorta proximal to the dissection, referred to as the landing zone. Subsequently, the endograft is expanded in the true lumen of the aorta to cover the primary entry tear and re-expand the true lumen. The distal extent usually covers at least half of the descending thoracic aorta or more. Ideally, the false lumen thromboses completely and the dissection heals. In most cases, however, a distal portion of the dissection remains perfused, mandating life-long surveillance and follow-up.

**Fig. 7.**
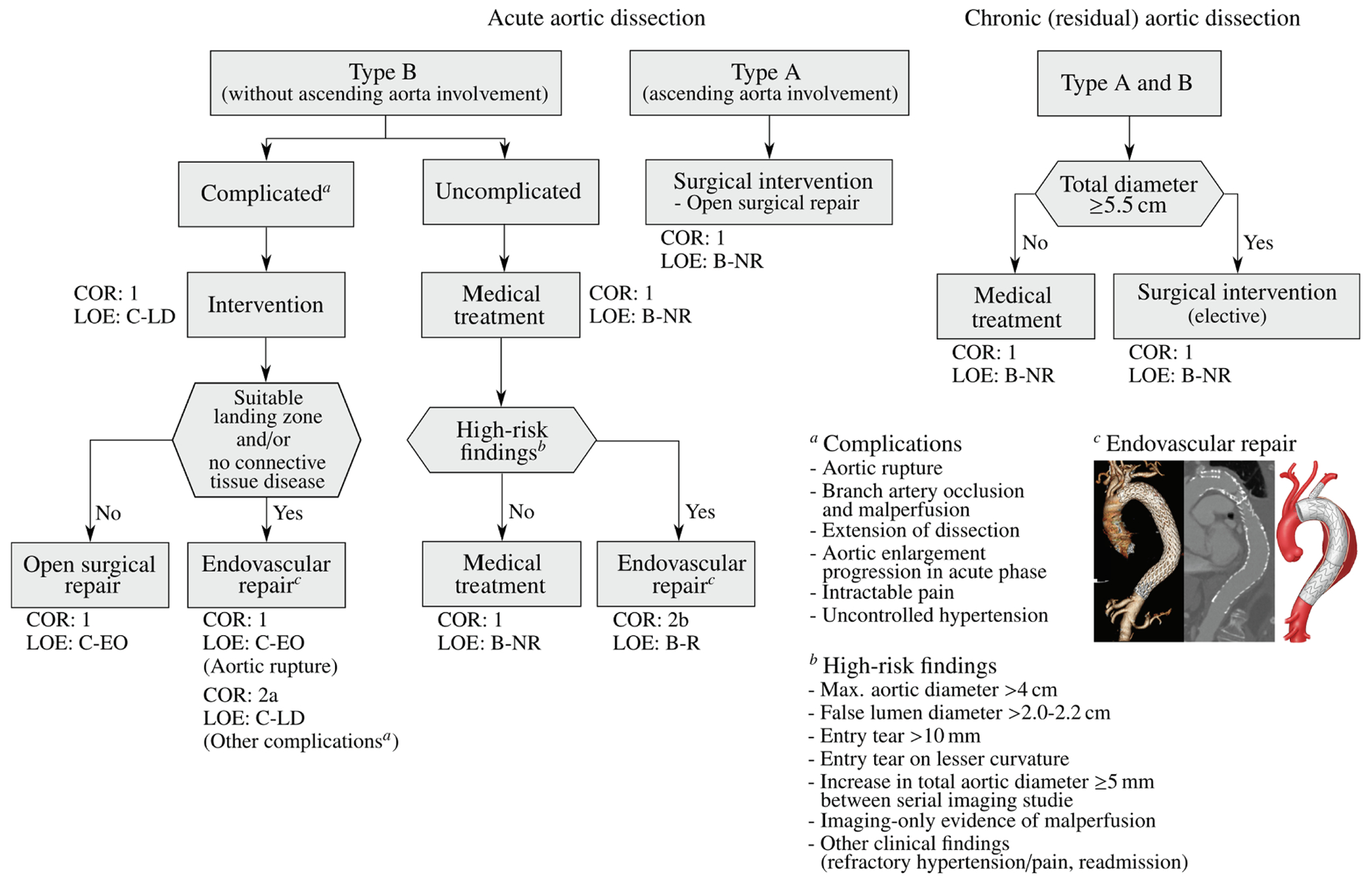
Key synopsis of treatment strategies for aortic dissection, including the class of recommendation (‘COR’) and level of evidence (‘LOE’), based on the 2022 ACC/AHA guidelines [126]. The figure showing endovascular repair is adapted from Vacirca et al. [136] with permission from Elsevier.

Endovascular technology is rapidly evolving and a growing number of patients have become anatomically suitable even if the proximal landing zone would be considered too short for a descending thoracic aortic endograft. Indications for TEVAR are also evolving, with the main controversy related to the treatment of patients with uncomplicated type B dissection (Fig. 7). While TEVAR has the potential to prevent or delay late adverse events, only a subset of patients with uncomplicated type B dissection are likely to benefit from the procedure, which is not without its own procedural risks.

Despite being minimally invasive, endovascular surgery can involve several complications. One common issue is an endoleak, where blood leaks into the dilated aorta outside the stent-graft, potentially causing persistent or recurrent dilatation and possibly requiring further procedures to correct. Another complication is the migration of the stent-graft from its intended position. Additionally, endografts can cause new intimomedial injuries, a condition known as stent-graft-induced new entry (SINE) or distal SINE (dSINE).

#### Hybrid procedures.

Hybrid procedures refers to the combination of open surgical repair with endovascular techniques. Consider a patient with a type A dissection who might undergo open surgical repair of the ascending aorta and the entire aortic arch, while the surgeon simultaneously inserts a stent-graft into the true lumen of the descending thoracic aorta. Another example involves the placement of an open surgical left carotid to left subclavian artery bypass graft. This procedure subsequently allows an aortic endograft to be landed in the aortic arch, covering the subclavian artery ostium. It thus facilitates an endovascular treatment option for an individual with an inadequate landing zone in the descending thoracic aorta.

#### Treatment strategy.

All patients with aortic dissection are medically managed. The selection of open and endovascular interventions depends predominantly on the anatomy involved (type A versus type B dissection), and on the presence or absence of acute complications (in type B dissection). All patients who survive an aortic dissection remain at high risk for late adverse events and require lifelong specialized care and surveillance imaging to monitor the size of the dissected aorta. See the key synopsis of treatment strategies for aortic dissection, including the class of recommendation and level of evidence based on the 2022 ACC/AHA guidelines [126].

#### Alternative treatment concepts.

**Fig. 8.**
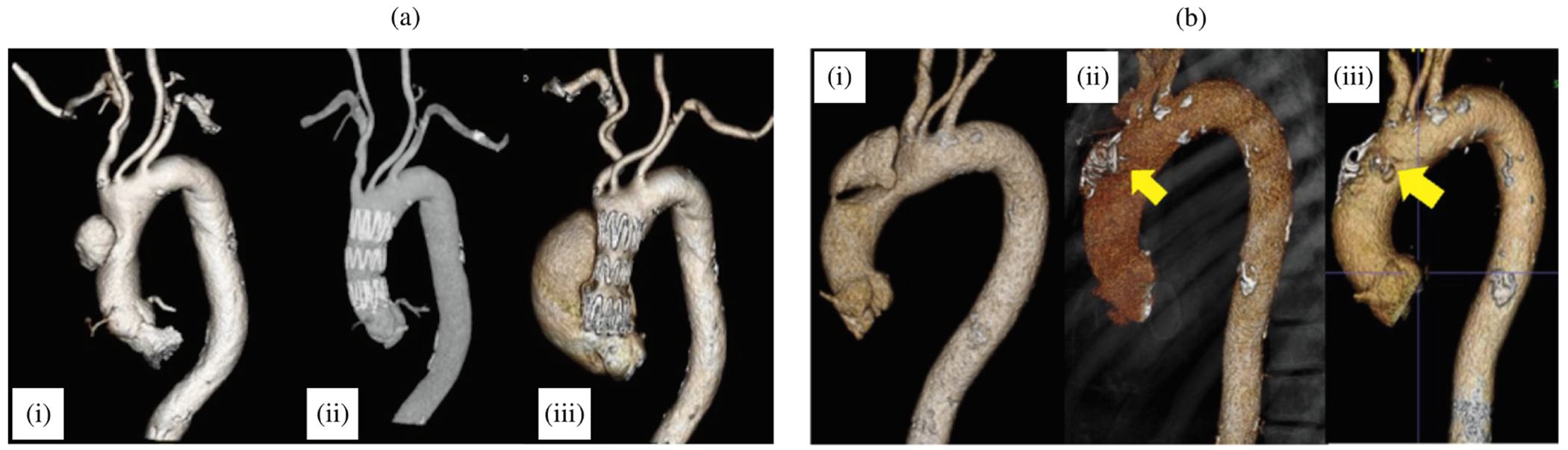
(a) A representative patient case of late complications after TEVAR in ascending aortic dissection with (i) a localized type A dissection, (ii) excellent results after TEVAR with a short stent-graft, and (iii) a stent-induced re-entry tear (erosion) from the proximal contact between the crown of stent-graft and the out-curve of the aorta after six months. (b) A representative patient case for a false lumen intervention to promote remodeling and thrombosis (FLIRT) procedure, showing (i) a depiction of a localized type A dissection with an entry tear at the outer curvature of the aorta just proximal to the innominate artery, (ii) the result of endovascular treatment with coils inserted into the false lumen to promote thrombosis and a patent for a men ovale occluder (arrow) to isolate communication between the true and false lumina, and (iii) a complete remodeling of the aorta without any complications after two years of surgery. Figure is adapted from Yuan et al. [170] (licensed under CC-BY 4.0).

In addition to the primary treatment options recommended by the 2014 ESC [168] and the 2022 ACC/AHA [126], there are alternative options for patients who are at extremely high-risk for open surgery, such as elderly patients. According to the key synopsis of treatment options for aortic dissection based on the 2022 ACC/AHA guidelines (shown in Fig. 7), open surgery remains the primary treatment for type A dissections. This preference is due to the complex technical and anatomical challenges posed by endovascular therapy in the ascending aorta [170]. These challenges include the dynamic motion of the ascending aorta, the proximity of vital structures to the TEVAR landing zone such as the aortic valve, coronary arteries and supra-aortic branches, and the short and wide shape of the aorta. Furthermore, the lack of specially designed endografts further complicates endovascular approaches. These factors make endovascular approaches less viable and reinforce the preference for open surgery in these cases. Because TEVAR has been used in a small number of patients with type A dissection, clinical data are limited. In fact, 10 to 30% of patients are not accepted for surgery, making them potential candidates for TEVAR [171]. Furthermore, it has been shown that up to 50% of patients with type A dissection are technically suitable to TEVAR [172,173]. However, the outcome of TEVAR also depends on the location of the tear. A tear in the center of the ascending aorta is more beneficial for endovascular treatment than a tear near the aortic valve. One of the most common complications after stent-grafting is endoleak, which indicates inadequate sealing of the endograft and can lead to unfavorable aortic remodeling. This is illustrated in Fig. 8(a), where the TEVAR procedure resulted in a so-called SINE followed by significant aortic dilatation.

Another alternative treatment method is false lumen intervention to promote remodeling and thrombosis, abbreviated as FLIRT [170]; the interested reader is also referred to [174]. This treatment option has so far received little attention. FLIRT is based on minimalist, percutaneous interventions with occluder devices, vascular plugs, distal stent-grafts, or coils and glue, applicable to both chronic type A and B dissections. These occluder devices are used to cover the primary intimal tear, which helps create favorable hemodynamic conditions that promote remodeling and subsequent false lumen thrombosis, as shown in a representative patient case in Fig. 8(b).

Finally, the multilayer flow modulator should be briefly introduced, especially due to its recent interest in *in silico* modeling, as shown later in Section 6. The multilayer flow modulator is a device consisting of interconnected wires forming a mesh with a specific porosity of 60 to 70%, tailored for applications such as aneurysm or dissection repair [175]. The design of the multilayer flow modulator directs and laminates blood flow along the vessel wall, aligning it with systemic pressure flow to eliminate turbulence and vortices. Vortices, in particular, are dangerous because they are known to increase peak stresses in the wall and alter viscoelasticity, plasticity, and cellular wall activity in aneurysms, resulting in damage at specific regions in the dilated aortic wall. Unlike traditional stiff stent-grafts, the multilayer flow modulator’s compliance closely matches the native aorta, reducing peak stresses in the wall and minimizing complications such as stent-graft migration and aortic neck dilatation due to rapid endothelialization. While there is ongoing debate about its long-term impact on aortic aneurysms and dissections, the multilayer flow modulator has shown its technical feasibility and acceptable survival rates [176]. However, it remains controversial whether treatment with the multilayer flow modulator affects the natural history of aortic aneurysms and dissections [177–180].

### 2.4. Congenital, hereditary, and other related risk factors

The majority (~80%) of thoracic aortopathies – dilatation, dissection, and rupture – tend to present sporadically in men and women, with hypertension and vascular aging among the most common risk factors. Hypertension is defined as a sustained elevation of blood pressure. Although hypertension initially increases circumferential wall stress, this stress tends to be restored toward normal via mechanobiological responses by the cells of the wall (endothelial, smooth muscle, fibroblasts, and resident macrophages) that thicken the medial and adventitial layers [181,182]. Because functional elastic fibers are produced primarily during development, this thickening necessarily results largely from an increase in mural collagens, SMC mass, and PGs, which by definition alters the otherwise finely balanced composition and properties of the wall. In this way, the homeostatic tendency to reduce wall stress can nevertheless compromise aortic function and, in some cases, compromise its structural integrity [79,183]. Noting that hypertension results in a structural stiffening along the entire aorta, this global effect increases the aforementioned pulse wave velocity, which, in turn, causes earlier reflections of the pressure wave from distal sites and, thus, an earlier return of the reflected waves that augment proximal pulse pressure. In this way, hypertension not only alters the properties of the proximal thoracic aorta, it also increases the hemodynamic load on the proximal segment, which appears to increase its vulnerability to aortopathy. In this case, therefore, local mechanobiological responses result in global pathological consequences, thus driving a potentially positive feedback loop that can promote disease progression [184,185]. Albeit via different cell mediated mechanisms, vascular aging also results in local changes in composition and mechanical properties [186,187], with increasing central artery stiffness also resulting in proximal pressure augmentation [188].

By contrast, the remaining (~20%) presentations of thoracic aortopathy stem from either congenital or hereditary causes. The primary congenital defect predisposing to thoracic aortic aneurysm is bicuspid aortic valve, in which the valve has two rather than the normal three leaflets. It is widely thought that the associated perturbations in hemodynamic loading on the aortic root and ascending aneurysm contribute to the progressive mural deterioration that characterizes these aortopathies ([189]; also see the discussion of computational fluid mechanics in the following sections). The list of genetic mutations predisposing to hereditary thoracic aortopathies continues to grow (Table 2), with the first mutation discovered in 1988. Advances in genomics, transcriptomics, and proteomics, as well as the development of mouse models of the human mutations, continue to yield increasing insight into the underlying biological and mechanical mechanisms responsible for both the pathogenesis and the progression of disease, but there remains a pressing need to connect molecular mechanism and mechanical failure.

As noted throughout this review article, the aortic wall consists of a complex collection of multiple cell types and myriad (~100) intramural proteins, glycoproteins, and PGs. In 1988, the first connective tissue disorder definitively associated with arterial disease, including thoracic aortopathy, implicated mutations to *COL3A1* [192], the gene that encodes the individual alpha helices that constitute the collagen III molecule. Noting that type III collagen is the second most abundant collagen within the aortic wall, found primarily in the medial layer, defects in collagen III render the wall fragile, susceptible to dissection and rupture [213]. The associated syndrome is known as vascular Ehlers-Danlos syndrome, which is rare, with an incidence of one in 50,000 to 200,000 live births. A mere three years later, it was reported in three simultaneous studies that mutations to the gene *FBN1* that encodes fibrillin-1 predispose to thoracic aortic aneurysm and subsequent dissection and rupture [196,214,215]. The associated syndrome is known as Marfan syndrome, which is also rare but not uncommon, presenting in approximately one out of 5,000 live births. Importantly, the ECM glycoprotein fibrillin-1 associates with elastin to form the elastic fibers of the medial lamellar units; it also exists as microfibrils that connect the SMCs to these laminae. Although it was initially thought that fibrillin-1 promoted elastogenesis (i.e. the *de novo* formation of elastic fibers), early mouse models of Marfan syndrome suggested that disrupted fibrillin-1 affects the structural stability of the elastic fibers [216], reducing their otherwise long half-life (which is estimated at 50+ years for normal elastic fibers). Recalling again that functional elastic fibers appear to be produced and organized only during aortic development, reducing the structural stability of these fibers has dire consequences, both accelerating its fatigue considerably and resulting in elastic fragments that promote proteolytic activity. Of course, compromising elastic fiber integrity also diminishes the primary mechanical function of the aorta, to store elastic energy during systolic distension and to use this energy to work on the blood during diastole to augment antegrade and retrograde blood flow [217]. Although fibrillin-1 mutations affect all segments of the aorta, disease first manifests at the aortic root and proximal ascending aorta, the region most susceptible to dissection and rupture in Marfan syndrome, again likely accelerated in part by proximal pulse pressure augmentation [218].

In contrast to these direct connective tissue disorders (*COL3A1* and *FBN1* mutations), well over a decade later it was discovered (in 2005) that mutations to genes (*TGFBR1*, *TGFBR2*) that encode receptors of the transforming growth factor-*β* (TGF-*β*) system also predispose to thoracic aortopathies [197,219]. The cytokine TGF-*β* has diverse effects on cell function, including synthesis of ECM constituents and smooth muscle contractile proteins, which play critical roles in aortic development, homeostasis, and remodeling. Importantly, subsequent studies further identified other mutations within the TGF-*β* system that predispose to thoracic aortopathies, including mutations to ligands (*TGFB2*; [203,204], and similarly *TGFB3*; [220]) and downstream signaling species (*SMAD3*; [202,221], and similarly *SMAD4*; [205]). TGF-*β* is a highly mechano-regulated cytokine – increases in wall stress can drive both its synthesis [222] and its activation within the ECM from the latent form in which it is secreted [223]. There are also documented interactions between TGF-*β* and integrin signaling [224].

At about the same time that TGF-*β*-opathies were discovered, it was also found (in 2006 and 2007) that mutations to genes that encode intracellular contractile proteins also predispose to thoracic aortic aneurysms and subsequent dissections [198,200]. The first two causative mutations were in *MYH11*, the gene that encodes smooth muscle myosin heavy chain, and *ACTA2*, the gene that encodes smooth muscle alpha-actin. Additional contractile related mutations were discovered soon thereafter [201,208]: loss-of-function mutations to *MYLK*, the gene that codes myosin light chain kinase, and gain-of-function mutations to *PRKG1*, the gene that codes a cyclic guanosine monophosphate-dependent kinase; both predispose to thoracic aortopathies, noting that these two mutations similarly serve to reduce smooth muscle contractility. Although mutations to these four genes first implicated diminished vasoconstriction secondary to reduced smooth muscle contractility, it is important to note the following. First, the primary role of the SMCs of the aorta is to establish, maintain, and remodel the ECM of the medial layer, not to vasoregulate the lumen; second, actomyosin activity is also central to other cellular functions, including migration and mechanotransduction. Interestingly, mutations to the gene (*FLNA*) that encodes the actin cross-linker filamin-A also appear to predispose to thoracic aortopathies [225].

Finally, note that mutations to the gene (*LOX*) that encodes lysyl oxidase, a potent cross-linker of newly deposited elastic and collagen fibers, have also been found to predispose to thoracic aortopathies [210,211]. Consistent with this discovery, it has been known since the 1960s that the lathrogen *β*-aminopropionitril, which blocks lysyl oxidase, also gives rise to aortic dissections and rupture [226,227], thus emphasizing further the overall importance of ECM integrity, which is primarily but not exclusively dictated by competent elastic fibers and fibrillar collagens.

#### Dysregulated mechanosensing and mechanoregulation.

Given that many dissections occur secondary to aneurysms, it is instructive to realize that aneurysms arise similarly in cases wherein genetic mutations affect ECM, mechanosensitive signaling pathways, or intracellular contractile proteins (Table 2). Importantly, these three classes of mutations affect components along what is known as the mechanotransduction axis – that is, the axis that enables cells to use actomyosin machinery and transmembrane protein complexes to assess their local mechanical environment. It has been suggested, therefore, that thoracic aortic aneurysms and their sequelae result, in part, from compromised mechanosensing and mechanoregulation of the ECM that otherwise endows the wall with its mechanical functionality and structural integrity [228]. Diverse aspects of this failed mechanobiological process in the context of thoracic aorta aneurysm, dissection, and rupture are discussed in related papers [78,229–233].

Briefly, mechanobiology is the study of biological responses to mechanical stimuli, which ultimately depend on three cell activities – transduction, transcription, and translation. Translation refers to the actual production of gene products based on the associated gene expression (or transcription), but within this context often includes processes that also affect post-translational modifications, including cross-linking. Importantly, it appears that cells do not merely synthesize and secrete the extracellular proteins that contribute to the mechanical integrity of the ECM, they also work on and organize (i.e. mechano-regulate) this matrix to endow it with desirable properties that affect stiffness and strength. Such work requires actomyosin activity. A cell’s ability to assess external mechanical stimuli (i.e. mechano-sense) requires both transmembrane protein complexes (i.e. clusters of integrins that are referred to as focal adhesions) to couple the intracellular cytoskeleton to the ECM and actomyosin activity to enable the cell to probe or assess the mechanical state of the ECM [234,235]. Transduction of these mechanical stimuli occurs both directly and via second (chemical) messengers, both typically resulting in altered gene expression (transcription). Although it has been suggested that cells sense continuum concepts of stress or strain, or metrics derived from them, this is unlikely [236]. Rather, the cells likely sense conformational changes in mechanosenstive molecules within the cell membrane, the cytoskeleton, or the nucleus, or a combination of all three. Nevertheless, it has proven convenient to correlate mechanosensitive biological responses, including altered turnover of cells and matrix, with continuum level quantities such as stress, strain, and stiffness, as this facilitates computational modeling [237].

Even under normal quiescent conditions (i.e. homeostatic), the ECM of the aorta is nevertheless undergoing continual turnover, that is, there is continual production of matrix to replace that which is removed consistent with the finite half-life of all biological materials and cells. So-called growth (changes in mass) and remodeling (changes in structure) can thus be modeled mathematically given three classes of key constitutive relations, those for the production, removal, and material properties of individual structurally significant constituents [237], which in the normal aorta includes elastic fibers and fibrillar collagens. That one must track different constituents, having different rates of turnover and different mechanical properties, naturally suggests the use of a continuum theory of mixtures [237]. Indeed, recalling that mutations to genes that affect diverse ECM components or contribute to their integrity, ranging from fibrillin-1 to lysyl oxidase, reminds us that modeling the evolving biomechanical properties increasingly demands greater consideration of the many constituents that contribute to structural integrity, not just the elastic and collagen fibers. Similarly, noting that this integrity ultimately stems from gene products that result from complex cell signaling, there is clearly a need to pursue multiscale modeling that couples transcriptional changes with tissue-level manifestations (cf. Irons et al. [238]).

#### Other strong risk factors.

As noted above, two of the strongest risk factors for aortic dissection are vascular aging and hypertension. Much has been written on the effects of both of these risk factors on the aorta, hence the interested reader is referred to related review articles, see, e.g., [185,188]. Of particular note, albeit for different reasons, both of these conditions tend to increase central artery stiffness, which increases pulsatile loading on the proximal thoracic aorta that is most vulnerable to dissection, namely type A dissection. This increase in loading stems from an increase in the pulse wave velocity, which depends on the material stiffness of the wall as well as its thickness. Hypertension tends to drive a thickening of the aortic wall in large part due to an increase in collagen accumulation. Vascular aging similarly tends to include increased accumulations of collagen fibers and GAGs, though also with a progressive loss of elastic fiber integrity. As a result, aging affects aortic distensibility [239] as well as biaxial mechanical properties [186,240].

Another situation of critical importance to understanding the risk of aortic dissection is pregnancy. Again, much has been written and the interested reader is referred to multiple review articles [241–245]. There is, however, a dearth of information on changes in the mechanical properties of the aorta during pregnancy, particularly in cases where a mutation exists (e.g., Marfan or Loeys-Dietz syndromes) that predisposes to aortic dissection. As noted below, mouse models can be useful in studying mechanisms, and this is a pressing need with regard to biomechanical changes during pregnancy. Finally, it is noted that the risk of aortic dissection can also remain high for the mother after birth, with a study in mice suggesting that it is increased oxytocin (which drives uterine contractions and lactation) that increases risk [246]. Much remains to be learned, however.

## 3. Pathological changes associated with aortic dissection

The pathological changes associated with aortic dissection significantly alter both the aortic anatomy and the microstructure of the wall. These modifications lead to changes in mechanical behavior and hemodynamics, potentially resulting in thrombus formation within the false lumen. Some of these changes may not be immediately evident but become more pronounced as the condition becomes chronic.

**Fig. 9.**
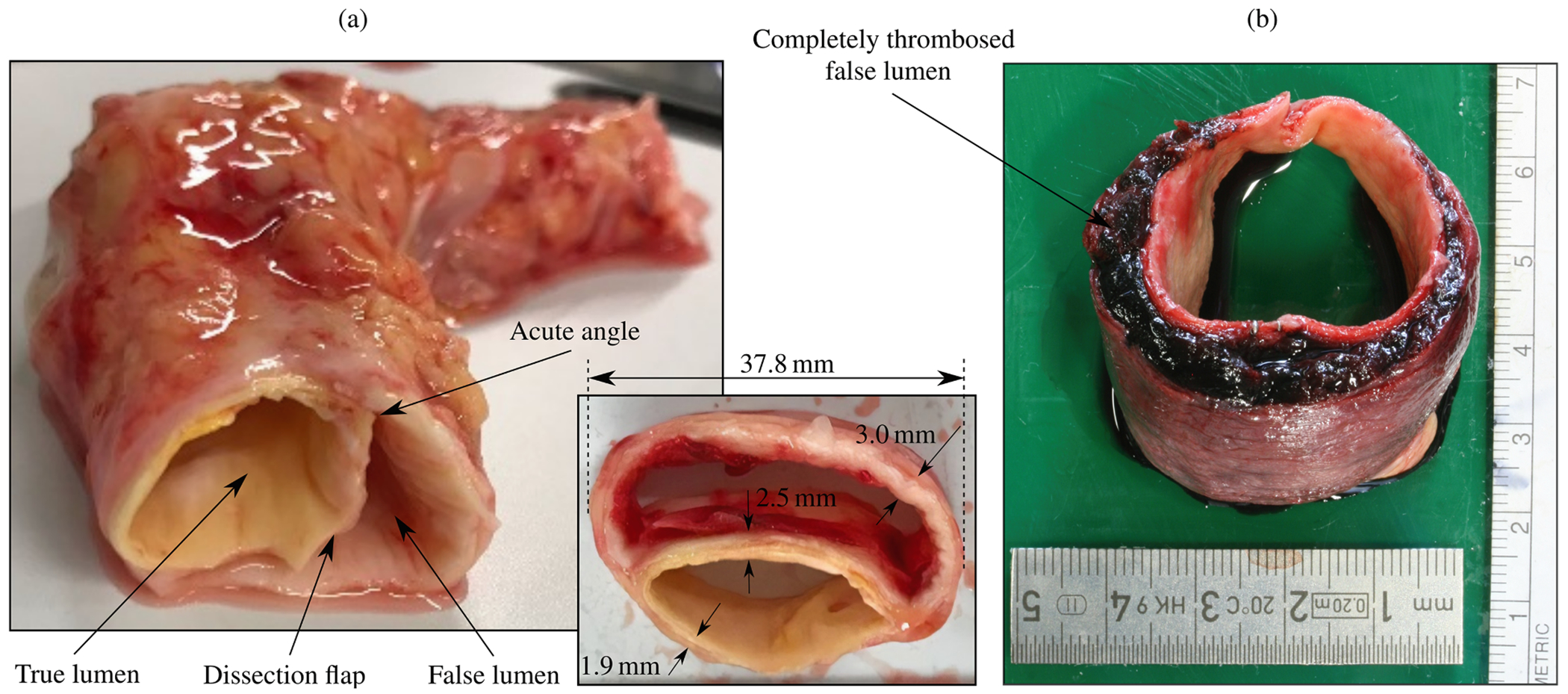
Image of harvested tissue samples from patients with aortic dissection: (a) remodeled thoracic aorta showing the true and the false lumen and (b) cross-sectional ring sample with a thrombosed false lumen. Figures (a) is adapted from Amabili et al. [250] with permission from Elsevier, while (b) is unpublished data provided by S. Sherifova (Institute of Biomechanics, Graz University of Technology, Austria).

Two representative tissue samples harvested from the thoracic aorta of a patient with aortic dissection are shown in Fig. 9. During the event of an acute dissection, the dissection flap is visible, separating the true and false lumina and usually comprising the intima and part of the media. The acute angle formed where the dissection flap and the outer wall of the false lumen intersect is typically sharp in acute aortic dissection. However, local thrombus formation and neointimal growth can cause this angle to become less defined, or, in other words, result in a more obtuse configuration. This will also be demonstrated later in Section 4.1 (Fig. 35(b)). The acute angle, also known as ‘beak sign’ [247–249], appears at the lateral, distal, and proximal ends of the false lumen. This edge, which consists of the borders of the outer wall and flap, may be thrombosed. As the cylindrical ring extracted from an aortic dissection case in Fig. 9(b) shows, the false lumen can be entirely thrombosed. Curiously, the outer wall shows significant pathological changes. Over time, chronic aortic dissection may result in remodeling of the microstructure of the wall and the anatomy, depending on the individual patient case.

### 3.1. Pathophysiology of aortic remodeling

An aortic dissection is associated with a remodeled anatomy that can include various characteristics. One of these features is the formation of a dissection flap, which is typically fenestrated by one or more intimal tears. In addition, the anatomy of the aorta can remodel over the course of the disease, leading primarily to dilatation, but also to axial growth, an altered angulation, and an increased tortuosity. However, the implications and potential impact of these remodeled anatomical features on the initiation and progression of aortic dissection, which may be interrelated, are not always obvious.

#### 3.1.1. Extent and configuration of the dissection flap

The dissection flap, a characteristic feature of aortic dissection, separates the true from the false lumen, and is often referred to by various terms in the literature, such as ‘dissection septum’ [251], ‘intimal flap’ [252], or ‘dissection membrane’ [253]. Although these terms share the same meaning, some may not be entirely accurate. As Berguer et al. [254] pointed out, the term intimal flap is misleading because it implies involvement of only the intimal layer. This terminology is inaccurate, as we will demonstrate later. The dissection flap is typically fenestrated by a single or multiple intimal tears, which can vary in location and size.

#### Location, number, and size of intimal tears.

The intimal tear connects the true and false lumina, enabling blood flow between the two lumina. In the literature, various terms are used to describe this characteristic feature, such as ‘entry’ and ‘re-entry’ tears, ‘primary’ or ‘secondary’ tears, ‘entry’ and ‘exit’ tears, ‘fenestrations’, ‘connections’, or a combination thereof. However, distinguishing between entry and re-entry tears is often challenging. A significant number of aortic dissection patients present multiple intimal tears, adding complexity to the classification. While some of these terms may not be entirely incorrect, they can imply specific pathophysiological sequences, including intimal tear creation, blood entering the media, cleavage plane or false lumen formation within the wall, and intramural hemorrhage decompression back into the true lumen. Recently, Czerny et al. [138] suggested replacing the terms ‘multiple entries and re-entries’ with ‘most proximal tear’, ‘communications between lumina’, and ‘most distal tear’, in addition to the term ‘primary entry tear’. Despite this proposal, the classification of intimal tears remains contentious and unresolved [138,141,255,256]. In this review article, we aim to maintain consistent and clear notation with respect to the intimal tear.

The location of the intimal tear can be identified using various medical imaging methods, see Section 2.3.1, the most common being CT or MRI [257], but in many cases determining the exact location has proven challenging [258–260]. Studies have shown that intimal tears are most commonly located in the initial few centimeters of the ascending aorta and just distal to the left subclavian artery in the descending aorta, also called aortic isthmus [258]. In addition, intimal tears can be located proximally to the brachiocephalic artery in the ascending aorta, within the aortic arch or proximal to the celiac or renal arteries, as well as proximal to the iliac arteries [137,257,261–264]. Cuellar-Calabria et al. [265] reported on the most frequent location of the distal tear in 72 chronic type B and repaired type A dissection patients: the tear was most commonly found in a single iliac artery (56%), followed by the abdominal aorta (22%), with bilateral iliac distal tears observed in 18% of the cases. Others stated that the location of the intimal tear was related to either the convexity or concavity of the aorta [266] and found that 48% of intimal tears occur at the convexity (the outer circumference of the aorta) and at the concavity (the inner circumference of the aorta) in 52%. Nonetheless, Roberts [261] acknowledged numerous exceptions and noted that intimal tears typically involve the right lateral aortic wall along the aortic convexity, including the ascending aorta, the aortic arch, and the descending thoracic aorta. Dziodzio et al. [267] argued that an intimal tear proximal to the ligamentum arteriosum, at the aortic concavity, is relatively rare. In contrast, Peery [268] and Shennen [269] argued that the aortic wall is weaker and more degenerated at the ligamentum arteriosum’s insertion point, rendering it more susceptible to tearing. This increased vulnerability is attributed to the hinge-like motion at the junction between the aortic arch and the descending aorta during each pulsation, which is caused by the ligamentum arteriosum’s location between the relatively free aortic arch and the relatively fixed descending aorta. These conflicting viewpoints illustrate that the precise location of a potential future intimal tear cannot be predicted with absolute certainty.

The number and sizes of intimal tears, in addition to their locations, play a significant role in the progression of aortic dissection, specifically the extent of the dissection flap and how it is treated medically. Various studies have examined the average number of intimal tears present in each aortic dissection patient. Khoynezhad et al. [263] discovered up to ten intimal tears along a single aorta using CT imaging, with an average of 2.8±2.11 intimal tears per patient. Similarly, Quint et al. [256] identified up to seven intimal tears along a single aorta and an average of 2.48 intimal tears. In contrast, some studies have identified cases in which no intimal tear was detected. This supports the hypothesis that an intramural hemorrhage may precede aortic dissection, as we will discuss in Section 3.2.1. As an instance, Hayashi et al. [258] reported an absence of intimal tears in 5% of aortic dissection patients. Ergin et al. [257] found similar results while examining type A and B dissections. They consistently identified intimal tears in type A dissection cases, but the intimal tear was absent in 5 to 10% of type B dissection cases.

Each patient is unique, and despite advances in imaging techniques, detecting all fenestrations remains challenging. Besides the intimal tears visible in the dissection flap using, *inter alia*, CT imaging, it is not uncommon for numerous additional tears, known as natural fenestrations, to be present as well. In a normal aorta, numerous intercostal arteries originate from the aorta, although the exact number may vary among individuals. When an aortic dissection initiates, it is inevitable that some intercostal arteries will be affected. This may result in the development of small natural fenestrations within the dissection flap, potentially allowing blood flow from the false lumen, even if such connections are too small to be identified with current CT technology [270]. These natural fenestrations can significantly affect hemodynamics in the false lumen, particularly blood pressure. However, they are often neglected or not taken into account because they are difficult to identify using currently available imaging techniques.

The size and shape of intimal tears can vary among patients, locations, and dissection types. For example, Evangelista et al. [264] reported an average intimal tear size of 10.4±5.2mm for patients with type B dissection and 7.3±4.2mm for those with type A dissection. The size was quantified as the maximum diameter measured by TEE. In their study, the tear size ranged from 3 to 27mm across all patients. Quint et al. [256] similarly conducted a study on the size of 129 intimal tears in 52 patients, providing both the width and length of these tears. The width was defined as the maximum distance between the free edges of the flap, a dimension obtained from CT images. Meanwhile, the length was defined as the thickness of images in which each tear was visible, referring thereby to the axial extent of the tear. Their findings revealed that the tear size varied between 2×1 and 21×45mm, with most of the tears (68%) measuring ≤10mm in each dimension. No significant differences were noted in the size of intimal tears in the ascending aorta, the aortic arch, or the descending aorta. However, the tears in the thoracic aorta were significantly larger than those in the abdominal aorta. Additionally, Cuellar-Calabria et al. [265] conducted a study on the intimal tear size in 72 patients with repaired type A and chronic type B dissections. They found that the proximal tear area was significantly larger in the type B dissection group, with an average size of 1.5cm^2^ (ranging from 0.6 to 2.4cm^2^), compared to the type A group, which had a smaller average tear area of 0.4cm^2^ (ranging from 0.1 to 1.35cm^2^). In contrast, the distal tear area was larger in the type A group, with an average size of 1.0cm^2^ (ranging from 0.55 to 1.5cm^2^), compared to the type B group, which had an average size of 0.4cm^2^ (ranging from 0.2 to 1.1cm^2^). Furthermore, their analysis revealed that a difference greater than 1.2cm^2^ between the proximal and distal intimal tear areas during the subacute phase of aortic dissection was associated with an increased risk of adverse events.

In contrast to the size of intimal tears, their shape has often been examined to a lesser extent. Hirst et al. [141] reviewed 161 dissecting aneurysm cases and characterized the orientation and shape of intimal tears. They found that the tears were most commonly circumferentially oriented, although some were axially oriented. Moreover, they demonstrated that the shape of intimal tears varied significantly, making it difficult to determine a general shape. They identified various shapes, including round, elliptical, and zigzag forms. Similarly, Hukill [271] reported a single case with an intimal tear measuring 35mm in length and oriented at a 45° angle to the circumferential axis. Thubrikar et al. [272] used a mathematical model of an aneurysm to explain the circumferential orientation of the tear. In the dilated aorta, they noted that in certain regions, axial stress exceeded circumferential stress. This finding is consistent with the characteristics of aortic dissection, which is often associated with dilatation, too. Since the rupture strength in the axial direction is generally lower, a rupture caused by axial stress will result in a circumferential tear. Additionally, many of the medical and *in silico* studies discussed in this and subsequent sections typically report the size and shape of intimal tears, as well as the number of tears identified in case reports. However, accurately determining the precise size and shape of intimal tears from medical imaging often proves to be challenging.

#### Extent of the dissection flap.

The path of the false lumen or the dissection flap along the true lumen may vary due to multiple factors. However, the most common locations are on the right side and anteriorly in the ascending aorta, superiorly in the aortic arch, and on the left side and posteriorly in the descending and abdominal aorta. Moreover, the false lumen in the descending thoracic and abdominal aorta often spirals posterior and appears on the right side of the true lumen [258,273]. Although the propagation direction of the false lumen is not yet fully understood, it is clear that anatomical barriers significantly affect its course. These barriers may not be immediately apparent, but the aortic branches, including the numerous intercostal arteries or anchor points such as the ligamentum arteriosum, can considerably impede the progression of the false lumen [267,268].

The dissection flap can propagate in either antegrade or retrograde directions, with a helical shape often observed when it propagates in the descending aorta. Additionally, the flap can naturally extend in the circumferential direction. Weiss et al. [266] conducted a study on type B dissection and compared retrograde dissection propagation based on the intimal tear’s location at the convexity or concavity. They examined 52 patients and found that the retrograde dissection propagation by the left subclavian artery was obstructed when the intimal tear was located at the convexity and served as a natural barrier. In contrast, intimal tears at the concavity allowed retrograde dissection propagation in the aortic arch and ascending aorta in the absence of obstructing vessels at the concavity, typically leading to a higher risk of complications. These findings align with the study of Segesser et al. [262]. However, Roberts [261] described retrograde dissection propagation as a rare occurrence, asserting that most dissections involve the entire aorta and that dissection propagation is only prevented by extensive obstructions such as atherosclerotic plaques or the ligamentum arteriosum. In such cases, the ligamentum arteriosum acts as a natural barrier that prevents anterograde dissection progression, making retrograde type A dissection more likely, which in turn increases the risk of complications [267].

#### Configuration of the dissection flap.

It is known that the dissection flap can exhibit different configurations throughout the cardiac cycle as it demonstrates significant motion, particularly during the acute phase. The configuration of the dissection flap is primarily influenced by the pressure difference between the lumina [4], the remodeled microstructure of the flap, and the circumferential extent of the outer wall of the false lumen. Previous studies have attempted to track the motion of the dissection flap throughout the cardiac cycle, from the acute to chronic phases, and to classify the predominant configurations.

**Fig. 10.**
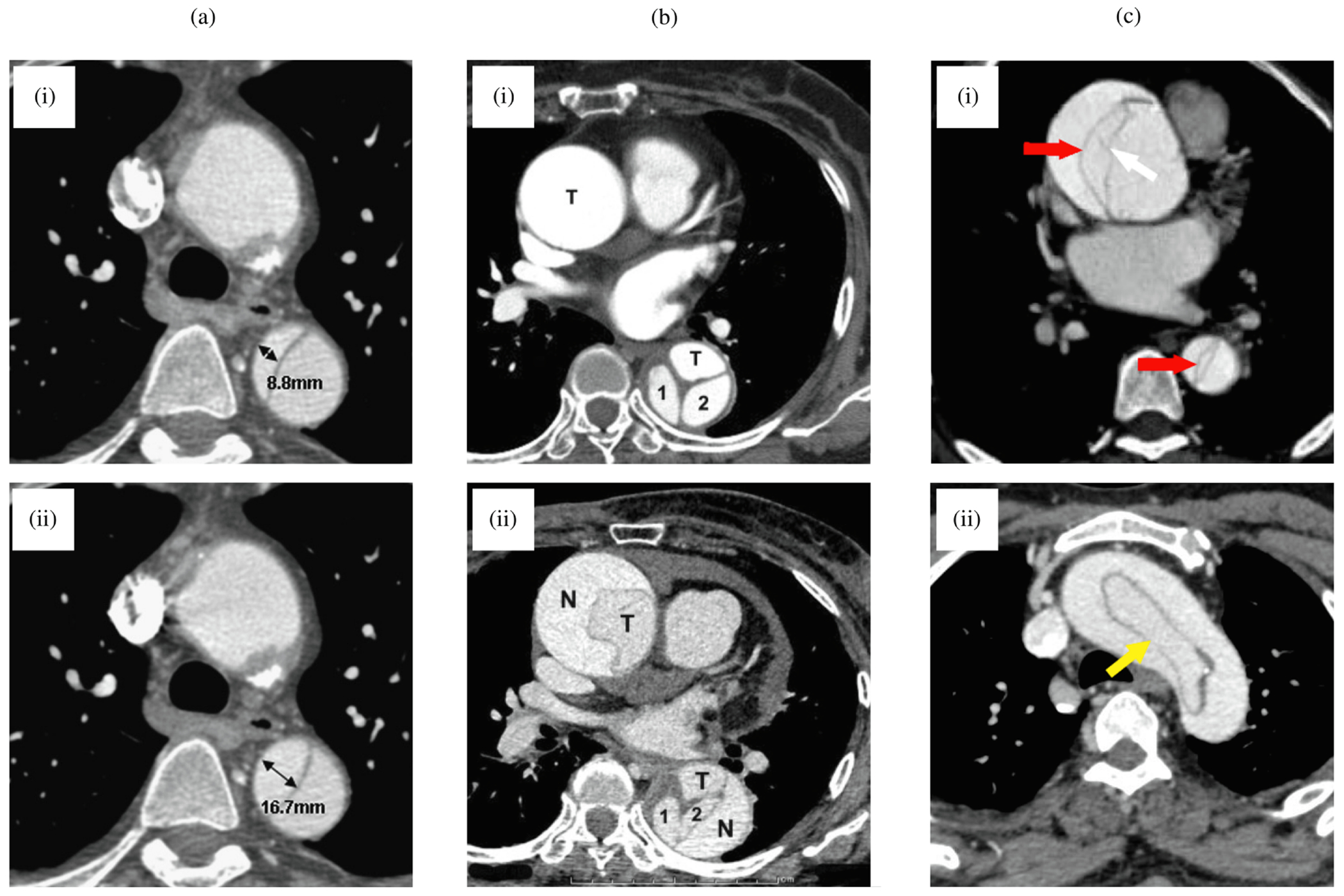
(a) A representative CT image shows a patient with a dissection flap oscillation amplitude of 7.9mm, which is due to different luminal pressures, with (i) a minimal short axis diameter of 8.8mm (ischemic configuration) and (ii) a maximal short axis diameter of 16.7mm (benign configuration) measured at the end of the aortic arch. (b) Another CT image shows (i) a ‘triple-barreled’ dissection in the descending aorta (‘T’: true lumen; ‘1’ and ‘2’: false lumina), which later developed into (ii) a ‘quadruple-barreled’ dissection (‘N’: new false lumen). (c) In addition, a CT image shows (i) the dissection flap (red arrow) in the ascending and descending aorta with linear hypodense structures within the false lumen (white arrow) resembling the dissection flap, known as ‘cobweb sign’, and (ii) a type A dissection with intimointimal intussusception, in which the circumferential dissection flaps with oval morphology (‘windsock sign’) involve the aortic arch (yellow arrow). Figure (a) is adapted from [278] with permission from Elsevier, while (b) and (c) are from Awal et al. [279] (licensed under CC-BY-NC 4.0), and (d) is from Sueyoshi et al. [280] with permission with Elsevier.

In 1997, William et al. [274] proposed a classification system for the dissection flap, distinguishing between ischemic and benign configurations. The ischemic configuration is characterized by a dissection flap curving toward the true lumen, causing the true lumen to take on a C-shaped envelope. This is mainly attributed to a pressure deficit in the true lumen that occurs during diastole, where the term ischemic configuration is derived from the word ‘ischemia’, implying that an insufficient amount of blood flows through the true lumen. Conversely, the benign configuration is more commonly observed during systole when the true lumen pressure is hemodynamically adequate or higher compared with the false lumen pressure. In this configuration, the dissection flap curves toward the false lumen. Lee et al. [275] described these configurations as resembling a gibbous and crescent moon for the true and false lumina, respectively. Both configurations can be observed within a single cardiac cycle, demonstrating the dynamic motion of the dissection flap [276]. The motion of the dissection flap is related to the blood pressure in the true and false lumina and correlates with the phase of the cardiac cycle. Yang et al. [277] found that the measured maximum motion was 12.2±4.1mm, ranging from 2.6 to 17.4mm. Conversely, the minimum motion measured was 6.7±4.1mm, ranging from 0 to 15.3mm. Moreover, the maximum and the minimum dissection flap motion was at 15 and 75% of the R-R interval, respectively. The benign configuration was most frequently observed, followed by the ischemic configuration, with the dissection flap appearing nearly flat in some parts of the R-R interval. LePage et al. [247] examined the flap configuration at three locations along the dissected aorta, comparing acute and chronic dissections, as shown in Fig. 10(a) with exemplary CT images of ischemic and benign configurations. In chronic aortic dissection, the dissection flap appeared flat in all three locations. In acute aortic dissection, there was significant variability among flat, ischemic, and benign configurations at one, two, and three-quarters of the distance along the dissected length of the aorta. This suggests that the configuration also depends on the distance to the intimal tear.

An alternative classification for flap motion in type B dissection was proposed by Weber et al. [152]. This method allowed for a more accurate assessment of the motion of the dissection flap and provided insights into the dynamics of the dissection process. They determined the change in area and diameter of the true lumen at three different locations during the R-R interval of the cardiac cycle, which correlates with the motion of the dissection flap. The relative area changes for the true and false lumina were 16.±5.9 and 10.5±5.7%, respectively. Additionally, the diameter was measured at the major and minor axes of the true lumen throughout the cardiac cycle. The relative diameter changes for the true and false lumina were 5.9±2.0 and 6.1±3.6% at the major axis, and 12.7±6.3 and 6.0±2.2% at the minor axis, respectively. They noted that the area and diameter fluctuated during the R-R interval of the cardiac cycle.

Similarly, Murayama et al. [281] proposed a potential classification system for aortic dissection that distinguishes between pulsating and static types to identify unstable aortic dissection. This classification is based on the area change of the true lumen and aims to predict future events such as dilatation, occurrence of additional fenestrations, and death. They measured the true lumen area at three different locations in the descending aorta and established a threshold value to distinguish between pulsating and static types. According to their classification, the pulsatile type is present when the maximum area of the true lumen in any of the three locations exceeds 125% of the minimum area of the true lumen, and when the intimal tear is not situated at the distal portion of the descending branch with retrograde flow. The results indicated that the pulsatile type occured 4.3±5.9 months after onset, while the static type occured 42.0±13.0 months after onset.

Ganten et al. [278] investigated pulsatile changes in the aorta, which contributed to the highly variable motion of the dissection flap. They found that the true lumen diameter decreased by up to 29% (mean decrease of 4.4%) during certain phases of the cardiac cycle, which is directly linked to flap motion. The area of the true lumen, associated with its diameter, changes not only throughout the cardiac cycle but also with the progression of aortic dissection. After the initial dissection event, the true lumen area undergoes significant alterations but eventually stabilizes as the aortic dissection progresses until the area remains constant.

Lescan et al. [253] studied the impact of pre-operative dissection flap motion on aortic remodeling and development of dSINEs in patients undergoing TEVAR. They assessed pre-operative dissection flap mobility by measuring the true lumen strain from diastole to systole. They found that the true lumen strain was superior in the acute and subacute groups compared with the chronic group. Moreover, a mobile flap with a mean true lumen strain greater than 22.5% was associated with a higher true lumen expansion rate in the thoracic aorta and a comparable distal stent-induced new entry incidence compared with the immobile flap. These findings suggest that pre-operative assessment of dissection flap mobility can be useful in predicting aortic remodeling and post-operative outcomes in patients undergoing TEVAR.

Similarly, Lortz et al. [252] examined the relationship between the mobility of the dissection flap and aortic remodeling in patients with chronic type B dissection after a TEVAR or a frozen elephant trunk procedure in 52 patients with a mean follow-up of 26.6±20.7 months. They classified non-mobility as ≤3mm and considered a real-time dissection flap movement of >3mm with a high amplitude as mobile and a low amplitude as non-mobile. They found that patients with a highly mobile dissection flap prior to operation had improved aortic remodeling and a lower re-intervention rate compared with those with impaired dissection flap mobility. Intravascular ultrasound assessment showed that the mobility of the dissection flap led to a greater true luminal loss and shift in the mobile flap group during every cardiac cycle. These findings suggest that intravascular ultrasound assessment of dissection flap mobility can be useful in predicting post-operative outcomes in patients after TEVAR or frozen elephant trunk.

Numerous studies have also reported the collapse of the true lumen. For example, Williams et al. [282] attributed collapse to the loss of transmural pressure across the dissection flap, indicating a decrease in radial tension but not necessarily axial tension. As a result, the elastic fibers recoil and shorten the flap due to the release of transmural pressure. Furthermore, they suggested that an increased circumference of the outer wall of the false lumen could be an indicator of potential collapse of the true lumen.

In conclusion, the motion of the dissection flap typically diminishes during the transition from the acute to the chronic phase of aortic dissection. This reduction is primarily associated with microstructural remodeling of the flap, see Section 3.2. The stiffness of the flap also serves as a decision criterion for surgical intervention. While increasing stability can enhance the safety of TEVAR, it can conversely decrease its likelihood of success [283]. In other words, immediate intervention could offer benefits because the dissection flap is in its most pliable state and provides the greatest possibility for complete remodeling [284]. However, this advantage needs to be balanced against the potential increased risk of injury to the acutely vulnerable and fragile inflamed aorta with the stent-graft, which could make the patient more susceptible to complications [285].

#### Rare manifestations of the dissection flap.

Typically, the true and false lumina are separated by a single dissection flap, as previously discussed. In some cases, however, the false lumen is ‘multi-barreled’, exhibiting multiple false lumina at the initial presentation or during the follow-up period, leading to a ‘triple-barreled’ or ‘quadruple-barreled’ aortic dissection [131,132,271,279,286], see Fig. 10(b). This manifestation of the dissection flap is not widely recognized and has been considered a relatively rare condition; in approximately 9% of acute aortic dissection patients, a multi-barreled false lumen is observed [130]. These cases are typically only documented in individual case reports. Patients with multi-barreled aortic dissections show significantly poorer survival rates than patients with double-barreled aortic dissection.

Other relatively rare observations in clinics include the ‘cobweb’ and the ‘windsock’ sign. The cobweb sign, as introduced by Williams et al. [287], appears as slender linear areas of low attenuation that are specific to the false lumen and are caused by residual ribbons of media that have incompletely sheared off during the dissection process. Example medical images can be found in Fig. 10(c) or in the literature [248,288]. The term ‘windsock sign’ in type A dissection was introduced by Karabulut et al. [289] to describe an intraluminal windsock-like appearance filled with contrast material and surrounded by a contrast column, a curvilinear intimal flap in the aortic root, the absence of a flap in the dilated mid-ascending aorta, and a dissection with intimointimal intussusception, see also [279,290–293]. This involves a circumferential dissection of the aortic lumen, followed by impediment of the origin of the supra-aortic vessels and occlusion of the aortic lumen, as shown in Fig. 10(c).

#### 3.1.2. Dilatation and growth of the aorta

**Fig. 11.**
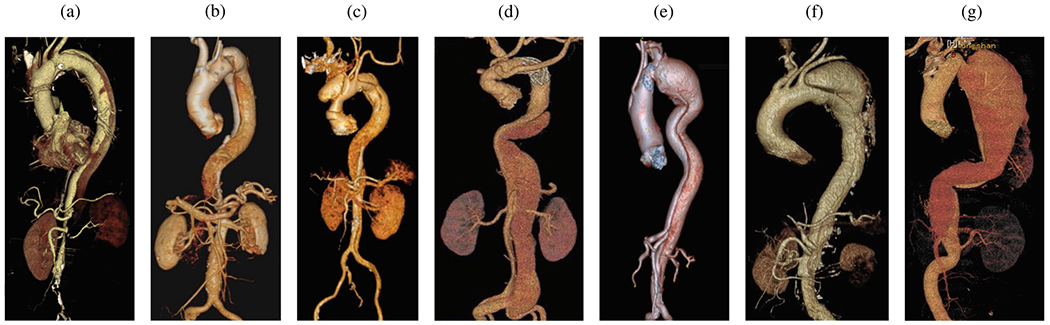
(a) to (g) CTA images demonstrate various remodeled anatomies of aortic dissection patients and showcase dilatation and growth of the aorta, including altered elongation, tortuosity, and angulation. Figure (a) is adapted from Chan et al. [294] with permission from Elsevier, while (b) and (c) are from Lopes et al. [295] (licensed under CC-BY-NC-ND 4.0), (d) is from Dai et al. [296] (licensed under CC-BY-NC 4.0), (e) is from Sultan et al. [297] (licensed under CC-BY 4.0), (f) is from Anwar and Hamady [298] (licensed under CC-BY 4.0), and (g) is from Ma et al. [299] with permission from Elsevier.

In addition to the development of a dissection flap and possible intimal tears, the aortic anatomy undergoes remodeling over time. Some patients experience aortic dilatation, while others may exhibit elongation or axial growth of the aorta, increased tortuosity, pronounced angulation in the aortic arch, or a combination thereof. Some of these remodeling processes can be associated with age-related growth, making them potential risk factors for aortic dissection. In other words, certain types of remodeling might occur pre-dissection, making the aorta more prone to developing a dissection, while others are caused by the dissection itself. As an illustration, Fig. 11 presents a comparison of various morphological changes resulting from significant aortic remodeling, such as dilatation, increased tortuosities, and altered angulations, in aortic dissection patients, as captured by CT imaging. To gain a clearer understanding of aortic dilatation in dissection, it is beneficial to first revisit Laplace’s law.

#### Laplace’s law of wall tension.

Laplace’s law is a fundamental relation in physics that explains the relationship between the tension in an elastic idealized thin tube and the pressure exerted by the wall on the fluid inside [300]. The circumferential tension in the wall of a cylindrical tube is expressed as *T* = *Pa*, where *P* is the internal pressure and *a* is the inner radius of the tube, with *a*<*r*<*b*, where *b* is the outer radius. The circumferential stress within the tube wall is given by *σ* = *Pa*/*h*, where *h* is the thickness of the wall.

This law implies that the inward pressure exerted by the blood on the arterial wall is directly proportional to the tension in the wall and inversely proportional to the radius of the artery. Consequently, larger arteries must have thicker walls to withstand higher tension, while smaller arteries can afford thinner walls. In other words, at the same pressure and wall morphology, arteries with larger diameters experience greater wall tension than those with smaller diameters. Therefore, arteries that carry higher blood pressure have thicker walls compared to veins. When an artery wall develops a weak spot and dilates, the dilatation subjects the weakened area to even more tension, which can lead to a aneurysm (i.e. a dilatation of 50% or more) or a rupture. Although rupture is rare when the diameter is less than 55 mm. A possible protective mechanism against additional dilatation is wall thickening, which will decrease wall stress and be explained in more detail in Section 3.2.2. Wall stress alone does not determine the likelihood of aneurysm rupture. Material failure occurs when the mechanical stress exceeds the strength of the material. The diameter of the aortic aneurysm alone does not fully account for either wall strength or stress [302,303].

In conclusion, although Laplace’s law helps to understand the fundamental principles of arterial wall tension and pressure, predicting the risk of aortic dilatation and its rupture requires a more sophisticated approach that considers the complex geometry of the aortic wall and the material properties of the vessel. In fact, aortic dilatation is the most commonly observed factor to assess long-term survival of patients with aortic dissection.

#### Dilatation.

In type B dissection, aortic dilatation and the rate at which the aorta dilates are crucial factors in assessing long-term survival of patients. False lumen dilatation, as shown in Fig. 11 and in Fleischmann et al. [34], is the primary reason for reintervention in patients with chronic aortic dissection [34,304]. Furthermore, many patients often experience false lumen dilatation following dissection onset, typically observed in the upper descending aorta [2]. According to the IRAD and others, only 65.5 and 26.7% of type B dissection patients are free of false luminal dilatation three and five years after onset, respectively [305,306]. In contrast, type A dissection rarely progresses into the chronic phase due to the high mortality rate of patients. In surveillance imaging, dilatation and annual growth rate are typically determined based on the difference between the initial and most recent maximum diameter measurements. These measurements are most commonly obtained in clinical settings for the total aortic diameter.

**Fig. 12.**
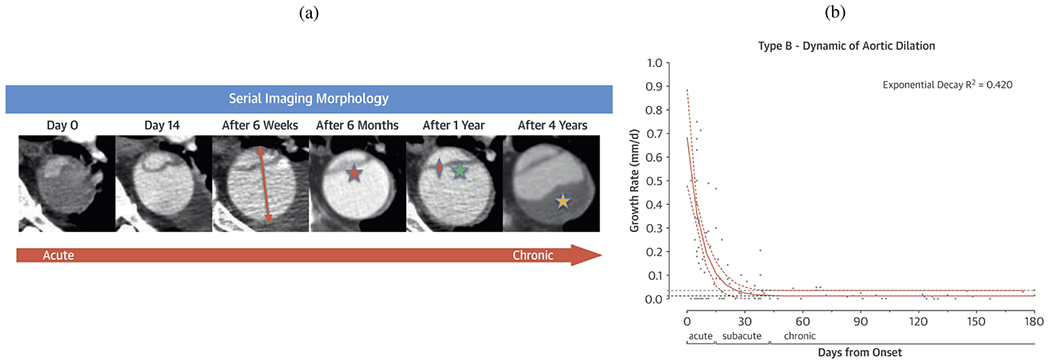
(a) Evolving morphology of type B dissection based on follow-up CT imaging in a single representative patient at the same aortic levels, showing notable changes, particularly in the degeneration and dilatation of the false lumen, with important observations including a significant initial increase in aortic diameter (orange arrow), progressive intimal thickening (orange star), decreased movement of the aortic flap over time (orange triangles), gradual straightening of the flap (green star), and increasing false lumen thrombosis lumen over time (yellow star). (b) Growth rate (mm/day) of aortic dilatation following an exponential decay function with rapid growth in the acute phase, deceleration in the subacute phase, and a plateau in the chronic phase. Figure is adapted from Peterss et al. [14] with permission from Elsevier.

When calculating the annual growth rate, it is typically assumed that the aorta grows uniformly and linearly over time, an assumption that can be questioned. Peterss et al. [14] reported that a rapid change in diameter occured early in the post-dissection period, stabilizing after 25 days and reaching a plateau after 88 days, see Fig. 12(b). From this point onward, the increase in dilatation occured much more slowly and there were no longer any further variations over time. The dilatation rate was estimated to have a mean of 9.31mm/year in the acute phase, 1.30mm/year in the subacute phase, and 0.32mm/year in the chronic phase.

Several important predictors that may influence dilatation and growth rate were summarized and identified by Spinelli et al. [15]. These included the total cross-sectional size of the aorta, the cross-sectional size of the false lumen, the location and size of the proximal intimal tear, the number of intimal tears, the status of the false lumen with respect to thrombosis, axial extension of the false lumen, and branch vessel involvement. This underlines the highly correlated remodeling phenomenon. However, the various studies presented did not always report consistent findings.

In order to measure the maximum diameter of the total aorta, i.e. the cross-sectional size, various methods have been used in multiple studies. For example, it was measured as the largest diameter perpendicular to the center-lumen-line or as the maximum short-axis distance in axial view [307–309]. Others developed a vascular deformation mapping technique to provide a reliable quantitative assessment of 3D aortic growth and growth patterns in patients with thoracic aortic aneurysms and dissections undergoing CT surveillance [310,311]. This technique is one of several methods used to describe the geometric evolution of the aorta; see also the recent work of Khabaz et al. [312]. Despite the diverse imaging techniques employed, a good agreement was found among these studies concerning its prognostic value for adverse events and mortality. In the majority of the examined studies, a large aortic maximum diameter in the acute phase was a positive predictor for late aortic growth or negative outcomes. Intriguingly, certain studies identified a link between initially smaller aortic diameter and unfavorable consequences [313–316], whereas other investigations failed to establish a significant correlation. Additional studies established a threshold of 40 to 41mm, measured in the acute phase, indicating an increased risk of late adverse events. This benchmark was first introduced by Kato et al. [317] in 1995, who observed no late aortic growth below this limit. Later, in 2011, Miyahara et al. [318] confirmed the threshold value of 40.5mm, although this finding might only be applicable to patients with a height of less than 180cm [319]. Most recently, Evangelista et al. [320] showed that an initial aortic diameter of >45mm in the acute phase identified patients with increased risk for complications.

To assess the false lumen cross-sectional size, various methods have been employed, either as absolute values or relative to the true lumen. For example, the cross-sectional area of the false lumen was evaluated by calculating the ratio of the true lumen to the entire lumen at the maximum diameter level [317] or by determining the maximum area of the false lumen [321]. In 2007, Song et al. [307] discovered that an initial absolute false lumen diameter at the upper descending aorta ≥22mm was predictive of late aneurysmal change, which was defined as a total aortic diameter greater than 60mm. The ratio of false lumen to total aortic diameter increased over time in patients experiencing aneurysmal changes. Other researchers reported that the circular configuration of the true lumen was significantly associated with a reduced growth rate [322], or that the circumferential extent of the false lumen, which is the angular distance in degrees between the two insertion points of the dissection flap, predicted adverse events [270]. In addition, others found that false lumen volume and the ratio between true and false lumen volume were indicators of delayed aortic intervention [323]. In summary, numerous findings were presented, but the employed measurement techniques vary and statistical significance could not be shown for all outcomes [264,309,324,325]. Also note that information about the presence of false luminal thrombus and its impact on the measurements obtained was usually not provided.

The presence of an entry tear in the thoracic aorta was also identified as a significant predictor of aortic growth by Kato et al. [317]. However, in their study, it was reported merely as the presence or absence of an entry tear in the thoracic aorta. In two studies, Sueyoshi et al. [326,327] examined the presence of an entry tear in the aortic arch as a predictor of aortic growth in two separate studies, finding it insignificant in 2004 and significant in 2009. Similar to others, some studies investigated the role of the distal exit tear or its location on the aortic arch’s concavity and its distance from the left subclavian artery [266,328]. The latter was associated with a more complex clinical course. In summary, there is no consensus. It has been hypothesized that a single proximal entry tear is linked to increased false lumen pressurization and a higher risk of growth and adverse events [264]. Results are however inconsistent [315,329–331]. The number of intimal tears was found to be protective against aortic growth in two studies [309,332]. In addition, Sailer et al. [270] discovered that the number of intercostal arteries was also protective against adverse events, possibly due to natural fenestrations. Earlier studies, on the other hand, reported conflicting findings, with the number of branch vessels as predictor for higher mortality and complications [321,333–335].

**Fig. 13.**
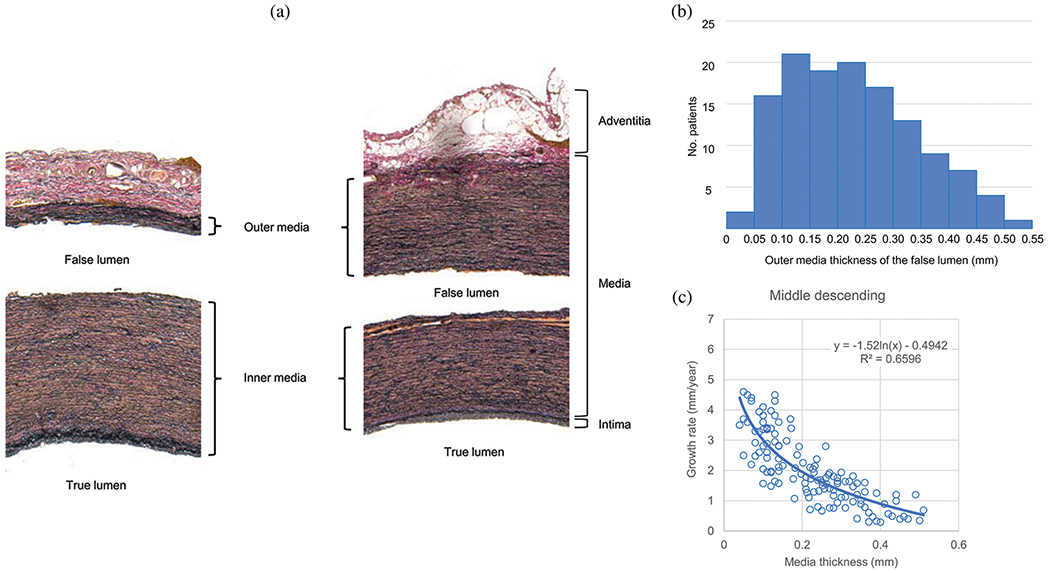
(a) Representative examples of a thin and a thick outer media (original magnification 40x, Elastica van Gieson stain). (b) A histogram of measured outer media thickness showing a right-skewed distribution normalized by square-root transformation. (c) Scatter plots illustrating the inverse relationship between outer media thickness and total annular growth rate in the middle descending aorta. Figure is adapted from Kinoshita et al. [336] with permission from Elsevier.

An alternative methodology for evaluating aortic dilatation was introduced by Kinoshita et al. [336]. They measured the outer media thickness of the false lumen using microscopic images of specimens taken intraoperatively (see Fig. 13) and assessed its influence on secondary dilatation of the downstream aorta, similar to the investigation on intramural hematoma in 2005 [337]. Of the 238 investigated patients, 129 met the inclusion criteria of this study: DeBakey type I dissection with a patent false lumen, histopathological examination of the full-thickness aortic wall, and at least one follow-up CT scan more than three months after the surgical procedure. The aortic wall specimens were stained with hematoxylin and eosin and Elastica van Gieson stains, and the thickness was measured by two physicians at eight equally distributed locations, from which average values were calculated. Overall, they noted two important findings. First, there were considerable variations in the thickness of the outer media ranging from 0.04 to 0.51mm, with the distribution shown in Fig. 13(b), in aortic wall specimens obtained during surgery for acute aortic dissection. Second, the growth rate of the distal remaining dissecting aorta was inversely correlated with the thickness of the outer media (see Fig. 13(c)). In particular, patients in the lowest tertile of outer media thickness (0.04 to 0.15mm) exhibited remarkable dilatation within one year in the early postoperative period, with about 5 to 8mm/year growth observed in the first year, while the change after that was only 1 to 2mm/year. It was also found that the risk of aortic-related events is significantly higher in patients with an outer media thickness of 0.04 to 0.15mm. They later compared the location of the false lumen within the medial layer between an acute intramural hematoma and an acute aortic dissection and showed that the acute intramural hematoma group had significantly thinner outer media thickness than the acute aortic dissection group [336].

Recently, Houben et al. [338] have expanded the analysis of aortic conditions by going beyond measuring maximum aortic diameter. They used available CTA scan images to correlate 3D aortic growth and cyclic transmural wall stress with the location of intimal tear formation. This study retrospectively identified three patients with type B dissection and connective tissue disorder, each with two available ECG-gated pre-dissection CTA scans and no surgical repair during the pre-dissection interval. 3D aortic growth between two pre-dissection clinical CTA studies was quantified using vascular deformation mapping, a method previously introduced by [310,311]. Vascular deformation mapping involves three primary steps: segmentation, image registration, and deformation quantification. This technique may be more suitable for investigating the relationship between aortic dimensions and the risk of aortic dissection because it is less prone to measurement variability and can represent the 3D nature of growth. While it works well for small changes in aortic dimension, the predictability for large changes is not yet reliable, which is why short time intervals between follow-up examinations are required. In addition, finite element models of the structural domain were developed to estimate the cyclic transmural wall stress in patient-specific baseline CTA geometries (Section 8.1). The findings showed that focal areas of growth and transmural wall stress co-localized with the site of intimal tear formation. This supports the hypothesis that the risk of dissection is highest at sites where inadequate wall integrity (e.g., growth) and abnormal wall stress intersect. These hypothesis-generating results suggest a new analytic avenue for a more sophisticated assessment of the factors leading to initiation of dissection in patients with connective tissue disease. In particular, the application of vascular deformation mapping emerges as a promising approach that can not only enhance existing risk-stratification techniques but also deepen our understanding of aortic dilatation throughout disease progression, as recently demonstrated in Marway et al. [339].

**Fig. 14.**
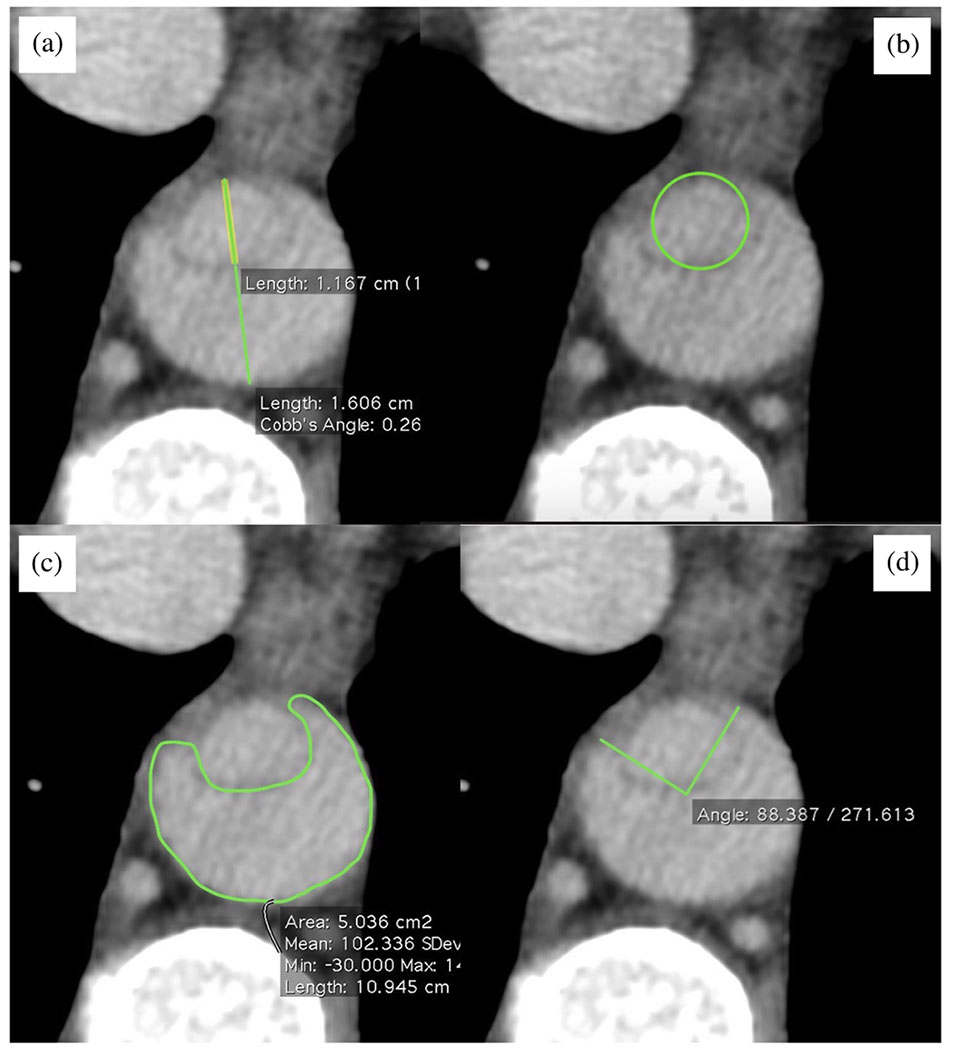
Illustration highlighting the difficulties in measuring the size of the false lumen for predicting patient risk, and the resulting variations in assessing potential risks. The false lumen size is considered as a predictor of lower risk according to Song et al. [307] ((a); initial false lumen diameter of <22mm), Evangelista et al. [264] ((a); true lumen diameter is greater than one-fourth of the total diameter), Tolenaar et al. [332] ((b); true lumen circular shaped), and Chang et al. [321] ((c); initial false lumen area of <922cm^2^). Conversely, it is an indicator of higher risk according to Sailer et al. [270] ((d); angular extent of initial false lumen of >249%). Figure is reprinted from Spinelli et al. [15] with permission from Elsevier.

In summary, the initial aortic size in the acute phase – typically measured either as the total aortic diameter or the false lumen diameter – is the most extensively used morphological parameter to monitor false lumen dilatation. Interestingly, a large initial false lumen diameter consistently predicts the risk of adverse events and mortality in both early and late stages. Notably, multiple studies support a 40mm threshold value, with initial aortic diameters above this value independently predicting adverse events. While less studied than the initial aortic diameter, the initial false lumen size also appears to be a significant prognostic factor, with reasonably consistent results across studies. Various methods have been employed to measure the initial false lumen size, yielding partly contradictory results, as seen in Fig. 14. Established threshold values for the initial false lumen include a diameter of 22mm and an area of 922mm^2^. Due to their predictive capabilities, diameter plots have already been partially integrated into clinical practice [340,341]. They are used for long-term surveillance of aortic dissection patients to visually compare complex features and assess the associated risk of aortic dissection, as exemplified by Bäumler et al. [342], particularly due to their simplicity. In addition to the aortic diameter, they can be augmented to show features such as blood supply to the aortic branches or previous treatments. Moreover, compression of the true lumen by the false lumen indicates false lumen pressurization, which is thought to promote aortic growth and complications. However, the significance of other factors remains controversial, especially because measuring these factors consistently is challenging. In the following sections, the impact of the flow pattern and thrombus formation in the false lumen on the onset of dilatation post-dissection onset will be explored. It is important to note that both depend heavily on the number, size, and location of intimal tears.

#### Elongation, tortuosity, and angulation.

In contrast to previously discussed aortic dilatation, the roles of elongation, tortuosity, and angulation in aortic dissection remain largely uncertain. However, these factors have the potential to be risk factors. It is still unclear to which extent geometric remodeling and growth prior to dissection should be considered risk factors, especially when compared with the remodeling that occurs post-dissection. This type of geometric remodeling is frequently observed in clinical settings but has not been studied adequately, partly due to challenges in measurement. It is widely believed that the aorta, like other large elastic arteries, grows in the axial direction, meaning that it elongates, developing increased tortuosity and a pronounced angulation with age. Studies have reported an axial growth of 0.07 to 0.09mm/year [168,343–345]. Aortic elongation, similar to dilatation, may also contribute to increased tortuosity and changed angulation, likely resulting from a sustained loss of elastic fibers and, consequently, a decrease in arterial wall elasticity. This phenomenon may be attributed to the aortic tissue experiencing ‘material fatigue’ with advancing age, leading to fragmentation and disorganization of elastin fibers and elastic lamellae [343] or adverse mechanobiological responses [346]. Hence, many of the subsequently presented studies discuss geometrical remodeling with respect to aging effects. This is also because the role of elongation, tortuosity, and angulation in aortic dissection has still gained little attention as a risk factor for dissection onset or mortality.

Due to the non-intuitive nature of the measurements, a variety of techniques exist for studying these anatomical features [347]. Therefore, it can be difficult to compare absolute values from different studies. Aortic length is often defined as the centerline distance between two corresponding planes. The axial length of the true and false lumina in the descending aorta are determined by establishing their respective centerlines. In contrast, aortic tortuosity can be calculated by dividing the centerline length by the linear distance between the first and last points in each aortic segment. Finally, aortic angulation can be defined as the angle formed by tangent lines drawn along the first and last points of the aortic centerline in each segment.

In the 1950s, Dotter et al. [348] conducted one of the first studies on the length of the thoracic aorta, as there had been no previous reports regarding this measurement. The study aimed to investigate the relationship between aortic length and factors such as age, arteriosclerosis, syphilis, and hypertension. The authors concluded that the normal length of the thoracic aorta spans a relatively wide range, and despite the influence of age, no significant correlations were observed. Sugawara et al. [343] conducted a study on aging with 256 healthy adults aged 19 to 79 years. They discovered that the length of the ascending aorta increased with age, while the length of the descending aorta exhibited no correlation with age. Moreover, elongation of the ascending aorta was associated with increased pulse wave velocity in the aorta, suggesting that elastic properties and local pulsatile pressure might play a causal role. These findings were also observed in adults without apparent cardiovascular disease. Morrison et al. [239] also investigated age-related effects by conducting a study on 14 patients aged 35 to 80 years who had no visible aortic pathology. They measured the cyclic strain of the arch and descending thoracic aorta. Cyclic axial strain was found to decrease by 50% with increasing age, from 2.0±0.4% in the younger group to 1.0±1% in the older group. However, arch length increased by 14% from the younger to the older group. Therefore, the thoracic aorta elongates with increasing age and has a significantly lower axial strain.

A series of studies by Kruger and his colleagues examined various aspects of aortic length in patients with aortic dissection, pre-dissection, and healthy controls. In the first study [349], they identified a positive correlation between age and aortic length in healthy controls. Dissected aortas and aortas prior to dissection were significantly elongated compared with healthy counterparts, suggesting that aortic length parameters are related to morphological changes before or during aortic dissection. The midline length of the healthy ascending aortas was 71mm, while the same values for thoracic aortas pre- and post-dissection were 81 and 92mm, respectively. The second study [350] revealed that the ascending aorta and the aortic arch were significantly longer in both thoracic aortas pre- and post-dissection compared with control aortas. Aortic morphology correlated with age but not with parameters of body size. Similarly, in the control aortas, the central line distance from the aortic valve to the brachiocephalic trunk was 93mm. In thoracic aortas pre-dissection, it was 111mm, and in thoracic aortas post-dissection, it was 117mm. The third study [351] analyzed aortic lengths in healthy, ectasia, aneurysm, pre-dissection, and post-dissection groups. It demonstrated that the ascending aorta was significantly longer in all groups compared with the control group. However, the correlation between diameter and length of the ascending aorta indicated that both parameters must be examined separately. Finally, in Lescan et al. [352], they retrospectively compared a healthy control group and patients with type B dissection. The segment with the most considerable difference in elongation between the study groups was the non-dissected aortic arch of patients with type B dissection. Together, these studies highlight the importance of considering aortic length as a relevant parameter in the context of thoracic aortic dissection and related conditions.

Similar findings were reported in two subsequent studies, which first investigated the role of aging with respect to aortic elongation and then compared healthy control patients with aortic dissection patients. Adriaans et al. [353] conducted a cross-sectional case study with 210 consecutive patients. They discovered that the length of the thoracic aorta was significantly related to age, increasing by 59 and 66mm in males and females, respectively, over the age range from 20 to 80. The lengthening was most pronounced in the proximal descending aorta, which displayed an almost 2.5-fold length increase during a person’s lifetime. The lengthening of the thoracic aorta was accompanied by marked changes in geometry, with the aortic apex shifting to a more distal position in elderly patients. Later, in a study by Heuts et al. [354], 250 patients were included to investigate the relationship between acute type A dissection and aortic dimensions. The ascending aorta was found to be longer in acute type A dissection patients compared with healthy controls. However, no differences were observed in the lengths of the aortic arch and descending aorta. Moreover, the results revealed that the ascending aortic length and diameter were independent predictors for acute type A dissection.

Analogously, Wu et al. [355] discovered that an ascending aortic length of ≥13cm was associated with an almost fivefold higher average yearly rate of aortic adverse events compared with an ascending aortic length of <9cm. They suggested an aortic elongation of 11cm as a potential intervention criterion for acute type A dissection. The mean estimated annual aortic elongation rate was 0.18cm/year, and aortic elongation was found to be age-dependent. Eliathamby et al. [356] demonstrated that the ascending aorta was longer in patients immediately following acute type A dissection, in the pre-dissection group, and in the aneurysm group compared with controls. Furthermore, Sun et al. [347] found that the length of the ascending aorta and the total aorta was significantly longer in the acute type B dissection group, while in the acute type B dissection group the length of the total descending aortic lumen was significantly longer than in the control group. In a follow-up study, Sun et al. [357] then divided 112 patients into three groups: with normal aortic diameter, with ascending aortic aneurysm, and with type A dissection. The ascending aortic length was correlated with age. In their study, the length of the ascending aorta in the adjusted dissection group was 14.5mm longer than that in the aneurysm group and 25.3mm longer than that in the control group. Interestingly, in the dissection group, there was no correlation between the length of the aorta and age, and the median age was younger than that in the aneurysm group. Cao et al. [358] showed that the ascending aorta and the aortic arch lengths in acute type B dissection increased significantly compared with controls. Shirali et al. [359] compared hypertensive patients with and without aortic dissection and found that the length of the proximal and entire aorta was greater in the aortic dissection group.

Aortic tortuosity and angulation have been the focus of numerous studies, investigating their significance in the context of aortic dissection in various patient populations. Gode et al. [360] found that among patients undergoing ascending aortic surgery due to aneurysm or aortic dissection, ascending aortic angulation was significantly less in aortic dissection patients compared with aneurysm patients. In a separate study of patients with Marfan syndrome, Franken et al. [361] observed that increased aortic tortuosity was an independent predictor of clinical events, including aortic dissection. Focusing on patients with acute type B dissection, Cao et al. [358] found that aortic arch tortuosity was increased and angulation was more pronounced in this group compared with controls. Aortic arch tortuosity was also identified as a significant factor in bicuspid aortic valve patients by Alhafez et al. [362], as it may help identify those at increased risk for aortic dissection. Other studies have demonstrated correlations between aortic tortuosity and various patient groups. Jie et al. [363] found a moderate correlation with ascending aortic tortuosity in acute type A dissection patients, while Shirali et al. [359] noted increased aortic tortuosity in hypertensive patients with acute type B dissection compared with normotensive and hypertensive patients without aortic dissection. Rylski et al. [364] discovered that acute aortic dissection led to increased tortuosity in the originally non-dissected ascending aorta, with this increase observed only in the ascending aorta and not the proximal descending aorta or arch. Finally, Sun et al. [347] compared acute type B dissection patients with a control group and found smaller angulations in the ascending aorta and aortic arch, along with significantly increased tortuosities in the aortic arch and total aorta. Although the mechanisms of tortuosity remain to be determined, complex mechanical and mechanobiological factors likely contribute [346]. Regardless, these studies underscore the relevance of examining aortic tortuosity and angulation as relevant factors in the context of aortic dissection and related conditions [365].

In addition to diameter, other factors such as an elongated aorta, increased tortuosity, and a smaller angulation angle may significantly contribute to the pathophysiology of aortic dissection. These factors could potentially be age-related risk factors for aortic dissection, as demonstrated by numerous studies, particularly in the ascending aorta and the aortic arch, which have been observed to elongate. As discussed by O’Rourke et al. [366], it is noteworthy that the aortic arch is anchored to surrounding structures by three branches, while the proximal descending aorta is tethered to the spine by intercostal arteries. In contrast, the proximal aorta connects to the heart, which moves within the pericardial sac during the cardiac cycle. As the ventricle contracts, the aortic valve annulus moves downward, leading to axial movement of the most proximal aorta. This suggests that not all parts of the aorta grow similarly. Due to the tethered descending aorta, the aorta might be more prone to developing a lower angulation angle and increased tortuosity to balance elongation. In fact, ascending aortic tears are typically transverse rather than axial, suggesting greater disruptive strain in the axial direction than in the radial direction. Additionally, as reiterated by numerous studies, changes in aortic dimensions might be associated with increased aortic pulse wave velocity. Current guidelines recommend treatment based on hinge points in aortic diameter as the sole criterion, which might be insufficient [367,368]. Recent results also suggest that volumetric distensibility, which is correlated with pulse wave velocity, can serve as a new metric in the risk-stratification process, as it strongly correlates with aneurysm failure risk [63]. However, the development of a comprehensive global risk score with threshold values is unlikely to encompass all aspects of this complex disease. Moreover, there is no consensus on how to accurately measure these quantities, particularly tortuosity and angulation, making it difficult to compare absolute values between studies.

### 3.2. Pathophysiology of mural remodeling

#### 3.2.1. Degenerative aortic histopathology

#### Idiopathic cystic medial necrosis according to Erdheim-Gsell.

In 1929, Jakob Erdheim reported an unusual disease in his analysis of a patient’s case [369]. This specific case involved a patient with a dissecting aneurysm without any noticeable atherosclerosis. Due to the non-atherosclerotic nature of the intima, it was delicate, smooth, and translucent, allowing detailed study of the aortic media. Intriguingly, Erdheim initially misinterpreted the described symptoms as signs of syphilis.

**Fig. 15.**
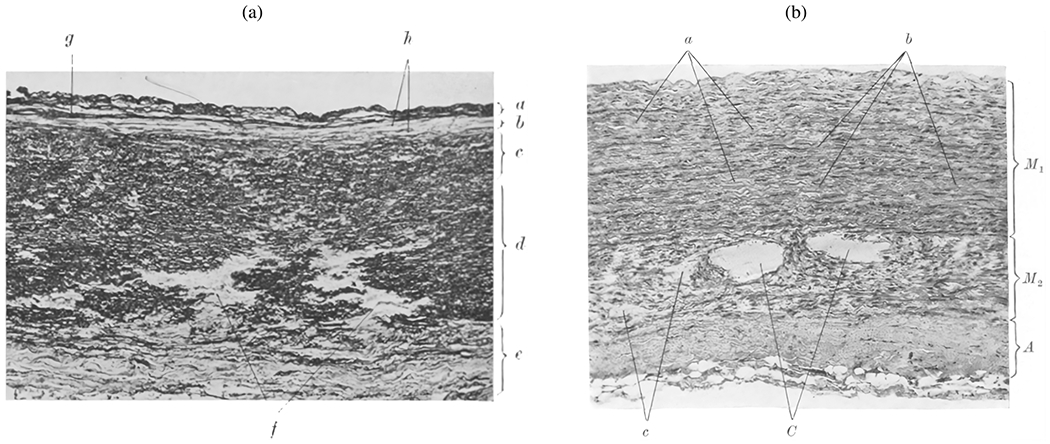
Two histological images showcase the main findings of Erdheim, denoted as cystic medial necrosis. (a) A histological image of medial necrosis features the inner elastic intimal layer (‘a’), the markedly thin outer muscular intimal layer (‘b’), and the inner and outer medial layers, (‘c’) and (‘d’). Notably, the inner medial layer (‘c’) is abundant with cell nuclei, while (‘d’), the outer medial layer, is necrotic, extending close to the adventitial layer (‘f’) and is replaced by degenerated tissue devoid of cell nuclei. Finally, (‘e’) represents the adventitia. (b) The distribution and condition of tissue in the medial layers, where (‘M1’) signifies the inner two-thirds of the original media, marked by several focal areas of cell apoptosis, (‘a’) and (‘b’). (‘M2’) represents the outer third of the media, exhibiting regenerated tissue with mucoid accumulation (‘c’) and small cysts (‘C’). The adventitia, labeled (‘A’), is seen thickened and poor in nuclei. Figure (a) is adapted from Erdheim [369], while (b) is from Erdheim [370], with permission from Springer Nature, respectively.

Erdheim, an Austrian pathologist, documented two primary histological findings predominantly found in the outer media. First, he noted focal areas of necrosis. Second, he identified a degradation of the medial tissue, located primarily in the outer half of the media, while the remainder of the wall remained relatively normal. He reported that the latter observation was related to degenerative changes characterized by sharply defined patches of different appearance. These patches often appeared in a linear pattern. Moreover, the typically clearly visible elastic lamellae were either completely missing or barely visible, as demonstrated in Fig. 15(a). The loss of collagenous tissue occurred gradually, possibly resulting in the absence all three main components of the media. Interestingly, in certain cases the SMCs persisted until the final stages of the disease. It has also been observed that the fluid contents of these cavities seep into the surrounding tissue crevices. The second observation, necrosis, resulted in a conspicuous absence of nuclei. In areas of advanced changes, necrosis played a significant role, resulting in widespread cell apoptosis and tissue degradation. Beyond gross changes, necrosis gave the impression of a uniform reduction in the number of cell nuclei, suggesting a slow, progressive process of SMC loss that eventually led to a complete absence of nuclei.

The relationship between necrosis and tissue degradation seemed complex, and was not entirely clear to Erdheim. Nevertheless, he proposed two possible scenarios. In the first case, necrosis could precede and lead to subsequent tissue degradation. This hypothesis was supported by the fact that the process of necrosis could be traced microscopically. However, there were many cases without visible signs of necrosis. In several cases, tissue degradation without preceding necrosis was observed. The localized loss of non-necrotic media supports the concept that in a given area, elastic fibers and collagenous tissues often disappeared first, leading to cavity formation, while SMCs remain preserved. In conclusion, tissue degradation can occur either secondary to necrosis or primarily parallel to it.

Erdheim also examined the remaining inner media, intima, adventitia, the presence of vasa vasorum, and the area of dissection. In fact, necrosis played a less prominent role in the inner media. Areas of media loss were replaced by collagen deposits; these zones were smaller but more common. Elastic lamellae maintained a parallel orientation at specific intervals, but they thinned significantly due to subtle tissue degradation. Elastic fibers disappeared first without preceding necrosis, followed by SMCs. Concurrently, the connective tissue became denser, indicating an increase in collagen deposit. What remained was a connective tissue scant in nuclei, devoid of a layered arrangement. The partially restored media, rebuilt through regenerative processes, contains the same components as the original media but in an irregular composition or ratio. In contrast, the adventitia and intima were not primarily affected by the above-mentioned pathological changes, but rather secondarily. The proliferation of SMCs, which typically occur in diseases such as syphilis, was less pronounced. In the intima, hypertrophic SMCs were observed, arranged in an irregular pattern, indicating a regenerative process and the development of new or altered intima. Interestingly, the vasa vasorum was never found in the focal areas and was predominantly found in the outer medial layer, suggesting that it does not play a significant role. Finally, Erdheim studied the area of rupture or delamination. The tear zigzags through all wall layers, including zones of strong nuclear staining but significant medial degeneration, and through areas with necrotic foci. Examination revealed that the dissection was not immediate. In fact, the adventitia partially resisted and allowed the tear to penetrate easily.

In summary, the observed pathological findings results in reduced resilience of the aortic wall and a clear decline in its ability to perform the Windkessel function, as the degeneration of its two crucial components – elastic fibers and SMCs – is integral to the is integral to the recoil and contraction process. Elastic fibers recoil elastically due to their inherent restorative force when stretched – a purely mechanical process – while the actively stimulated SMCs perform the majority of the work driven in part by the nervous system. Moreover, Erdheim repeatedly emphasized that the described medial changes that lead to necrosis occur without an inflammatory reaction. This enigma was one of his main points of interest; he could not explain why there were no inflammatory reactions and associated cells despite significant changes. He was unsure of the causes of this novel disease and called it ‘idiopathic medial necrosis’.

One year later, in 1930, Erdheim examined a second patient case [370]. Unlike the previous case, medial necrosis was less dominant. Instead, the presence of scattered mucoid accumulation was highly significant, leading to a degeneration with cystic changes. The appearance of distinct cysts, predominantly filled with mucoid, gave this case a unique characteristic, as shown in Fig. 15(b). These cysts were mainly found in the medial layer of the tissue. As a result, he extended the previously introduced term to ‘idiopathic cystic medial necrosis’. As a side note, this term was originally coined by Babes and Mironescu [371] in 1910, but received little interest [271], until after 1928 it was Gsell [372] and then Erdheim who made this term public.

While Erdheim’s terms garnered significant interest and acceptance in the decades that followed, there were later considerable doubts as to whether this term accurately described the lesions or if cystic medial necrosis is in fact the most common lesion found in dissecting aneurysm [373]. The term ‘cystic’, proposed by Schlatmann and Becker [374,375] in 1977, seemed inappropriate because the lesions do not form true cysts with a distinct lining. Instead, they represent structural gaps or voids in the media, which become filled with semi-fluid ground substance due to intramural tension. In addition, the term ‘medial necrosis’ might not be entirely justified because, as Erdheim himself conceded, necrosis is rarely encountered in the lesions. Unfortunately, no specific terms have as yet gained full acceptance. Meanwhile, the term is often replaced by ‘medial degeneration’. Because cystic medial degeneration or necrosis indicates disparate histopathologies in different studies, it is often not possible to distinguish subtle histopathological differences between diseases [376]. Despite the different opinions based on the above observations, the term ‘cystic medial necrosis’ is still overwhelmingly used today.

While cystic medial necrosis has been identified in histopathological studies of tissue from patients with aortic dissection, it is certainly not unique to this condition. For example, a clinicopathological study of 513 consecutive ascending aortic surgical specimens revealed the presence of cystic medial necrosis in 40.7% of patients and in 56 (or 51.4%) of the 109 patients diagnosed with aortic dissection [377]. It was associated with connective tissue disorders, such as Marfan syndrome [378,379] and Ehlers-Danlos syndrome [380], or was found in patients with annuloaortic ectasia [381]. Furthermore, in patients with aortic stenosis [382] and, in addition, in patients without Marfan syndrome, cystic medial necrosis is more commonly found in geriatric and hypertensive individuals [383]. Several authors reported it in congenital disorders, instances of coarctation of the aorta and bicuspid aortic valve associated with cystic medial necrosis [379,384–386]. Other reported that in patients receiving surgical treatment for aortic dilatation with aortic valve disease, the incidence of cystic medial necrosis was 100% [387]. It is notable that patients with bicuspid aortic valve and aortic aneurysm exhibit cystic medial necrosis unrelated to age [388]. In summary, cystic medial necrosis is typically due to the fragility of the aortic wall due to degenerative changes, which may also be related to the normal aging process [376]. Nevertheless, it remains controversial whether cystic medial necrosis directly leads to aortic dissection, is merely a coincidental finding in the diseased aortic wall, or develops as a consequence of the dissection [389]. For a more detailed exploration of cystic medial necrosis, the reader is referred to [390], among others.

#### Medial degeneration.

In 2016, Halushka and his colleagues, all members of the Society for Cardiovascular Pathology and the Association for European Cardiovascular Pathology, made efforts to generalize the aforementioned histopathological terms [66]. Their use is now recommended for general surgical pathology reports and for categorizing histopathologies. Moreover, since the degree of medial degeneration can vary widely, ranging from minor to severe destructive alterations of the lamellar units in the aortic wall, a grading classification was introduced. The grading classification of these lesions is crucial as medial degeneration can vary significantly between patients. Overall, medial degeneration is a consequence of individual histopathological degenerative lesions.

**Fig. 16.**
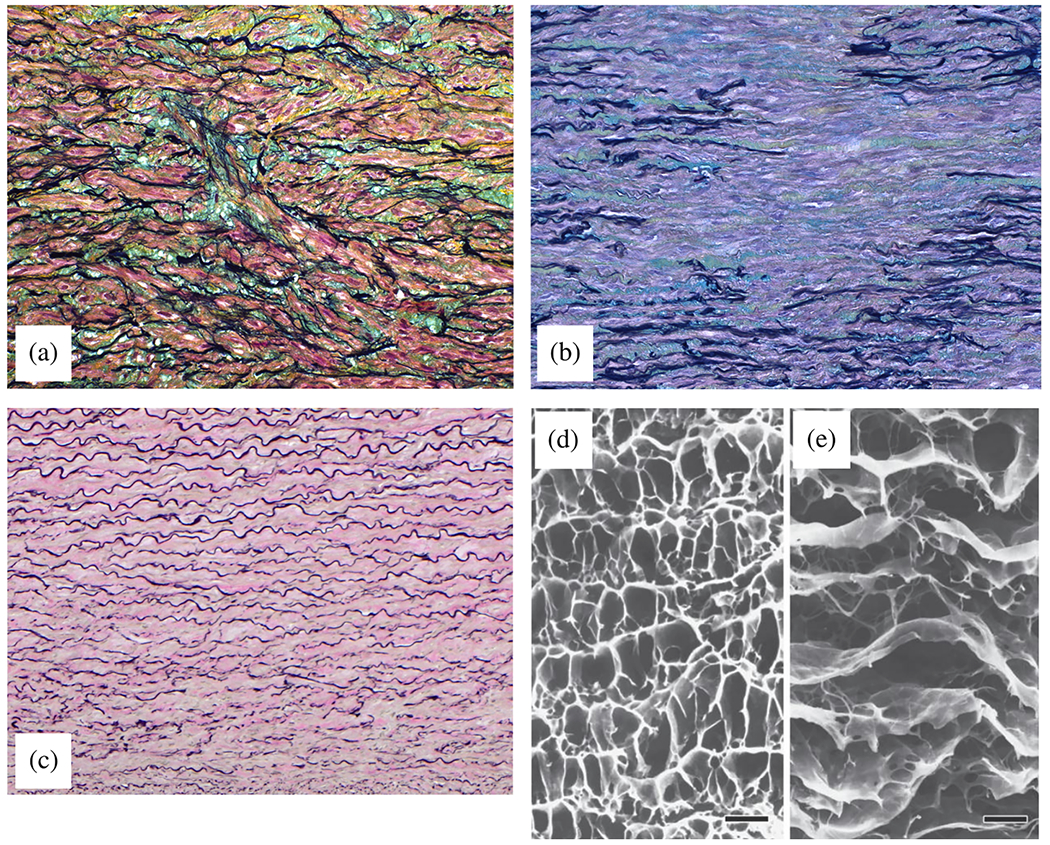
Demonstration of (a) fragmentation, loss, and disorganization of elastic fibers that no longer follow a strict circumferential course and may be oriented perpendicular to the lumen wall (400x, Movat pentachrome strain); (b) patchy-like loss of elastic fiber areas with possible complete loss of elastic fibers (400x, Movat pentachrome strain); and (c) thinning of elastic fibers (20x, Verhoeff-van Gieson strain). 3D architecture of the elastic fibers in (d) a normal subject and (e) an aortic dissection patient in which the aorta was treated with formic acid to remove components other than the elastic fibers and then examined using scanning electron microscopy. The top image represents the intimal side, the bottom the adventitial side (scale bar: 20μm). Figures (a) to (c) are adapted from Halushka et al. [66] with permission from Elsevier, while (d) and (e) are from Nakashima [400] (licensed under CC-BY-NC-SA 4.0).

**Fig. 17.**
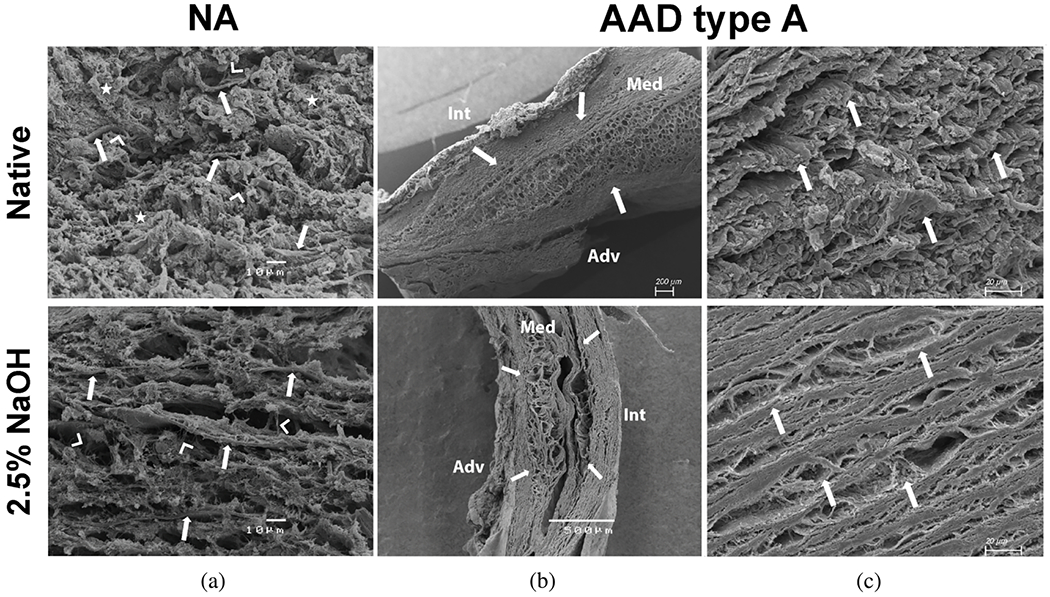
Scanning electron microscopy-based evaluation of elastic lamellar sheets and other extracellular matrix components in control tissue and tissue from dissection patients, with native tissue in the top row and decellularized tissue in the bottom row. (a) A representative scanning electron microscope image of control (‘NA’) tissue showing elastic fibers (white arrows), cellular material (white asterisks), and interlaminar elastin fibers (white arrowheads). Two representative scanning electron microscope images of dissection tissue illustrate (b) the dissection lamellar sheet (white arrow) before and after decellularization and (c) the extracellular structure adjacent to the dissection lamellae with white arrows pointing to melded thin fibers (bottom row). In the dissected media, a completely destroyed structure can be seen in the scanning electron microscope images. Thin individual extracellular matrix components cannot be clearly distinguishable and appear fused together, even with the elastic lamellae. Removal of the cell leads to the complete collapse of the elastic fiber structure. Figure is adapted from Mimler et al. [401] (licensed under CC-BY 4.0).

First, alterations in elastic fibers stand out as a prominent histopathological finding [93,391–401]. Specifically, reports indicated fragmentation or loss of elastic fibers. This implies the creation of increasingly extended translamellar spaces due to the absence of elastic fibers, along with an increase in gaps within the elastic fiber lamellae. This degradation of elastic fibers was often reported to be patchy in nature. In addition, thinning of the elastic fibers and noticeable disorganization was observed, the latter being due to the locally non-parallel arrangement of the elastic fibers. Examples of these findings are shown in Figs. 16(a) to (e) and Fig. 17, and were often observed in the context of SMC disorganization or local accumulation of GAGs.

**Fig. 18.**
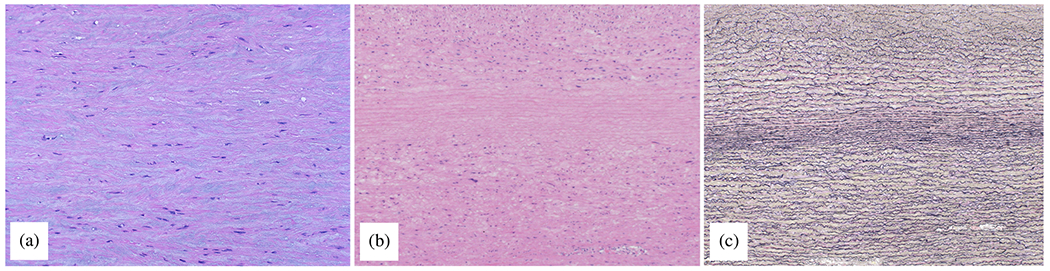
Histological images of (a) patchy and (b) band-like loss of SMC nuclei (100x, Movat’s pentachrome stain), and (c) laminar medial collapse in conjunction with a loss of SMCs in the lamellar units (200x and 160x, H&E stain). Figure is adapted from Halushka et al. [66] with permission from Elsevier.

The second finding is associated with apoptosis and dysfunction of SMCs. This loss of cell nuclei can manifest in patchy (Fig. 18(a)) or band-like patterns (Fig. 18(b)), a finding also known as ‘laminar medial necrosis’, see, e.g., [402–404]. In fact, this condition was not only observed in aortic dissection patients, but also in non-dissected and normal aortas [403–405]. It was strongly associated with aging and ischemic injury responses following aortic dissection [404,406]. This finding is similar to, but not identical with, ‘laminar medial collapse’, where the lamellar units collapse together, as shown in Fig. 18(c). In addition, a disorganization of SMCs was found, which is characterized by the non-parallel arrangement of the SMCs in the media, leading to focal disarray.

**Fig. 19.**
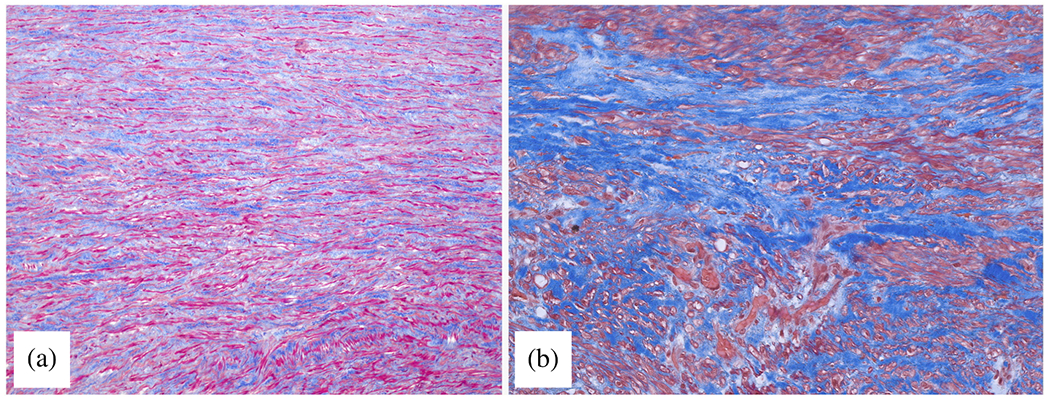
Histological images showing medial fibrosis: (a) intralamellar accumulation of collagen (royal blue) preserves the integrity of the lamellar unit and (b) translamellar fibrosis disrupts the lamellar unit and may be associated with disorganization of SMCs (100x, Masson’s trichrome stain). Figure is adapted from Halushka et al. [66] with permission from Elsevier.

Third, medial degeneration is also associated with remodeling of fibrillar collagen, possibly due to SMC dysfunction. In particular, a focal increase in collagen fibers was observed, leading to areas of substitutive fibrosis or a widening of intralamellar spaces in the media, a condition known as medial fibrosis, see Figs. 19(a) and (b). Additionally, collagen fibers often appeared thinner and disorganized. For more details, the interested reader is referred to the literature [399,407–411].

**Fig. 20.**
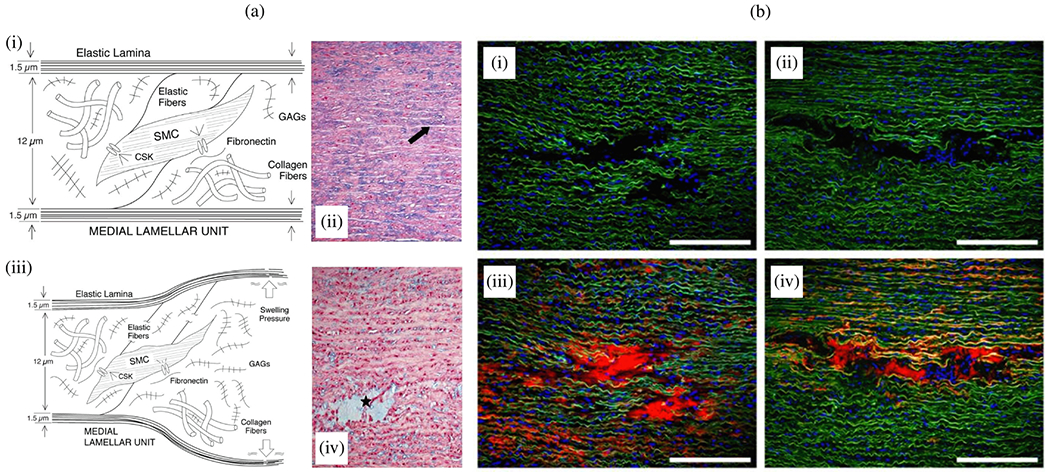
(a) Schematic representation of (i) a normal aortic medial lamellar unit composed of paired elastic laminae (top and bottom) encasing a SMC, and collagen fibers, adhesion molecules (e.g., fibronectin), and GAGs (not to scale). Also illustrated are thin ‘radially oriented’ elastic fibers (composed primarily of elastin and fibrillin-1 associated microfibrils) that may provide direct mechanical connections between the elastic laminae and the SMC, thus extending mechanosensory capabilities beyond the typical cytoskeletal (‘CSK’) – integrin – extracellular matrix (fibronectin/collagen) axis. Negatively charged GAGs may attract water, potentially contributing to normal intralamellar pressure that could help maintain tension in the thin elastic fibers. (ii) Previous image is accompanied by a histological image of the normal medial layer of the human ascending aorta with GAGs (blue) (Alcian blue stain), where the black arrow points to a normal structure, primarily consisting of SMCs, collagen, and modestly distributed GAGs. (iii) Schematic representation conveys the central hypothesis: localized accumulation of GAGs, on the right side of the medial lamellar unit, leads to increased Donnan swelling pressure. This may separate the elastic laminae and possibly disrupt connections between the SMCs and either thin elastic fibers or the collagenous matrix. Such effects could initiate local delamination and/or an altered mechanosensitive cellular response, resulting in dysregulated wall homeostasis. (iv) Medial layer of a human ascending aorta, stained with Alcian blue (which colors GAGs blue) for an aneurysmal wall. Note the significant accumulation of GAGs in the latter, indicated by the black star, and the absence of SMCs or elastic fibers within these GAG pools. (b) Areas of medial degeneration in cases of (i, iii) idiopathic aortic aneurysm and dissection, as well as (ii, iv) Marfan syndrome, are shown with and without intense aggrecan and versican staining (red). These areas are identified here as regions with fragmented or absent elastic fibers (stained green), and few SMCs (nuclei stained blue) in aortic aneurysms and dissections (scale bars: 200μm). Figure (a) is adapted from (i, iii) Humphrey [103] with permission from Karger Publisher and (ii, iv) Borges et al. [417] with permission from Elsevier, while (b) is from Cikach et al. [93] with permission from American Society for Clinical Investigation.

Finally, the accumulation of GAGs, or mucoid extracellular matrix, has been frequently observed in histopathological investigations; a unique feature that continues to be recognized as singularly characteristic of aortic aneurysms and dissections, see [374,375,412]. However, this occurrence was seen across a wide range of ages and in diverse disease origins [376,413–423]. For example, Cikach et al. [93] reported PG accumulation, including aggrecan and versican, in thoracic aortic aneurysms and dissections (Fig. 20(b)).

In 2013, Humphrey described for the first time a possible biomechanical consequence of pooled GAGs/PGs [103] in the context of aortic aneurysms and dissections. Accumulation of GAGs/PGs in the media, particularly in compromised regions of elastic fibers and SMCs, is frequently associated with elevated TGF-*β* activity. As previously mentioned, dysregulated TGF-*β* activity can lead to increased production of GAGs/PGs in the arterial wall, similar to what occurs in other tissues. Furthermore, the enzymes MMP-3 and −7, known for their role in degrading elastin and collagen III, have been associated with these accumulations and may play a role in medial degeneration. It was hypothesized by Humphrey [103] that the accumulation of GAGs, along with the associated Donnan swelling pressure that arises from negatively charged GAGs – a phenomenon well-documented in cartilage tissue [424] – could modify the intramural mechanical stress field, potentially causing aortic medial delaminations. For more details, the interested reader is referred to [103]. The swelling pressure, possibly between 16 and 170kPa [425,426], can be comparable to or even significantly higher than standard compressive stresses, which are typically in the range of −10 to −16kPa. Both experimental models of dissecting thoracic aneurysms and human studies indicated that regions with accumulated GAGs/PGs had larger separation distances between elastic lamellae and were associated with marked pooling of GAGs/PGs [420,427], as depicted in Fig. 20(a). This swelling could initiate the separation of elastic lamellae [428], potentially initiating dissections and disrupting matrix turnover and wall homeostasis by affecting cell signaling pathways. The structural integrity of the wall might further deteriorate due to elastin degradation caused by swelling. Furthermore, swelling-induced disruptions of smooth muscle interactions with ‘radially-directed’ elastic fibers, caused by swelling both before and after local dissection, could have negative effects on cellular mechanotransduction [394] and potentially lead to harmful alterations in the signaling pathways of the cells as well as the matrix turnover and the wall homeostasis [429]. Similar concerns were raised regarding disrupted mechanotransduction in dysregulated smooth muscle contractility [416]. Additionally, the production of degradation products from swelling-induced damage to inter-lamellar elastic fibers could destabilize wall homeostasis and exacerbate the loss of wall structural integrity [430].

Evidently, the histopathological findings associated with medial degeneration are not solely linked to aortic dissection. This suggests that there might not be a single histopathological finding that leads to aortic dissection in all cases. Instead, the specific severity and distribution of focal lesions might determine the initiation of dissection. However, the accumulation of GAGs and the associated mechanical impact could play a significant role. Some emphasized that it remains unclear whether PGs/GAGs contribute to disease onset or whether they react to early disease-related changes after the initial dissection event [431]. Notably, early studies, such as those by Erdheim, highlighted the non-inflammatory nature of this condition. However, assessing the role of inflammation prior to dissection in patients remains challenging. This is largely due to the difficulty of studying human tissue, which will likely soon be dissected, thus providing insight into the causes of disease. On the other hand, it is more common to acquire human tissue after dissection, which is likely to exhibit substantial inflammatory processes as part of the injury response. This topic will be discussed in more detail in Section 3.2.3.

#### Initiation of dissection.

The classic definition of the initiation of aortic dissection is as follows: aortic dissection is initiated as a small tear in the intimal (inner) layer of the aortic wall before it extends to the medial layer, causing a wall separation. Subsequently, the tear propagates along the axial direction of the medial layer resulting in a false lumen. The tear can be triggered by focal defects in the aortic wall, as previously described in this section, making it prone to delamination. Once dissection is initiated, it tends to propagate more easily within elastic lamellae than across the lamellae [432,433], a phenomenon clearly governed by the microstructure. However, there is a second scenario that has received significant attention in recent years. Instead, an intraluminal hematoma might cause the delamination of the medial layer, potentially initiating aortic dissection, which subsequently triggers an intimal tear at a later stage of the disease [103]. The aforementioned accumulation of GAGs, along with the related delamination process, may be of significant importance for this hypothesis. This controversy demonstrates that although we know much, this remains an unresolved question. Is the initial event a primary tear of the intima leading to secondary dissection of the media, or is the initiation of dissection due to an initial defect or delamination, leaving the wall vulnerable to subsequent dissection and possible rupture? Histological examination can provide evidence for both scenarios, suggesting that both cases could potentially occur. The wall appears to be riddled with various material and geometrical discontinuities, which could lead to the delamination of the media or even a fatal rupture of the aortic wall, depending on the size, distribution, and severity of the focal lesions. Material or geometric discontinuities can result in stress concentrations, which can typically increase local stress two- or threefold, initiating aortic dissection, as demonstrated later in Section 5.3.

Aside from the two primary dogmas determining the onset of dissection, another hypothesis has been notably overlooked. This hypothesis originates from the observation that vasa vasorum dysfunction due to malperfusion or leakage can accelerate atheroma formation, referred to as ‘outside-in progression of atherosclerosis’ [434,435]. Obstruction of the vasa vasorum is believed to result from viruses, bacteria, and tiny dust particles. This is likely influenced by risk factors such as hypertension, smoking, and age. It is crucial to understand that the rupture of the vasa vasorum and subsequent bleeding into the media play a role in the development of intramural hematomas, which could potentially lead to a subsequent dissection. Generally, malperfusion of vasa vasorum, exacerbated by the compressive forces of hypertension, can induce hypoxic conditions in the outer media. Such hypoxia is thought to stimulate angiogenesis of the vasa vasorum, a mechanism that yields new, potentially fragile and leaky neovessels, especially in the nascent stage. If the outer media remains in its hypoxic state, it can quickly become ischemic and promote necrosis and medial degeneration, which could result in aortic dissection [436,437]. As suggested by Haverich and Boyle [438], both aortic dissection and intramural hematoma could be different phases of the same condition: a vasa vasorum disease. This hypothesis rests on five key observations: (i) both conditions manifest at identical anatomical sites (ascending aorta and aortic arch); (ii) vasa vasorum dysfunction has been identified in both diseases; (iii) there have been instances where an intramural hematoma evolves into an aortic dissection, suggesting a two-stage disease; (iv) both conditions are characterized by medial necrosis and inflammatory processes; and (v) they share several common risk factors.

Although the precise initiation event remains elusive, it appears improbable that the majority of aortic dissection cases are triggered by vasa vasorum dysfunction. Undoubtedly, a rupture in the vasa vasorum can lead to intramural hematoma, which eventually results in an aortic dissection if an intimal tear develops. However, a rupture in a vessel with a radius in the micrometer range is mechanically unlikely because the wall tension is miniscule; it should also be taken into account that the vessel is surrounded by a high pressure environment. Although this can occur, it is probably not the cause for the majority of a dissections. Moreover, while it is undeniable that aortic dissection and intramural hematoma are similar diseases, they are in general not related to the vasa vasorum. Nevertheless, the underlying condition points to medial degeneration with obvious loss of cohesive forces between the medial layers. However, the arguments postulating vasa vasorum as the primary cause are often weak and rarely discussed in the literature; one of the few reports on this was published by Osada et al. [439], and such arguments have never been conclusively proven. Moreover, if bacteria invaded the aortic wall, the likelihood of aortitis [440] or mycotic aneurysms [441] would be greater. Only a few studies, mainly related to coronary arteries, provide interpretable results in this context [442,443].

#### 3.2.2. Layer-specific wall remodeling

#### Dissection flap.

The dissection flap separates the true from the false lumen and balances the pressure differences between them. Especially in acuity, it moves over the cardiac cycle driven by the pressure difference, as described above. Typically, the dissection flap consists of the intima and part of the media. Immediately following dissection, one might anticipate that the dissection flap and the outer wall of the false lumen would be roughly equal in thickness to the outer wall of the true lumen. However, CT imaging often reveals a thicker dissection flap, especially in chronic aortic dissection. Due to remodeling processes, the dissection flap gradually becomes thicker and loses mobility as the aortic dissection progresses [14].

Studies have reported that the thickness of the dissection flap can sometimes exceed the thickness of the outer wall of the true lumen. This phenomenon may be attributed to the recoil of elastic fibers [275,282] due to the disappearance of transmural pressure in the dissection flap, leading to a loss of circumferential tension. However, axial tension may not necessarily be affected. It has also been shown that the thickened dissection flap results from the formation of a neointima, see, e.g., [258]. The neointima is a newly formed intimal layer in the false lumen that develops in response to the absence of an intima in the false lumen, similar to the reaction to injuries [444]. Neointima formation begins within a few hours of the dissection onset and involves the replication of SMCs in the media and their migration through the internal elastic lamina to form the neointima alongside an ECM. This process can significantly affect the thickness of the flap. An example of neointimal formation in the false lumen is presented in Section 4.1 (Fig. 35(b)).

**Fig. 21.**
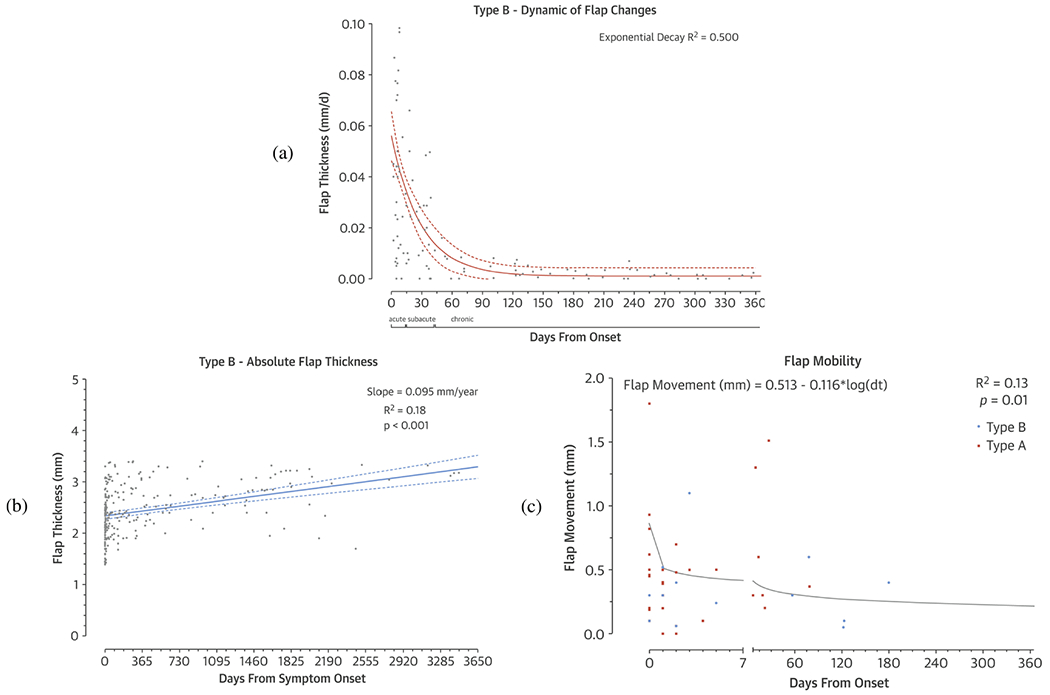
(a) Absolute thickness and (b) thickening rate (mm/day) of the dissection flap, which follows an exponential decay function with a rapid decline in the initial thickening rate, a deceleration starting at 83 days, and a plateau after 235 days after start. (c) Dissection flap movement (mm) in type A and B dissection, with a gradual decrease in flap movement over time. Figure is adapted from Peterss et al. [14] with permission from Elsevier.

Peterss et al. [14] characterized the increase in dissection flap thickness as an exponential decay over time, with the change in thickness decelerating at approximately 83 days and reaching a plateau at approximately 235 days after the onset of the aortic dissection (Figs. 21(a) and (b)). They estimated the average change in dissection flap thickness per year, which amounted to 1.2mm during the acute stage, 0.41mm in the subacute stage, and 0.02mm in the chronic stage. A thickened dissection flap is strongly associated with altered elasticity and mobility (Fig. 21(c)). The change in stiffness can be attributed to fibrosis, which is associated with decreased mobility. Furthermore, studies using CT and MRI have shown that, over time, the dissection flap loses its curvature and waviness, appearing more straightened [14,278,445,446]. While the dissection flap remains elastic and flexible during the initial days following the onset of aortic dissection, it becomes stiffer and thicker within the first few weeks and months. Karmonik et al. [447] demonstrated that this decreased motion is observed in both short-term and mid-term examinations, with high variability between patients in the short term and low variability in the mid-term. The short-term variability among patients is high but decreases during mid-term examinations. The pathological changes in the dissection flap are crucial indicators of chronic aortic dissection, but their systematic examination is still inadequate. Tang and Dake [283] discuss how the success of stent-graft deployment depends on an understanding of the dissection flap configuration. As time progresses, the dissection flap matures, becoming thicker and more stable. This increased stability facilitates the deployment of medical devices and reduces the risk of requiring surgical re-intervention. Bäumler et al. [448] point out that it is clinically important to determine whether the stiffness of the dissection flap and its reduced motion correlate with an increase in intraluminal traction. The pressure difference between the true and false lumina causes intraluminal traction, which can increase as the dissection flap becomes stiffer and can no longer balance the pressure difference. However, the detailed microstructure of the dissection flap remains largely unknown and has rarely been investigated.

#### Outer wall of the false lumen.

The outer wall of the false lumen serves as a load-bearing structure and arises from the intact aortic adventitia after the initial delamination of the intima and variable portions of the medial layer within the aortic wall. The hemodynamics and resulting loading conditions can vary depending on the size of the false lumen, and the location, number, and size of intimal tears. In most cases, the maximum pressures in the false lumen are lower than in the true lumen. Post-dissection, it is often observed that the outer wall of the false lumen is thin and expanded. For example, Roberts [261] reported that the thickness of the outer wall of the false lumen is approximately one-quarter of the outer wall of the true lumen. However, this changes over time. An early response is neointimal formation in the false lumen, leading to thickening over time.

Due to altered mechanical stresses within the false lumen, the thin wall may respond to the changed loading conditions by undergoing remodeling, potentially becoming thicker. A chronic case example was shown in Section 3 (Fig. 9(a)). The restoration of false lumen wall strength and homeostasis is maintained by adventitial fibroblasts, which are sensitive to changes in wall tension. These mechanosensing fibroblasts respond with proliferation, increased synthesis, and cross-linking of collagen. This was observed at late surgical intervention as a strong, leathery thickening of the adventitial layer. Even a marginally inadequate tissue response to wall tension can initiate a vicious cycle in which any increase in false lumen diameter proportionally translates into ever-increasing wall tension, resulting in continuous growth of the aortic diameter over time. Once restorative capabilities are exhausted and wall tension exceeds tensile strength, structural failure and aortic rupture follow. This occurs with increasing frequency when the maximum aortic diameters exceed 55mm in men and 60mm in women, respectively [34,301]. However, the effects of this remodeling on the microstructure remain largely unclear.

**Fig. 22.**
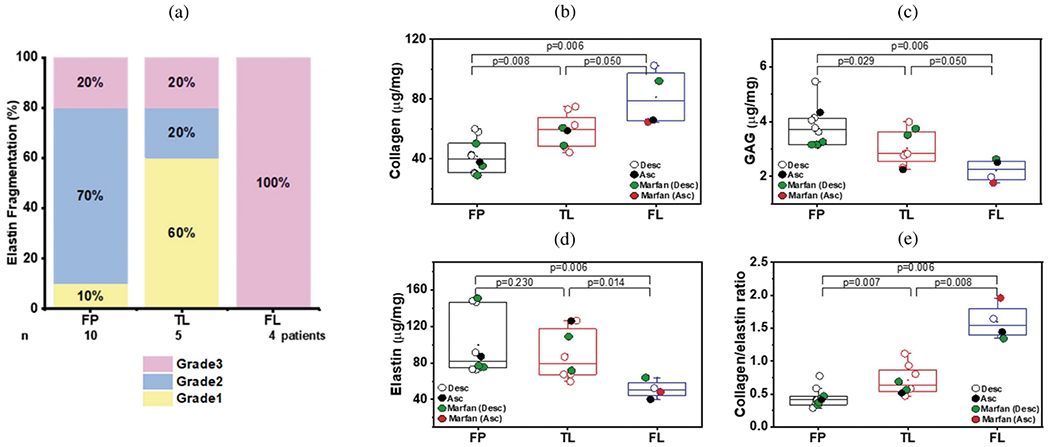
Biochemical analysis of the dissection flap (‘FP’), outer wall of the true lumen (‘T’), and outer wall of the false lumen (‘FL’): (a) overall percentages of elastin fragmentation, graded by age for each layer, were determined using criteria modified from Schlatmann and Becker [374], and, additionally, boxplots illustrate the statistical significance of (b) collagen levels, (c) GAG levels, (d) elastin levels, and (e) the ratio between collagen and elastin across patient groups and sample regions: descending (‘Desc’) and ascending (‘Asc’) regions in aortic dissection patients with and without Marfan syndrome. Figure is adapted from Panpho et al. [449] (licensed under CC-BY 4.0).

Recent biomechanical investigations on the outer wall of the false lumen showed an increase in collagen and a decrease in elastin due to fragmentation, see Fig. 22. When mechanically loaded, this results in an initially very compliant behavior due to the lack of elastin, which is responsible for the load-bearing at lower stretches. At higher stretches, the outer wall of the false lumen exhibits then a significant increased stiffness. Despite the lack of extensive mechanical and microstructural studies, the results of Amabili et al. [250] confirm this finding.

#### Mechanical behavior.

Mechanical tests are required to determine the layer-specific mechanical behavior of tissue samples from aortic dissection patients. The currently available experimental studies on such tissue are summarized in Table 3. These studies involve uniaxial and biaxial extension tests and may selectively examine specific layers, including the outer wall of the true lumen, the dissection flap, and the outer wall of the false lumen, either individually or a combination thereof. This layer-specific approach is essential, as each layer may undergo distinct remodeling processes during the progression of an aortic dissection, influenced by the loads they experience and their unique microstructures, which may be altered by inflammatory responses triggered by associated wound healing. Notably, these studies fail to compare mechanical behaviors between tissue samples from acute and chronic patients, whose mechanical properties can differ significantly due to remodeling. Currently, the data primarily reflect passive behavior; active behavior has not yet been characterized. Given the rarity of data on the active behavior of the normal human aorta [450] and considering that tissue cells die within hours of death, it is unlikely that such data will be obtained from patients with aortic dissections in the near future. For more details on active behavior in healthy or normal human tissue, see, e.g., [451].

Although some review articles also included histological examinations, comprehensive information on fiber alignment and dispersion in dissected tissues is lacking. It should also be noted that some studies in Table 3 focus primarily on material or computational modeling and use a limited number of tissue samples to determine relevant material parameters [450,458]. Additionally, follow-up studies used published experimental data on dissected tissue to identify material parameters for specific constitutive models, as shown by [461,462]. Note also that we omitted a summaiy of delamination and failure tests on human and animal tissues, as these topics have been extensively summarized and reviewed in recent literature, see, e.g., [17–19]. It is important to recognize that these tests did not involve tissues from aortic dissection patients, who may exhibit different delamination characteristics due to potentially reduced cohesive forces between layers. Nevertheless, such tests offer valuable insights into the general behavior of delamination and its underlying mechanisms.

#### Role of mouse models.

Although the ultimate goal is to understand dissection of the human aorta, mouse models have emerged as important, which is due primarily to the availability of relevant genetically modified mice. Motivated by mutations that predispose humans to thoracic aortic aneurysms and dissections, mouse models are available for Marfan syndrome, Loeys-Dietz syndrome, and vascular Ehlers-Danlos syndrome, among others [191]. Moreover, these mice can be rendered hypertensive using different approaches (e.g., chronic infusion of angiotensin II, high salt combined with endothelial nitric oxide inhibition, and transverse aortic constriction), which increases hemodynamic loading and thereby risk for dissection. For studies on mouse models, we refer to the work of Trachet and his colleagues, which offer exemplary research on angiotensin II-infused *Apoe*^−/−^ mice for preclinical investigations of aneurysms and dissections [463–467]. Importantly, mouse models enable much greater control of the altered hemodynamics and more convenient studies of the natural history. As in humans, lesions tend to present in the thoracic aorta, but have also been found in the suprarenal abdominal aorta in the angiotensin II model. Noting that mutations to lysyl oxidase also predispose to thoracic aortopathy, an inhibitor of lysyl oxidase (*β*-aminopropionitrile) has also been used to increase disease risk [226].

**Fig. 23.**
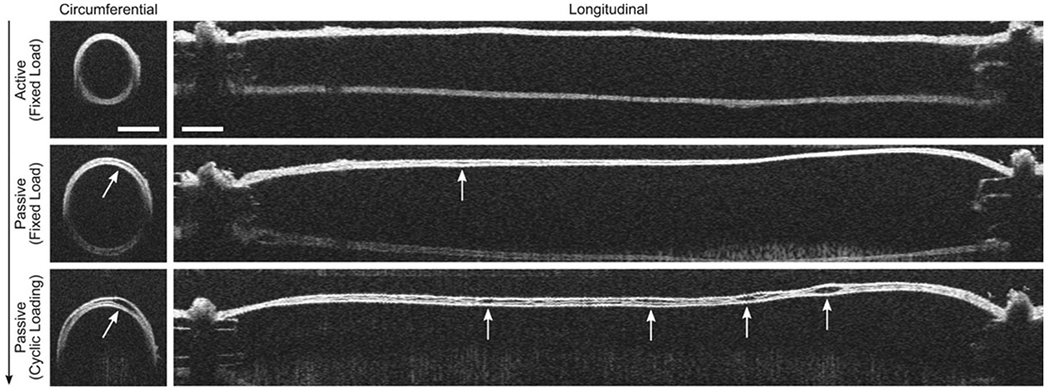
Optical coherence tomographic images display circumferential and axial (here: longitudinal) sections of a representative cannulated artery of a mouse model during biaxial mechanical tests on a rapamycin-treated, *Tgfbr2* disrupted descending thoracic aorta subjected to 70mmHg luminal pressure at *in vivo* axial stretch. The artery underwent vasoconstriction (top row) followed by a 40 min vasodilation using multiple washouts (middle row) and finally experienced cyclical pressurization from 10 to 140mmHg under passive conditions (bottom row). Intramural delaminations appeared locally upon SMC relaxation and propagated along the length and circumference, occurring most frequently in rapamycin-treated, *Tgfbr2* disrupted samples. This phenomenon was absent in control samples and occurred in only one non-treated *Tgfbr2* disrupted sample. White arrows indicate the initiation and progression of delamination, while the dark arrow on the left side shows the temporal progression of the experiment. Scale bars (top row) represent 400μm. Figure is reprinted from Ferruzzi et al. [474] with permission from Wolters Kluwer Health, Inc.

Given the small size of the murine aorta, the emergence of mouse models of thoracic aortopathy has driven the development of new multimodality, multiscale methods for studying the biomechanics of the aorta. Such methods have employed or combined various techniques, including micro-CT, high-resolution ultrasound, optical coherence tomography, and panoramic digital image correlation [468–472]. Fig. 23 shows an optical coherence image from a study of *Tgfbr2* null mice, depicting significant intramural delamination of the wall under physiological loading. Importantly, this delamination initiated once the aorta was rendered passive, suggesting that active stresses associated with SMC contraction can be partially protective by reducing overall stress in the aortic wall. Compromised actomyosin activity is a characteristic feature of many genetic mutations that give rise to thoracic aortopathy, see Table 2, including Marfan syndrome and the familial conditions resulting from *Myh11* and *Acta2* mutations, with associated mouse model (*Myh11* and *Acta2*) mutants available. See for example a biomechanical comparison across multiple mouse models that revealed increased circumferential material stiffness as most highly correlated with increasing diameter [473]. Continued studies in mouse models are thus warranted.

#### 3.2.3. Roles of inflammation and proteases

**Fig. 24.**
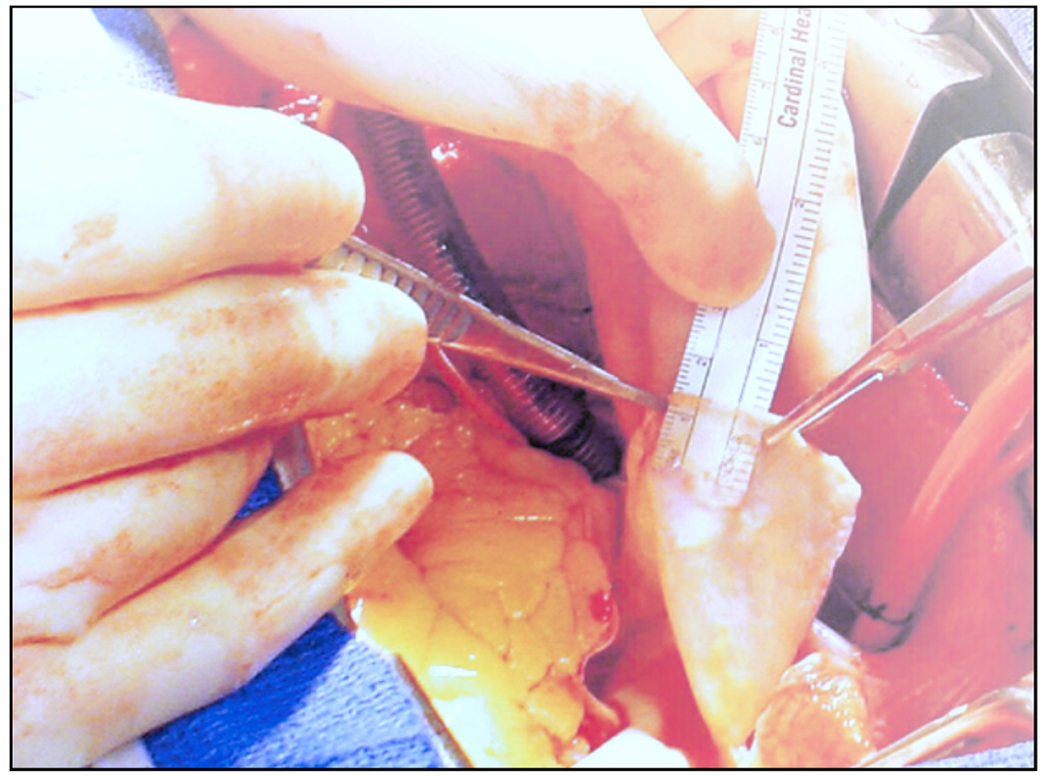
Aortic wall from an ascending aneurysm that has been so strongly degraded that one can read the markings on a ruler through the aortic wall. It is frightening to envision this thin aortic wall restraining the bloodstream and the systolic blood pressure.

As noted above, diverse molecular mechanisms are thought to contribute to degeneration of the thoracic aortic wall, ultimately resulting in aneurysms and dissections [475]. These mechanisms lead to histologic disruption, including the appearance of ‘cystic spaces’ among the lamellar layers (see Section 3.2) and wall thinning (see Fig. 24). Such spaces may provide a substrate for blood to enter and disrupt the lamellar structure of the aortic wall, resulting in propagating aortic dissection. In this section, we will concentrate on two additional important processes that disrupt the aortic wall: inflammation and proteolytic activity.

#### Inflammation.

**Fig. 25.**
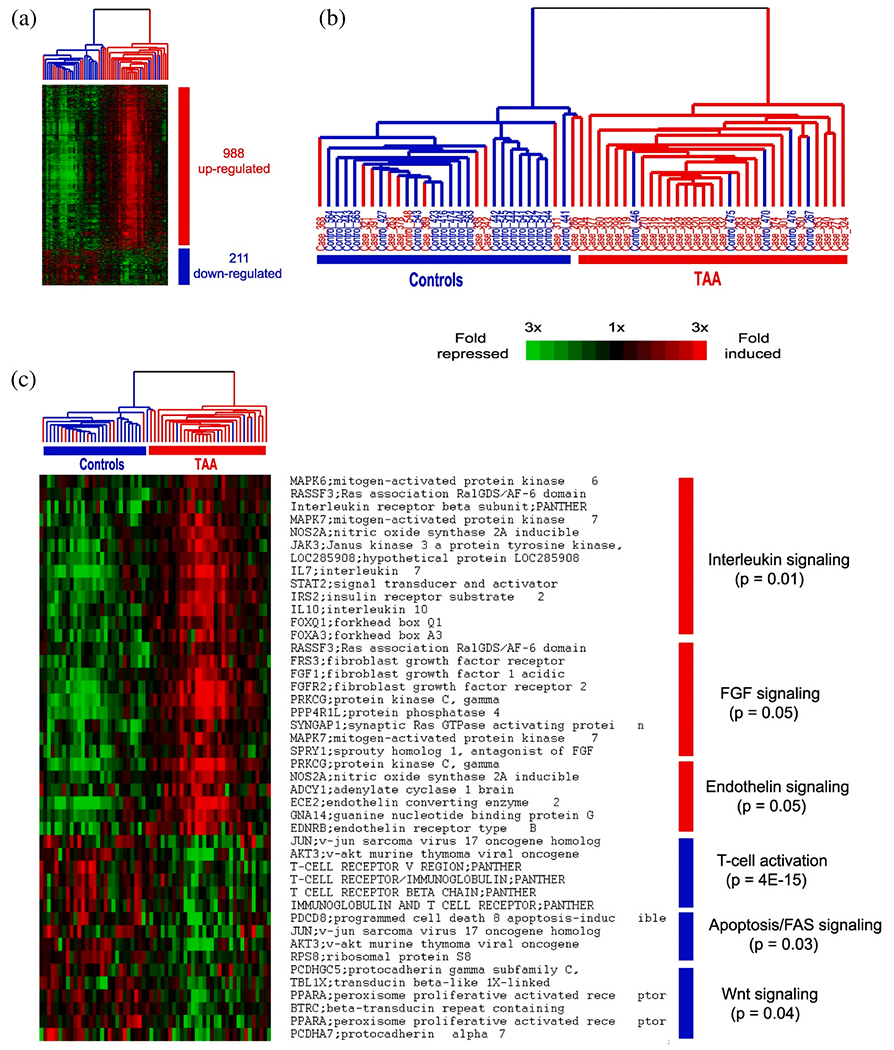
Hierarchical clustering of 61 whole blood samples analyzed using the 1, 199 differentially expressed genes determined by significance analysis of microarray. The level of expression of each gene in each sample, relative to the mean level of expression of that gene across all the samples, is represented using a red–black-green color scale as shown in the key (green: below mean; black: equal to mean; red: above mean). (a) Scaled down representation of the entire cluster of the 1,199 signature genes and 61 whole blood samples. (b) Experimental dendrogram displaying the clustering of the samples into two main branches: the thoracic aortic aneurysm branch (red) and the control branch (blue) with a few exceptions. (c) Gene expression pattern of representative genes within biological pathways that are statistically significantly over-represented (random overlapping *p*-value, 0.05) by the up-regulated (red bars) or the down-regulated (blue bars) signature genes of thoracic aortic aneurysm. Note departure from control levels in interleukin signaling and T-cell activation pathways. Figure is reprinted from Wang et al. [484] (licensed under CC-BY 4.0).

It has long been recognized that inflammation plays an important role in diverse aspects of degeneration of the aortic wall as well as in the body’s response to injury, including aortic dissection [476]. This evidence of the importance of inflammation has accumulated over decades, especially regarding abdominal aortic aneurysm, dating from an initial key publication by Tilson and his colleagues who found evidence for both humoral and cellular influences [477]. Elastin degradation products appeared to be at least one important target for an excessive immune reaction. Whereas inflammation clearly plays a significant role in disease progression in abdominal aortic aneurysms [478,479], which tend not to dissect, the role of inflammation in the pre-dissection diseased thoracic aorta is less definitive but emerging [480]. Tellides and his colleagues implicated a role of IFN-*γ* producing T-cells in outward remodeling of the thoracic aorta [481], while Milewicz and her colleagues demonstrated that T-lymphocytes and macrophages contribute to the pathologic process, with interleukins and interferon subtypes playing major roles [482,483]. Wang et al. [484] also found interleukin signaling and T-cell activation pathways to be upregulated in patients with ascending aortic aneurysms, see Fig. 25.

Heightened inflammation and the corresponding immune responses have also been implicated as a possible instigating factor for acute aortic dissection [485]. Infiltrating immune cells can have myriad effects, however, including promotion of fibrosis in the adventitial layer [486]. An exuberant accumulation of collagen in the adventitia reduces aortic function (compliance and resilience) but can serve to protect against transmural rupture, perhaps contributing to non-lethal dissection by leading to contained rupture and false lumen dilatation. By contrast, inflammation necessarily plays a central role in the post-dissection thoracic aorta – accumulation of blood within the medial layer provides an unencumbered inlet for monocytes and leukocytes, which are expected to play a role in the attempted ‘wound healing’ response. A large study from China suggested that, given the inflammatory nature of aortic dissection itself and the vigorous inflammatory response to open heart surgery for treatment of acute type A dissection, treatment with the inflammation inhibitor ulinastatin might be beneficial post-operatively [487]. Some supportive evidence was amassed in a large multicenter clinical trial. There is, however, a need for caution – to balance immuno-suppression that reduces disease progression versus that which attenuates inflammation-mediated tissue repair. Much remains to be learned regarding the myriad roles of inflammation in thoracic aortopathy and its treatment.

#### Proteases.

The accumulation of blood within the medial layer not only allows an influx of inflammatory cells, it also promotes an intramural thrombotic response [488,489]. The serine protease plasmin plays a central role in subsequent fibrinolysis, that is, resolution of the fibrin mesh within the thrombus. Plasmin plays many other roles as well, including loss of vascular SMCs, proteolysis of adhesive proteins, release of sequestered TGF-*β* from the extracellular matrix, and activation of MMPs, all of which have been implicated in thoracic aortic aneurysms and dissections [490]. There exist over 20 different MMPs, many of which are produced by the different cells that are involved in aortic remodeling, repair, and disease progression, including SMCs, fibroblasts, and of course macrophages [491]. The ultimate effects of MMPs on the extracellular matrix of the aortic wall depend on many factors, including their increased production in a latent form (precursors of MMPs), activation by blood pressure-induced mechanical stresses within the wall, cell-mediated tractions, and other proteases, and inhibition by tissue inhibitors of MMPs (i.e. TIMPs).

Nearly two decades ago, Koullias et al. [398] evaluated MMP and TIMP levels in aortic tissue specimens from patients with ascending aortic aneurysms and dissections compared with control aortas. They found a significant increase in expression of MMP-1 (a collagenase) and MMP-9 (a gelatinase) in aneurysm and dissection patients compared with controls. Aortic dissection patients manifested even higher MMP-2 (a gelatinase) and MMP-9 levels that non-dissected aneurysm patients. The ratio of MMP-9 (proteolytic) to TIMP (proteo-protective) was elevated in both the aneurysm and dissection patients, thus indicating a net excess tendency toward proteolysis. Since that early study, overwhelming evidence has accumulated that excess MMP activity, coupled with decreased activity of TIMPs, results in net degeneration of the extracellular matrix, with accompanying loss of mechanical strength of the wall [492–494]. This is vividly evident in the extraordinarily thin aortic wall seen in Fig. 24.

Finally, the aforementioned marked accumulation of mucoid material within the degenerated thoracic aorta, historically associated with cystic spaces, can play multiple adverse biomechanical and biological roles [93,103]. This accumulation of mucoid material, that is, aggregating GAGs/PGs, implies either heightened production or reduced removal, or both. The ADAMTS (a disintegrin and metalloproteinase with thrombospondin motifs) family of proteases play key roles in the turnover of GAGs and PGs [495], and some have been implicated in thoracic aortic aneurysm and dissection [496,497].

In summary, extracellular matrix turnover plays a key role in aortic homeostasis and its loss [498] and there is a pressing need to understand better the effects of diverse proteases (e.g., serine, MMPs/TIMPs, or ADAMTS family) and how their regulation or dysregulation contributes to the structural integrity of the wall as well as the extracellular matrix ligands that are presented to the resident vascular cells and influence their gene expression. Much has been learned, but much remains to be discovered regarding roles of both inflammation and proteolysis.

### 3.3. Changed hemodynamics

Hemodynamics in aortic dissection has a significant impact on patient outcomes and influences aortic dilatation, organ malperfusion, thrombus formation, remodeling processes, and more. Therefore, it plays a crucial role in the progression of the disease and possible complications and should also be considered in treatment strategies. Despite recent advances in medical imaging, hemodynamics is usually not considered in everyday clinical practice; rather, it is performed exclusively for research purposes. In general, blood flow velocity in the true and false lumina can be determined using advanced imaging methods such as 4D-flow MRI [499,500], as introduced in Section 2.3.1. These mostly non-invasive techniques enable the assessment of anatomical and hemodynamic features of the dissected aorta. Furthermore, they allow evaluation of hemodynamics in true and false lumina using calculated parameters such as WSS, kinetic energy, flow helicity, and stasis. However, note that, for instance, the assessment of WSS depends heavily on the accuracy of the surface reconstruction, which is still very low and therefore often error-prone due to the spatial resolution of 4D-flow MRI [163–165]. Accurate hemodynamic data can, in turn, be used to validate *in silico* models, which will be discussed in the following sections. Readers are also referred to the review article by Sherrah et al. [32] for a systematic overview.

In 1988, after an earlier study by Mohri et al. [501], Bogren et al. [502] investigated flow rates in three patients with chronic aortic dissection and observed that antegrade flow rates in the false lumen were low and remained constant throughout the cardiac cycle. In contrast, they observed high flow rates during systole and normal flow rates during diastole in the true lumen. In the same year, Mitchell et al. [503] reported similar observations in a study analyzing blood flow patterns in a single patient with chronic aortic dissection. They found antegrade flow in both lumina during peak systole, albeit at a reduced rate in the false lumen. During late systole, antegrade flow persisted in the true lumen, while retrograde flow occurred in the false lumen. In diastole, flow nearly stopped in both lumina. A delay in the maximum flow rate in the false lumen compared with the true lumen was observed, possibly due to the presence of an intimal tear. Mohr-Kahaly et al. [504] subsequently performed imaging in a larger patient cohort, comparing blood flow patterns in 18 patients with and without prior aortic dissection surgery, focusing on type I, II, and III dissections. They detected systolic laminar flow in the true lumen, while in the false lumen, blood flow was observed only during late systole and diastole. Two distinct blood flow patterns in the false lumen were identified: laminar biphasic flow and slowly circulating flow. Some patients exhibited delayed laminar flow in the false lumen during systole, which moved distally and reversed during diastole. Others displayed a slowly circulating flow in the false lumen, characterized by a ‘swirling pattern’. Blood flow was found to be influenced by the location, number, and size of intimal tears, which impacted the pressure difference between lumina. Unidirectional flow was observed in proximal intimal tears, while bidirectional flow occurred in more distal intimal tears. Flow from the true to the false lumen was detected during systole and reversed during diastole. Chang et al. [499] measured mean flow velocity and peak systolic velocity in the true and false lumina. They recorded a mean velocity of 134±14.0mm/s in the true lumen and 31±8.4mm/s in the false lumen, with peak systolic velocities of 436±72.0 and 143±23.0mm/s for the true and false lumina, respectively. However, no significant differences were observed in the average flow rate between the true and false lumina, which could be attributed to the larger size of the false lumen compared with the true lumen.

The finding of the studies discussed were then confirmed by other publications in the twenty-first century. In the study by Strotzer et al. [505], 14 patients were examined to determine the mean flow rate and average peak velocities of the true and false lumina. The mean flow volume per minute was found to be 1982ml in the true lumen and 1052ml in the false lumen. Similarly, the average peak velocities were significantly higher in the true compared with the false lumen, with values of 980 and 470mm/s, respectively. Additionally, ten patients exhibited bidirectional flow in the false lumen, with a retrograde flow ranging from 6.8 to 98%. The false lumen displayed an early systolic peak and a delayed systolic negative peak, while the blood flow in the true lumen was unidirectional.

Subsequently, Markl et al. [506] analyzed the blood flow in the thoracic aorta for both healthy subjects and those with a diseased aorta, one of which exhibited an aortic dissection. The quantitative results revealed that there was almost no blood flow in the false lumen throughout the cardiac cycle. In contrast, the blood flow velocity in the true lumen was higher than in the aortic outflow region due to the larger area. Additionally, the authors observed a retrograde flow in the entire ascending aorta during diastole.

**Fig. 26.**
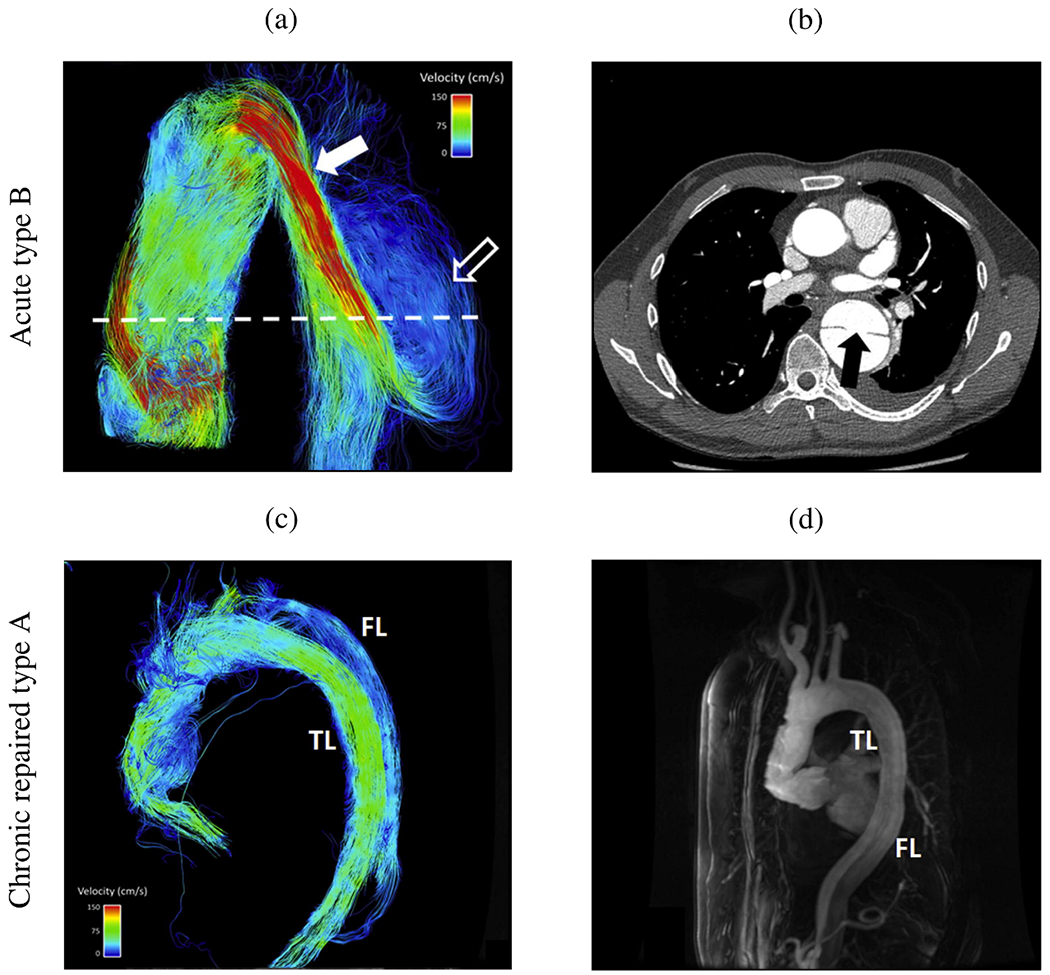
A representative case of acute type B dissection shows (a) a color-coded streamline visualization from 4D-flow MRI during mid-systole, highlighting flow acceleration (open arrow) in the proximal descending aorta directed into the false lumen through a large intimal tear ((b), black arrow), seen on axial CTA at the indicated level (dashed line), with false lumen flow characterized by a large vortex ((a), white arrow), resulting in predominantly retrograde flow. A representative case of chronic repaired type A dissection shows (c) a color-coded streamline visualization during mid-systole revealing slow flow through the false lumen (‘FL’) compared to the true lumen (‘TL’). This can also be seen in the subtracted maximum intensity projection images (d) acquired with time-resolved contrast-enhanced MRA. Figure is adapted from Francois et al. [159] with permission from Elsevier.

François et al. [159] investigated flow patterns in 12 patients. They found higher total flow and peak flow in the true lumen compared with the false lumen. Furthermore, they characterized the flow pattern in the false lumen as complex, abnormal, and non-laminar, with a higher retrograde flow than in the true lumen. In contrast, the true lumen primarily exhibited laminar flow. As a representative patient case, Figs. 26(a) and (b) show a visualization of flow acceleration in the proximal descending aorta, while Figs. 26(c) and (d) illustrate low flow velocities in the false lumen.

**Fig. 27.**
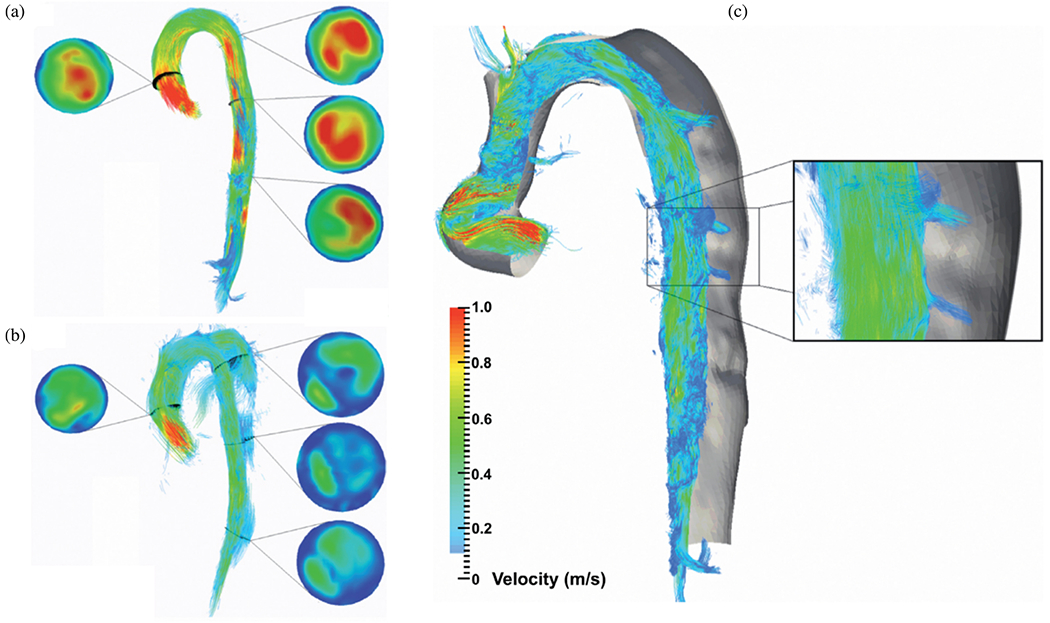
Visualization of sagittal pathline views in the isolated thoracic aorta during peak systole within one cardiac cycle: (a) from a control patient, (b) from a chronic type B dissection patient with isolated pathlines for both the true and false lumina, and (c) showing the pathline isolated for the true lumen in a representative patient with chronic type B dissection, specifically highlighting blood flow through the fenestrations connecting the true and false lumina. Figure is adapted from Sherrah et al. [507] (licensed under CC-BY 4.0).

In the study by Sherrah et al. [507], ten patients with chronic type B dissection were examined after undergoing surgical repair of ascending aortic dissection. The findings showed that, at peak systole, the overall flow profile in the false lumen was lower than in the true lumen and normal aortas (Figs. 27(a) and (b)). Additionally, the peak systolic flow rate per aortic lumen area was lower in the false lumen compared with the true lumen, and both rates were lower than those observed in control aortas. Patients experienced higher retrograde flow in the false lumen than in the true lumen throughout the descending aorta. Moreover, the derived pulsatility index was higher in the true compared with the false lumen among all patients. Briefly, the pulsatility index equals the difference between the peak systolic flow velocity and the end diastolic flow velocity, divided by the mean flow velocity. Thus, it is a dimensionless parameter that provides insight into the resistance to blood flow and the pulsatility of the blood flow waveform. The pathline images generated in the study provided detailed views of the flow patterns in both lumina, including the fenestration sites between the true and false lumina, as shown in Fig. 27(c).

Liu et al. [508] analyzed the hemodynamic characteristics within the true and false lumina in 16 patients with aortic dissection of different types. They found that peak flow velocity and peak flow rate, among other parameters, were substantially higher in the true lumen. Furthermore, they identified a negative correlation between the size of the intimal tear and both the flow velocity and flow rate in the true lumen, as well as a positive correlation with the average through-plane velocity, average net flow, and peak flow rate in the false lumen. These results indicate a direct relationship with the number and size of intimal tears. Additionally, the blood flow indices in the true lumen were enhanced with an increase in the number of intimal tears, and the peak flow in the false lumen was reduced. In cases where false luminal thrombi were present, the average through-plane velocity and peak velocity magnitude in the true lumen were found to be significantly higher. To conclude, they posited that blood flow velocity, flow rate, flow pattern, and pressure differences between the true and false lumina are crucial factors affecting the collapse of the true lumen and dilatation of the false lumen.

Allen et al. [509] conducted a study on 19 patients with type B dissection in which active fenestrations were assessed hemodynamically. They examined the number and location of fenestrations, as well as the direction of flow. The results indicated that different methods detected different numbers of fenestrations. More fenestrations resulted in biphasic flow over the cardiac cycle, with flow entering the false lumen during systole and exiting during diastole. Antegrade flow was most apparent in large fenestrations, while retrograde flow from the false to true lumen (diastolic flow) was often useful in identifying smaller fenestrations.

In a longitudinal case study by Takei [510], which focused on patients with chronic type B dissection, changes in flow direction and rate within the aortic lumen, as well as at entry and fenestration sites, were analyzed. They conducted this analysis following entry closure using thoracic endovascular aortic repair. The study assessed one entry tear and four fenestrations. Before the operation, the entry site produced a significant antegrade flow toward the four fenestration sites. However, shortly after the closure of the entry site, fenestration sites one to three transformed into a new entry site, exhibiting a retrograde flow pattern. This change led to an increase in the false lumen rate during the acute phase. As a result, the flow from the true lumen to the false lumen at the previous re-entry sites gradually decreased. This process ultimately led to aortic remodeling, which was characterized by a reduction in the size of the false lumen.

In the study conducted by Jarvis et al. [162], 20 chronic descending dissection patients were examined, along with 21 age-matched controls. Of the patients, six were medically managed, while 14 had undergone repair procedures. Hemodynamics were assessed in these individuals, and repaired dissection patients had received either an ascending aortic repair or an elephant trunk stage one procedure. The results showed that patients with repaired type A dissection had higher levels of retrograde flow and increased kinetic energy in the true lumen compared with the control group. They also exhibited higher true lumen kinetic energy than patients with type B dissection. Additionally, repaired type A dissection patients were associated with higher false lumen kinetic energy and lower false lumen stasis compared with type B dissection patients. Imaging demonstrated both global and regional hemodynamic differences between patients with descending aortic dissection and the control group. Repaired type A and type B dissection patients exhibited significantly changed regional true and false lumina hemodynamics. A patient with ascending aortic repair showed elevated antegrade flow, retrograde flow, and kinetic energy in the true lumen when compared with a patient with type B dissection and a control subject. On the other hand, the type B dissection patient had elevated levels of stasis. This information suggests that patients with repaired type A dissection may encounter complex flow situations that could potentially lead to late complications. One possible explanation for the difference in kinetic energy and retrograde flow after ascending aortic repair could be the use of a noncompliant graft replacing the ascending aorta, although this hypothesis is still speculative. Overall, they proposed a data-driven classification of aortic dissection patients for risk stratification, which also takes hemodynamic parameters into account.

Then, Evangelista et al. [320] presented a prospective study of 131 consecutive patients, from which 78 were surgically treated type A dissections and 53 were medically treated type B dissections. In the acute phase, these patients initially showed a persistent patent false lumen in the descending aorta. They reported a false lumen systolic antegrade flow of ≥30% relative to total systolic antegrade flow in combination with retrograde diastolic flow of ≥80% relative to total diastolic false lumen flow, which were identified as predictors of a higher risk of complications.

Cosset et al. [511] investigated the influence of TEVAR on blood flow in the aorta and its branches in patients with type B dissection using 4D-flow MRI. Seven patients scheduled for TEVAR underwent 4D-flow MRI before and after the procedure. The results showed that TEVAR significantly increased the antegrade flow in the true lumen from 59.9 to 81.6% and significantly decreased both antegrade flow (from 15.9 to 3.3%) and retrograde flow (from 10.3 to 4.6%) in the false lumen. Interestingly, the retrograde flow in the true lumen increased from 4.36 to 10.8% after TEVAR, which may be due to the increased wall rigidity caused by the metallic stent. Additionally, user-independent helicity quantification revealed elevated helicity at the level of the secondary entry tears, which had been missed by streamline visualization.

In earlier discussions, we emphasized the critical role of aortic or false lumen dilatation in dissection as a significant risk factor (Section 3.1.2). The surgical treatment of a progressively dilating false lumen is vital to reduce the risk of rupture. Numerous studies have investigated the relationship between dilatation during the chronic phase and hemodynamics, underscoring the importance of understanding these dynamics in managing aortic dissection.

In two subsequent studies, Inoue et al. [512] investigated the relationship between blood flow characteristics and aortic dilatation. They examined six patients with chronic type B dissection and an existing intimal tear, which allowed communication between the two lumina. In five of these patients, they observed bidirectional flow in the false lumen. They provided a more detailed analysis of two patients’ results. One patient showed a peak average velocity and flow rate in the true lumen of 700 and 100ml/s, respectively. In contrast, the flow rate in the false lumen was extremely low, with retrograde flow noted during diastole. During systole, the output volume in the true and false lumina was 34 and 4ml, respectively, resulting in a ratio of 0.12. Another patient exhibited a dilated false lumen with a flow rate exceeding that of the true lumen during systole. The authors hypothesized that the dilated false lumen compressed the true lumen, impeding its circulation. In this instance, the ratio between the output volume of the true and false lumina during systole was 1.68. Moreover, they investigated the relationship between the false lumen diameter and the blood flow ratio in the true and false lumina. They discovered an inverse correlation between the maximum diameter of the false lumen and the peak average velocity of the true lumen. As the maximum diameter of the false lumen increased, the peak average velocity of the true lumen decreased. In a subsequent study, Inoue et al. [513] analyzed 21 patients and discovered that flow patterns in the false lumen could be divided into three categories: primarily antegrade flow, primarily retrograde flow, and bidirectional flow. They found that the flow rate in the false lumen, when compared with the total flow rate in the true and false lumina, was significantly higher in patients with a primary antegrade flow pattern. Additionally, the average yearly growth rate of the maximum diameter of the dissected aorta was also higher in these patients. There was a notable correlation between the flow rate in the false lumen relative to the total flow rate in the true and false lumina, and the average yearly growth rate of the maximum diameter of the dissected aorta. The blood flow velocities in the true and false lumina, as well as the direction of the flow were highly dependent on the location and size of the dominant intimal tears.

**Fig. 28.**
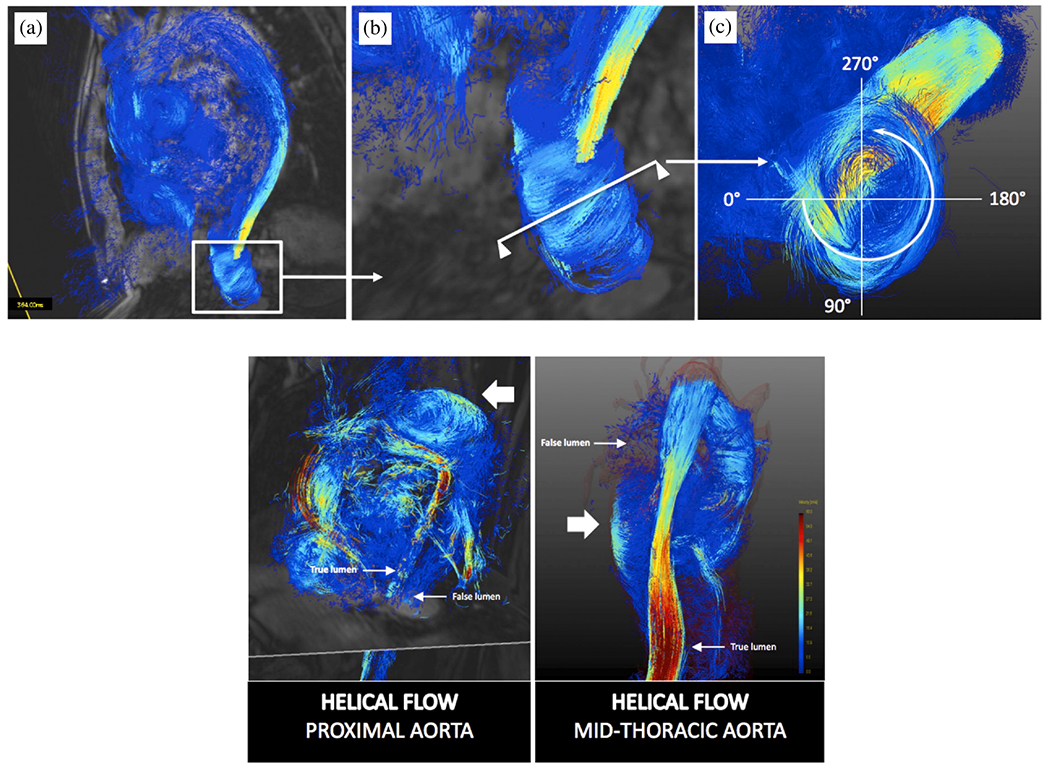
Visualization and quantification of helical flow in a patient with type B dissection assessed with 4D PC-MRI: (a) entire aorta examined to identify regions exhibiting helical flow, (b) plane placed perpendicular to the primary flow direction, and (c) helicity (in degrees per second) calculated by evaluating the amount of in-plane rotation and the start and end times of the cardiac cycle. Figure is reprinted from Clough et al. [158] with permission from Elsevier.

In a later study, Clough et al. [158] aimed to visualize and quantify flow characteristics in patients with type B dissection, exploring their relationship to the rate of aortic dilatation. They discovered that an increased false lumen stroke volume and the velocity were associated with faster aortic dilatation. Helical flow, as demonstrated in Fig. 28, was observed in the false lumen in eight out of 12 patients and was also linked to the rate of aortic dilatation. In five patients, helical flow occurred at a single location, while it was present at two locations in one patient and throughout the false lumen in two patients. The helical flow typically developed in early systole before disappearing in early diastole. Furthermore, the rate of rotation of the helical flow was found to correlate with the rate of aortic dilatation.

**Fig. 29.**
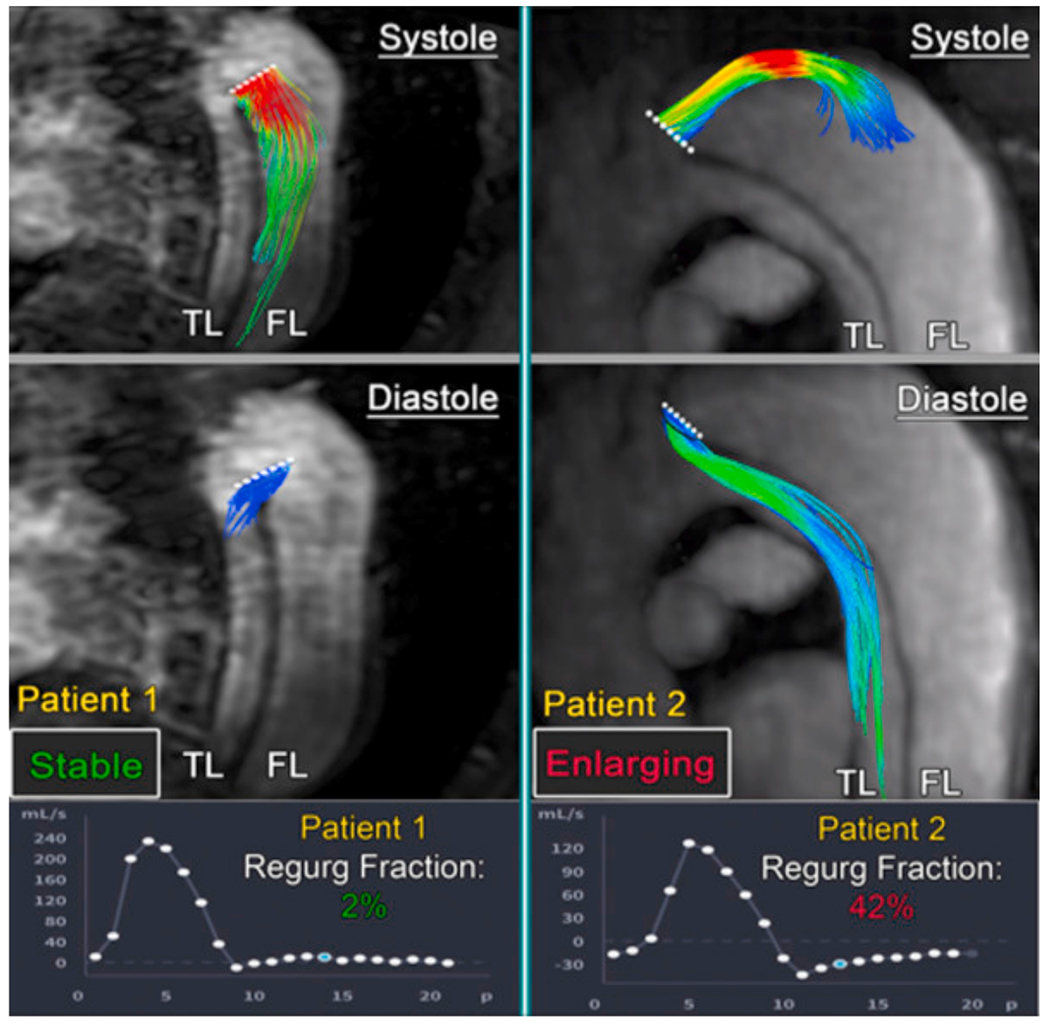
3D PC-MRI results of two representative patients with a history of a stable and dilating false lumen (‘FL’) (‘TL’: true lumen). Figure is reprinted from Burris et al. [514] with permission from Elsevier.

In a series of studies, Burris and his colleagues investigated the role of hemodynamic stress as a primary factor in the progressive dilatation of the false lumen in chronic type B dissection. In their first study [514], the authors examined 12 patients and determined and evaluated the entry tear regurgitant fraction, defined by the ratio of retrograde flow rate at the dominant entry tear during diastole divided by the antegrade systolic flow rate. For their assessment, they first located the primary entry tear, placed a 2D flow analysis plane into the 3D dataset of the 4D-flow MRI, and then calculated the antegrade and retrograde flow. They found that entry tear regurgitant fraction was significantly higher in patients with a history of false lumen dilatation, which was likely due to inadequate distal outflow pathways, see Fig. 29. As previously mentioned, these results support the 2007 statement by the IRAD, which suggests that reduced outflow in partial false lumen thrombosis leads to increased false lumen pressures, i.e. to increased hemodynamic stress in the false lumen [515]. However, the association between elevated regurgitant flow and false lumen dilatation is likely not just a result of aneurysmal degeneration. Instead, retrograde flow is more likely a direct consequence of decreased false lumen outflow, as shown by computerized blood flow simulations on nonaneurysmal models [270,516]. False luminal outflow tells only half of the hemodynamic story and its direct measurement is almost impossible because of the often numerous and small outflow pathways. Therefore, alternatively, direct measurement of the entry tear regurgitant faction using 4D-flow MRI is an intuitive and simplified approach that aims to quantify the false lumen hemodynamic stress that occurs as a result of inadequate outflow pathways in the false lumen.

In a subsequent study [161], an analysis of 18 patients with chronic type B dissection was performed to further evaluate the ability of various hemodynamic variables to independently predict aortic growth rate during follow-up, with particular emphasis on the false lumen ejection fraction, previously termed entry tear regurgitant fraction. Retrograde flow in the false lumen was particularly common, with 16 patients showing measurable retrograde flow during diastole, while the flow in the true lumen was predominantly antegrade. The mean false lumen ejection fraction (28.9±24.4%) did not correlate well with the false lumen retrograde flow fraction measured 3 cm distal to the primary tear. However, it was significantly higher in patients with increased aortic dimension than in patients with stable dimension (43.8±22.1 versus 10.3±10.1%). In patients with a dilated aorta, the entry tear peak velocity was lower. Key observations include that peak velocity at the entry tear jet is lower in patients with a history of a dilating false lumen, likely due to obstructed or insufficient false lumen outflow. Furthermore, false lumen ejection fraction demonstrates a moderate to strong correlation with aortic growth rate. Finally, baseline maximal diameter and the distance of the entry tear from the left subclavian artery are independent predictors of aortic growth rate. Despite the limitation of investigating a relatively small and specific cohort composed only of chronic dissections and considering the timing of 4D-flow MRI, which raises the possibility that elevated false lumen ejection fraction is a consequence of aortic growth rather than a cause, their findings provide further evidence for the importance of false lumen hemodynamics in the risk assessment of patients with type B dissection.

**Fig. 30.**
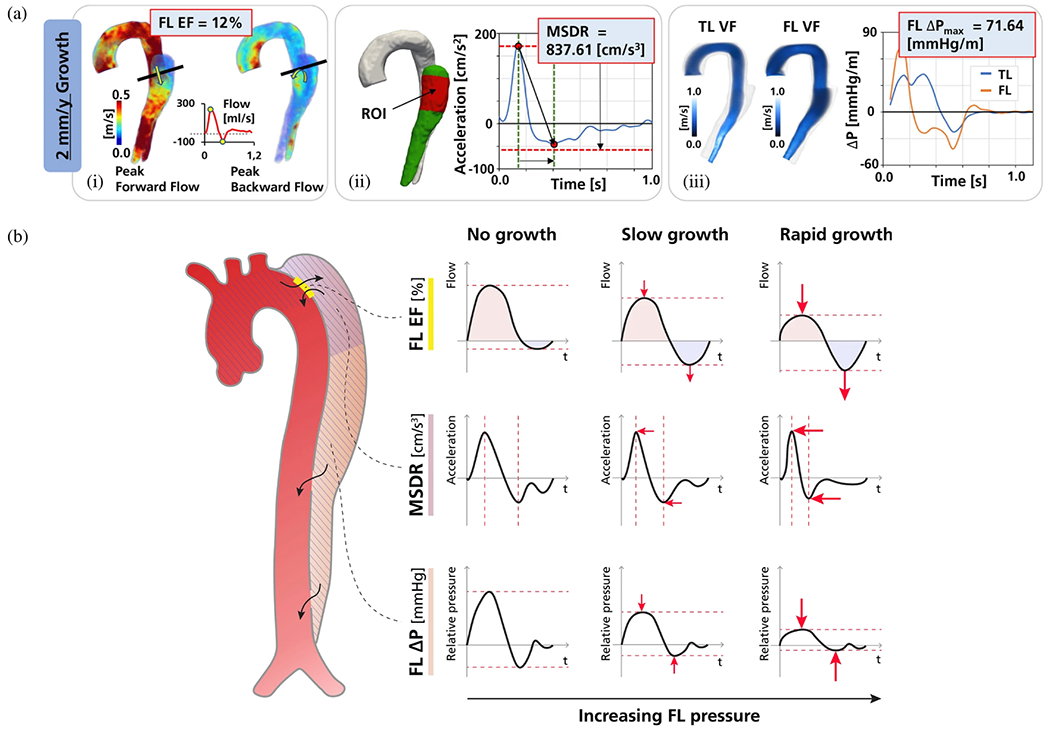
(a) Representative patient examples for false lumen (‘FL’) hemodynamic evaluation in patients with slow aortic growth: (i) extraction of false lumen ejection fraction (‘FLEF’), (ii) maximum systolic deceleration rate (‘MSDR’), and (iii) false lumen maximum relative pressure (‘FL ΔP_max_’) is shown along with the associated output (‘VF’: virtual field). (b) A conceptual model illustrates the relationships between aortic growth and 4D-flow-derived markers of false lumen pressurization and shows that increased false lumen ejection fraction is correlated with increased retrograde flow (light blue), maximum systolic deceleration rate reflects a faster flow deceleration due to higher pressurization, and a false lumen maximal relative pressure indicates a reduced pressure difference with increased aortic growth rate. Figure is adapted from Marlevi et al. [517] (licensed under CC-BY 4.0).

In a third study [517] with 12 patients, they investigated the relationship between aortic growth and three different methods for assessing hemodynamic stress in the false lumen: (i) false lumen ejection fraction, analogousto their previous studies; (ii) maximum systolic deceleration rate; and (iii) false lumen relative pressure. The maximum deceleration rate was calculated as the difference between maximum and minimum acceleration during systole divided by the corresponding time interval between these two points, while the false lumen relative pressure was based on a non-invasive measurement of intravascular pressure drop [518]. The results shown for a patient with slow aortic growth and a conceptual overview depicting the proposed relationship are presented in Fig. 30. These findings emphasize the variable nature of aortic hemodynamics in patients with type B dissection and suggest that these three hemodynamic metrics may be helpful in identifying patients at highest and lowest risk for progressive aortic growth and complications.

In a related study, the aim of Chu et al. [519] was to evaluate the predictive value of the hemodynamics in the true and false lumina using 4D-flow MRI compared to aortic morphology measurements for adverse aorta-related outcomes and aortic growth in patients with type B dissection. The study included 51 patients, including 26 with a type B dissection and 25 with a repaired type A dissection, who had residual type B dissection, all with at clinical follow-up of at least six months. Patients were categorized based on the presence or absence of adverse aorta-related outcomes and an aortic growth rate of ≥3mm/year. Their findings indicated that patients with *de novo* type B dissection and adverse aorta-related outcomes had larger baseline diameters, lower false lumen stasis, and lower true lumen peak velocity. Additionally, a higher kinetic energy ratio was observed in patients with aortic growth of ≥3mm/year across both the overall cohort and the *de novo* type B dissection subgroup.

**Fig. 31.**
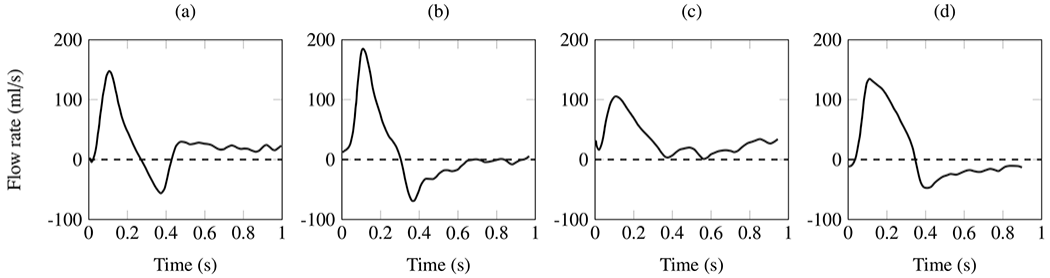
Four representative false lumen flow patterns observed in aortic dissection patients at the diaphragmatic level. These patterns are classified based on the directionality of flow during systole and diastole, with positive values representing antegrade flow and negative values denoting retrograde flow: (a) biphasic systolic flow with predominantly antegrade diastolic flow, (b) biphasic systolic flow with predominantly retrograde diastolic flow, (c) monophasic systolic flow with predominantly antegrade diastolic flow, and (d) monophasic systolic flow with predominantly retrograde diastolic flow. Figure is adapted from Rudenick et al. [516] (licensed under CC-BY 4.0).

A combined clinical and computational study was developed by Rudenick et al. [516] to investigate the aortic wall stiffness, the size and location of the tear, and the presence of abdominal side branches arising from the false lumen. These factors, among others, influence the dilatation of the false lumen, and are often associated with chronic aortic dissection. For this purpose, they monitored the false lumen flow pattern of 33 patients with chronic descending aortic dissection from three to 12months after the acute phase in a cross-section by PC-MRI. After quantifying the measurements, four characteristic false lumen flow profiles, as shown in Fig. 31, were identified in 94% of all cases: (i) systolic biphasic flow and primarily diastole antegrade flow (18%), (ii) systolic biphasic flow and primarily diastole retrograde flow (42%), (iii) systolic monophasic and primarily diastole antegrade flow (27%), and (iv) systolic monophasic flow and primarily diastole retrograde flow (6%). Results from the *in silico* model are presented in Section 6.

The study by Ruiz-Muñoz et al. [520] aimed to understand the relationship between false lumen anatomy (diameter, entry tear location, and size), fluid dynamics (inflow, rotational flow, WSS, kinetic energy, and flow acceleration and stasis), and biomechanics (pulse wave velocity) with the presence and extent of a thrombus in the false lumen. The study included 68 patients with chronic non-thrombosed or partially thrombosed false lumen in the descending aorta after aortic dissection using CTA, cardiovascular MRA, and 4D-flow MRI. The study found that aortic growth rates in patients were higher with partial thrombosis than with a patent false lumen. False lumen kinetic energy was the primary indicator for distinguishing between patients with a patent false lumen and partial thrombosis and was independently associated with the extent of the thrombus.

Numerous studies have examined the hemodynamics of aortic dissection, focusing on kinetic flow patterns, flow rates, flow velocity, helicity, kinetic energy, and pressure in patients with varying disease manifestations and outcomes. Some studies also highlight local phenomena and associated hemodynamic changes, such as flow acceleration due to narrowing in the true lumen or the flow jet impacting on the outer wall of the false lumen, which is primarily influenced by the location and size of the intimal tear. More specifically, the flow patterns in the true and false lumina differ significantly during both the systolic and diastolic phases of the cardiac cycle. During systole, the laminar flow is predominantly antegrade in both true and false lumina. However, the maximum flow rate in the false lumen is slightly delayed and often considerably lower. In diastole, a primary retrograde flow in the false lumen is typically observed, leading to a bidirectional flow in the lumina throughout the cardiac cycle. Several studies by Burris and his colleagues also aimed to find a correlation between hemodynamic markers, such as the introduced false lumen ejection fraction, and late adverse events like aortic dilatation, yielding promising results. These findings are particularly appealing due to the simplicity of the markers used. In conclusion, hemodynamic studies repeatedly confirmed that blood flow between the lumina is consistently determined by the location, number, and size of intimal tears, which subsequently affect the flow pattern. However, the detailed hemodynamic conditions are always patient-specific.

Moreover, the distribution and role of WSS in aortic dissection *in vivo* have not been extensively studied using techniques such as 4D-flow MRI, which may be due to the challenging assessment requiring high spatial resolution. However, similar studies have been performed for other diseases. As an example, Guzzardi et al. [422] observed in bicuspid aortic valve patients that regions with higher WSS experienced greater elastin degradation, which is associated with aortic wall remodeling, compared to adjacent regions with normal WSS. For a more extensive discussion, interested readers are referred to Section 9.2. Similar imaging techniques could potentially be used to examine aortic dissection and provide further detailed insights. However, due to errors related to the low spatial resolution of 4D-flow MRI measurement, the results should generally be interpreted with caution. In conclusion, despite many important findings that have improved our understanding of aortic dissection, the available data are too sparse to generalize many of the findings due to the complexity of the disease and the individuality of the patients. To improve statistical significance, larger patient cohorts are required. Table 4 provides an overview of the presented studies on hemodynamic changes in aortic dissections, along with additional studies that have not been previously mentioned.

### 3.4. Role of thrombus formation

Aortic dissection can lead to the formation of false lumen thrombosis [126,533]. A thrombus usually forms due to slow or stagnant blood flow, endothelial damage, inflammation in the aortic wall, or changes in the hemodynamics, such as favorable WSSs and pressures [534]. These factors are influenced by pathological changes in the aortic structure. However, the specific factors that contribute to or prevent thrombus formation in aortic dissection are not fully understood. Understanding these mechanisms is crucial for developing effective treatments and surgical interventions, as the level of thrombus formation is tightly linked to the mortality rate. Thrombus formation within the false lumen in chronic aortic dissection might lead to several complications, including false lumen dilatation, occlusion of aortic branches, or embolization.

#### Thrombosis.

Blood primarily consists of plasma, its liquid component, and formed elements, which include red blood cells, white blood cells, and platelets [535]. Platelets, also known as thrombocytes, play a critical role in hemostasis, which is the body’s natural mechanism to stop bleeding and promote wound healing through the formation of blood clots [536]. The clots are the result of the coagulation cascade and consist mainly of platelets and red and white blood cells in a fibrin network. Thrombin, an enzyme that catalyzes the conversion of fibrinogen to fibrin and activates platelets and other coagulation factors, plays a key role in coagulation. In addition to its role in coagulation, thrombin is involved in the activation of platelets and other coagulation factors, and functions as an antithrombotic agent by regulating the coagulation process through the production of inhibitors.

The formation of blood clots is a complicated process that involves a combination of biochemical mechanisms, as well as transport mechanisms [535]. During hemostasis, platelets are sensitized and activated, causing them to adhere to injured blood vessels, form aggregates, and release additional agonists. This leads to a feedback mechanism that promotes further platelet activation. The coagulation cascade, which is the final step in hemostasis, involves intricate biochemical processes that ultimately lead to the formation of blood clots. This complex mechanism can be divided into three major phases: platelet adhesion and activation, aggregation, and coagulation cascade.

Initially, when the body experiences an injury, platelets, which can exist in both resting and active states, undergo a conformational change and become activated [537], adopting a rounded shape that enables them to adhere to damaged tissue and form a platelet plug. Platelets become activated when they encounter injured areas of the vascular wall, as specific glycoprotein receptors interact with components of the ECM, such as von Willebrand factor or collagen. This interaction initiates the process of platelet activation. A feedback mechanism then stimulates the release of other chemicals, such as adenosine diphosphate, thromboxane *A*_2_, and thrombin, promoting further platelet activation. Shear stress is considered an important factor in thrombus formation because it can impact the function and phenotype of endothelial cells, which can influence platelet activation. Further information regarding the impact of shear stress on platelet activation can be found in relevant literature [538,539].

In the second phase, aggregation is triggered by the release of chemical agonists, resulting in platelet-to-platelet adhesion and activation of receptors. This process leads to the formation of the primary platelet plug, also known as primary hemostasis, which temporarily seals the injured area and reduces minor bleeding. Finally, in the third phase, known as the coagulation process or secondary hemostasis, fibrin is produced, which cross-links with the platelet plug to form stable clots. This mechanism is regulated by coagulation factors and cofactors, along with calcium and platelets. The coagulation process consists of three pathways: extrinsic, intrinsic, and common pathways, which are described in more detail in relevant literature [536].

**Fig. 32.**
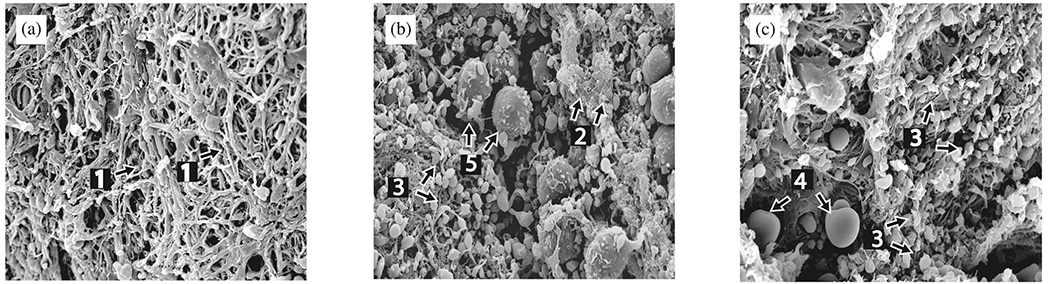
Representative scanning electron microscope images of coronary arterial thrombi: (a) arterial thrombus consisting mainly of fibrin fiber bundles (1) and fibrin sponge (2), and (b, c) dense, contracted thrombi with platelet aggregates (3) surrounded by fibrin and red blood cell balloons (4) trapped within the fibrin mesh; white blood cells (5) are also present. Figure is reprinted from Chernysh et al. [540] (licensed under CC-BY 4.0).

Yet there are remarkably few detailed analyses of thrombus structure that enhance our understanding of its relationship to vascular origin and duration *in vivo*, as well as the composition or internal structural features of thrombi most closely associated with embolization risk [540]. In other words, the composition, physical properties, and evolution are likely to differ based on factors such as their location (e.g., arterial and venous thrombi or pulmonary embolism), local conditions, and the time since formation. As they age, thrombi form a dense, stiff fibrin network that becomes stiffer over time [541]. For example, Fig. 32 illustrates the microstructure of coronary artery thrombi, which primarily consist of fibrin and platelets. The dense fibrin structure appeared inhomogeneous, with prominent thick bundles and a highly branched ‘fibrin sponge’. Most red blood cells were found in compressed forms, with smaller amounts of normal biconcave and balloon-like red blood cells. Cellular microvesicles and leukocytes were also present in the thrombi.

As previously mentioned, several factors may contribute to the formation of thrombi in patients with aortic dissection. Local pathological changes in morphology can cause hemodynamic alterations that result in complex and convoluted geometry and result in areas of stasis, turbulence, or local flow disturbances, which collectively increase the risk of thrombus formation [542–544].

Turbulent flows enhance mass transfer and local mixing of coagulation factors, influence endothelial cell function, and bring platelets into close proximity to vessel walls [545]. In contrast, areas of recirculation and stasis are associated with low shear rates (below 100s^−1^) and long residence times – the time a platelet remains in contact with a wall. These conditions promote deposition through the action of fibrinogen, increasing local blood viscosity and thereby enhancing the adhesion of platelets to the vessel walls [534,546]. This effect is further amplified by the tendency of cells to accumulate in recirculation areas due to transport mechanisms that move them from regions of high shear rates to regions of low shear rates.

In addition to shear rates, platelet activation can also be triggered by shear stress [543], as both can act as physical agonists to promote platelet activation [544] and affect endothelial function, which leads to hemolysis of red blood cells and the resulting release of adenosine diphosphate [547]. While lower shear stresses prolong the interaction time between platelets and coagulation factors, high shear stresses affect platelet activation and adhesion. Therefore, the effect of shear stress on platelet activation largely depends on the duration of exposure, i.e. the residence time, with longer exposure times being required for low shear stress levels and *vice versa* [548]. Interestingly, exposure to pulsatile shear stress can elicit a much stronger response and a significantly higher level of platelet activation, highlighting the importance of shear gradients in thrombosis [549–552]. Finally, platelet activation can be triggered by the tearing of the aortic wall when blood cells come into contact with different and potentially thrombogenic surfaces between the first two wall layers, analogous to an injury response.

#### Correlation between thrombosis, dilatation, and mortality rates.

**Fig. 33.**
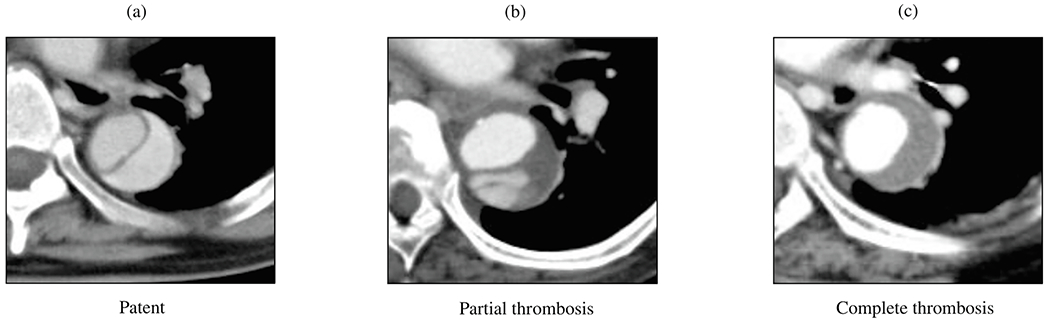
Representative enhanced CT images showing the status of the false lumen in aortic dissection: (a) patent, (b) partial thrombosis, and (c) complete thrombosis. Figure is adapted from Tanaka et al. [553] with permission from Elsevier.

The progression of false luminal thrombi has been found to have a major impact on patient survival and therapeutic outcomes. Several studies have investigated the correlation between the status of false luminal thrombus and long-term outcome in patients with aortic dissection. These studies typically involve patients with type B dissection, type A dissection following surgical repair, or a combination thereof. Study cohorts are often categorized according to false lumen patency, distinguishing between patent, partially thrombosed, or completely thrombosed false lumina, as shown in Fig. 33. However, some studies did not distinguish between the latter two categories, making an interpretation of the results difficult. According to the definition of Tsai et al. [515], a partially thrombosed false lumen is defined as the concurrent presence of both blood flow and thrombus. Interestingly, the outcome of these studies can be contradictory at times. Additionally, the terminology used to describe the false lumen status is not consistent in the literature, with some studies referring to incomplete or complete thrombus formation [4] or other terms. We have adapted the most commonly used and unambiguous terminology of the false lumen status.

Tsai et al. [515] determined that the risk of death is 2.7 times higher for partially thrombosed false lumina compared with patent false lumina. However, for completely thrombosed false lumina, the risk is only 1.9 times greater. Following their study, they found that the mortality rates after three years were 13.7±7.1, 31.6±12.4, and 22.6±22.6% for patent, partially thrombosed, and completely thrombosed false lumina, respectively. They also linked the occurrence of partially thrombosed false lumen to an increased risk of false lumen dilatation caused by diminished outflow and thus increased mean pressure in the false lumen, which is consistent with other studies [554–556]. Particularly, areas featuring new or increased partial false luminal thrombi are strongly associated with late adverse outcome, following Higashigaito et al. [556]. In Trimarchi et al. [554], mortality rates of patent (37.5%), partially thrombosed (56.3%), and completely thrombosed (6.3%) false lumina were observed. They stated that a thrombus might lead to a diameter stabilization, but also to a higher incidence of malperfusion, i.e. the loss of blood supply to vital organs. Consequently, these studies suggested that a partially thrombosed false lumen is an independent risk factor for surgical re-intervention and overall survival rates. It is noteworthy, however, that in contrast to Tsai et al. [515], Trimarchi et al. [554] reported that a persistent patent false lumen carries the highest risk of complications, which is consistent with most other studies.

By contrast, the remaining studies did not associate partial thrombosis of the false lumen with worst late outcomes, such as a higher mortality rate or faster aortic growth, compared with a patent false lumen. Although some studies did not observe a correlation between the mortality rate and the thrombosis status [553,557,558], most studies have reported that a completely thrombosed false lumen is beneficial for late outcomes, whereas a patent false lumen increases the mortality rate [264,308,327,329,559–564]. In the study by Fattori et al. [562], for instance, it was found that the ten-year survival rate for patients with a patent false lumen was 58.8±3.5%, whereas for those with a completely thrombosed false lumen, the rate was significantly higher at 89.8±2.1%. Note however, they did not distinguish between a partially and completely thrombosed false lumen. These findings were also confirmed by De Leon et al. [564].

**Fig. 34.**
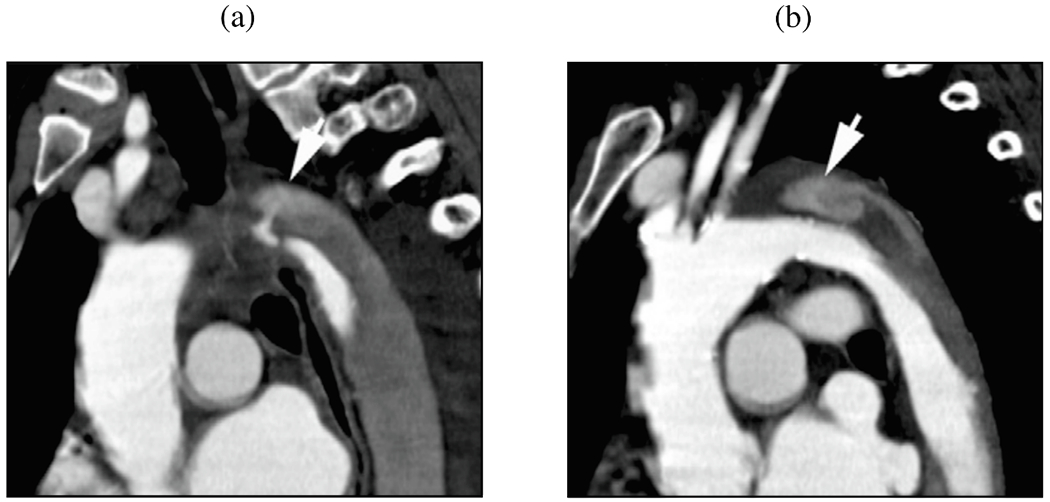
Two phases of imaging after contrast injection, (a) and (b), of enhanced CT images showing a sac-like formation of partially thrombosed false lumen, defined as a blind pouch with a persistent entry tear (arrows). Figure is reproduced from Sueyoshi et al. [327] with permission from Elsevier.

Other studies have reported that a partially thrombosed false lumen predicted a higher operation rate but does not affect long-time survival rates [558]. Tanaka et al. [553] found that the probability of requiring surgical treatment was 0% for completely thrombosed, 16% for partially thrombosed, and 26% for patent false lumina, with no observed differences in the long-term mortality. Late mortality was instead associated with patient age (>70 years old) and cerebrovascular accidents, coma, or renal failure [557]. Similarly, Bernard et al. [560] found that in patients older than 70 years, the presence of a patent false lumen after surgery increased the relative late mortality risk by a factor of 3.4. While the mortality rate tended to increase with an age of 70 years, patients with a patent false lumen were usually younger. Mean ages per group as reported by Tanaka et al. [553] were: 58.3±16 years old for patent, 72.4±10.3 years old for partially thrombosed, and 68.4±11.1 years old for completely thrombosed false lumen. Larsen et al. [557] reported that patients with a patent false lumen were on average three years younger than those with a partially thrombosed and six years younger than those with complete false lumen thrombosis. These findings suggest that the probability of thrombus formation tends to increase with age, which could be related to the increased likelihood of blood coagulation with age. The increased late mortality associated with partially thrombosed false lumina might be explained by the possible occurrence of local hypoxia in the aortic wall adjacent to a formed thrombus [515]. Similar to aortic aneurysms, hypoxia could increase local inflammations, neovascularization, and localized wall weakening [565,566], thus making the aortic wall more prone to fail. To explain why partially thrombosed false lumina are often associated with an increased late mortality, Sueyoshi et al. [327] subdivided partially thrombosed false lumina into a sac formation type and a non-sac formation type. Of the patients with a partially thrombosed false lumen, 15% showed a sac formation and 85% did not. The sac formation type was defined in this study as a partially closed false lumen at the distal site of the false lumen, forming a blind pouch with a persistent intimal tear, as shown in Fig. 34. A sac formation was also associated with higher pressures in the false lumen [515], which was in turn associated with a worse outcome. Due to the low incidence of 15%, these results did not support the hypothesis that the formation of a partial thrombus *per se* leads to an increased risk of death. In summary, it might not be correct to state that partially thrombosed false lumina increase the mortality rate of patients. Specifically, some authors classify cases with a marginal amount of thrombus in the patent group, while others categorize them into the partially thrombosed cohort. This discrepancy does not allow formulating a clear statement.

Although some findings have been controversial, a patent false lumen appears to be associated with high risks, while complete false lumen thrombosis has been linked to improved outcomes for patients. According to [567], surgical intervention can result in complete false lumen thrombosis in up to 91.3% of patients, along with morphological evidence of remodeling over time. Therefore, it is essential to understand the conditions that enable complete false lumen thrombosis. In addition, studies suggested that reabsorption of the area occupied by the false lumen contributes to patient recovery after complete thrombosis [308,560]. Although early case reports on self-healing of dissection did not specifically refer to complete false lumen thrombosis [271,568], it is reasonable to assume that thrombus formation could potentially stop aortic dissection or even facilitate healing in some patients.

As previously mentioned, the occurrence of late adverse events is associated with aortic dilatation, which, according to various studies, can also be linked to the status of the false lumen. While some studies focused solely on false lumen dilatation, this condition typically occurs alongside overall aortic dilatation, and the two are often interconnected and inseparable. In particular, the absence of a thrombus in the false lumen is often associated with dilatation [313,315,316,326,327,554,560,562]. In other words, a patent false lumen can accelerate growth of the aorta whereas a completely thrombosed false lumen can potentially stop it. Thus, a formed thrombus might act to stabilize the diameter of the aortic or the false lumen. For example, Fattouch et al. [562] reported the lowest growth rates for a completely thrombosed false lumen. Based on their clinical data, a patent false lumen grows at 2.8±0.4mm/year and a completely thrombosed false lumen at 1.1±0.2mm/year in patients with and without Marfan sydrome. Another related study was published by Sueyoshi et al. [326] assessing 62 patients. Over a follow-up period of ~ 40.1months, they observed growth rates in five aortic segments: the arch, the suprarenal abdominal aorta, the infrarenal abdomonial aorta, and the iliac arteries. The mean growth rates were 3.3±4.2 and −1.4±8.6mm for a patent or partially thrombosed and a complete thrombosed false lumen, respectively. Another interesting observation pertains to the difference in the mean growth rate between the thoracic and the abdominal aorta, which are 4.1 and 1.2mm, respectively. Overall, the fastest growth rates were found in the descending aorta. After Tsai et al. [515] identified partial false lumen thrombosis as a predictor of mortality in 2007, this variable was further investigated, also with respect to dilatation. Trimarchi et al. [308] reported growth rates in 84 patients of 2.10±5.56, 4.25±10.18, and 1.51±5.65mm for a patent, partially thrombosed, and completely thrombosed false lumen, respectively. Hence, aortic growth rates were highest in those segments having a false lumen with partial thrombosis. Song et al. [555] made similar observations in the ascending aorta, the arch, and the descending aorta. In contrast, Sueyoshi et al. [327] reported that partial thrombosis was generally not a significant risk factor for false luminal growth. They reported that the highest growth rates were 4.9±4.5, 4.0±4.3, and −0.2±0.6mm, for a patent, a partially thrombosed, and a completely thrombosed false lumen, respectively. Different results were obtained when classifying cases with partial thrombosis into saccular and non-saccular formations. The sac-type formation had annual growth rates of 12.7±1.1mm, while the non-saccular type achieved 2.6±2.7mm. It is also worth noting that inconsistencies were present in terms of definitions and inclusion criteria. Some studies included intramural hematoma and others did not make a distinction between a completely thrombosed false lumen and an intramural hematoma. Hence, the results are not completely consistent, as discussed before. Albeit in the minority, some studies did not associate the status of the false lumen with its growth [569].

## 4. Experimental *in vivo*, *ex vivo*, and *in vitro* models

Early experiments on aortic dissection used animal, human, and phantom models to study disease onset and progression, micro-structural changes in the aortic wall, and to evaluate the safety and efficacy of medical devices such as endovascular implants. At that time there were few or no computational models, so the results of experimental models were crucial and the only viable option. However, with the rise of *in silico* models over recent decades, experimental models have taken on an additional role in validating these models. This development helps to investigate various pathological phenomena without relying on often costly experimental models. Moreover, animal models can help in understanding the microstructural and mechanical changes in the newly formed layers from the acute to the chronic phase. Specifically, creating an artificial dissection in porcine aortas can provide valuable insights into the microstructure of the dissected aorta [455]. For this purpose, mock circulation loops play a crucial role [570], as they enable the controlled evaluation of animal, human, or phantom models with artificial dissections.

This section offers a overview of *in vivo*, *ex vivo*, and *in vitro* experiments on animal, human, and phantom models. *In vivo* experiments are carried out on a living organism of a human or animal, while *ex vivo* experiments test tissue samples taken outside the body in a more artificial environment, such as a mock loop. Note that in the presented *in vivo* models, the aortic dissection was artificially created or modified, typically through surgical intervention. *In vitro* experiments, on the other hand, take place in an artificial environment with phantom models, some of which may be 3D printed.

### 4.1. Replicating aortic dissection to evaluate implant deployment

**Fig. 35.**
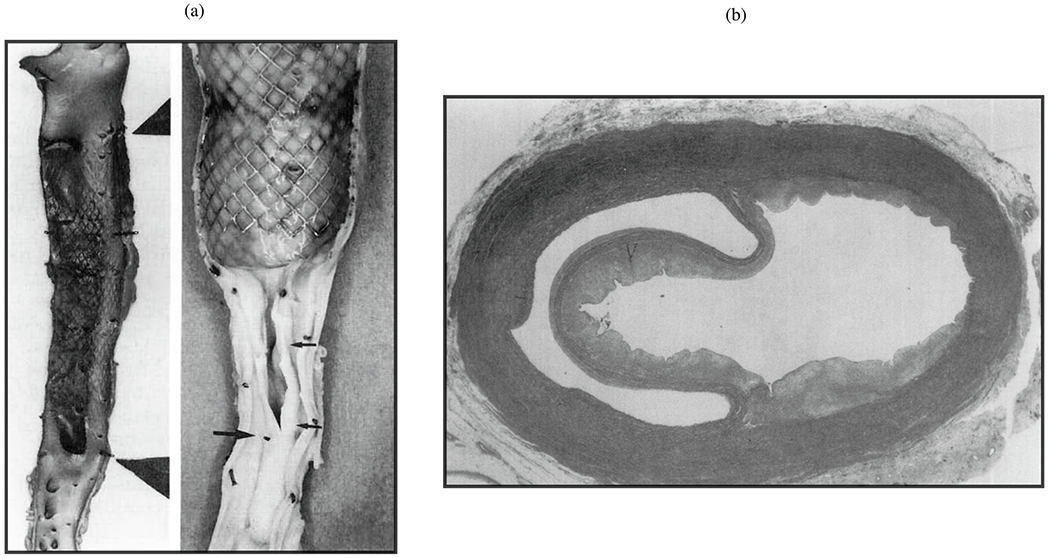
(a) Photograph showing destruction of the dissection after placement of stents along the entire length of the dissection, with a false lumen still visible in the aorta segment beneath the stent. (b) The true lumen appears histologically normal but is compressed, with neointimal proliferation present around the entire false lumen. Figure (a) is adapted from Marty-Ane et al. [586], while (b) is from Moon et al. [583], with permission from Elsevier, respectively.

Quite early on, a number of *in vivo* experimental models were developed to reproduce aortic dissection with the aim of evaluating potential treatments and management strategies. In 1959, one of the first models in this regard was published by Blanton et al. [571]. The aortas of dogs were slit circumferentially to create a cleavage plane within the media and then sutured at the interfaces to maintain the opening, or tear, in a funnel-like position. Of the 26 dogs, 13 developed a dissection extending distally from the intimal opening, while the proximal segment remained collapsed. The time required for dissection was between two and three minutes. Other studies used similar techniques to create artificial dissections [572–579], which were then used to test the effectiveness of the fenestration procedures [580,581] and the efficacy of the use of vascular implant deployment [582–588] or other medical devices [589]. For example, Marty-Ané et al. [586] showed the continued existence of a false lumen in an aortic segment beneath the stent, as shown in Fig. 35(a), while others demonstrated the presence of neointimal proliferation around the inner surface of the false lumen [583], see Fig. 35(b). Another experimental study was published by Moore et al. [590]. They developed an experimental flow loop that included the true and false lumina and tested 24 shear-thinning biomaterials made from blends of gelatin, silicate nanoparticles, and silk fibroin. These biomaterials were able to occlude 99% of blood flow from the true to the false lumen and represent a novel, minimally invasive approach to the treatment of type B dissection by false lumen embolization.

### 4.2. Dissection propagation with a single entry tear

In the past, researchers have created artificial dissections by inserting a needle and injecting a fluid, such as India ink, into the aortic media under constant or pulsating pressure. By increasing the pressure gradually, they obtained volume-pressure curves that provided data on (i) the peak pressure, which is indicative of the strength of the media; (ii) the distensibility of the media defined by the initial linear portion when the pressure rises but the volume changes only very little; and (iii) the pressure drop representing the propagation of the dissection. However, long-term effects could not be investigated because the exposure times were only in the range of minutes. In addition, many others have investigated the propagation of dissection and the pressure necessary to achieve it by constructing models that have only one entry tear.

In the first documented needle experiments done by Halliday and Robertson [591], a constant pressure of 600mmHg was used to induce artificial dissection, but the number of aortas was not enough for meaningful results. Robertson and Viner Smith [592] later conducted consecutive experiments with 42 human aortas, all of which were flat and unstretched. They found a wide range of pressure needed to initiate dissection, the lowest being 230mmHg and the highest 975mmHg. No gender differences were observed, but regional differences were seen with the greatest resistance in the ascending aorta, followed by the aortic arch and the lowest in the descending aorta. Additionally, a weak negative correlation between resistance and age was noticed.

Contrary to that, Hirst and Johns [593] employed the water-injection method, a modification of the technique of Milazzo [594], resulting in a range of outcomes. A total of 63 human aortas were tested with a needle, which was inserted either in the center or outer third of the media, as later ascertained by histology. No distinctions was detected between segments, genders, and races. In terms of age-related effects, the least pressure needed to initiate dissection was between 320 and 520mmHg for aortas from individuals in their first four decades of life, with no significant variations in aortas from older donors. The findings were then validated with two dog aortas with similar results.

**Fig. 36.**
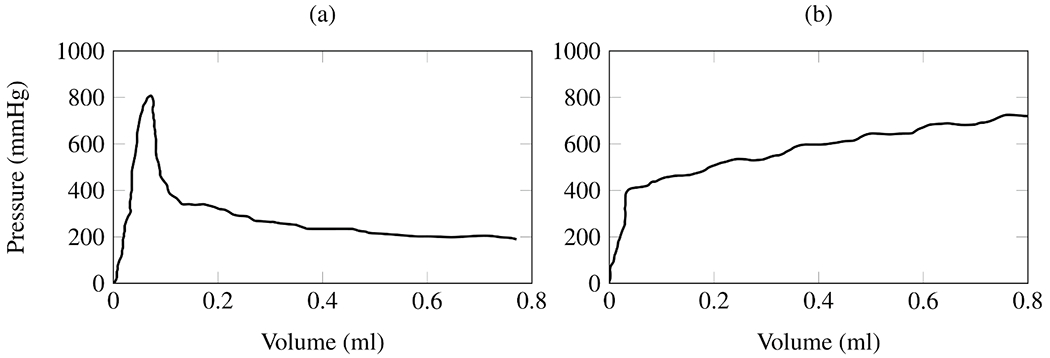
Pressure–volume curves obtained from needle experiments by creating a bleb on thoracic and abdominal aortas, based on experimental data from Roach and Song [596]. (a) In the thoracic aorta, the slope of the first portion of the curve represents the distensibility, while the tearing pressure corresponds to the maximum pressure. The portion to the right of the peak pressure is associated with dissection propagation. (b) In the abdominal aorta, the initial tear occurs at a relatively low pressure, but a higher pressure is required for dissection to occur. The area under the respective curves reflects the work required for dissection.

Some years later, in 1987, Roach and his colleagues conducted three consecutive needle experiments, one on dog and two on porcine aortas. First, van Baardwijk and Roach [432] conducted 19 successful needle experiments on thoracic aortas from dogs. However, an additional 17 attempts were unsuccessful. They inserted a needle into the aorta and an oscillating pressure was applied until the dissection stopped propagating. The circumference length of the tears was measured after the experiment and ranged between 36 and 81% of the circumference, without taking into account the needle penetration depth. The least important factor in the propagation of dissection was the peak pressure that was measured. On the other hand, a larger tear was linked to a slower rate of dissection, and it was found that the rate of dissection was proportional to the rate of change of pressure (d*p*/d*t*) in a significant manner. More specifically, the rate of dissection was determined to be proportional to the square of the rate of change of pressure, i.e. (d*p*/d*t*)^2^. An intriguing observation was that the dissection front always advanced during the upstroke of the pressure wave. Second, Carson and Roach [595] conducted experiments with 34 upper descending thoracic porcine aortas to acquire pressure–volume curves. However, their attempts were not always successful. From the obtained pressure–volume curves, the peak pressure for tearing was estimated to be 579mmHg, the initial slope to be 3.02±0.28MPa^−1^, and the work per unit area of tissue required to tear to be 15.9±0.9mJcm^−2^. The work per unit area was calculated from the area under the pressure–volume curve when the dissection occurred. Notably, there was no correlation between tear depth and peak pressure. In the third study of their series, Roach and Song [596] harvested 17 porcine aortas and applied their experimental protocol to compare results from five locations between the heart and the lower abdominal aorta. Interestingly, the lower abdominal aorta was found to tear at a lower pressure, but required more energy to propagate the dissection. Peak pressures of 634±204 and 816±816mmHg were reported, and the dissection work was determined as 1.88±0.89 and 11.34±4.05mJcm^−2^ in the lower abdominal and lower thoracic aorta, respectively. They also identified two different types of dissection propagation, as shown in Fig. 36, with mixed types existing. The first type, which was found in the thoracic aorta in 50% of the cases and less than 25% in the abdominal aorta, propagated readily once the tissue tore. The second type, however, required a constant increase of pressure to propagate the dissection. This could be attributed to the structural differences between the two aortas, as observed in previous studies using scanning electron microscopy [597–599]. The thoracic aorta had primarily parallel sheets of elastin, appearing attached to each other with thin fibers, whereas the abdominal aorta had a honeycomb structure. Finally, the experiments did not include results based on direction, meaning that differences between circumferential and axial propagation were not examined. Nonetheless, it was suggested that these differences could be similar. In their last study of this type, Roach and Kratky [600] conducted needle experiments on 48 pig specimens. They applied a constant pressure of 130mmHg to bubbles of different depths; however, the tearing and propagation processes were not affected by the depth of the bleb. They reported an average tearing pressure of 547mmHg with a range of 208 to 995mmHg and an average propagation pressure of 54mmHg, which represent the pressures required to initiate the tear and to further propagate the dissection, respectively.

Differently, Prokop et al. [601] developed an experimental approach to explore the impact of pulsating blood pressure on the extension and rupture of dissections. They used two models, a phantom and a dog model of the aorta, to examine the effects of non-pulsating and pulsating blood flow at different pressures. The phantom model consisted of an outer layer of Tygon tubing and an inner layer of rubber cement, which were connected through holes representing intimal tears. When non-pulsating blood flow was applied up to 400mmHg, no dissection was observed in the phantom model. When a pulsating blood flow was used, in contrast, a dissection occurred at a pressure of 120mmHg. Based on the evidence, they determined that the primary sources of the dissecting pressure are the pulsating blood flow and the form of the pulse wave. The results were subsequently validated with descending aortas from 15 dogs. It was proposed that the media surrounding the defect in the aortic wall does not necessarily expand at the same rate or to the same extent as the remaining intact aortic wall. This discrepancy in expansion could create a pressure difference, which then generates a force that causes the dissection. This explanation was supported by the fact that no dissection occurred until the threshold value for the rate of change of pressure (d*p*/d*t*) was reached, here 790mmHg/s. Additionally, they concluded that shear forces acting in the direction of the flow did not have a mechanical impact on the dissection propagation, as a dissection was not observed even when the Reynolds number was high. Furthermore, they noted that shear forces could not explain a dissection propagation opposite to the flow direction.

Then, *in vivo* experiments on 30 dogs with three distinct groups were carried out by Carney et al. [602]. They studied the effect of myocardial contractility, or the rate of change of pressure, i.e. (d*p*/d*t*), and systolic pressure on the development of aortic dissection. The results revealed that a decreased myocardial contractility by itself did not inhibit the advancement of aortic dissection. Moreover, when hypotension was added to a decreased myocardial contractility, they found that aortic dissection was completely blocked.

In another *in vivo* experimental study, Senoo et al. [603] divided 49 dogs into two groups, acute and chronic aortic dissections, and monitored them for one year starting three days after the disease onset to study the predisposing factors and the propagation mechanisms in dissection. To initiate the condition, they used a modified version of Blanton’s method [571], which involved the surgical creation of a transversely oriented entry tear above the right coronary cusp. They found that the length of the dissection was correlated with the pressure amplitude in systole and diastole and, in addition, that the extent of the dissected layer of the pocket was not a factor in dissection progression. The depth of the entry tear was, however, a major factor influencing the propagation of the aortic dissection. Histological investigations of the aortic wall revealed that the aortic wall was fragile in the acute phase but organized itself two weeks after the onset of the aortic dissection, and that ischemic lesions in the medial layer were observed. They speculated that these changes were secondary to the aortic dissection and caused by the disrupted vasa vasorum. Upon realizing that the inner media, located between the true and the false lumen, was nourished by the diffusion of nutrients from the lumina, they noted the effects of a damaged vasa vasorum. Specifically, the central zone was no longer supplied with nutrients, leading to ischemic changes occurring within three days of disease onset. Moreover, they identified a pseudointima (or neointima) on the inner aortic wall of the false lumen two weeks after the onset of the aortic dissection, consisting of a proliferation of collagen and elastic fibers and a loss of SMCs. However, the elastic lamellae were not disrupted, but instead were stretched. Finally, they identified five factors responsible for dissection propagation: blood viscosity, blood pressure, shearing force, turbulence, and the pressure gradient. They concluded that the pressure gradient and the blood pressure were the two most important factors.

Then, several years later, Mitsui et al. [604] conducted a study on aortas isolated from 99 adult mongrel dogs to investigate the relationship between the depth of an intimal tear and the progression of aortic dissection. Using the Blanton method [571], they created an aortic pocket on the aortic wall and connected the aorta in series with a closed circuit at pressures of 150mmHg/100mmHg and a rate of 60 beats per minute. The results showed that the progression of aortic dissection occurred most prominently in the aortic pocket prepared in the first one-third of the external media, with an occurrence rate of 87.5%. In fact, they found no correlation between the pocket width and the progression of the dissection.

In 1998, Tam et al. [605] conducted *in vitro* experiments using 20 porcine thoracic aortas to investigate the influence of entry tear depth on propagation pressure. A saline solution was injected into the medial layer to create a bleb, which was then connected to the true lumen through a circumferential incision. The aorta was pressurized under no-flow conditions until an antegrade or retrograde propagation occurred. Histology was then used to measure the depth of the entry tear, which was between 0.44 and 0.89mm. The results of the experiment showed that the depth of the intimal tear correlated with propagation pressure, with deeper tears associated with lower propagation pressure.

**Fig. 37.**
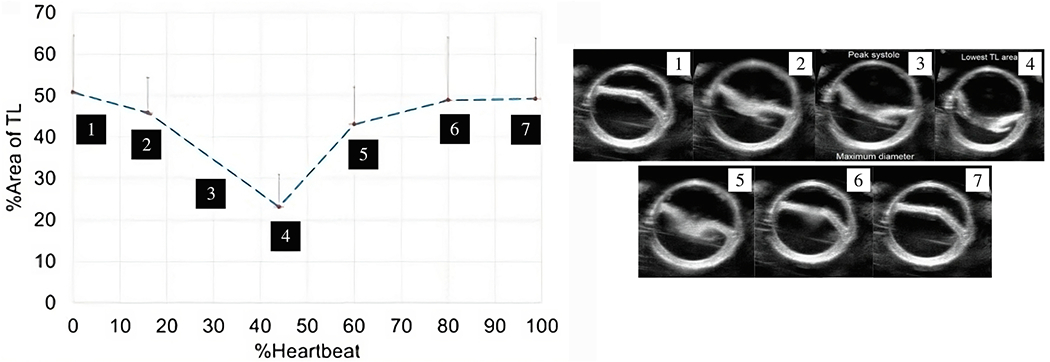
Flap motion at a given mean pressure of 100mmHg with changing pulse pressure over the cardiac cycle. At the beginning of the cycle, the flap was curved toward the false lumen and as the vessel diameter increased, it was pushed toward the true lumen. At peak systole, the vessel diameter was at its maximum and the flap was further pushed toward the true lumen. As the diameter decreased, the flap was pushed toward the true lumen resulting in the lowest point during the cycle. The flap returned to its original position as the cycle progressed. Figure is adapted from Peelukhana et al. [606] (licensed under CC-BY 4.0).

Peelukhana et al. [606] studied how the geometry of entry tears and hemodynamics affected the propagation of a dissection in *ex vivo* models of 36 porcine aortas, each with a single entry tear. The circumference of the tear was varied between 15 and 65% of the aortic circumference and its axial length between 5 and 32mm. The depth of the tear was either one third or two thirds of the wall thickness. A pulse duplicator was used to apply a mean pressure of 100 and 180mmHg and a pulse pressure amplitude ranging from 40 to 200mmHg. The results were summarized into three main findings: first, the length of the dissection was greater for a deep entry tear, but only when the circumference of the tear was >36% and its axial length >12mm. Second, pulse pressure, rather than mean pressure, was the primary factor in flap motion and propagation, and, third, the flap was usually curved toward the true lumen before propagation. Therefore, it can be inferred that the size of the entry tear significantly influences the propagation of dissection. In addition to that, the energy acting on the flap before propagation was calculated by summing the elastic energy stored in the flap, the energy required for the flap and outer wall of the false lumen to move, and the energy needed to break the aortic wall. These findings were consistent with previous studies. As shown in Fig. 37, they also visualized the flap motion at a given mean pressure of 100mmHg with changing pulse pressure over the cardiac cycle.

**Fig. 38.**
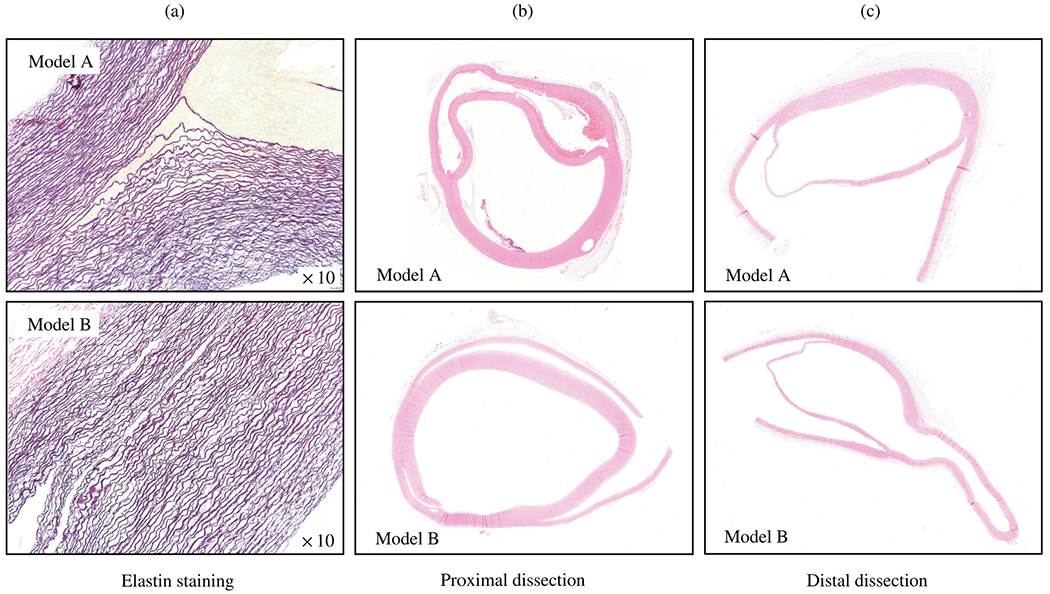
Destruction of the elastic lamellar architecture in the medial layer of the aortic wall was observed through microscopic examination of elastin staining. The elastic fibers on the adventitial side of the false lumen were more stretched than those on the intimal side in both models, with model A having a thin flap and model B having a thick flap. In addition, it was found that the entry tear in model B was deeper in the proximal dissection than in model A. In contrast, in the distal dissection, the exit tears developed superficially, and the flap thickness appeared similar in both models: (a) elastin staining, (b) proximal dissection, and (c) distal dissection. Figure is adapted from Guo et al. [607] with permission from Elsevier.

In a more recent *in vivo* study, Guo et al. [607] evaluated the correlation between dissection propagation and the depth of the entry tear in 12 porcine aortas. For this purpose, they created models with either a thick or a thin dissection flap, see Fig. 38, and found that the axial propagation length was significantly greater in the thick flap model than in the model with a superficial tear. Additionally, 24 months after the onset of the aortic dissection, the antegrade propagation length was significantly longer in the thick flap model. However, the retrograde propagation length did not differ between the two models. Doppler wire measurements revealed that the average peak blood velocity was higher close to the entry tear in the thick flap model. In addition, they observed an exit tear at the ostium of the aortic branch vessels after 24 months, which they suggested was caused by aortic pressure and shear forces. Histology then showed that the collagen fibers in the outer wall of the false lumen had reached their straightened length and formed a stiff, ‘jacket like’ tube that prevented the wall from rupturing.

To investigate the progression of dissection, Chi et al. [608] created two silicone models with varying pocket sizes that included a torn area and a primary tear feature on the inner layer. To assess changes in the diameter and volume of the lumina during the study, CT imaging and laser lightening were applied. Pressurization was made possible by a pipe circulation system that did not have pulsating characteristics. Pressure was increased and CT imaging was carried out alternately until the tear development process was initiated. After that, only CT imaging was conducted. Their findings indicated that the model with larger interlayer adhesion damage required lower pressure to initiate dissection. As part of the investigation, they also attempted to present an analytical framework for the general mechanical behavior of dissection initiation based on the prevailing forces.

**Fig. 39.**
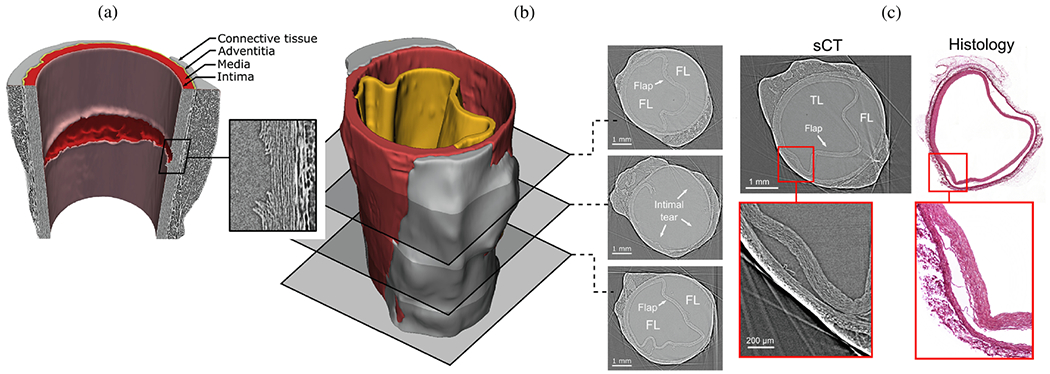
3D segmentation of a specimen showing a circumferential notch post-aortic dissection: (a) 3D perspective of the specimen at 0mmHg, highlighting the initial notch, and (b) 3D depiction of the dissected sample at 0mmHg showing three cross-sections at different positions along the axial axis with the dissected flap colored in yellow. (c) Synchrotron CT (‘sCT’) images (‘TL’: true lumen; ‘FL’: false lumen) and histology images of a dissected descending aorta, imaged after complete dissection at a pressure of 0mmHg with a critical pressure of 251mmHg, show a cross-section of the dissection flap indicated by a white arrow. The histology section, which approximately aligns with the synchrotron CT image locations, exhibits morphological variations due to transportation and histological processes (section thickness: 5μm thick; original magnification 4x, Picro-Sirius red staining). Figure is adapted from Brunet et al. [609] with permission from Elsevier.

In a recent exceptional study by Brunet et al. [609], aortic dissection was artificially induced in *ex vivo* aortic segments from 11 New Zealand white rabbits, after which the propagation was visualized. For this purpose, they combined tension-inflation tests with both conventional and synchrotron CT imaging. This synergy made it possible to capture vivid images of macro- and microstructural changes in the aortic wall during dissection, and achieve real-time visualizations with impressive clarity, as depicted in Figs. 39(a) and (b). Utilizing a custom-made tension-inflation device, samples of aortic segments were placed within a 50mm cylindrical PMMA tube, designed for reduced X-ray interference. An axial pre-stretch of 1.5 was maintained to mimic physiological states. Meanwhile, 3D imaging captured the evolution of the dissection under varying static pressures ranging from 100 to 300mmHg. During imaging, the pressure was maintained constant. Additionally, tears were induced in both circumferential and axial directions. The scans not only provided detailed contrasts of aortic layers but also highlighted the intricate interplay between tear morphology and pressure during dissection. It is noteworthy that the dissections often covered up to three quarters of the aortic circumference. Mean critical pressures were recorded at 595±189 and 685±88mmHg for tears in circumferential and axial directions, respectively. It is worth noting that three samples dissected at pressures below 300mmHg. In most specimens, after the dissection, the external wall of the false lumen failed, leading to vessel rupture. Interestingly, they observed a directional shift of the circumferential tear at 100mmHg, which triggered a dissection between the medial lamellae in the axial direction. This dissection amplified axially at 200mmHg and advanced to the distal segment at 251mmHg. Confirmations of these observations based on synchrotron CT images were supported by histological findings, see Fig. 39(c). The study underscored the potential to quantify tear and aortic morphologies at different stages of aortic dissection and establishes a clear association between tear geometry and critical pressure thresholds.

### 4.3. Effect of intimal tears on hemodynamics and dissection flap

**Fig. 40.**
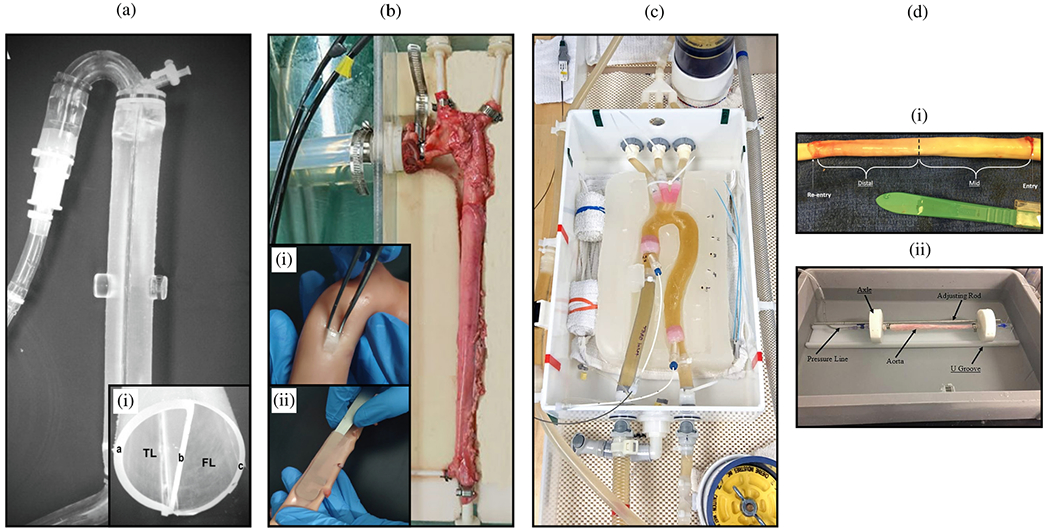
Four representative experimental models, each embedded into a flow circuit to simulate realistic flow and pressure conditions. These conditions are achieved through the use of either a pulsatile pump and control system or a simple pump to apply pressure. The models are as follows: (a) flow circuit featuring an aortic dissection model made of polymeric silicone (‘TL’: true lumen; ‘FL’: false lumen); (b) flow circuit utilizing a porcine model with an aortic dissection, where intimal tears were created using plastic tweezers (i) and scalpels (ii); (c) MRI-compatible flow circuit with a 3D printed aortic dissection model that has varying tear sizes; and (d) inverted porcine aorta with an imposed dissection (i), accompanied by the bench test setup (ii). Figure (a) is adapted from Tsai et al. [610] with permission from Elsevier, while (b) is from Liang et al. [611] with permission from Sage Publications, (c) is from Zimmermann et al. [612] (licensed under CC-BY 4.0), and (d) is from Ahuja et al. [613] (licensed under CC-BY 4.0).

Several research studies have analyzed how the location, size, and depth of intimal tears, mainly with a single entry and exit tear, impact hemodynamics. This includes the pressures and flow velocities within the true and false lumina, and the motion of the dissection flap. These studies utilized animal, human, and phantom models, some of which are illustrated in Fig. 40, to investigate various phenomena. In order to replicate realistic flow and pressure conditions in their respective models, many of these studies developed a mock loop or similar flow circuits.

In 1991, Iwai et al. [614] created experimental phantom models made of styrene–butadiene–styrene rubber, a flexible and compliant material, to analyze the flow patterns in aortic dissection. This study used three phantom models that varied in the location of the intimal tear, which was approximately 5mm in diameter. The models had either a proximal tear, both a proximal and a distal tear, or only a distal tear. To visualize and analyze the blood flow in the lumina, the flap motion, and the wave propagation, cine PC-MRI and standard magnitude MRI were used. The blood flow in the false lumen was similar with either a proximal tear alone or both proximal and distal tears, but was lowest when there was only a distal tear. The model with only a proximal tear had the highest overall flow rate in the false lumen and the greatest changes in the size of false lumen during the cardiac cycle. They also monitored the characteristic flow jet from the true into the false lumen and *vice versa*. When only a proximal tear was present, a flow jet was observed into the false lumen during late systole and into the true lumen during diastole and early systole. For the model with both proximal and distal tears, a flow jet into the false lumen was present in mid-systole, and a reversed jet into the true lumen was present in mid-diastole at the upstream entry tear. At the distal entry, the flow jet into the true lumen was identified at early diastole and into the true lumen at mid-systole. When the lumina were connected only by a distal tear, the flow jet was observed entering the false lumen during late systole and early diastole, and entering the true lumen during end-diastole and late systole. The results showed that bidirectional flow in the false lumen only occurs when multiple tears were present. When bidirectional flow was present, the upstream and downstream flow waves collided and dispersed in the false lumen.

At the start of the twenty-first century, Chung et al. [615,616] used two *in vitro* models with a pulsating mock-flow loop to investigate the causes of true lumen collapse in aortic dissection. The first model consisted of a compliant, opaque bicycle tire inner tube combined with a PTFE graft material, while the second model was a rigid, transparent model made of PTFE. Through these models, they studied the effects of anatomic factors such as the location, number, and size of intimal tears, as well as the branch-vessel flow distribution. They also examined the effects of physiological factors such as the peripheral resistance in the branch vessels, the pump rate, and its output. Results showed that the collapse of the true lumen was associated with an increase in the size of the proximal tear. Furthermore, a decrease in the false lumen outflow rate was attributed to an occlusion of the false lumen branch vessels, while an increase in the true lumen outflow rate could be achieved by lowering the peripheral resistance in the true lumen branch vessels. Thus, both anatomical and physiological factors can potentially affect the true lumen collapse. They hypothesized that the differing inflow and outflow rate ratios between the true and false lumina create a pressure difference between the lumina, which subsequently moves the flap. In fact, it was more difficult to relieve the true lumen collapse than to prevent it. Repair of a proximal tear was the most effective treatment for preventing true lumen collapse in aortic dissection because it decreased the blood flow into the false lumen.

The impact of morphological parameters of the intimal tear (e.g., location, number, and size) on the pressure in the false lumen was also investigated by Tsai et al. [610]. They used three phantom models simulating the most common configurations in aortic dissection patients, see Fig. 40(a). These models included a proximal and a distal tear, only a proximal tear, and only a distal tear. The tear size varied between 3.2 and 6.4mm. Each model was connected to a pulsating pump to mimic the blood flow conditions in the lumina. The results showed that with a proximal and a distal tear, the systolic blood pressure was slightly lower and the diastolic pressure was slightly higher in the false lumen when compared with the true lumen. When the distal tear was absent, the diastolic pressure was elevated in the true lumen and the systolic pressure was also significantly higher. Conversely, in the absence of a proximal tear, the diastolic pressure was elevated and the systolic pressure was significantly decreased within the false lumen. The highest diastolic pressure in the false lumen was observed with a smaller proximal tear size and the absence of a distal tear. These *in vitro* models suggest that distal tears are associated with significant pressures in the false lumen that may not lead to thrombosis and remodeling.

In 2011, Dziodzio et al. [267] conducted an *ex vivo* study of the entire thoracic aorta of 26 pigs, including the supra-aortic branches. In the experiment, pulsating blood pressure was applied to the aortic annulus and intimal tears occurred in two distinct locations. The first location was 15mm downstream from the origin of the left subclavian artery, specifically at the concavity. This location typically represents a clinical manifestation that is considered rare. The second location was at the convexity site of the aorta. Analysis of the dissections revealed that 65% had a retrograde component, with the median antegrade and retrograde propagation lengths being 65 and 20mm, respectively, when the intimal tear was located at the convexity site. The retrograde dissection usually stopped at the left subclavian artery. For an intimal tear at the concavity site, the median retrograde propagation was 21mm, and in 16% of cases the dissection extended into the ascending aorta. However, in 31% of cases, antegrade propagation was stopped by the ligamentum arteriosum, which acted as an anatomical barrier. Additionally, retrograde propagation occurred at a later stage of the experiment, while antegrade propagation occurred in the initial stage.

One year later, Qing et al. [617] used 15 porcine aortas as *ex vivo* models to evaluate the hemodynamics in three different model types, which were distinguished by the location and number of intimal tears. They also developed models with only a distal tear, only a proximal tear, and tears present in both the distal and proximal areas. A pulsating pump was used to replicate the cardiac cycle, causing the dissection flap to move back and forth due to the pulsating blood pressure. According to the results, the flap played a role in controlling the blood pressure between the lumina. For instance, during systole, the false lumen began to expand, leading to compression of the true lumen. Additionally, in the model with only a proximal tear, the higher pressure in the false lumen caused more significant compression of the true lumen.

Rudenick et al. [618] conducted an *in vitro* study where they used a phantom model made of latex with synthetic geometry to create three different models. These models had either a distal tear, a proximal tear, or both to analyze the pressure, velocity, and flow pattern in the true and false lumina. The diameter of the intimal tears varied between 4 and 10mm, corresponding to 10 and 25% of the dissected segment. Larger tears caused pressure equalization in both the true and false lumina, leading to slower velocities and a complex flow pattern in the false lumen. In contrast, smaller tears not only resulted in lower pressure but also in higher tear velocities and a well-defined flow pattern within the false lumen. This suggested that the hemodynamics of the false lumen are highly dependent on the cumulative size of the tears. The study also revealed that peak systolic pressure in the false lumen was lower and occurred later in the cardiac cycle than in the true lumen. Furthermore, blood flow across the intimal tear was bidirectional and independent of the model used. Interestingly, there was minimal blood flow between a distal and proximal tear or *vice versa*.

In their *ex vivo* experimental study, Faure et al. [619] investigated whether the location of an intimal tear in human aortas is related to the pattern of dissection propagation and also evaluated the suitability of endovascular implants for human use. To accomplish this, they harvested 20 human aortas and created a 20mm intimal tear below the left subclavian artery at four different locations: lateral, medial, anterior, and posterior. They then used a bench-top pulsating flow model to induce an antegrade dissection propagation. The results showed that the antegrade propagation always reached at least the celiac trunk. In 80% of cases, the dissection propagated to the renal arteries, and in 35% of cases it even propagated to the infrarenal aorta. Notably, when the intimal tear was located at the lateral or medial site, the false lumen typically involved both the left and right renal arteries.

In a series of studies, Veger and his colleagues then studied different *ex vivo* porcine aortas. In one study, Veger et al. [620] created artificial dissections in two *ex vivo* porcine aortas to examine the role of branch vessels in type B dissection. For this purpose, a thin cannula was inserted into the false lumen, mimicking a branch vessel originating from it. The aorta was then placed in a validated circulatory system, which replicated physiological pulsatile flow and pressure characteristics of 130mmHg/70mmHg. This cannula was connected to a small silicone tube equipped with an adjustable valve mechanism. Three distinct valve settings were applied to create outflow from the false lumen: fully closed, half-open (50%), and fully open. Lumen areas and flow rates were measured using 4D-flow MRI with periodic triggering. They found that an increase in antegrade outflow through the branch vessel of the false lumen significantly expanded the mean false lumen area at both proximal and distal locations in both models. In conclusion, when no distal entry tear was present, increasing antegrade outflow through a branch vessel originating from the false lumen resulted in an expansion of the cross-sectional area of the false lumen. Utilizing the same validated *in vitro* circulatory system, Veger et al. [621] first conducted measurements of the true and the false lumen volume via CT imaging across six porcine models. In these models, a dissection flap was created in an aortic segment and then axially divided to induce fenestrations at various locations. Subsequently, in their third consecutive study, Veger et al. [622] employed three *ex vivo* porcine aortas and performed pressure measurements in both the true and the false lumen at several locations. After conducting baseline experiments, the aortic wall elasticity was adjusted with silicone, and the experiments were repeated to examine the effects of arterial stiffening and the resultant aortic dilatation. Finally, Veger et al. [623] conducted a study using a single dissected porcine model to investigate the impact of heart rate on hemodynamics, utilizing 4D-flow MRI data. Notably, also calculated WSS in the false lumen from the imaging data. For validation purposes, a healthy porcine aorta was also tested.

A silicone model of the aorta with a single proximal tear was developed by Birjiniuk et al. [624], utilizing CT imaging data. Flow velocity was measured and flow rates were calculated using a 4D-flow MRI. The study aimed to observe the properties of aortic flow and suggested that the model could be applied to more complex dissection geometries in the future. As a result of this baseline study, three consecutive studies were published, all of which were based on the same model. In the first study, Birjiniuk et al. [625] developed two models: a normal aorta model and a dissected aorta model, none of which included branches. The dissected model had a proximal and a distal tear, each of which was incised circumferentially along the flap on the lateral wall. The study found pockets of reverse flow and vortices primarily in the false lumen of the dissected aorta, with flow reversal being significantly greater in the proximal to middle dissection area compared with normal. The pulsatility caused unsteady vortices and a pumping motion of the distal dissection flap that corresponded to a flow reversal. The time-averaged velocity near stagnation in false luminal flow reversal could induce future thrombosis. Focal vortices could identify the location of tears that can potentially be covered with endovascular implants. The shape of the dissection flap changed over the extent of the dissection, mainly assuming a sinusoidal cross-sectional profile, with the greatest displacement of the flap observed at the proximal and distal tears. During peak systole, the distal portion of the flap remained collapsed onto the false lumen near the distal tear, achieving maximum true lumen distension. In their second study [626], they extended the model by adding linear, transverse fenestrations, approximately 25mm in length at locations 50 and 75% of the distance from proximal to distal tear, creating models with two, three, and four fenestrations. The study revealed the presence of pulsatile vortices and jet-like structures at fenestrations immediately distal to the proximal tear. The addition of fenestrations significantly reduced false lumen flow reversal, from 19.2±3.3% in dissections with two tears to 4.67±1.5 and 4.87±1.7% with each subsequent fenestration. In contrast, increasing pressure did not affect flow rates, flow reversal, or vortex formation. Moreover, increasing the number of additional fenestrations decreased flow reversal compared with two-tear dissection, which may prevent false lumen thrombosis, promoting persistent false lumen flow. Finally, in their third consecutive study, Birjiniuk et al. [626] extended the model to study the effect of endovascular stent-graft deployment in a model with multiple fenestrations, where fenestrations were placed similar to the previous study [626]. An initial 60mm graft was deployed to cover the proximal tear and to provide coverage of 60% of the initial dissection. Each subsequent graft was inset in the previous one to provide continuous coverage of the dissection. The study found that complete obliteration of the false lumen was achieved in the grafted aorta, resulting in normal parabolic flow profiles in the true lumen. A blind false lumen pouch was created distal to the grafted region with low velocity and highly reversed flows. In the distal free false lumen segments, flows were comparable to those without grafting. Visualization studies revealed antegrade flow in these regions with left-handed vortices of true-to-false lumen. The study suggests that coverage of the proximal tear alone is insufficient to restore true lumen area and flow along the entire extent of the dissection, suggesting inadequate collapse of the false lumen distal to the grafted region.

Marconi et al. [627] presented an *in vitro* study on the effect of location and size of the intimal tear on the hemodynamics in dissection using a pulsatile mock loop. A simplified geometry was 3D printed using a transparent rigid resin to form a stiff case. The silicone elastomer used in the experiment imitated the dissection flap separating the two lumina. The true lumen had a wall thickness of 3mm, while the false lumen and the flap had thicknesses of 1 and 2mm, respectively. In total, six access points were placed to measure pressure at different positions. Circular tears were created with three different diameters: 3,6, and 10mm, and in three variations: proximal, distal, and both proximal and distal. In addition, one reference configuration without tears was created. According to the study, a single intimal tear leads to higher relative false lumen pressure than two tears, particularly for the configuration with a proximal tear. The difference in relative false lumen pressures between a proximal and distal tear was minimal. Furthermore, the diameter of the tear affected the false lumen pressure, with a 3mm diameter resulting in a higher relative false lumen pressure than a 10mm diameter. Interestingly, the intermediate diameter of 6mm consistently produced lower relative false lumen pressures than both 3 and 10mm. Heart rate and mean blood flow within physiological ranges had an insignificant impact on false lumen pressure compared with the effects of proximal tears within physiological ranges. The study concluded that tears have a considerable impact on the connection between true and false lumina, but in different cases other factors such as the vessel wall elasticity, the presence of false luminal thrombus, and aortic geometry may can also play a role in various behaviors observed in clinical practice.

Following their initial study on layer-specific material properties in aortic dissection [455], Ahuja et al. [613] employed five *ex vivo* porcine models. These models were created with both entry and exit tears, designed for bench testing under static pressure, as shown in Fig. 40(d). In the experiment, a balloon was inflated to apply bending forces to either the middle or distal region of the dissection flap within the static aortic pressure environment. The main objective was to identify the radial pressures required to reassemble the middle and distal ends of the dissected aorta. To ensure the validity of their findings, they compared the experimental data with computational models, as described in Section 5.2.

**Fig. 41.**
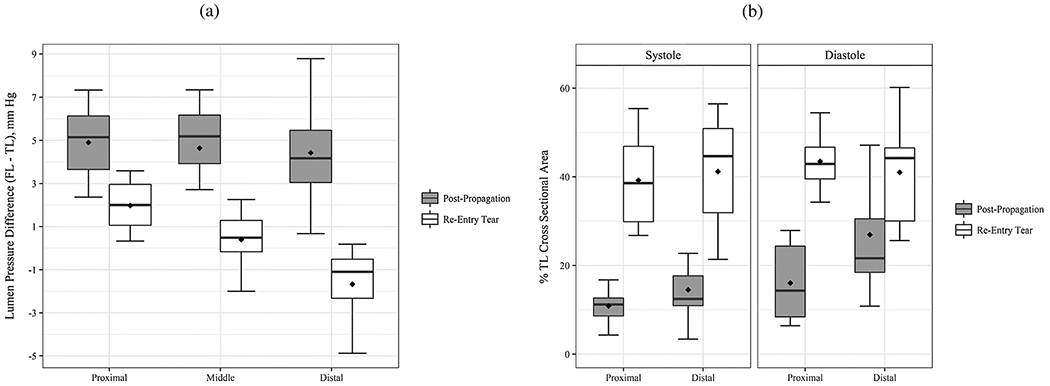
The boxplots illustrate (a) the spatial variation and mean lumen pressure difference between the true lumen (‘TL’) and the false lumen (‘FL’) compared for conditions both with and without a distal re-entry tear, and (b) the distribution and the mean percentage of true lumen cross-sectional area at peak systole and diastole, compared under conditions with and without re-entry tear development. Mean values are denoted by a diamond symbol within each boxplot. Figure is adapted from Canchi et al. [628] (licensed under CC-BY 4.0).

Canchi et al. [628] conducted an *ex vivo* study using 16 porcine aortas to assess the difference in luminal pressure and configuration of the dissection flap following dissection. Similar to Peelukhana et al. [606], they created a single proximal tear in each aorta, and used a pulse duplicator setting to apply an initial pulse pressure of 120mmHg/80mmHg, which increased to 140/80 and 160mmHg/80mmHg during the experiment. The pressure difference was then measured at three locations, namely proximal, middle, and distal, along the dissection flap, both with and without an additional distal tear. The results showed a mean pressure difference of 4.6mmHg across all locations when only a proximal tear was present, with higher pressures in the false lumen and a decreasing pressure difference along the length of the dissection flap, reaching a maximum difference at the distal end. However, the pressure difference disappeared with a distal tear, see Fig. 41(a). The study also evaluated the configuration of the dissection flap and found that it curved toward the false lumen during systole in 37.5% of cases, which increased to 75% with a distal tear. A dynamic movement of the dissection flap over the cardiac cycle was also observed, attributed to the pulsatile pressure. This, combined with the higher pressure in the false lumen, caused compression of the true lumen, as shown in Fig. 41(b), through the cross-sectional area of the true lumen. A notable finding was that increasing pulse pressure did not result in significant changes in the dimensions of the dissection flap in the circumferential and axial directions, indicating a structural limitation and an increase in stiffness of the dissection flap.

In their research, De Beaufort et al. [160] examined the precision of 4D-flow MRI scans for evaluating true and false lumina flow rates in *ex vivo* porcine dissection models. This involved the inversion of a porcine aorta, the creation of intimal tears, and the expansion of the false lumen using a balloon catheter. After these procedures, the porcine model was integrated into a pulsatile flow loop. Flow rates were measured by two procedures, and the averages of these measurements were used for comparisons. The 4D-flow MRI and 2D PC-MRI were compared against each other, as well as against a reference standard of sonotransducer flow measurements. The conclusion drawn from this study was that the 4D-flow MRI method is a reliable tool for assessing flow within the true and false lumina of the aorta.

Salameh et al. [629] created an *in vitro* model using eight plexiglass models to study the hemodynamics of patent and non-patent aortic dissection. The model mimicked only the dissection present in the aortic arch and was attached to a continuous flow circulatory system with water acting as a blood surrogate. Particle image velocimetry was utilized to measure the velocity field, while laser-induced fluorescence was used to visualize the flow and quantify the perfusion of injected dye, similar to contrast-enhanced CT. The model was separated into two categories: four with a patent false lumen and four with a non-patent false lumen. Each group was then divided into four cases, with one of the tears having a different size than the other model and a rigid flutter feature appearing at either the proximal or distal tear. The location of the initial tear in the aortic arch in a patent false lumen had a significant impact on the velocity of blood flow in the true and false lumina. The velocity of blood flow was determined by the size of the tear, with the smaller tear providing the maximum velocity in the false lumen. As the true and false lumina merged at the distal tear, the velocity difference created vortices that moved downstream along the wall of the non-dissected aorta. In a non-patent false lumen, there was no flow and all the blood flowed through the true lumen. However, in a patent false lumen, the perfusion rate of the dye was comparable to that in the true lumen and occurred simultaneously.

A combined experimental and computational study was conducted by Zadrazil et al. [630] to analyze the flow characteristics in the true and the false lumen over a range of intimal tear sizes. To do this, a simplified model made of perfluoroalkoxy was created, which included the ascending aorta, the aortic arch, and the descending aorta. The model was designed to be a 1:2 scale of an idealized aortic geometry, with a constant internal diameter of 16mm. To obtain realistic flow conditions, deionized water, seeded uniformly with inert particles, was pumped through the simplified model with a pulsatile blood pump. Four different tear sizes and size ratios were tested, and the phase-averaged velocity vector maps at mid-acceleration, mid-deceleration, relaminarization, and peak-systole were measured. The experimental results showed that an increase in the distal tear size increased the flow rate, decreased the WSS, and decreased the true-to-false lumen pressure difference. Conversely, an increase in the proximal tear size increased the flow rate, the WSS, and the pressure difference. Notably, the highest WSS was located at the edge of the intimal tear. The experimental results were then used to validate an *in silico* model, which is further discussed in Section 6.1.

Liang et al. [611] conducted a study using 24 porcine aortas to investigate the hemodynamic effects of various *ex vivo* aortic dissection models. The aortas were randomly divided into four groups, with group A serving as the control and groups B to D representing the aortic dissection group. To create the aortic dissection models, as shown in Fig. 40(b), an intimal tear was made 30mm below the left brachiocephalic artery using a scalpel. This tear was then enlarged to 18 to 20mm using plastic tweezers. A spatula was used to separate the intima and media until the false lumen reached a length of 150 (group B) or 200mm (group C). Group D had a 200mm false lumen with a proximal and a distal tear. The groups were placed in a mock circulation loop to simulate realistic flow and pressure conditions, with the flow distribution rate of the aortic branches calculated, and Doppler ultrasound used to visualize the aortic dissection structure and measure the velocity of flow in both lumina. This study found that the systolic pressure at the aortic inlet was higher in the aortic dissection group compared with the control group. The dissection flap showed pulsatile movement, particularly in groups B and C without a distal tear, suggesting that the flap motion was associated with comparatively higher pressures. In contrast, the pressure in the true and false lumina of model D was almost the same due to the lower flap oscillation. The study also revealed that the flow velocity at the distal end of the false lumen in the aortic dissection group was lower than that in the true lumen, which may lead to stagnant blood flow and platelet aggregation.

In the study by Chen et al. [631], patient-specific silicone models of both a normal aorta and an aortic dissection were incorporated into a mock circulation loop that enabled precise measurements of flow rates and pressure conditions in the aortic branches. Attached to each of the 12 branching vessels was a three-element Windkessel model. Significant differences were observed when comparing the normal and dissected aortic models under resting conditions. In particular, the dissected aortic model exhibited elevated systolic and diastolic pressures. Additionally, the time-average velocity was higher in the true lumen than in the false lumen. The direction of blood transport between the true and false lumina varied in different fenestrations, providing further insights into the intricate dynamics of aortic dissection.

Based on a previous study on a normal aorta [632], Zimmermann et al. [612] performed an *in vitro* study using 4D-flow MRI and coupled *in silico* simulation through FSI to examine how changing the size of proximal and distal tears affects hemodynamics. They utilized these *in vitro* results to confirm the accuracy of the coupled *in silico* simulations. The study used a patient-specific 3D printed aortic dissection model, which was created in three versions: one original model based on CTA, and two others with smaller proximal and distal tears, respectively. The model was placed in a flow-controlled and pressure-controlled setup to perform MRI imaging (Fig. 40(c)). The luminal pressure data were recorded at different locations in the true and false lumina, while an MRI-compatible flow circuit with glycerol-water as a blood-mimicking fluid was used for MRI signal enhancement.

**Fig. 42.**
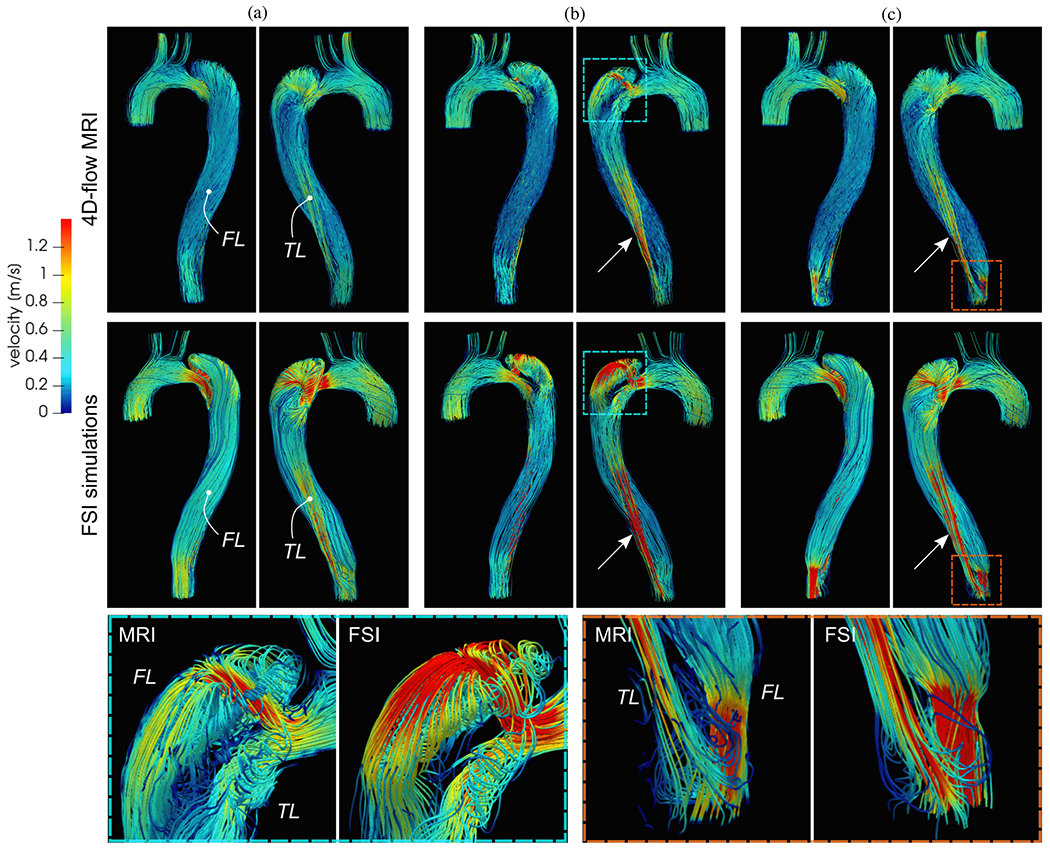
Comparison between 4D-flow MRI images and FSI simulation results showing the streamlines at peak systole for three models: (a) original model (‘TL’: true lumen; ‘FL’: false lumen), (b) model with a smaller proximal tear, and (c) model with a smaller distal tear. Each model has distinct local flow characteristics. First, the region of the proximal tear experiences increased flow velocities, particularly in model (b), and there is localized helical flow in the proximal true lumen and the false lumen near the proximal tear. Second, in models (b) and (c), the flow velocity in the true lumen is increased (arrow), and finally, the measurements in model (c) show a flow jet through a small distal tear with recirculating true lumen flow distal to the distal tear. Figure is adapted from Zimmermann et al. [612] (licensed under CC-BY 4.0).

The study found that each of the three models had unique flow patterns and velocity distributions, as shown in Figs. 42(a) to (c). The proximal tear region exhibited highly complex flow patterns, while helical flow patterns were observed in the proximal true lumen near the proximal tear. The modified models in the distal part of the dissected aorta showed increased flow velocities in the true lumen, consistent with higher true lumen net flow volumes in these models. The model with the smaller distal tear also showed a recirculation zone distal to the distal tear, while the other models showed unidirectional laminar flow in this region. During diastole, additional flow oscillations were observed through the proximal tear and helical flow in the false lumen was retained. The size of the tears had a significant impact on the inter-luminal pressure differences and flow splits, with the difference between peak and systolic pressure increasing in the reduced proximal tear model but becoming negative in the reduced distal tear model. The inlet to outlet pressure drops were lowest in the original model and larger for the modified models, while both modified models showed a significant reduction in false lumen flow ratio compared with the original model. This suggested that the flow is determined by the total resistance and not by the location of the narrowing. The model with the smaller distal tear had increased outflow resistance and false lumen flow ratio, which contributed to false lumen pressurization. In addition, they observed that in the model with a smaller proximal tear, the flow jet impinged on the opposite wall of the false lumen through the entry tear. They hypothesized that this could potentially result in tissue degradation and destructive remodeling via mechanobiological pathways, leading to false lumen dilatation due to the presence of local pressure gradients at the inner aortic wall. Readers are referred to their follow-up study for further investigation on this matter [342], see Section 7.2.

The main limitation of this study is related to the comparison between 4D-flow MRI and FSI simulations, which requires further attention. When comparing the visualized streamlines from 4D-flow MRI and FSI simulations, they appear remarkably similar, including recirculation zones, flow helices, and the flow jet through the tears. However, FSI simulations generally showed higher flow velocities compared to 4D-flow MRI, except during diastole where 4D-flow MRI displays higher velocities. They attributed these differences to two factors: first, the lower resolution of 4D-flow MRI compared to 2D PC-MRI may reduce peak flow velocities. Although 4D-flow MRI often underestimates peak velocities compared to 2D PC-MRI, this difference depends generally on several factors [633]. Second, FSI simulations experience lower damping due to differences between the computational model and the 3D printed model, as the latter has larger cross-sectional areas, leading to greater compliance, possibly due to underestimated resin stiffness. This is evident from the initial well-matched flow rates at the landmark inlet, but higher systolic and lower diastolic flow rates in FSI simulations at all downstream landmarks, as shown in their study. Overall, the well-matched streamlines between 4D-flow MRI and FSI simulations are particularly noteworthy.

**Fig. 43.**
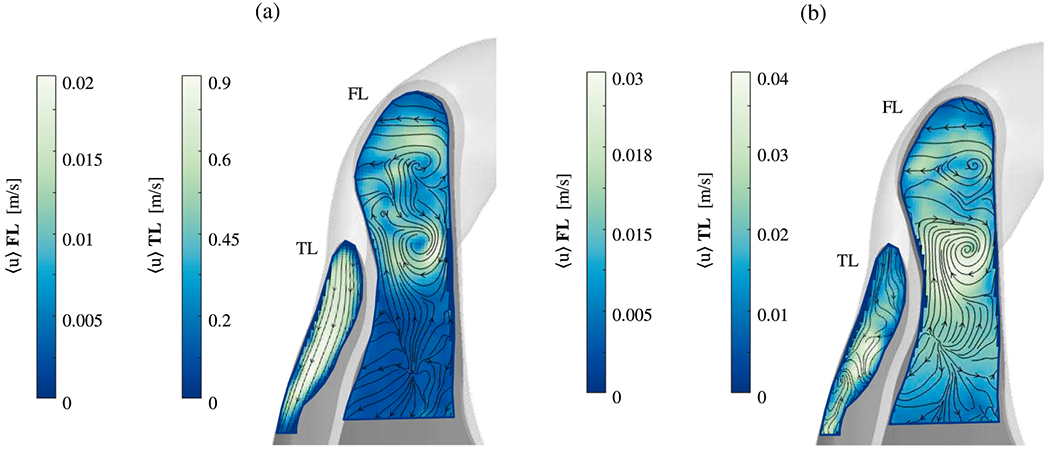
Phase-averaged velocity magnitude fields ⟨*u*⟩ were analyzed in the proximal part of the true (‘TL’) and the false lumen (‘FL’) at (a) peak systole and (b) diastole. The velocity magnitude is color-coded, while the flow direction is indicated by overlapping streamlines. Figure is reprinted from Franzetti et al. [636] (licensed under CC-BY 4.0).

In another study series, Franzetti et al. [634] developed a mock loop in which a computer-controlled pulsatile pump system combined with a tunable three-element Windkessel simulator was used to reproduce *in vitro* vascular physiological conditions to study complex patient-specific vascular pathologies. In this initial study, this mock loop was tested to verify its overall mechanical and hydraulic behavior. Subsequently, a follow-up study was published by Bonfanti et al. [635], which was based on the previously developed mock loop. Using 3D printing technology, a rigid and transparent 3D model of a patient-specific chronic aortic dissection was fabricated from TuskXC2700T. The model featured inlet and outlets such as the brachiocephalic trunk, left common carotid, left subclavian artery, and descending aorta, where it was connected to the remaining experimental setup. However, for simplicity, abdominal aortic branches were excluded. According to the CT scans, there was a proximal tear approximately 10mm distal to the proximal end of the dissection. No further communication between the lumina was evident from the CT data. *In vivo* waveform data were obtained from 2D PC-MRI scans acquired at multiple locations along the aorta. The study found that during the acceleration phase and the peak systolic phase, the velocity profiles in the aortic arch were shifted toward the inner curvature of the arch. Moreover, a highly organized motion with parallel streamlines was observed during the systolic phase, as shown in Fig. 43. Flow separation and bidirectional velocity profiles were observed during the deceleration phase and early diastole. Flow in the aortic arch during diastole was characterized by disorganized streamlines. Particle image velocimetry-derived phase-averaged velocities showed organized and high-velocity flow in the narrowed true lumen during the systolic phase, whereas the false lumen was dominated by vortical and complex flow structures during both systole and diastole. The absence of distal tears in the patient led to a zero net-flow entering the false lumen via the proximal tear, with recirculating flow patterns throughout the cardiac cycle. The flow structure and velocity magnitude in the false lumen remained relatively constant throughout the cardiac cycle, with slightly higher velocities observed during diastole. The results were then used to validate *in silico* hemodynamics simulations. In their study, Franzetti et al. [636] re-examined and expanded previous findings and investigated the evolution of vorticity, Reynolds shear stress distribution, and turbulent kinetic energy. Vortex formation with low shear rate values occurred in the false lumen throughout the cardiac cycle. This pattern has been linked to false lumen thrombosis due to the presence of small vortices and stagnant flow. During acceleration, deceleration, and diastole, the flow fields became disorganized and flow reversal occurred, leading to enhanced flow mixing, increased velocities and velocity gradients. Reynolds shear stress and turbulent kinetic energy also exhibited higher values during deceleration and diastole, while the latter showed low values during acceleration and peak systole due to the stabilizing effect of flow acceleration. Overall, hemodynamic indices have been associated with platelet activation, hemolysis, and blood coagulation.

Building on previous studies and the developed experimental model, a reduced-order model was subsequently developed to encapsulate the primary flow features within a patient-specific aortic dissection [637]. A proper orthogonal decomposition and robust principle component analysis was applied on *in vitro* hemodynamic data acquired by particle image velocimetry. The decomposed flows were then compared with those derived from hemodynamic simulations for the same geometry and flow conditions. This approach significantly simplifies the problem and promotes computationally more efficient flow simulations. Such advances could pave the way for translating these models into clinical practice.

Lastly, Aghilinejad et al. [638] developed a framework for 3D printing compliant, patient-specific aortic dissection phantom models. This framework was based on CT imaging and utilized a novel deep-learning-based segmentation method. Notably, data from 19 patients were used in the process. The deep-learning architecture was trained on a data set of 15 unique CT imaging scans from dissection subjects and then blind-tested on four additional sets of scans intended for fabrication. After segmentation, 3D models were created and printed using polyvinyl alcohol coated with latex. These models were then coated with latex to create compliant, patient-specific phantom models and implemented in a systemic circulation system. The *in vitro* experiments showed that the fabricated phantoms provided physiologically accurate pressure results that were comparable to MRI scans.

### 4.4. Key findings and publication index

To summarize this section, we present the publication index of experimental models with a focus on *in vivo*, *ex vivo*, and *in vitro* approaches in Table 5. Essential information about the modeling approaches and the objectives of each study is included. Finally, the main findings are listed conclusively.

- Needle experiments yielded pressure–volume curves necessary for dissection propagation, which were classified into three distinct categories: distensibility, peak pressure, and dissection propagation. The pressure required for dissection propagation may vary, depending on the site and intrinsic microstructure of the aorta. An increase in pressure is needed for propagation in the abdominal aorta, while the required pressure for dissection propagation in the thoracic aorta decreases as the volume of injected fluid increases.
- Blood pressure and pressure gradient were the most important factors in dissection propagation. In fact, the pressure gradient, referring to the pressure change over time, i.e. (d*p*/d*t*), appears to have the greatest impact.
- The dynamic motion of the dissection flap over the cardiac cycle was driven primarily by the pressure difference between the true and false lumina. This difference, typically in the range of 5mmHg, was determined by the location, number, and size of the intimal tears. In particular, a larger size of intimal tears size usually resulted in lower pressure differences.
- Anatomical barriers such as the ligamentum arteriosum or plaque in atherosclerotic aortas could stop or impede dissection propagation. Additionally, antegrade propagation typically occurred in the early stage of aortic dissection, while retrograde propagation was observed at a later stage.
- Luminal pressures in both the true and false lumina were influenced by the location, number, and size of intimal tears. During diastole, the false lumen exhibited higher pressures, whereas during systole, the pressures in the false lumen was lower. A large entry tear led to equalized pressure between the lumina, while a smaller entry tear created a higher pressure difference.
- A correlation was found between the depth of the intimal tear and the pressure required for dissection propagation. Specifically, a deeper tear required less pressure to initiate dissection propagation. This phenomenon could be attributed to the increased stress in the outer wall of the false lumen, which leads to a greater circumference of the outer wall of the false lumen.
- The flow was characterized by high-velocity flow in the narrowed true lumen during the systolic phase, while the false lumen was dominated by vortical and complex flow structures during both systole and diastole. Moreover, the flow throughput was mainly determined by the total resistance of the tears. Because there was no exit tear, there was almost zero net flow entering the false lumen. Bidirectional flow was regularly observed in multiple tears, particularly during the deceleration phase and early diastole.
- Smaller entry tears led to a stronger flow jet through the tear, which impinged on the outer wall of the false lumen. This could potentially activate specific pathways of SMCs and subsequently lead to wall remodeling.

## 5. Multiscale material models on damage and failure

Mechanical models in continuum mechanics lead to initial and boundary value problems in 2D or 3D. As outlined above, the involved aortic wall is characterized by spanning multiple scales, from macromolecules, to fibers and layers up to the organ level. Given the complexity of the mechanical organization and behavior of the aortic wall, (nonlinear) multiscale models with nonlinear characteristics become a natural and crucial component for providing meaningful descriptions of the mechanics in both healthy and pathological aortic states. For an introduction to the various applied models in this section, readers are referred to the relevant literature on fibrous tissue modeling [83,110,641], damage and failure modeling [642,643], and crack propagation [25,644–646], as these topics are beyond the scope of this review article.

In this section, we review multiscale material models that have been developed to investigate the pathological wall of the aorta from a solid mechanics perspective. By demonstrating the outcomes of different damage and failure processes, these models offer valuable insights into the initiation and propagation process of aortic dissection. They account for several key factors, including the delamination behavior of dissected tissue based on experimental data, the direction of dissection propagation, the degradation of specific constituents, the impact of focal areas of medial degeneration such as the accumulation of GAGs, and the growth and remodeling of the different tissue layers. In conjunction with other computational techniques, most importantly, finite element methods (FEMs) and some of their variants are essential tools to approximate solutions to boundary value problems derived under specific model assumptions. While modeling the dissected aorta at different scales might not immediately affect the clinical outcomes for patients with aortic dissection, it can foster a better understanding of pathological processes. Furthermore, when implemented in patient-specific studies, it may contribute to long-term improvements in clinical treatments.

### 5.1. Delamination of the wall

While there may be other studies that have applied multiscale material models to aortic delamination, the first substantive discussion on material models in relation to the pathology of aortic dissection was by Rajagopal et al. [11]. They introduced a continuum model that accounted for the orthotropic and viscoelastic behavior of the aortic wall to study aortic dissection delamination. An initiation criterion was proposed based on stress in the delamination area, with the assumption that prolonged exposure to high stress could cause delamination. Although this study did not apply the proposed model, it discussed key hemodynamic and mechanical factors contributing to aortic dissection initiation and propagation. They posited that the two primary hemodynamic factors were mean and maximum systolic aortic pressure, pulse pressure, and cycle frequency, all of which relate to the cyclic, pulsating load on the aorta. The main mechanical factors identified were aortic wall anisotropy, causing high axial and circumferential stresses, significant shear stresses from aortic torsion, and the composite structure of the aortic wall, which is held together by adhesion. Dissection propagation is thus promoted by cyclic loading on the anisotropic aorta. However, they noted that tears between aortic layers can only occur due to microcracks or inherent weaknesses, both acting as local stress concentrations. They also questioned whether maximum systolic ejection max(d*p*/d*t*), a putative hemodynamic factor for dissection progression introduced by Prokop et al. [601] and later validated experimentally [43,647], is an appropriate independent hemodynamic measure for dissection propagation. They argued that max(d*p*/d*t*) may be a helpful predictor but oversimplifies the complex hemodynamics and aortic mechanics. Pressure is position-dependent, and using a global, position-averaged pressure over time is inaccurate, especially since dissection is spatially inhomogeneous. Additionally, no experimental studies have yet demonstrated that dissection propagation is a function of d*p*/d*t*, where d*p*/d*t* is an independent hemodynamic variable. In fact, d*p*/d*t* is more related to pulse pressure and heart rate and influences dissection propagation indirectly.

It appears that the computational model published by Gasser and Holzapfel [648] was one of the first to investigate the delamination process in aortic dissection. To simulate aortic delamination, they used the partition of unity FEM (PUFEM), in which a constitutive relationship, based on a transversely isotropic traction separation law of exponential type with isotropic damage, was employed to characterize the cohesive zone. The resulting model was then applied to examine balloon angioplasty coupled with aortic dissection using a thin aortic slice obtained from MRI. For this analysis, the aortic wall was separated into two layers: the media combined with the fibrous intima, and the adventitia. Assuming a stress-free configuration, residual stresses were not taken into account. Material parameters of the aortic wall were derived from previous experiments [649] using the model by Holzapfel et al. [65], referred to as the Holzapfel-Gasser-Ogden (HGO) model, while the cohesive zone constitutive parameters were based on peeling experiments [433]. Interestingly, the results showed that dissection propagation protected other parts of the aortic wall from damage. Furthermore, they discovered that mode I fracture plays a prominent role in dissections, making it perhaps the most important mechanism for dissection progression.

**Fig. 44.**
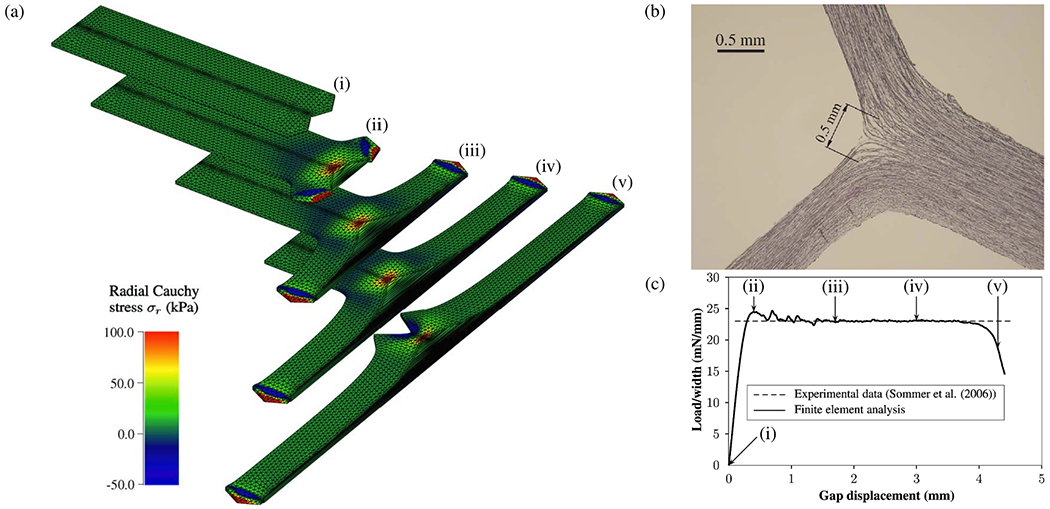
(a) Distribution and evolution of radial Cauchy stress during propagation of aortic media dissection driven by displacements on two attached rigid bodies. (b) Photomicrograph of a dissected human aortic media cross-section from a peeling experiment showing distinct cleavage between elastic lamellae and fiber bridging within the cohesive zone – cohesive length approximately 0.5mm. (c) Comparison of the average experimental load with the calculated load per width for the propagation of human aortic media dissection, showing very good agreement. Figure is adapted from Gasser and Holzapfel [650] with permission from Elsevier.

In a subsequent study, in 2006, Gasser and Holzapfel [650] utilized the computational framework they had developed earlier [648] to conduct a peeling test on aortic tissue. In this study, they chose a simple stress-based initialization criterion as described in Gasser and Holzapfel [651]. The peeling test of a medial strip, which utilized the so-called Gasser-Holzapfel-Ogden (GHO) model [652] incorporating an isotropic ground substance and dispersed collagen fibers, was validated by experiments involving direct tension and peeling tests on aortic tissue [433]. Fig. 44 shows the strong correlation between numerical outcomes and experimental data.

Following a different approach, Ferrara and Pandolfi [653] employed a 3D finite element model to examine dissection progression by performing a peeling test on a medial strip. They used the HGO model for aortic tissue modeling and an anisotropic cohesive model with a linearly decreasing cohesive law, adapted from a previous study [654], to describe crack propagation. This model includes an anisotropic failure criterion and a cohesive law, allowing a distinction between cohesive responses along various directions on the cohesive surface. The failure criterion relies on the normal and shear traction resistance of the surface. The geometry of the medial strip, material parameters, and cohesive model parameters were based on Gasser and Holzapfel [650]. The numerical results demonstrated good agreement with experiments. Dissection in the axial direction exhibited oscillating behavior, while it appeared more regular in the circumferential direction. Moreover, they found that reduced shear strength of the aortic tissue resulted in a smoother response and decreased average pulling force. Then, experimental observations by Tsamis et al. [655] inspired the development of a microstructure-based peeling test. These observations revealed that elastic and collagen fibers form bridges between the layers of the media, which often appear disrupted and broken. Building on this, Pal et al. [656] modeled radially-oriented collagen fibers as bridges between layers that rupture when a specific dissection energy threshold is reached, using a nonlinear exponential force-separation law with a linear drop. The failure energy of each fiber bridge is based on the individual fiber energy, which is represented by the area under the force-separation diagram. A 2D peeling test was conducted with constitutive parameters derived from previous experimental studies [657,658]. When analyzing the computational results, the average peeling tension remained constant and corresponded to the experimentally determined delamination strength. Overall, they achieved strong qualitative agreement with the experimental findings.

Later, Shah et al. [659] proposed a multiscale model to characterize both pre-failure and failure responses of the porcine ascending aortic media. This multiscale model consists of three levels: the finite element domain at the millimeter-scale, the representative volume element domain at the micrometer-scale, and the fiber domain with radii at the nanometer-scale. The representative volume element, defined by an idealized network of discrete fibers with uniform diameter embedded in an isotropic matrix material, employed the GHO model to describe its mechanical behavior. Failure was modeled at the fiber domain, assuming that individual fibers fail when stretched beyond a critical threshold. To consider a wide range of failure lengths, a stochastic failure model was applied. The multiscale model was successfully validated against uniaxial failure experiments in both circumferential and axial directions as well as equibiaxial extension experiments as part of the study. Witzenburg et al. [660] built on and improved the computational model developed by Shah et al. [659], while also conducting peeling and shear tests on porcine tissue. Driven by the initially inaccurate out-of-plane behavior of the model, they incorporated the layered structure of the lamellae and their interlamellar connections. After validating the model against their experimental results and the experiments of Shah et al. [659], the model successfully captured the clear difference between the high tissue strength in the lamellar plane and the low strength of the interlamellar connections.

The delamination of aortic plaque was modeled by Leng et al. [661] using a cohesive zone model. They employed the HGO model for material representation and conducted delamination experiments. The applied traction-separation law, which included damage initiation, evolution, and element removal, featured failure criteria based on the energy release rate. Cohesive model constitutive parameters were fitted to experimental data [410,650], although some origins remained unspecified. The finite element model, comprising medial and adventitial layers, was consistent with experiments on delamination of atherosclerotic plaques in abdominal aortas of mice [410]. Later, Leng et al. [662] utilized the cohesive zone model to simulate aortic delamination, incorporating the HGO model. They developed a detailed aortic strip consisting of intimal, medial, and adventitial layers, and meshed the geometry with hybrid elements to ensure incompressibility. To determine the anisotropy of the aortic wall, mixed-mode and T-shaped peeling tests were performed in axial and circumferential directions. The energy release rate was calculated based on peeling tests on porcine tissue as described in their study and incorporated the experimental results of [661]. The computational approach demonstrated strong agreement with the experimental findings.

In 2016, Thunes et al. [663] then introduced a 3D finite element model of the lamellar unit, a representative volume element for the ascending thoracic aorta, which directly incorporated the collagen fiber microstructure. Several network features, such as orientation distribution, intersection density, and areal concentration, were used to model the fiber architecture. The data were obtained from multiphoton microscopy images taken from human aneurysmal ascending aortic media specimens with a bicuspid aortic valve phenotype. The 1D fibers were embedded directly into a non-fibrous matrix. A bilinear constitutive relation with a tension–compression switch was employed for the collagen fibers, while the constitutive behavior of elastic fibers was modeled using a neo–Hookean model. The model was applied to a uniaxial extension test, with data from aneurysmal tissue obtained from a previous study [664]. The model was generally able to reproduce the typical J-shaped constitutive response. Notably, not all fibers began bearing load simultaneously, leading to regions of higher stress that SMCs could potentially be exposed to, triggering growth and remodeling. The degree of heterogeneity resulted from the local network architecture. Moreover, the stress distribution of the non-collagenous matrix after fiber recruitment was homogeneous at low stretches and highly heterogeneous at higher stretches. Later, Thunes et al. [665] extended this model to assess the role of the implemented structural features in determining in-plane tissue strength, which governs dissection initiation, i.e. the formation of an intimal tear. A damage parameter was introduced to monotonically decrease the stress upon reaching a peak stretch, thereby enabling the modeling of localized collagen fiber breakage. The results also indicated that the colocalized elastic lamellar stress exceeded the elastic strength.

Conversely, Nobel et al. [666] investigated dissection propagation using a rigid, planar wedge penetrating the delamination zone to study catheter-induced dissection in coronary arteries. They employed an incompressible Ogden model [667] with a traction-separation law and determined the critical release rate associated with tissue dissection from experimental studies [433,668,669]. Compared with the wedge-driven dissection experiments on porcine aortas [669], the finite element analysis demonstrated qualitative agreement with experimental data, although with less variation and size between samples.

Brunet et al. [461] proposed an analytical approach to replicate observations from X-ray imaging during uniaxial extension tests [670]. A 1D model was created, consisting of multiple layers representing the media of the aortic wall, each possessing distinct elastic and damage properties. The material behavior was modeled adopting a Mooney-Rivlin model [667]. Additionally, a stress criterion in the normal direction of the crack was defined with a linear damage evolution. However, this approach could only predict mode I failure. The results were subsequently validated against a 2D finite element model using a cohesive zone model, which showed good quantitative agreement. The model demonstrated that a crack initially forms due to mode I failure, and then propagated in mode I in the transverse direction. When the elastic recoil stress surpasses the mode II strength of the axial plane, the crack propagated in the direction requiring less energy, leading to the formation of a delamination plane. Therefore, mode II failure might play a crucial role in keeping layers together (due to earlier rupture in a parallel delamination plane) and has a significant impact on crack propagation.

**Fig. 45.**
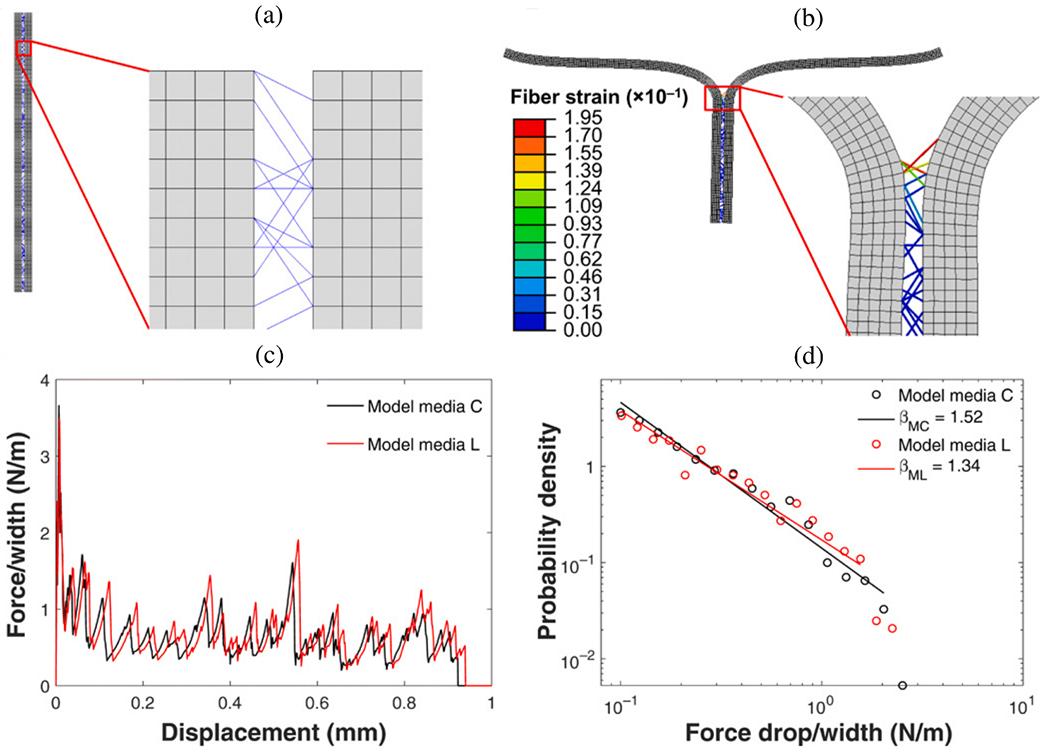
(a) Finite element model of two medial strips connected by discrete interlamellar collagen fibers and removed upon failure. (b) Engineering strain in fibers during dissection – most stretched fibers at the separation front. (c, d) Peeling force versus displacement in the finite element simulation reveals avalanche-like aortic failure during delamination (‘MC’: model outcome for circumferential direction; ‘ML’: model outcome for axial direction), where *β* is defined as the negative slope of a straight line fit. Figure is reprinted from Yu et al. [671] (licensed under CC-BY-NC 4.0).

Inspired by experimental findings, Yu et al. [671] conducted a peeling test involving two lamellae to demonstrate that the delamination process exhibits an avalanche behavior, described by a power law, as shown in Fig. 45. To achieve this, they modeled two lamellae, characterized by an isotropic neo–Hookean model, interconnected by discrete and randomly oriented collagen fibers. These fibers were represented by Timoshenko beam elements and a specific stress–strain relationship [672]. The connecting fibers were removed upon reaching an experimentally specified failure strain [673]. The random structural arrangement of interlamellar fibers, interpreted as the source of energy-releasing avalanches on a wide scale, was based on multiphoton imaging presented in their study. These multiphoton images revealed that the two elastic lamellar layers were connected by both elastic and collagen fibers, which were sparsely arranged and non-uniformly distributed in density and orientation. Additionally, peeling tests were performed in circumferential and axial directions using aortic media samples to validate the computational model. These experiments showed a sharp initial increase in delamination force before reaching a plateau region, followed by a sudden drop. The probability density distribution of these force drops suggested a power law distribution across nearly two orders. A comparison between the experiments and the computational model led to satisfying results. Furthermore, the finite element analysis showed that the more interlamellar fibers engaged before failure, the higher the failure strain. Consequently, the probability of higher force drops increases, resulting in larger avalanches and a smaller power law exponent.

Building on previous research, Wang et al. [674] explored the contribution of elastin and collagen fibers to intralamellar bonding through mechanical testing, multiphoton imaging, and finite element modeling. Mechanical testing involved peeling tests and biaxial extension tests in both circumferential and axial directions with a porcine aortic media and a purified elastin network, respectively. A comparison between the media and the purified elastin network revealed that the peeling force and energy release rate associated with mode I failure were significantly higher for the media. It should be noted that the peeling force oscillated considerably due to discrete microstructural events. Furthermore, axial peeling consistently exhibited higher energy release rates and strength. Multiphoton imaging revealed the recruitment of both elastin and collagen fibers, while also providing evidence that in-plane anisotropy of fiber directions could potentially explain the direction-dependent phenomena observed in peeling tests. Experimental results informed the development of a 3D finite element model of a peeling test, which included two initially separated tongues at the top and one layer of cohesive elements. The cohesive zone model featured a bilinear traction-separation law and focused on mode I failure due to the chosen testing methods. The constitutive model of the aortic media was represented by the GHO model. Biaxial extension test simulations using shell elements with an inherent plane stress assumption were also performed to determine constitutive parameters. In contrast, the constitutive stress versus stretch relation of the purified elastin network was obtained from a previous study [675]. The findings demonstrated that both elastic and collagen fibers play a crucial role in interlamellar bonding, strength, and toughness. They also emphasized that the contribution of elastin fibers to interlamellar bonding should not be overlooked, as pre-stretched elastin exerts an intrinsic compressive stress on collagen fibers, resulting in their waviness. A loss of interlamellar elastin also leads to stress concentration around the remaining fibers.

In summary, numerous multiscale models have been developed over the past decade using various approaches. These models serve as valuable tools for examining the failure behavior of the aortic wall during delamination and revealed, for example, a power law behavior in dissection propagation. However, it remains uncertain to what extent this behavior is related to the false lumen propagation in aortic dissection. As it has already been demonstrated that delamination strength decreases with age [676], it is likely that the pathologically altered aortic wall significantly influences delamination behavior and mechanisms. Consequently, related constitutive parameters are also greatly affected. Nonetheless, investigating delamination behavior is crucial to enhance our understanding of the delamination process in aortic dissection. Additionally, some of the models discussed here have the potential to be incorporated into a more comprehensive patient-specific, organ-scale analysis to model dissection propagation, which will be addressed later in this review article, see Section 9.2.

### 5.2. Dissection propagation and flap configuration

Modeling dissection propagation in a patient-specific context remains numerically challenging and seemingly infeasible. Consequently, recent developments in constitutive modeling have also focused on creating simplified or idealized geometries to study dissection propagation. These models effectively investigate dissection propagation and its direction by incorporating the healthy or pathological wall microstructure. Moreover, they allow for a detailed examination of the flap configuration throughout the cardiac cycle and possible changes during dissection propagation.

Wang et al. [675] conducted a study on tear propagation in soft tissues, focusing on the assessment of the energy release rate through an effective numerical method that employs an adaptive tear length. They utilized the HGO model to describe the uniform, fiber-reinforced material. To explore the impact of fiber orientation on dissection progression, they implemented a 2D strip featuring a single axial tear under internal pressure. The surrounding tissue was represented using a linear elastic model. The constitutive parameters of the HGO model were derived from studies on rabbit carotid arteries [65]. The findings indicated that fiber orientation, tear length, and surrounding tissue could influence the critical pressure for dissection. Notably, as fibers became more parallel to the tear, the energy release rate decreased due to the fibers bearing a greater load to resist tear opening. In contrast, the energy release rate increased consistently with tear length. The stiffness of the surrounding tissue was found to be a key factor in the propensity for tear propagation, with lower stiffness promoting dissection progression.

**Fig. 46.**
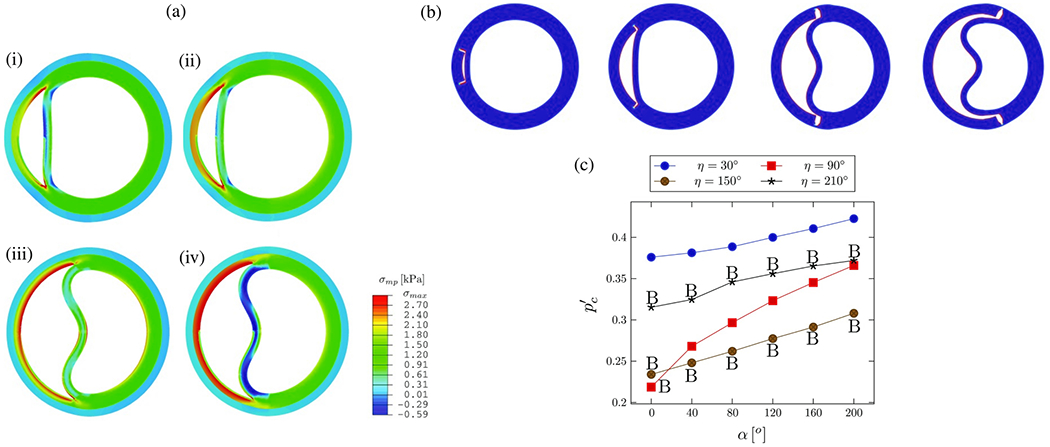
(a) Deformed configuration displays the maximum principal stress with varying tear lengths subjected to the critical pressure. Simulation results indicate that the dissection flap tends to buckle beyond a specific propagation length. (b) Steady deformed configuration with different tear length (between 30° and 210°), where all the tears propagate radially. (c) Dimensionless critical pressure $p_{c}^{\prime }$ versus the opening angle *α*, for four different tear lengths, where the letter ‘B’ indicates that the inner wall is buckled. Figure is adapted from Wang et al. [677] (licensed under CC-BY 4.0).

In the subsequent study, Wang et al. [677] expanded on their earlier approach by employing the extended FEM (XFEM) to simulate dissection propagation in a 2D cylindrical tube with a predefined tear. To ensure incompressibility, they utilized plane-strain hybrid elements to discretize the 2D geometry. In addition to applying a linear cohesive traction-separation law, residual stresses were incorporated into the model. Residual stresses in arteries can be determined experimentally using so-called opening angle experiments. In such experiments, a ring-shaped section of the artery is cut, causing it to spring open. The angle at which the ring opens, the so-called opening angle, directly measures the circumferential residual stress, which can then be taken into account in a constitutive framework [678]. Consistent with their previous research, the constitutive parameters were obtained from the literature [65]. By altering the tear length, they discovered that a longer tear is more prone to propagation, with the shortest and longest tears being the most stable. They also observed that all tears tended to expand radially outward (Fig. 46(b)). The dissection flap was found to buckle at certain tear lengths (Fig. 46(a)), which was subsequently confirmed by CT imaging of aortic dissection patients. Furthermore, residual stresses appeared to offer protection against tear propagation, as shown in Fig. 46(c). In summary, the study concluded that tear propagation is governed by an inherent interplay of tear length, wall buckling, fiber orientation, and residual stresses.

In their third study, Wang et al. [679] further applied the computational model introduced in their 2017 study to both stable displacement-driven peeling and unstable pressure-driven tear propagation. They demonstrated that the thickness of the flap might play a crucial role in predicting dissection evolution. Several key observations were made. First, for a long and shallow dissection flap, the flap would collapse, potentially slowing down tear propagation. Second, the critical propagation pressure was higher for a deeper and shorter tear. Third, a deep tear without buckling tended to propagate at a lower critical pressure, implying that the buckled state of the dissection flap redistributes energy and stresses within the wall, potentially hindering tear propagation. Finally, tear propagation typically followed the direction of the material axis with the highest stiffness, which corresponds to the fiber orientation. These findings shed light on the complex interplay of factors that influence the progression of dissection in soft tissues.

**Fig. 47.**
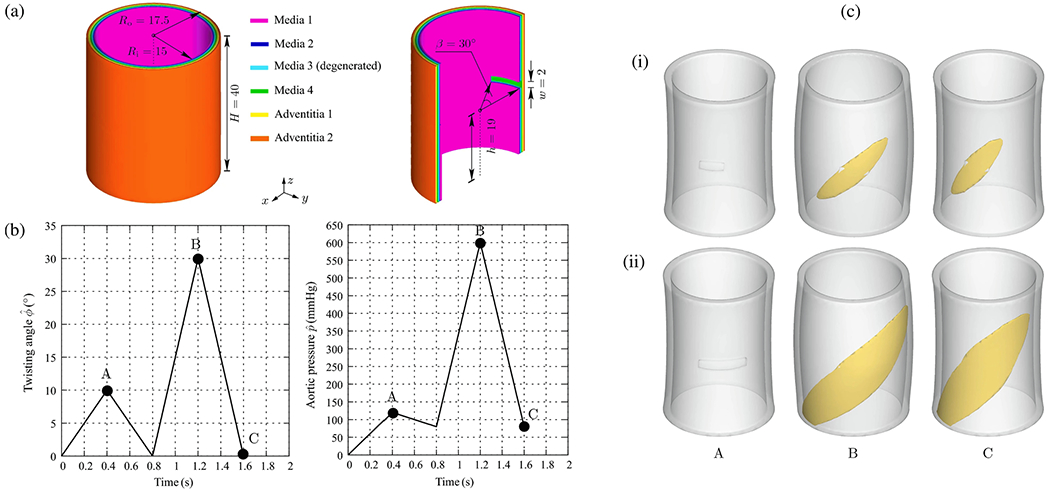
(a) Idealized geometry of an extracted 3D segment, consisting of four associated medial sub-layers and two represented adventitial sub-layers. Note that media 3 refers to a degenerated layer (with lower strength), where the sliced view of the geometry represents the prescribed representative initial tear size. All dimensions are in millimeters. (b) Physiological and supra-physiological loading conditions were applied to the idealized geometry, encompassing end-systolic twist, internal pressure, and constant pressure. (c) 3D evolution of the crack phase-field at the three load points A, B, and C, showing the influence of the applied loads. This progression reveals a helical crack pattern in the degraded media, distinguished by tear sizes: small (i) and large (ii). Isosurfaces are employed to illustrate the extent of the damage zone. Figure is adapted from Gültekin et al. [683] (licensed under CC-BY 4.0).

Building on the framework established by Raina and Miehe [680], Gültekin et al. [681] employed a phase-field approach to model damage and failure initiation in biological tissues. They adapted the Ginzburg–Landau type phase-field method, originally designed for brittle fracture in isotropic solids, to model the anisotropic aortic wall. The study incorporated the HGO model to represent the anisotropy arising from the collagen fibers in the aortic wall. To simulate the fracture of arterial walls, they identified and implemented an energy-based anisotropic failure criterion. The model was then applied to experimental data from uniaxial extension and simple shear failure tests [454]. This analysis demonstrated that the finite element simulations aligned with the experimental findings, validating the effectiveness of the adapted phase-field approach for modeling anisotropic biological tissues. In two consecutive studies, Gültekin and Holzapfel [25] first conducted a review of computational models for rupture in soft biological tissues. They then introduced a rate-dependent phase-field evolution and compared various failure criteria, including energy-based, Tsai-Hu, principal stress, and stress-based Hill criteria, see Gültekin et al. [682]. They found that the energy-based criteria resulted in more stable and physically significant crack growth. However, it is important to note that due to numerical issues in this study, they did not use experimental data to calibrate their constitutive parameters. In the latest research, Gültekin et al. [683] employed the phase-field model to study aortic dissection. They used a symmetric cylinder with a predefined tear, subjecting it to axial stretch, end-systolic twisting, and internal pressure. The aortic wall was represented using multiple layers: healthy media, degenerated media, and adventitia, which were based on experimental data [454]. It is important to note that the material parameters for the degenerated media were simply reduced by 20% compared with the healthy media (Fig. 47(a)). To simulate physiological and supra-physiological loading conditions, they adjusted the end-systolic twisting and internal pressure (Fig. 47(b)). In fact, their numerical analysis revealed a helical crack pattern in the damage zone surrounding the tear, which aligned with the fiber orientation, see Fig. 47(c). Additionally, they identified potential damage and fracture mechanisms that could occur in the aorta under mode I, mode II, and mixed-mode fracture conditions, such as collagen fiber pullout, collagen fiber bridging, collagen fiber–matrix debonding, and matrix cracking. Upon examining the findings, they concluded that dissection progression may be more likely to occur due to mode II fractures rather than mode I.

Inspired by earlier experiments, Ahuja et al. [613] validated a computational model of flap reapposition by comparing it with *in vitro* experiments using porcine aortas. For details, see Section 4.3 and their previous study [455]. These experiments ascertained radial pressures when a balloon catheter was inserted into a parallel channel model under a static inflation pressure. This experimental analysis was subsequently mirrored in a finite element model. They applied an anisotropic model, namely the HGO model, and assigned different material properties to the middle and distal flap segments, where the parameters were calibrated to biaxial extension tests [455]. The finite element models successfully represented the variations in radial pressures necessary for the reapposition of the dissection flap in correlation with pressure.

Mousavi et al. [684] introduced a layer-specific damage model based on constrained mixture theory to estimate the risk of tear formation by considering the internal stresses within the aortic wall. In this model, they utilized a damage evolution approach with a linear softening law derived from continuum damage theory. The mechanical behavior of collagen and elastic fibers was integrated using the HGO model, with distinct material parameters for tension and compression. The constitutive parameters of the material model were adjusted to fit experimental data [104,105] before it was applied to three representative examples to predict damage evolution. These examples included an inflation test of a perfectly cylindrical artery, a uniaxial extension test of a human ascending aortic aneurysm strip, and a bulge inflation test. The computational results demonstrated that the onset of damage begins at the intimal layer and extends through the media, but does not penetrate into the adventitia.

**Fig. 48.**
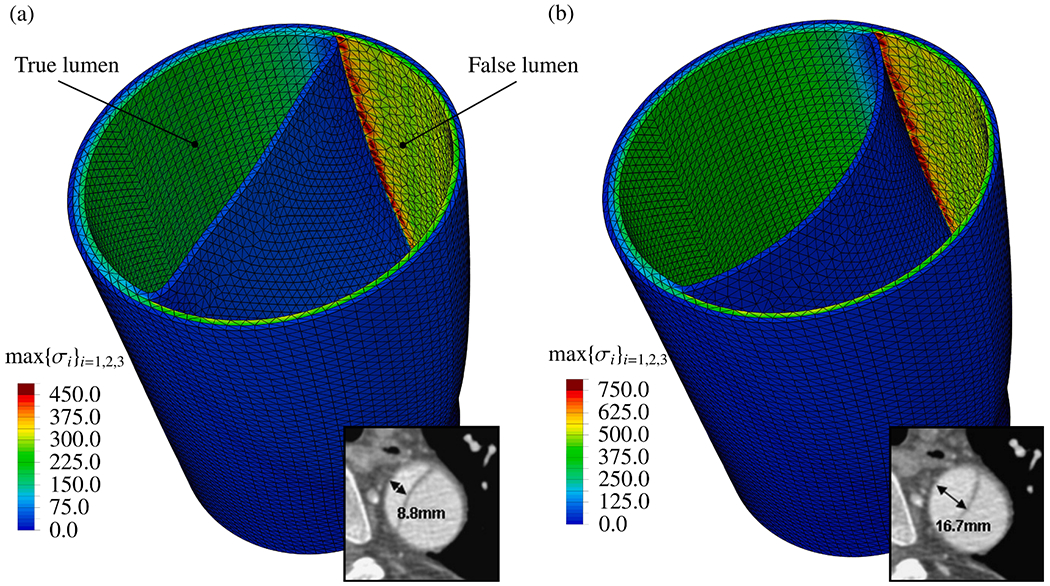
Computational results of the finite element analysis illustrate the maximal principal stresses (in kPa) in the true and false lumina at (a) diastole and (b) systole compared with evaluated CT images at the end of the aortic arch and show the minimum and maximum dimensions of the true and false lumina over the cardiac cycle. Figure is reprinted from Rolf-Pissarczyk et al. [685] (licensed under CC-BY 4.0).

Rolf-Pissarczyk et al. [685] later introduced a constitutive model that accounts for the pathological degeneration of inter-lamellar elastic fibers during aortic dissection. These fibers, aligned radially, contribute to the cohesion of lamellar units in the aortic media. The model incorporates a degradation parameter to exclude damaged or degraded elastic fibers from the strain-energy function by modifying the specific strain-energy term of the dispersed elastic fibers. For this purpose, they applied and extended a discrete fiber dispersion model, previously introduced for modeling collagen fiber dispersion [686]. They presented two computational models. First, the authors fitted the constitutive model of elastic fibers to experimental data from a single elastic lamella [687] and validated the model using two representative numerical examples: uniaxial extension and simple shear. Second, they developed an aortic dissection geometry with two distinct tissue layers, motivated by patient data [14,278], to investigate the effect of degraded radially-directed elastic fibers on stress distribution in aortic dissection. They adjusted the mechanical and structural parameters to match experimental data from their laboratory, existing literature [687–689], and thickness measurements [690]. The findings revealed that the stiffness contribution of elastic fibers might have limited impact on stress distribution at higher pressures. Additionally, they proposed a possible mechanism for dissection propagation: medial delamination caused by the stretch applied to the dissection flap. Specifically, the flap is stretched due to the cyclic dilatation of the true and false lumina, see Fig. 48, leading to its separation from the intact wall. In subsequent studies, Rolf-Pissarczyk et al. [450] utilized the discrete fiber dispersion method in a layer-specific model of the aortic wall, which accounted for passive constituents and the active contribution of SMCs, though only the mechanical behavior was modeled. Both fiber constituents and SMCs were represented using the discrete fiber dispersion method, enabling the modeling of pathological phenomena associated with aortic dissection such as lamellar loss of SMCs in dissected tissue.

The geometry of an intimal tear has a significant influence on the hemodynamics of the aortic dissection and thus on the dissection propagation. To quantify this effect on aortic dissection initiation and propagation, Brunet et al. [691] developed a model that compares various tear orientations to identify primary factors contributing to aortic dissection. This model featured a two-layered aortic wall, consisting of medial and adventitial layers, and employed the GHO model to represent collagen fibers. Residual stresses were incorporated using a uniform opening angle, while dissection propagation was modeled using an XFEM approach with a linear traction-separation law. Here, the stress-based anisotropic Hashin failure criterion was employed. Material parameters were calibrated using experimental data, including tensile extension tests from ascending aortas [692], radial and shear strength measurements from aneurysmal and dissected human thoracic aortas [433], and experimental data on the fracture energy [433]. The model was then applied to a tension-inflation test of an idealized aortic segment. Three distinct tear orientations were evaluated and a numerical design of experiments was employed to identify the most sensitive parameters affecting the aortic pressure required for intimal tear propagation. Seven key parameters were considered: crack length, depth, width, and position; tensile and shear strength; opening angle; and initial axial stretch. The tensile and shear strength direction components were varied equally. The outcomes of the analysis included the critical pressure and the plane of propagation. The average critical pressure ranged between 206 and 251mmHg. While shear strength did not demonstrate a significant influence, tensile strength was identified as the most influential factor due to the lack of consideration of the lamellar structure of the media. Additionally, the length and depth of the crack played a crucial role in the dissection propagation. For the aortic dissection to propagate closer to the adventitia, lower pressure was required, and the initial axial stretch contributed to a reduction in the critical pressure. In particular, the residual stress appeared to provide a protection against the dissection propagation in the aorta.

**Fig. 49.**
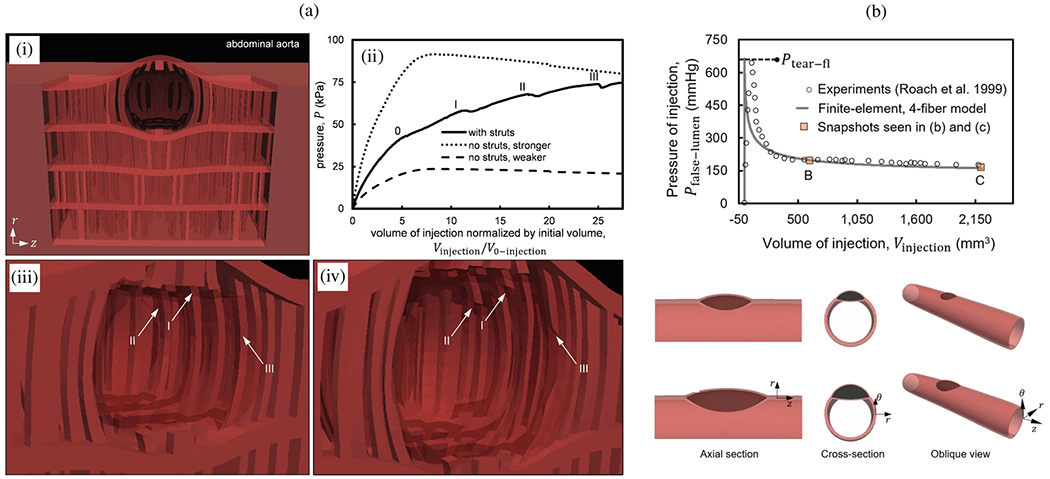
(a) Mechanism for the progressive, step-wise tearing of the abdominal aortic wall as observed in needle experiments by Roach and his colleagues: (i) snapshot of the microstructural model depicting the progressive tearing of the abdominal aorta; (ii) graph showing the injection pressure versus the injection volume normalized by the initial pool volume. Three cases are considered: a microstructural model, a model without struts and a weak, less stiff intralamellar material, and a model without struts but where the intralamellar material is represented by a tangential stiffness and *G_c_* equal to those of the surrounding medium. The peaks marked I, II, and III correspond to the pressure build-up and eventual tearing of the radial struts marked with the same numbers in (iii) and (iv). (b) Graph showing injection pressure–volume curves and the morphology of the injected volume, which are qualitatively and quantitatively consistent with the mentioned needle experiments. Figure (a) is adapted from Ban et al. [693], while (b) is from Ban et al. [696], with permission from Springer Nature, respectively.

Early needle-based experiments [592,593], followed by later studies of Roach and his colleagues [595,596], see Section 4.2, uncovered varying tear propensities to aortic dissection along the aorta, which can be explained by the inherent microstructure. In brief, the thoracic aorta displays thin elastic fibers that are oriented radially, whereas the abdominal aorta exhibits radial interlamellar connections resembling a honeycomb structure. As a result of this microstructural disparity, the abdominal aorta might be more prone to tearing, while the thoracic aorta is more prone to dissection propagation. Ban et al. [693] incorporated these failure characteristics into a phase-field model of the medial layer. Two models were developed: a homogenized model exhibiting nonlinear, anisotropic material behavior for the thoracic aorta, and a microstructure-based model for the abdominal aorta that explicitly included interlamellar struts. They utilized the neo–Hookean model for elastin and the HGO model with four fiber families to represent the passive behavior of SMCs and collagen fibers. Separate symmetric boundary value problems were established for the thoracic and abdominal aortas, each incorporating a damage zone for needle insertion to inject fluids. They also explored a second scenario that examined the behavior of multiple intramural mucoid fluid pools. The constitutive parameters for both models were derived from the literature [694,695]. Additionally, they conducted multiphoton imaging on mouse aortas to identify the elastin structure in the descending and abdominal aortas. As shown in Fig. 49(a), the descending aorta displayed large circular fenestrations, which were less common in the suprarenal abdominal aorta. Instead, the abdominal aorta exhibited numerous elongated and relatively thick radial elastin structures. These struts traversed the intralamellar space between SMCs, connecting adjacent elastic lamellae, and were absent in the descending aorta. The multiphoton imaging results informed their subsequent computational analyses. The key computational findings are as follows. First, in a single intramural injection into the thoracic aorta, increased injection volume resulted in two deformation regimes: wall elevation above the pool, followed by tearing that began at a critical pressure. The tear expanded only in-plane, initially in the axial direction and later in both axial and circumferential directions, which is consistent with experimental observations. Notably, they discovered that the square of tearing pressure was directly proportional to tissue stiffness and critical tearing energy and inversely proportional to the square root of the torn area. Second, during a single intramural injection into the abdominal aorta, the elastic lamellae stretched and the radial struts initially bent without tearing. However, at a critical pressure, a strut tore, causing a sudden pressure drop and subsequent pressure build-up. The pressure–volume curve displayed S-shaped steps, analogous to experimental findings. In addition, they conducted simulations comparing a weaker and stronger intraluminal media without struts. Finally, they modeled a case with multiple fluid injections. As the pools grew closer together, they began to coalesce after an initial period of growth, with tears occurring at lower pressures.

Motivated by Ban et al. [693], who demonstrated that the spatial distribution of structurally significant interlamellar struts can affect the likelihood of dissection, Yin et al. [697] developed a data-driven surrogate model to predict the delamination process for various strut distributions. This model utilized an operator-regression neural network [698] to enhance predictive accuracy. The neural network successfully predicted the pressure–volume curves of injected fluids and the damage progression field of the wall across diverse strut distributions. The results suggested that this composite branch-trunk neural network effectively captured the underlying functional relationship between unique microstructures and their mechanical properties. Furthermore, the incorporation of the current damage field allowed accurate characterization of characteristic pressure drops. To generate *in silico* data, a total of 2,100 phase-field solutions were created, with 1,900 used for training and 200 for testing. According to them, this was the first attempt to predict dissection progression and mechanical behavior in a heterogeneous aortic wall using scientific machine learning.

In a later study, Ban et al. [696] applied the previously developed phase-field model to simulate intra-lamellar tearing induced by intramural ink injections into the medial layer. They implemented two models: a phase-field model and a simplified model. For the constitutive model, they employed a Fung-type model with four collagen fiber families, using material parameters from the literature for both healthy human descending thoracic aorta [699] and diseased aorta [683]. Two main findings were reported. First, a power-law relationship was observed between pressure and key geometric and mechanical factors such as surface area, wall stiffness, and tearing energy. Second, it was found that focal regions of weakening or stiffening can influence the direction of tear propagation, such that the tear moves toward either the lumen or adventitia. To validate their findings, they replicated experiments from Roach et al. [600] with two needle sizes on 18 descending thoracic aortas of similar length (56±3mm) harvested from pigs. The results were consistent both qualitatively and quantitatively with those of Roach et al. [600], as shown in Fig. 49(b). Specifically, they discovered that the pressure at which tearing initiates depends nonlinearly on the pressure in the true lumen, wall stiffness, critical tearing energy, tear area, and axial stretch. A power-law relation emerged for the pressure at which tear propagation begins, with respect to wall stiffness, critical tearing energy, and the inverse of the initial area. Additionally, axial stretch often diminishes in vessels prone to dissection. At tear nucleation sites, normal static blood pressure is insufficient to separate layers and advance the tear, but the Donnan swelling pressure might be adequate [700]. As volume increases, the influence of Donnan swelling pressure diminishes and blood pressure becomes more significant, enabling tear propagation. This is due to the inverse relationship between pressure of tear resistance and increase in torn area. Finally, the study found that tear propagation follows oblique weak regions, meaning that localized areas of weakness or strength can affect the propagation direction. This can cause a tear to shift toward the lumen or adventitia, resulting in a fatal rupture or additional fenestration. A strong oblique inhomogeneity could also deflect the tear propagation path.

In 2022, FitzGibbon et al. [701] analyzed the mixed-mode behavior of exponentially softening path-dependent cohesive zone formulations. A new non-potential path-dependent cohesive zone model was used to simulate aortic dissection in two examples. The artery was modeled using a hyperelastic, anisotropic bilinear fiber model [702], with cohesive zone material parameters obtained from previous experimental-computational studies of mode II dissection [703]. In the first example, the risk of tear propagation under luminal pressure was investigated. To this end, dissection propagation was simulated in an artery with a pre-existing radial notch tear. A cohesive zone was prescribed in the circumferential plane around the tear, and a supra-physiological load of 500mmHg and 10% axial stretch were applied. The crack initiated at 275mmHg and continued to propagate, with final crack growth reaching 5.75mm at a pressure of 500mmHg; no crack propagation occurred below 275mmHg. Initiation and propagation occurred in pure mode II due to compressive tractions at the medial interface caused by hypertensive blood pressure. A 50% reduction in interface strength led to tear propagation under typical hypertensive blood pressure at 190mmHg. In the second example, the risk of further false lumen propagation in an artery with a pre-existing patent false lumen was investigated by coupling hemodynamics with solid mechanics. Contrary to other studies, the recent study suggested that the eventual formation of a false lumen, following mode II initiation, will result in mixed-mode conditions at the crack tip.

In their research, Li et al. [704] developed an idealized model of an artery, described as a single-layered, thick-walled axisymmetric tube with an axisymmetric tear. They accounted for residual stresses by employing the opening angle method. Both the lumen of the artery and the interior of the dissection were assumed to be under uniform, constant pressure, simulating conditions analogous to blood pressure. The HGO model was utilized to describe the material behavior. The progression of the tear was interpreted as an incremental deformation within the tube. The study found that changes such as a decrease in the fiber angle, a decrease in axial pre-stretch, and an increase in the opening angle contributed to the dilatation of the dissection. Furthermore, a gradual increase in pressure in the lumen and the dissection was identified as a factor leading to the dilatation of the dissection.

By applying a combination of experimental procedures, constitutive modeling, and computer simulations, Zhang et al. [705] examined the 3D residual stress field of the aortic wall. The authors performed biaxial tests and opening angle experiments [706] on healthy porcine aortic samples. A key facet of their research was the development of a stress-driven tissue growth model [707], which was built upon the decomposition of the total deformation gradient into two distinct parts: the elastic deformation and the volumetric growth. This model was developed with a particular focus on accounting for the stress deviation between the current and target stress in the *in vivo* stress distribution. After verifying their model, they pursued further validation of their methodology and examined the buckling behavior of the dissection flap using experimental and computational approaches. For this purpose, circular tears of varying sizes were manually induced on three ring-shaped samples of the same porcine aortas. They observed an intriguing inward buckling of the dissection flap. Their numerical simulations were successful in recreating the buckling behavior of the dissection flap of the dissected aorta, which was influenced by the residual stress. This study was pioneering in its inclusion of residual stresses in relation to aortic dissection, and it presented a correlation between the buckling phenomenon and the configuration of the flap. However, the extent of these residual stresses remains difficult to determine accurately without considering *in vivo* boundary conditions. As discussed earlier, the configuration of the dissection flap might be related to the pressure difference between the true and false lumina. Further investigations are necessary to ascertain the degree to which residual stresses influence the configuration, particularly as these stresses could potentially be modified in pathological tissue.

Subsequently, Han et al. [708] investigated tear propagation in aortic dissections using a model of an idealized cylindrical, tube-shaped, bilayer thick aortic wall. Their study focused on the influences of geometrical parameters, loading conditions, and residual stresses on tear propagation. They employed a cohesive zone method with a bilinear traction-separation law to model in-plane dissection propagation between concentrically distributed elastic lamellae in the aorta. The study used the incompressible HGO model to describe the material behavior of the aortic wall, which was modeled as three concentric layers and calibrated using experimental data [707]. Additionally, the model was calibrated for three modes of interfacial damage in the media, based on experimental findings [433,454]. A critical aspect of this model is the use of cohesive elements, with the force–displacement relationship characterized as a linear function of relative separation, and a power law defining the failure criteria. Two primary computational models were used in this study. The first model focused on calibration, incorporating delamination and shear tests to identify material parameters. In the second model, an idealized cylindrical, tube-shaped, bilayer thick wall of the aorta was subjected to axial stretch and inner pressure. This model featured a cohesive zone in the media and an initial tear characterized by its angle, axial length, and depth ratio. They investigated the influence of the initial tear, axial stretch, and residual stress on the critical pressure for in-plane dissection propagation. The outcomes of the model demonstrated that deeper and larger cracks tend to correspond to a lower critical pressure. Key discoveries from the study include, *inter alia*: (i) greater axial stretch results in a reduction in critical pressure, (ii) higher residual stress is accompanied by an increase in critical pressure, (iii) variations in pressure between the true and false lumina significantly affect the critical pressure, and (vi) the altered geometry of the tear influences the critical pressure.

Gheysen et al. [709] conducted an uncertainty quantification study on the wall thickness and stiffness of an idealized dissected aorta. They developed a model that assumed a cylindrical geometry with two layers, the media and the adventitia, and two tears, using length and diameter values from the literature of the normal descending aorta [710–712]. The circumferential false lumen size was determined from pre-operative CT scans of a patient-specific case with type B dissection [713], with the healthy pre-dissection diameter estimated based on the curvature of the true lumen wall. Similar to their previous study [714], they employed the GOH model with parameters fitted to uniaxial and biaxial experimental data of the descending aorta [240,715]. They ensured physiological behavior by coupling the parameters of the media and adventitia to pulse wave velocity and applying an axial stretch with a pre-stressing algorithm [716]. Uncertainties associated with unknown total wall thickness, medial thickness, flap thickness, and pulse wave velocity were analyzed. The resulting 4D input space was sampled using Latin hypercube sampling, assuming a uniform probability distribution for the four independent input parameters. A total of 300 simulations were performed and a surrogate model was developed based on these simulations. Due to the limited output of 300 simulations, a Gaussian process regression model was trained to account for uncertainty in the surrogate model itself rather than using a regular neural network. Based on the output distribution of 10,000 surrogate model simulations, different displacement measures and the maximum principal Cauchy stress at a specific flap position were evaluated. The parameter-specific uncertainty analysis indicated that the modeled material stiffness significantly influences the deformation of the dissected wall, while the relative thickness of the dissected flap is the most critical factor affecting the wall stress. The large ranges of uncertainty and the varying impact of often unknown wall stiffness and thickness parameters highlight the need for caution when interpreting the outcomes of dissected wall models.

In conclusion, the cohesive zone model, along with the phase-field approach and the XFEM, has been widely used to investigate dissection propagation. Key findings involve the detection of helically-shaped propagation patterns, with the tear generally not extending through the adventitial layer. Consequently, the findings suggested that the propagation remains predominantly in-plane. Moreover, the studies indicated that factors such as dissection flap stretching due to luminal expansion or motion can significantly influence delamination, with flap stiffness playing a crucial role in flap deformation. Additionally, flap thickness is identified as the most critical factor affecting wall stress, which may also impacts delamination. Given the challenges in accurately measuring flap thickness and its variable stiffness, caution is needed when interpreting the outcomes. Moreover, the size and depth of the intimal tear have a significant influence on its propagation. Observations showed that tear propagation tends to follow oblique regions of wall weakness, suggesting that localized areas of strength or vulnerability may influence the propagation direction. This underscores the need to consider inhomogeneities within the aortic wall when modeling, which will be discussed in the subsequent section.

### 5.3. Focal areas of medial degeneration

Modeling the focal areas of medial degeneration or material inhomogeneities in the pathological wall is essential, as recently emphasized [717]. Yet it is frequently overlooked. To date, research has primarily focused on modeling the accumulation of intraluminal GAGs and their effects on local stress distribution, among a few other studies.

The accumulation of GAGs in the aortic wall can result in considerable stress concentrations and an intra-lamellar pressure known as Donnan swelling pressure. This hypothesis, proposed by Humphrey et al. [103], served as the motivation for the computational study conducted by Roccabianca et al. [700]. For demonstration purposes, they modeled a rectangular section of the aortic media containing a central GAG inclusion and applied various stress ratios in the axial, circumferential, and radial directions. Notably, the radial direction consistently experienced compressive stress. The aortic media was modeled as a homogenized material, utilizing the neo–Hookean model to represent the matrix material and the HGO model with four fiber families to account for axial and circumferential alignment of collagen fibers. The GAG inclusion was represented using a modified neo–Hookean model, which exhibited minimal tensile stiffness, and incorporated osmotic loading of both the media and the inclusion. The osmotic loading in the inclusion was assumed to be significantly higher. To ensure accurate computational outcomes, the constitutive parameters were fitted to experimental data [699]. The finite element analysis revealed that both circumferential and axial stresses around the periphery of the inclusions were locally increased. Furthermore, radial stress exhibited a sharp increase directly in front of the tip of the inclusion along its major axis. The study thereby confirmed that spatial heterogeneities could contribute to the propagation of aortic dissection.

In a subsequent study, Roccabianca et al. [79] employed two distinct models to address a similar boundary value issue: a mechanistic, mixture-based finite element model and a phenomenological, continuum-based semi-analytical model. In this investigation, they represented the aortic wall as a two-layered cylinder with specified osmotic pressures for each layer, demonstrating that typical GAG accumulations within the aortic media might be equally crucial in both supporting and detecting mechanical loads. Analyzing the computational results, they proposed that a pathological increase in these GAG aggregates could potentially cause excessive pressure in the intralamellar units, which could lead to tension in the radially-oriented elastic fibers that connect SMCs to the elastic laminae. This tension could then trigger mechanosensing in SMCs.

Based on the same hypothesis but using a different approach, Rausch et al. [718] integrated the isotropic continuum damage theory with the smooth particle hydrodynamics (SPH) approach to simulate damage and failure in soft tissues. In short, unlike in mesh-based methods such as the FEM, SPH is a meshfree Lagrangian computational method used to simulate continuum media such as solid mechanics and fluid flows. It solves differential equations of motion using particles, smoothing quantities of interest. SPH can handle large deformations without remeshing and, as shown in this study, can account for macroscopic damage and gross failure. However, it is computationally expensive and defining boundary conditions is often a challenge. To model failure, the developed algorithm automatically identified damaged particles and separated the spatial domain. Constitutive relations for the aortic wall were chosen according to the HGO model, as it accounted for the orientation of collagen fibers. The computational findings of the SPH approach were then compared with predictions from uniaxial and biaxial extension tests. To conclude, the SPH approach was effectively validated against experimental data from a peeling test and failure under clamped uniaxial extension [433,668].

Building on the previously developed SPH approach, Ahmadzadeh et al. [719] later investigated the potential role of accumulated GAGs in initiating and propagating intra-lamellar delamination. To accomplish this, they expanded the SPH approach to model growth, coalescence, and propagation of GAGs. Additionally, they introduced the active contribution of SMCs, modeled using a purely mechanistic approach, and incorporated osmotic swelling pressure into the model. Consistent with previous studies [79,700], a modified neo–Hookean model was employed to capture the ability of GAGs to absorb compressive stresses. To assess the stress distribution surrounding GAG pools, an idealized 2D cylindrical aorta was created, and four different GAG pool sizes were compared. For the constitutive parameters, experimental data from murine aortas were used [720,721]. The computational findings revealed substantial intramural stress concentrations and a shift from typically compressive to tensile stress in the radial direction near the tip of pooled GAGs, which aligns with earlier studies [79,700]. Furthermore, the activation of SMCs in the media can partially shield the wall from swelling-induced damage and reduce the circumferential stress in the adventitia.

**Fig. 50.**
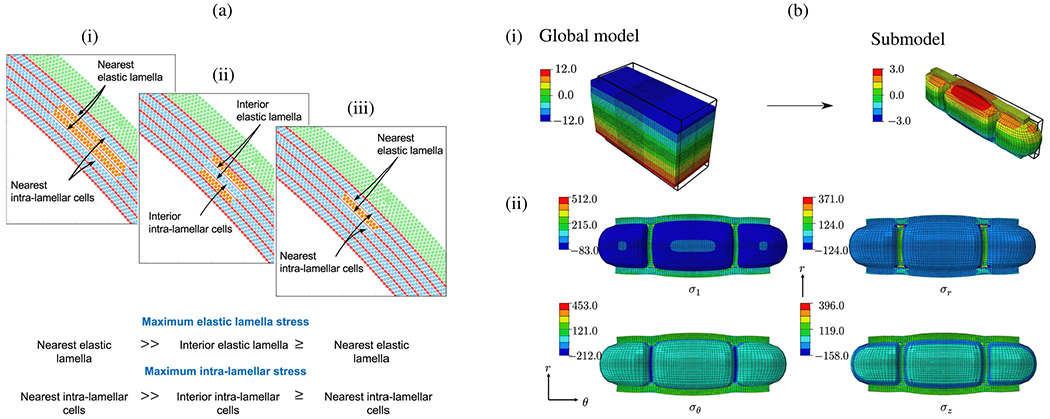
(a) Analysis using an SPH aortic model illustrating stress field variations based on different configurations: (i) a single pool of apoptotic cells; (ii) two separate pools; and (iii) two interconnected pools of apoptotic cells replaced by GAGs. The combined stress impact on elastic lamellae and intra-lamellar cells is most pronounced near the merged pools, suggesting an elevated risk of lamellar rupture and SMC impairment in this scenario. It is noteworthy that the symbol (≥) illustrates how the stress concentrations around a single pool and two separate pools are comparatively close to each other at lower pressures (102mmHg), but the separate pools induce greater stress in their vicinity at elevated pressures (184mmHg). (b) (i) Visualization of the deformation in both the global model and its submodel (in *μ*m) and (ii) observed results after the swelling phase with maximum principal Cauchy stresses and those in radial, circumferential, and axial directions (in kPa). Noticeable stress concentrations can be seen at the boundaries between the different materials. Consequently, the struts of the elastic fibers limit the radial expansion of the GAGs/PGs. Conversely, in the circumferential and axial directions, the primary resistance to swelling arises from the adjacent, relatively softer inter-lamellar material, which includes both the GAGs/PGs and the SMCs. Figure (a) is adapted from Ahmadzadeh et al. [722] (licensed under CC-BY 4.0), while (b) is from Liu et al. [724] (licensed under CC-BY 4.0).

Ahmadzadeh et al. [722] subsequently refined their approach by developing a multilayered model, as shown in Fig. 50(a), encompassing both the media and the adventitia, to investigate the stress field resulting from the disruption of individual elastic lamellae, loss of SMC contractility, and GAG production within an intra-lamellar space. The material response of collagen fibers was incorporated using four families of collagen fibers, while elastic fibers were explicitly modeled with an isotropic neo–Hookean model. The boundary value problem comprised approximately six nearly concentric elastic lamellae and five distinct layers of SMCs subject to pre-stretch. The constitutive parameters of the model were determined based on literature data [720–723]. Through computational analysis, they confirmed that GAGs could elevate radial stresses beyond a certain threshold, potentially leading to intra-lamellar delamination. Interestingly, as shown in Fig. 50(a), the stress effect on the elastic lamellae and intra-lamellar cells is most pronounced near the merged pools, suggesting an elevated risk of lamellar rupture. In addition, GAGs can grow circumferentially and even coalesce with other pools.

Continuing to model the effects of GAG accumulation, Liu et al. [724] adopted a distinct approach to model the swelling pressure of GAGs in the aortic media. They employed a strain-energy function with weighted fractions of individual constituents, including SMCs, collagen fibers, elastic fibers, and GAGs/PGs. The swelling pressure was modeled using swelling polymer theory. The constitutive behavior of collagen and elastic fibers was represented using the HGO model, while GAGs/PGs were modeled with an isotropic contribution, incorporating the osmotic or Donnan swelling pressure. Constitutive parameters were drawn from various sources [700,719,725–727] and three numerical examples were established. First, a thick-walled cylinder with two layers was generated, with inner pressure applied. The results indicated that the current fixed charge density is inversely proportional to the volume ratio and proportional to the reference fixed charge density. Second, seven lamellar units were modeled with fiber struts orthogonal to the lamellae. This submodel (Fig. 50(b)) was employed to represent the detailed microstructure using a finer mesh. The swelling pressure of GAGs generated failure stresses comparable in magnitude to the failure stress observed in tensile struts. Additionally, stress concentrations were identified between interlamellar struts, see Fig. 50(b). Finally, a comparison between pressurization with and without preconditioning was conducted, revealing that residual stresses contribute to increased load-bearing capacity with respect to aortic dissection.

A general method for modeling local inhomogeneities in the aortic wall was presented by Ranftl et al. [717], who illustrated its application by simulating the random distribution of degraded elastic fibers in aortic dissection. They applied a previously developed constitutive model [685] to a simple uniaxial extension test example. A beta random field was used to model the distribution of elastic fiber degradation, and uncertainties were determined using a Bayesian encoder-decoder network. Although the approach was demonstrated using a relatively simple numerical example and a detailed discussion of the theoretical framework, the computational method can also be employed for 3D simulations and patient-specific settings to model local inhomogeneities in both healthy and pathological aortic walls. This approach can be particularly effective because pathological changes in the microstructure typically occur on a local scale.

The outside-in progression hypothesis (see Section 3.2.1) suggests that dysfunction of the vasa vasorum may significantly reduce nutrient supply to the arterial wall, leading to a hypoxic state [438]. This hypoxic condition could initiate ischemic events, potentially catalyzing inflammatory responses and ultimately triggering arterial dissection. To address this, Soleimani et al. [728] introduced a mathematical framework for modeling the progression of atherosclerosis and arterial wall dissection, which accounts for potential tears or ruptures in the arterial wall due to microinjuries within the vasa vasorum. The proposed coupled multiphysical framework was described using finite strain theory and formulated within a 3D continuum framework. From a methodological standpoint, it postulates that nutrient transport within the arterial wall can be described by the classic diffusion–reaction equation. Additionally, the model applied a phase-field approach to represent inflammation, using a binary indicator to depict the inflammation status, as introduced in a previous study [729]. Subsequently, it connected the inflammatory state to the mechanics to model tissue overgrowth. To then model the rupture of the arterial wall, the model again used the phase-field approach. The developed framework was finally applied to selected numerical examples. For this purpose, material and geometrical parameters were collected from the literature. The first numerical example considered only atherosclerosis and illustrated the thickening of the arterial walls due to vasa vasorum malfunction. The inflammatory processes were governed by the nutrient gradient. Another numerical example, similar to the first but incorporating the damage mechanism, showed that a rupture occurs when a specific fracture threshold is violated. In this context, the intramural hematoma arised mainly from blood leakage from the vasa vasorum, which subsequently catalyzed the progression of the rupture. The results indicated pronounced stress concentrations at the rupture sites, with dissections progressing in both circumferential and axial directions.

In summary, most of the studies presented have examined pooled GAGs in depth. The growth and coalescence of GAGs can result in considerable stress concentration surrounding the pooling areas. In contrast, Ranftl et al. [717] proposed a general approach for modeling local material inhomogeneities, illustrated through the example of local elastic fiber degradation, which has the potential to be applied to patient-specific geometries. In addition, the role of vasa vasorum dysfunction in focal medial degeneration and subsequent arterial dissection has recently been investigated [728]. It is particularly important to consider local material or geometrical inhomogeneities resulting from medial degeneration, especially as they are likely to result in aortic dissection or fatal rupture of the wall. However, they can be difficult to identify in each patient’s aorta and may vary depending on the aortic region. For example, regions susceptible to abnormal WSSs or wall stresses could be susceptible to the development of medial foci of degeneration. A random field approach could thus be a viable solution to address this issue. Other researchers have employed a similar approach to patient-specific geometries, as demonstrated by its application to aortic aneurysms [730] or normal aortas [731].

### 5.4. Growth and remodeling

To date, only two *in silico* studies have delved into modeling growth and remodeling in aortic dissection, each addressing several critical aspects detailed in Sections 3.1 and 3.2. These approaches are particularly noteworthy because growth and remodeling are of significant interest given the distinctive and complex nature of aortic dissection. While it shares similarities with diseases like aortic aneurysms, notably in terms of dilatation, aortic dissection distinctively results in two lumina separated by a dissection flap, along with a newly formed outer wall of the false lumen. These characteristics are particularly relevant during the transition from the acute to the chronic phase and can only be properly understood when considered in the context of a growth and remodeling framework. To this end, the studies discussed below use the theory of homogenized constrained mixture [732], a special case of the constrained mixture theory [733].

**Fig. 51.**
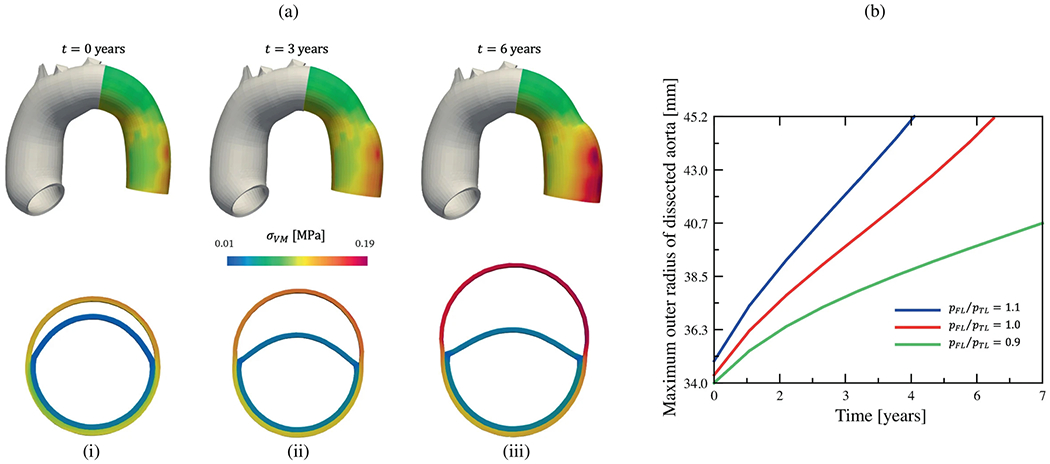
(a) Illustration of patient-specific results of an aortic dissection demonstrating both the geometric alterations and the corresponding von Mises stress changes at three selected time points over a six-year period following a chronic type B dissection. It also shows the dilatation of the circumferential cross section at the site of maximum dissection. These changes are depicted at three distinct time points: (i) at the onset, (ii) after three years, and (iii) after six years of growth and remodeling. (b) Impact of the luminal pressure ratio on aortic dilatation, depicting the temporal evolution of the maximum outer diameter of the patient-specific dissected artery over a period of seven years. Figure is adapted from Zhang et al. [734] with permission from Springer Nature.

In their pioneering computational study, Zhang et al. [734] focused on the role of growth and remodeling in false lumen dilatation following chronic type B dissection. To explore this, they developed a 3D finite element model of growth and remodeling for aortic dissection. This model integrates principles from mechanobiology of the aorta, formulated based on the homogenized constrained mixture theory, see [732,735]. In brief, the homogenized mixture comprises a matrix with a network of elastic fibers, passive reinforcements represented by collagen fiber families in various directions, and active reinforcements accounting for the contractility of SMCs oriented in the circumferential direction. Subsequent model applications represented the aortic wall, including the adventitia and media, connected by elastic springs with high stiffness to represent a healthy aortic wall in a homeostatic state. The false lumen was created by breaking interfacial elastic springs in a predefined region between the media and adventitia. Initially, the model was applied to idealized aortic geometries with cylindrical and toric shapes to validate its feasibility and efficiency. It was then applied to a patient-specific geometry of a thoracic aorta, obtained from [736,737], and demonstrated its potential for more complex and clinically relevant applications, as shown in Fig. 51(a). In each circumferential segment of the respective model, the length of interfacial springs to be broken was predefined, as this study did not primarily focus on the initial tear formation or the subsequent tear progression. The findings showed that growth and remodeling naturally trigger false lumen dilatation after dissection with the aim of restoring stress equilibrium. The so-called gain parameter, which is related to collagen fiber growth and remodeling, plays a pivotal role in the stability of aortic dilatation. A small gain parameter might provoke excessive false lumen dilatation, while a large gain parameter helps restore a stable state of the artery during the chronic phase. Other important mechanobiology-related parameters include the circumferential length of the dissection and the pressure in the false lumen, both of which can significantly impact the stability of the false lumen dilatation. The results suggested that both a wide tear and elevated false lumen pressure tend to promote an unstable development of false lumen dilatation, as depicted in Fig. 51(b).

**Fig. 52.**
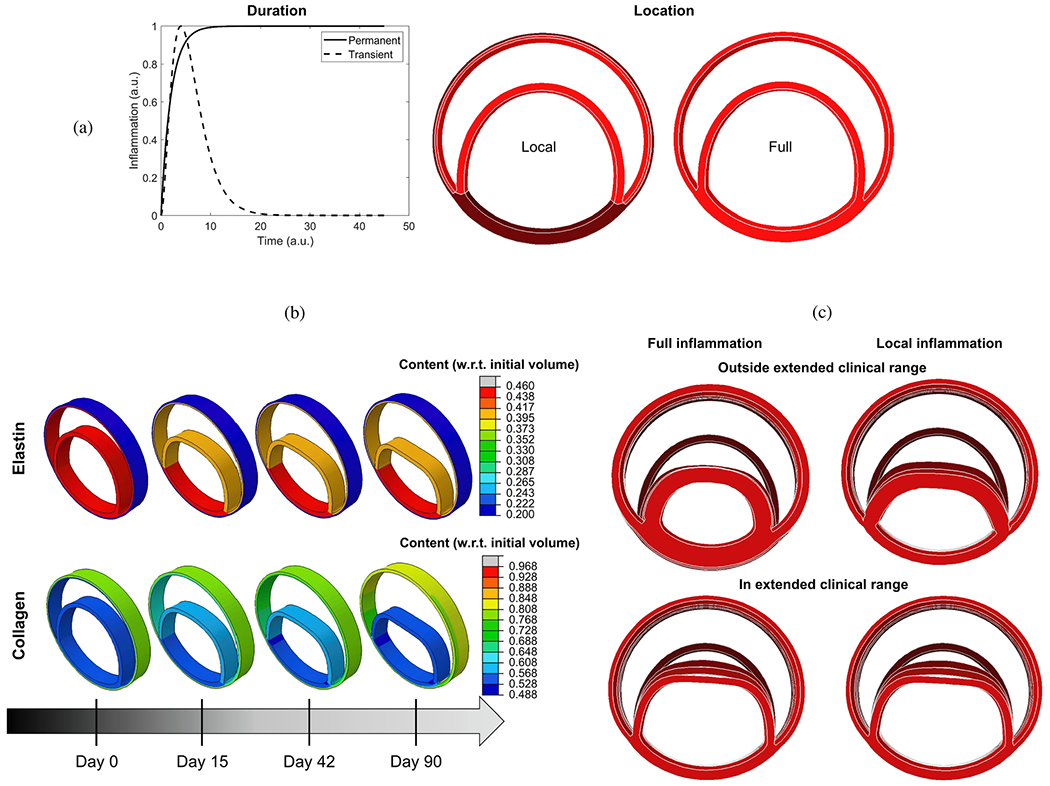
(a) Schematic overview of the inflammatory pattern (permanent or transient) and the location (local or full) indicated in red color. (b) Representative evolution of the elastin and collagen content over time with a local and transient inflammatory pattern, with content expressed as a fraction of the initial volume. (c) Predicted geometrical changes over time for samples with a transient inflammatory pattern applied over different locations (local and full) that resulted in thickening rates outside and within the extended clinical range. The deformed configurations at day 0, 15, 42, and 90 are shown in an increasingly bright shade of red color. Figure is adapted from Gheysen et al. [714] (licensed under CC-BY 4.0).

A second recent study that focused on the growth and remodeling of a dissected aortic wall and integrated the framework of homogenized constrained mixture theory [732] was published by Gheysen et al. [714]. Their study was motivated by the finding that the presence of inflammatory cells in the dissection suggests a role of inflammation in the thickening process of the dissected flap, see Section 3.2.3 and [483,738,739]. Intriguingly, this remodeling occurs even when the stress state matches the homeostatic level, a concept introduced by Latorre and Humphrey [721] in the context of severe inflammatory responses that cause maladaptation of arterial walls. To this end, they created an idealized model of a dissected wall to simulate the production and degradation of elastin and collagen under stress and inflammation-related conditions, see Fig. 52. The model assumed that the aortic wall remodels collagen to restore homeostasis by sensing differences between the current stress state and a homeostatic one [733]. The study used an idealized geometry of a dissected aorta section derived from CT imaging [609], applying an axial stretch, a pre-stressing algorithm, and an inner pressure of 80mmHg in both lumina. The acute material behavior of the medial and adventitial layers of the dissected wall was modeled with an HGO model that encompassed two non-dispersed fiber families. Notably, parameters were chosen based on pulse wave velocity, a method presented in [740]. The growth and remodeling framework used to model the transition from acuity to the chronic phase considered both transient and permanent effects of inflammation on the wall (Fig. 52(a)). Moreover, they applied this framework to all layers (full model) and to only the dissection flap and the outer wall of the false lumen (local model). They compared diameter dilatation, flap thickening, and microstructural shifts over a 90-day period. For the growth and remodeling framework, they implemented 30 growth steps, each spanning three days, to reflect the 90-day period of growth and remodeling. Due to the lack of established optimal growth and remodeling parameters for aortic dissections, they utilized a Latin hypercube sampling with 1,000 parameter combinations, evaluating four inflammatory patterns: transient and permanent inflammation, and a full and a local model. The results indicated a decrease in elastin and in most cases an increase in collagen in the dissection flap microstructure (Fig. 52(b)). The transient inflammatory pattern was particularly effective in replicating clinically observed rates of dissection flap thickening, diameter expansion, and microstructural adaptations (Fig. 52(c)). It is important to highlight that this study did not aim to reveal the ground-truth growth and remodeling mechanisms and corresponding parameters in aortic dissections. It simply supported the assumptions that transient inflammation potentially plays an essential role in tissue growth and remodeling of aortic dissections. Furthermore, it is important to note that despite the limitations mentioned in their study, they did not consider the pressure difference between the lumina and the effect of locally altered WSSs on endothelial mechanosensing. For example, as we will show later in Sections 6 and 7, locally altered WSSs were associated with the characteristic flow jet. These aspects are likely to have a significant impact on the process leading to growth and remodeling.

In summary, it is important to emphasize the significance of studying growth and remodeling particularly in chronic type B dissection, as demonstrated by these preliminary studies. Although there is room for improvement in various areas, these studies establish a promising foundation for future investigations into false lumen dilatation and other growth and remodeling-related processes.

### 5.5. Key findings and publication index

To summarize this section, we present the publication index of multiscale material models for damage and failure in Table 6. Due to the large number of studies available, only a selected range was discussed; however, this table covers an even broader range of studies. Essential information about the modeling approaches and the objectives of each study is included. Finally, the most important findings are listed conclusively.

- The direction of dissection progression generally follows the microstructure of the aorta and the direction of maximum stiffness, which coincides with the alignment of collagen fibers. This often results in a helical pattern.
- Both radially-directed collagen and elastic fibers appear to contribute to the delamination strength of the aortic media. The delamination process follows an avalanche-like failure pattern that results from the local buildup of strain energy and then leads to cascade-like failure of the distributed interlamellar fibers. Additionally, the delamination strength is influenced by the inherent microstructure of the aorta, which varies depending on its location along the vessel.
- Accumulation of GAGs through growth and coalescence can lead to significant stress concentration around the areas of pooling. Moreover, local inhomogeneities such as pooled GAGs or focal areas of medial degradation can influence the direction of propagation to either the lumen or the adventitia, regardless of whether they represent weaknesses or strengths. The propagation could then follow the oblique region of these local inhomogeneities.
- In vessels prone to dissection, axial stretch often decreases. Therefore, the presence of axial stretch could provide potential protection against dissection. Additionally, residual stress could play a role in both dissection propagation and the configuration and extent of the dissection flap. However, the exact nature of this role is not yet fully understood.
- The mean and maximum systolic aortic pressure along with the pulsating frequency are the most important factors for dissection propagation. In this context, Donnan swelling pressure plays a critical role in the initiation phase.
- Tear nucleation sites typically experience normal static blood pressure, which is usually too low to separate the layers and extend the tear. In these instances, Donnan swelling pressure might reach the threshold required to initiate separation. However, as the volume of the tear increases, the significance of Donnan swelling pressure diminishes, making blood pressure a more dominant factor. If it becomes sufficiently high, it can cause the tear to propagate further.
- The deposition of collagen fibers, the circumferential length of the outer wall of the false lumen, and the pressure within the false lumen may play a crucial role in the stability of aortic dilatation. Conditions such as low fiber deposition, a wide tear, and elevated pressure in the false lumen tend to favor false lumen dilatation.
- Comparative analysis between transient and permanent inflammation in aortic dissection layers shows that computational results favor a transient inflammatory pattern, especially when comparing the growth rates of dissection flap thickness and aortic diameter with those observed in clinical studies.

## 6. *In silico* hemodynamic models

Due to its complex nature, the vascular system in health and disease is initially modeled using core components that focus on single functions such as arterial fluid dynamics, wall mechanics, and even thrombus formation. Each core model requires thorough mathematical analysis and efficient numerical methods. These core models are then integrated into comprehensive, coupled models to represent significant parts or the entire system, which requires appropriate coupling conditions and innovative numerical strategies for stable and efficient solutions.

Clinical data, essential yet challenging for vascular system models, have advanced alongside computational modeling, largely due to improvements in medical imaging. Radiological images such as CT and MRI are crucial for creating computational domains, but geometric reconstruction, especially when modeling aortic dissections, is complex and requires advanced tools. Beyond image acquisition, segmentation, and geometric reconstruction, meshes must be generated, realistic boundary conditions obtained, ideally from *in vivo* data and biological vascular data acquired for various components, such as rheological parameters for the blood or stiffness parameters for the arterial wall. The governing equations for fluid and solid problems must be defined, coupled, and solved efficiently. Each step brings its own challenges, particularly in aortic dissection. Significant inter- and intra-patient variability and uncertainty further complicate model calibration and validation and underline the need for (patho-) physiological modeling of *in vivo* situations. Particularly noteworthy in this context is the pioneering work of Quarteroni and his collegues in the last decades, see, e.g., [744–746], as it provided the basis for the accurate modeling of real physiological conditions of the cardiovascular system.

Despite these challenges, numerous models have successfully addressed aortic dissection using computational fluid dynamics (CFD). These models aim to enhance the understanding of pathological processes and open new avenues for therapeutic planning and the design of implantable devices such as TEVAR or graft deployment. Although the elasticity of the arterial wall and dissection flap are neglected, unlike fluid–structure interaction (FSI) simulations, which are discussed in Section 7, CFD simulations can still provide valuable insights under certain conditions.

CFD simulations can be utilized, for instance, to estimate flow splits between true and false lumina, to predict stresses acting on the vessel interior or constituents of the fluid, or to provide hemodynamic indicators relevant for the quantitative comparison of alternative treatment options or in the context of parameter studies on virtual cohorts. With a particular focus on CFD applied to aortic dissection, the following selected works have significantly contributed to our current understanding of this complex disease, its genesis, possible influencing factors, and treatment options. The following studies can be categorized into four groups: (i) CFD studies that employed simplified geometries; (ii) cross-sectional case studies on patient-specific geometries; (iii) virtual case studies in which the geometry was artificially modified to investigate specific phenomena; and (iv) longitudinal case studies.

### 6.1. CFD studies that used simplified geometries

Initially, CFD studies were conducted to examine the hemodynamics of aortic dissection using simplified geometries, which was often necessitated by limited computational resources, lack of software infrastructure or medical data and suitable tools to process them. However, today, the state-of-the-art approach incorporates patient-specific geometries when necessary, while simplified studies on idealized geometries are employed for specific reasons. A key aspect in this regard is the development of computational technology, motivated by advances in the broad research area of biomedical engineering and computational mechanics, so that complex patient-specific geometries are now within reach.

**Fig. 53.**
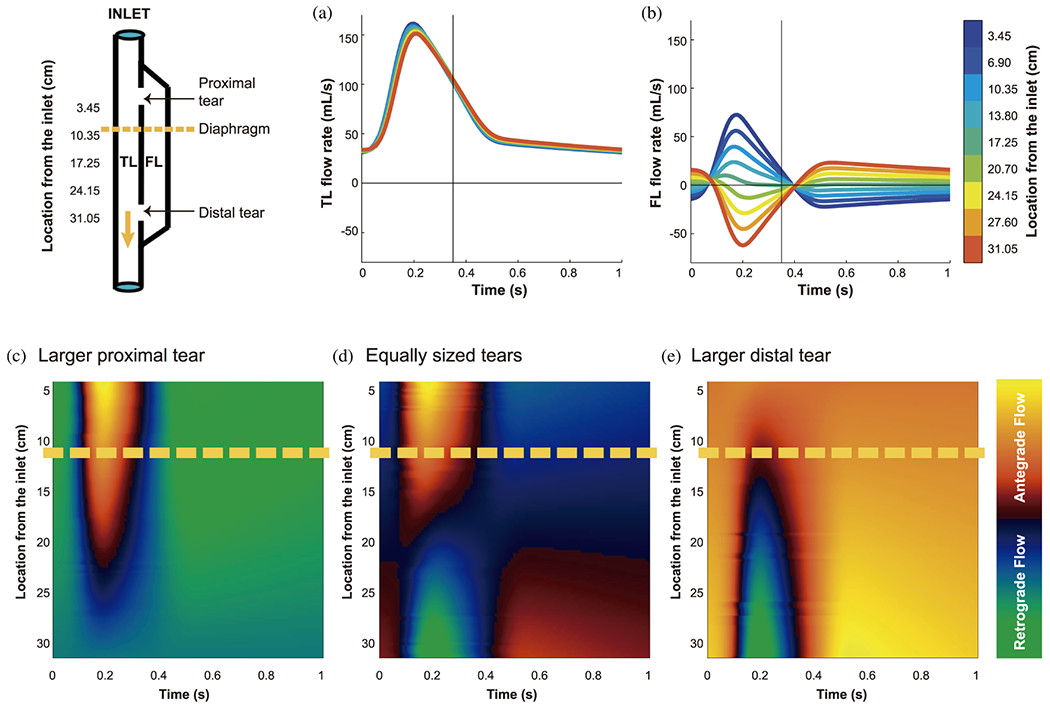
Illustration of a simplified aortic dissection geometry showing the true lumen (‘TL’) and false lumen (‘FL’) connected via two intimal tears. The baseline case has two large intimal tears without visceral side branches, demonstrating the spatial variability of the flow pattern in (a) the true and (b) the false lumen. Positive values indicate antegrade flows, and negative values indicate retrograde flows. The variability in the false lumen flow pattern is illustrated for scenarios with (c) a larger proximal tear, (d) equally sized tears, and (e) a larger distal tear. The yellow dashed lines represent the diaphragm level. Figure is adapted from Rudenick et al. [516] (licensed under CC-BY 4.0).

In their first attempt to quantitatively evaluate the accuracy of a CFD model in predicting intra-luminal pressures in various clinical scenarios for aortic dissections in 2015, Soudah et al. [747] demonstrated the ability of the CFD model to replicate the primary characteristics of an *in vitro* experimental setup accurately [618], as illustrated in Section 4.3. This study was subsequently followed by a series of additional studies. Then, in the work of Rudenick et al. [748], the influence of wall elasticity, also called compliance, on intraluminal hemodynamics in a dissecting aorta was investigated by developing and validating a lumped-parameter model based on the *in vitro* data obtained from a pulsatile hydraulic circuit [618]. The results indicated that setting wall elasticity to physiological values resulted in a decrease in systolic pressure by up to 33% and an increase in diastolic pressure of up to 63%, which consequently resulted in a reduction in pressure wave amplitude by up to 86%. Furthermore, a decrease in stiffness was associated with an increase in multidirectional intraluminal flows and a transition in behavior, with the vessel transforming from two parallel vessels to a vessel with a side-chamber configuration. This study highlighted the critical role that wall elasticity plays in determining intraluminal pressures and flow patterns in aortic dissection. Subsequently, Rudenick et al. [516] investigated how temporal variations in size and spatial distribution of tears, as well as the presence of abdominal side branches emanating from the false lumen, influence the flow pattern. A smaller tear resulted in a more damped and shifted flow profile in the false lumen. In contrast, a large tear increased the retrograde flow proximally and antegrade flow distally. In the true lumen, blood pressure was more likely to be hypertensive with a small tear or hypotensive with a large tear. As shown in Fig. 53, when more than 50% of the total tear area was located proximal, systolic false lumen flow became more monophasic with a decrease in the reverse flow and an increase in diastolic retrograde flow. Conversely, with more than 50% of the tear area located distally, the model showed a clear systolic biphasic pattern with an increase in systolic false lumen reverse flow and predominantly antegrade diastolic flow. Although the aortic wall stiffness did not change the temporal variation of the flow, a stiffer aortic wall increased pulse pressure in the true and false lumina, respectively. In systole, the pressure was then increased, while the maximum pressure decreased in diastole. The results were finally compared with patient flow profiles in the false lumen at the diaphragm level, obtained using PC-MRI. This comparison gave a satisfactory match with maximum flow rate errors below 10%.

Additionally, several studies were published using simplified CFD models, as outlined in the following. In their study, Fan et al. [749] used a simplified CFD model to study the effects of a TEVAR in type B dissection, particularly with regard to the coverage of the proximal entry tear during the post-repair process. The variations in the model also involved the ratio of the area of the false to the true lumen as well as the location and size of the exit tear. The results indicated that a smaller false lumen likely poses less risk because the region of stagnant fluid or complete thrombosis would be larger. Moreover, it has been found that a larger exit tear generally causes blood to move to a larger region. Guan et al. [750] investigated bypass treatment for type B dissection using 2D idealized geometric models and observed decreased blood flow velocity and pressure in the false lumen after treatment. Chitsaz et al. [751] developed five 2D models of aortic dissection to examine the role of length, antegrade propagation, and retrograde propagation. Of the five models, four had a single entry tear and one had a distal exit tear. They discovered that the tearing force was proportional to d*p*/d*t* and the squared dissection length, with pulsatile flow conditions resulting in higher tearing force as steady flow conditions. In fact, a second distal tear significantly reduced the tearing force. Tang et al. [752] developed a simplified 3D model of aortic type B dissection and investigated the effect of false lumen dilatation by creating five models with increasing diameters of the false lumen. Among their findings, they demonstrated that a flow jet through the intimal tear impinges on the outer wall of the false lumen, where the resulting force acting on the false lumen wall increases with increasing diameter. However, it might be difficult to generalize the observations from the developed model because their significance might be limited by the simplified geometric modeling that occludes all supra-aortic vessels, and the choice of outflow boundary conditions that directly determine the ratio of true-to-false lumen flow at the outlet. Ben Ahmed et al. [753] created 14 simplified chronic type B dissection models with straight and curved vessel shapes, varying numbers of proximal and distal tears, and a false lumen location in the inner and outer arch to virtually compare different surgical treatment options. The curvature had no influence on the pressure and flow ratios between true and false lumina. The authors concluded that the combination of proximal and distal tear sizes influences hemodynamics, with larger proximal tears increasing the false lumen pulsatile pressure and larger distal tears decreasing it. Occlusion of the proximal tear resulted in a 54% reduction in pulsatile pressure. Zadrazil et al. [630] investigated type B dissection using particle image velocimetry and CFD simulations in a simplified geometry and used the particle image velocimetry results as validation. The experimental and numerical results using the simplified geometry agreed well, even though the *in silico* model considered different tear sizes and size ratios. It was observed that increasing the exit tear size considerably increased the flow rate in the false lumen, significantly decreased the WSS, and reduced the pressure difference between the false and true lumina. Increasing the entry tear size increased the flow rate through the false lumen, slightly increased the WSS, and raised the pressure difference between the false and the true lumen. The study by Zorilla [754] is also worth mentioning in this context. This study validated the proposed CFD methodology using an *in vitro* reference test case and explored various configurations of dissection flap tears in type B dissection, thereby mimicking blood flow in real-life scenarios.

In addition to the previous studies, Peng et al. [755] conducted a study on acute type B dissection in which a total of 163 patients were examined and compared with a control group of 184 patients. The aim was to understand the morphological differences and the associated changes in blood flow. For this purpose, various parameters were statistically analyzed and idealized CFD models were established for hemodynamic simulations. The results of the study highlighted that the diameters at landmarks of the proximal aortic segment and the lumen volume were significantly larger in the dissection group, suggesting a positive correlation between volume and diameter. Furthermore, the study found that with increasing volume, the kinetic energy, time-averaged WSS (TAWSS), and oscillatory shear index (OSI) at the distal area of the left subclavian artery also increased. This suggested that these factors may contribute to disease progression. On top of that, Qiao et al. [756] developed idealized models of diseased aortas to simulate conditions such as aortic aneurysm, aortic coarctation, and aortic dissection. These models were then employed to virtually deploy a stent-graft introducer sheath commonly used in TEVAR to assess its hemodynamic effects. Comparative analyses from this study indicated that the obstruction caused by the introducer sheath led to an elevated blood flow to the supra-aortic branches. Furthermore, the sheath leads to significantly altered blood viscosity in the aortic arch and the descending aorta. This underscored the importance of using non-Newtonian models for more accurate simulations in scenarios where medical devices might cause reduced circulation, stagnant flow zones, or areas of high shear rates that could lead to shear-thinning.

Lastly, two consecutive studies are presented. In the first, Li et al. [757] constructed population-based 3D models of post-operative thoracic aortic dissection to investigate the hemodynamic consequences of varying tear characteristics. Factors such as the number of tears, the maximum distance between tears, and tear size were taken into account. Their findings revealed that the area of high OSI and activation potential of endothelial cells increased with increasing number and size of tears, although a consistent tear distance lead to a stable low relative residence time (RRT) area. Additionally, they noticed a significantly increased false lumen blood flow. Keeping the number and size of tears constant, increasing the maximum tear distance by 10mm resulted in an enlarged area of low RRT in the false lumen and a higher average pressure difference. Subsequently, Li et al. [758] applied this model to explore the influence of tear parameters on the formation of false lumen thrombosis after TEVAR. Simultaneously, they conducted a retrospective study of acute type B dissection, focusing on the prediction of false lumen thrombosis based on morphological parameters. Their computational findings reflected previous research. In particular, they found that as the number of tears increased, the flow field became more regular and stable, and flow jet was more evident at the most distal exit tear.

In summary, simplified geometries have proven effective to validate methods against experimental models, develop numerical methods, or investigate specific hemodynamic mechanisms in aortic dissection. For instance, a broad understanding of the effects of multiple tears on hemodynamics, which facilitate communication between the true and false lumina, could be developed. While some of these findings, such as the general presence of a pressure difference between the lumina or the occurrence of antegrade and retrograde flow, could be further confirmed using patient-specific models, many must first be adapted to patient-specific geometries to establish a practice-relevant proof of concept.

### 6.2. Cross-sectional case studies

After providing an overview of studies investigating simplified setups, we will now focus on patient-specific geometries. In the context of *in silico* hemodynamic models, this encompasses cross-sectional, virtual, and longitudinal case studies. First, an overview of cross-sectional case studies is provided, which involves the analysis of medical data collected from a representative case at a specific point in time. It therefore provides a snapshot of the subject under investigation with the aim of drawing conclusions or deriving connections from the currently observed patterns.

In 2010, Cheng et al. [759] investigated flow phenomena in a pre-surgical type B dissection using a shear stress transport (SST) turbulence model and neglecting vessel wall motion. A time-scaled, flat flow profile at the inlet and a zero reference pressure were specified as boundary conditions. In the presented case, 80% of the volumetric flow entered the false lumen, causing a high WSS near the flap in the true lumen wall and a locally highly perturbed, possibly turbulent flow with strong recirculations within the false lumen. TAWSS, OSI, and turbulence intensity were measured and a comparison of Newtonian and non-Newtonian fluid models showed rather small differences in the results obtained in terms of absolute value. However, the comparison of laminar and turbulent models showed a significantly increased WSS in the latter case. The highest WSS was reported at the coarctation of the aorta, which is due to the reduced diameter, which may in turn influence the subsequent dilatation and progressive weakening of the affected aorta.

**Fig. 54.**
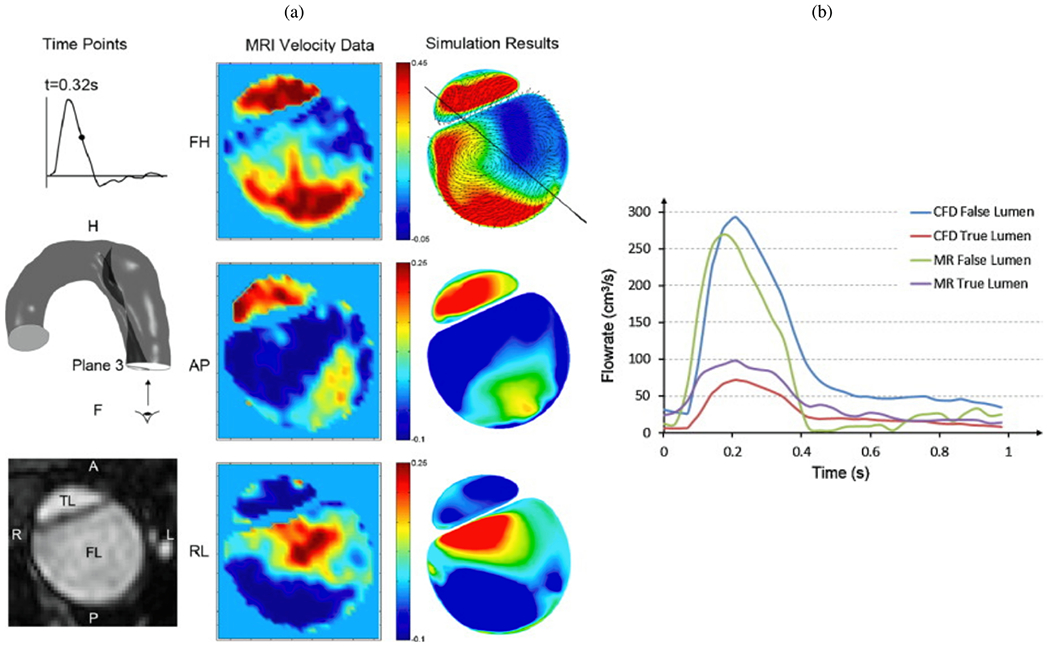
(a) Comparison of the velocity profiles in three directions: inferior–superior (‘IS’), anterior–posterior (‘AP’), and right–left (‘RL’) during the systolic deceleration phase of the cardiac cycle at imaging plane 3. The data includes PC-MRI velocity measurements (in m/s) and corresponding CFD simulation results. Red represents positive velocities, while blue indicates negative velocities. Note that each direction and time point may have different color scales, reflecting the range of velocity values. Secondary velocity vectors are also projected onto the plane for further detail. (b) The diagram shows the flow rate waveforms in the true and false lumina, derived from both CFD simulation results and PC-MRI velocity data at imaging plane 3. Figure is adapted from Cheng et al. [760] with permission from Elsevier.

The computational models were then validated in a follow-up study in 2014. In this study, Cheng et al. [760] compared the computational results with *in vivo* velocity data obtained via PC-MRI (see Figs. 54(a) and (b)). Regions with complex flow patterns such as reversal and recirculation were also qualitatively captured, assuming rigid walls, excluding arch branches, and using simple reference pressures at the outlets. In the CFD simulations, the false lumen flow rate was over-predicted by less than 25%, however, the overall good agreement confirmed the physiological relevance and validity of the computational model for type B dissection with a completely immobile dissection flap.

In their third study, one year later, Cheng et al. [761] then employed these developed numerical tools to compare two groups of four patients treated either medically or surgically via TEVAR. Hemodynamic factors were successfully linked to likely late outcome. High RRT of fluid particles in the false lumen was a strong predictor of false lumen thrombosis and high true-to-false lumen pressure differences, and its location might be associated with aortic dilatation. Furthermore, the entry tear size was positively correlated with the false lumen flow rate, whereas the distance from the arch top to the entry tear was negatively correlated. False lumen patency was promoted by high flow rates in the false lumen and high true-to-false lumen pressure differences, a large entry tear size, and an intimal tear near the aortic arch. Although some simplifications were made in the computational modeling (rigid walls, simplified outlet conditions, etc.) and the small patient cohort limits the statistical relevance, the results presented are of great value and consistent with clinical observations.

Djorovic et al. [762] investigated the hemodynamics in type A dissection using patient-specific geometries derived from CT imaging of two patients. The boundary conditions for the study were consistent with those used by Shang et al. [763], see Section 6.4, including zero outlet pressures, prescribed inflow at the aortic root, and no-slip conditions on the stationary vessel wall. Laminar flow of an incompressible Newtonian fluid was considered using physiological parameters. Similar observations were made: during systole, a high-velocity flow jet forms through the intimal tear, leading to flow disturbances and high WSSs. In addition, the authors reported on the true-to-false lumen pressure differences, from which no further relations could be derived because they were not found to be particularly insightful. Moreover, high shear stresses were observed where velocities occurred, i.e. in the aortic arch, the brachiocephalic trunk, and the exit tear regions. The latter could therefore represent potential zones of further ruptures, according to them. This study highlights the potential of CFD simulations for use in clinical support and personalized medicine. However, to increase significance, larger study cohorts than the two patients considered here are required.

Abazari et al. [764] further extended a previous framework [765,766] and incorporated a generalized Newtonian fluid adopting the Carreau-Yasuda law. Three-element Windkessel models were taken from a similar patient-specific case and the geometry was reconstructed from CT data. The main goal was to verify the effectiveness of anti-hypertensive treatment of type B dissection, modeled by adapting the stroke volume and heart rate accordingly. This *in silico* study investigated the effects of changing the prescribed inflow on TAWSS, OSI, and highly oscillatory low magnitude shear (HOLMES). The clinically observed effectiveness of the drug was confirmed and showed numerically: (i) reduction of high velocity flow jets and high shear rates, (ii) reduction of mean pressure drops, (iii) reduction of TAWSS in the false lumen and thereby a decrease of the risk of false lumen rupture, (iv) reduction of OSI to a moderate level and thereby reduction of the stress endothelial cells were exposed to, (v) that HOLMES indicated regions prone to calcification, and (vi) that the areas that have high OSI and low TAWSS were reduced, which indicated a high risk of rupture, calcifications, or wall thickening. Overall, these hemodynamic indicators, correlating with clinical observations, bear high potential for future use in surgical planning and optimal treatment design.

Chen et al. [767] investigated hemodynamics and the effects of laminar or turbulent flow assumptions and highlighted the importance of proper mesh construction including boundary layers. For this purpose, a patient-specific type B dissection geometry with multiple tears connecting true and false lumina was derived from CTA images. Based on the observation of a stiff dissection flap in the present scenario, the study was limited to pure CFD computations, i.e. rigid aortic walls. No patient flow data were collected, so a representative scale was computed from MRI measurements in 13 individuals, which provided realistic temporal scaling but did not necessarily fit to the specific case. In order to deal with missing data, severe simplifications were made to the boundary conditions – a scenario that has high practical relevance, but also has a strong influence on the computational results. Flow rates were prescribed in the supra-aortic vessels that scaled the inlet profile at the aortic root and produced zero pressure at the three remaining outlets. Since the outlet on the celiac trunk was located at the distal end of the false lumen, a prescribed zero pressure had a direct influence on flow splits and pressure gradients. The relevance of this study lies in the detailed analysis of the flow fields and WSS, the resulting volumetric flow rates, and the true-to-false lumen pressure differences. Great care was taken to investigate numerical aspects such as the influence of the choice of k-*ω* SST turbulence model or laminar flow assumptions and grid sensitivity. According to their study, constructing an improper mesh without boundary layers could dramatically affect the results, leading to a deviation of up to 30% of the solution components at critical points. In fact, the assumption of laminar flow led to reasonable results, with only a 1% difference compared with the solution obtained with a turbulence model, provided that boundary layers are properly accounted for. These two aspects are of particular importance for clinical use.

**Fig. 55.**
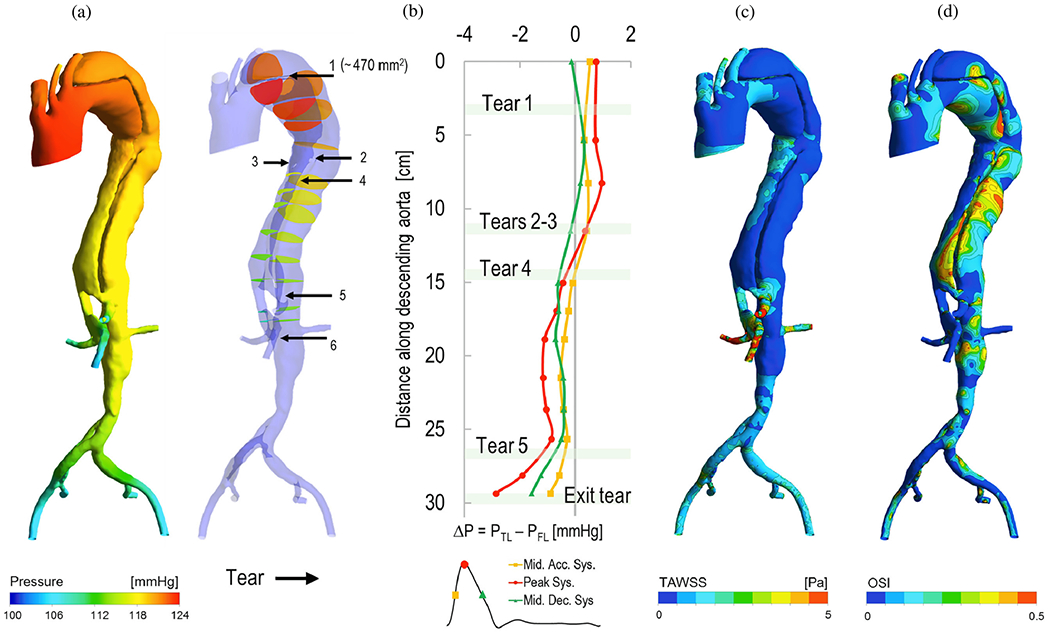
Results show (a) the pressure field at peak systole; (b) the true-to-false lumen pressure difference calculated during three phases of the cardiac cycle: mid-acceleration, peak, and mid-declining phases in systole; (c) TAWSS; and (d) OSI. The hemodynamic indicators show low TAWSS and high OSI in the false lumen, as well as high TAWSS at entry and exit tear positions and in regions of high flow rate in curved lumen regions. Figure is adapted from Bonfanti et al. [770] with permission from Elsevier.

Building on previous studies [768,769], which we will discuss later in Section 7, Bonfanti et al. [770] applied a lumped parameter model [771] to account for the distal vascular system and arterial compliance. This approach allowed them to omit FSI simulations or even flow simulations in instationary domains, while still retaining some compliance in the model. In three cases of type B dissections, only routinely acquired and commonly available clinical data sets were used for parameter estimation and showed good agreement with *in vivo* blood pressure measurements. However, for configurations with highly mobile dissection flaps affecting volumetric flow rates and pressures in true and false lumina, the necessary assumptions may not apply because vessel motion does not only cause a phase shift of the pressure wave but also leads to a strong coupled problem. Of particular note are the pressure field and differences between true and false lumina, as shown in Figs. 55(a) and (b). Substantial true-to-false lumen pressure differences lead to increased dynamic loading on the dissection flap. Hemodynamic parameters such as TAWSS, OSI, displayed in Figs. 55(c) and (d), and RRT computed during post-processing were related to the onset and progression of aortic dissection. These time averages represent vessel WSSs (TAWSS), fluctuation in disturbed flow (OSI), and fluid particle residence time (RRT) correlating with thrombus formation.

**Fig. 56.**
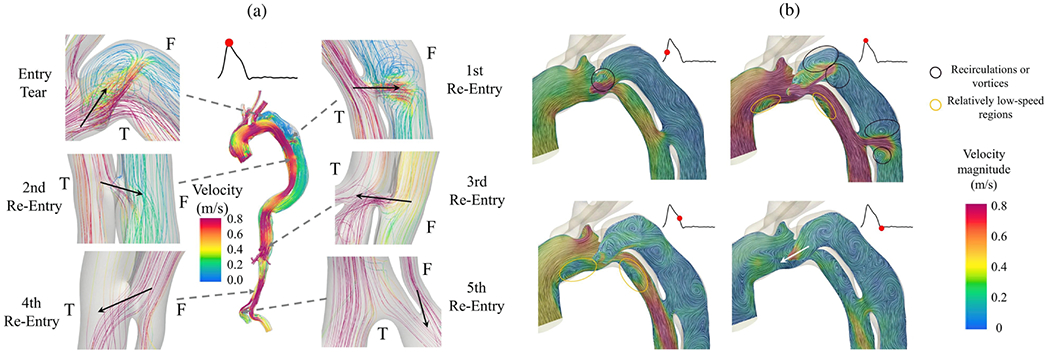
(a) Illustrations depicting the size and locations of six intimal tears, along with 3D velocity streamlines in the tear regions during mid-systole. The locations of the tears are marked with dashed arrows, while the direction of flow at each tear is indicated with solid arrows. (b) Visualization of the flow path within the cut plane of the thoracic aorta, shown using velocity vectors and line integral convolution contours at four time points: early systole, mid-systole, late systole, and early diastole. Blood recirculation and vortices are highlighted with black ovals, and regions with relatively low velocity are outlined with orange ovals. The direction of flow during early diastole is indicated by a white arrow. Figure is adapted from Wang et al. [772] with permission from Elsevier.

In a cross-sectional study by Wang et al. [772], a strong correlation between blood flow measurements from medical imaging and CFD simulations was demonstrated. This study not only validated the accuracy of these methods but also provided visually appealing insights into the hemodynamic characteristics associated with aortic dissection. The CFD model was based on a patient’s geometry post-surgery for type A dissection, which included various repairs such as ascending aorta replacement and aortic root repair, leaving a residual dissection extending from just before the left subclavian artery to the bifurcation of the right common iliac artery. By integrating CTA imaging, 4D-flow MRI, and routine blood pressure measurements, the study found consistent results between the CFD simulations and 4D-flow MRI measurements, with notable differences in diastole and the false lumen, where accurate flow estimations from 4D-flow MRI were challenging to obtain. Fig. 56(a) from the study illustrated the characteristics of six intimal tears in the aorta and how blood flows through these tears during mid-systole. It was observed that blood flowed from the true into the false lumen at the entry and the first two re-entry tears, and then returned to the true lumen at the more distal tears. These flow patterns were visually confirmed using 4D-flow MRI. Additionally, Fig. 56(b) showcased the flow path within the thoracic aorta at various cardiac phases, highlighting the formation of vortices and recirculation zones, particularly in the false lumen and around the graft site. At mid-systole, for instance, recirculation regions developed around the jet flow in the false lumen, particularly at the locations of the tears. In the thoracic aorta, two slower-moving zones were found after flow separation points at the inner corner of the aortic graft and also after the luminal narrowing in the aortic arch at the site of the distal anastomosis of the graft. This detailed visualization underscored the complex and dynamic nature of blood flow in the presence of aortic dissection, providing valuable information for future patient-specific CFD simulations.

Armour et al. [773] adopted a similar computational setup, as introduced by Xu et al. [774], which is described later in Section 6.4. A total of five type B dissection patients were included in this cross-sectional case study. CTA images, 4D-flow MRI data, and invasive Doppler-wire pressure measurements were collected. Inflow profiles were constructed from the 4D-flow MRI data and the pressure measurements taken at multiple locations during TEVAR were used to fit three-element Windkessel models. To account for the laminar, incompressible flow of a non-Newtonian fluid, the Quemada model was considered with parameters taken from the literature. They conducted grid convergence studies for all five cases, which featured a large variety in the morphology of true and false lumina. In all cases considered, the CFD models predicted velocity fields and thus flow splits well. The computational approach led to higher average velocities due to the neglected compliance, while the flow jet in the entry tear region was still underestimated. The Doppler-wire pressure measurements were in reasonable agreement with the numerical results, but errors were noted in both the absolute systolic and diastolic values. These deviations could be due to the assumption of a fixed grid, which typically results in increased systolic pressure and decreased diastolic pressure. The model assumptions therefore influenced the simulation results in the expected manner and motivate investigations considering vessel deformation. In general, there was good agreement between the measurement data and the CFD simulation results.

Another study on aortic dissection using CFD was performed by Kimura et al. [775]. A total of 19 patients were selected, seven of whom had type B dissection and 12 patients were classified as type non-A non-B dissection. In brief again, type non-A non-B dissection is a condition where an intimal tear is located beyond the ascending aorta. This type of dissection is confined to the aortic arch or can be described as a retrograde dissection originating from the descending aorta, extending into the arch, and stopping before reaching the ascending aorta. CTA data from each patient were used to construct computational models that prescribed non-patient-specific but realistic inflow rates, and Windkessel models tuned to achieve physiological pressure levels. They focused on the hemodynamic and morphological differences between type B and type non-A non-B dissection and compared true-to-false lumen pressure differences, intimal entry tear size, and WSSs between the groups. They reported that the median entry tear size was larger in the non-A non-B group, leading to increased false lumen perfusion. Furthermore, the proportion of cases in which the false lumen pressure was higher than the true lumen pressure increased significantly in early, middle, and late systole, while no relevant differences occurred in WSS. Therefore, they concluded that the increased systolic pressure in the false lumen, possibly caused by the increased entry tear size, could be associated with a complicated course and promote unstable conditions. Hemodynamic analysis may provide additional guidance for treatment planning in this regard, but larger study cohorts are required to further validate these results.

In their recent study, Moretti et al. [776] investigated flow patterns in the false lumen of three aortic dissections in the chronic phase: one fully perfused, one partially thrombosed, and one fully thrombosed. These flow patterns were compared with those observed in a healthy aorta. They reconstructed 3D geometries of the aorta using CTA and developed CFD models for analysis. The study revealed that partially thrombosed dissection and fully perfused dissection had the highest pressure and TAWSS values, which are significant risk factors for aortic degeneration and potential wall rupture. Furthermore, all cases demonstrated poor perfusion in the branch vessels connected to the false lumen. Additionally, both the true and false lumina showed elevated turbulence accompanied by critical stagnation points compared with the healthy aorta. However, a generalization of these results is difficult due to the distinct anatomical differences observed in the selected dissection cases, particularly regarding the location and extent of the false lumen. In addition, the determination of the parameters for the three-element Windkessel models, which were first determined for the healthy case and then applied to the dissection cases, could have a significant impact on the results.

**Fig. 57.**
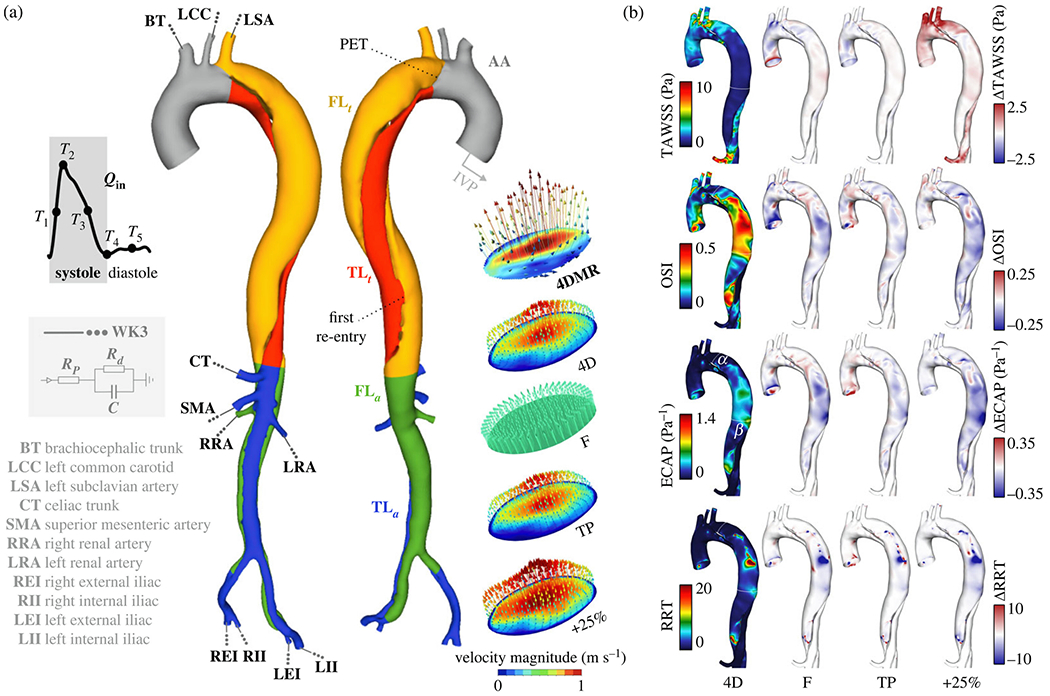
(a) Computational domain along with schematic representations of the inlet and outlet boundary conditions. The lower right corner shows the velocity magnitude contours and vectors captured at peak systole for each patient-specific inlet velocity profile: 3D three-component inlet velocity profile (‘3D’) derived from 4D-flow MRI data (‘4DMR’) with flow-matched flat (‘F’), through-plane profiles (‘TP’) and scaled inlet velocity profile (‘+25%’). Differently colored regions within the aortic geometry indicate the areas targeted for helicity analysis. (b) The left side shows contours for TAWSS, OSI, endothelial cell activation potential (‘ECAP’), and RRT in the thoracic aorta for the 3D case. The right side shows different contours compared with other cases. It is important to note that the contour ranges for TAWSS, endothelial cell activation potential, and RRT have been clipped to improve visual clarity. Contours vary between 25 and 50% of the boundaries set by the 3D contours. Figure is adapted from Stokes et al. [777] (licensed under CC-BY 4.0).

The influence of inflow velocity profiles on hemodynamics was examined by Stokes et al. [777], specifically focusing on the relationships between flow helicity, oscillatory WSS, and false lumen dilatation. These relationships were demonstrated using 4D-flow MRI. A patient-specific geometry of a type B dissection was employed and CFD simulations were conducted. Blood was modeled as an incompressible, non-Newtonian fluid using the Tomaiuolo formulation [778] of the Carreau-Yasuda viscosity model. Additionally, a k-*ω* SST Reynolds-averaged turbulence model was utilized, setting a low turbulence intensity (1%) at non-wall boundaries. To evaluate different inflow profiles, the study compared simulations using the gold-standard 3D three-component inlet velocity profile derived from 4D-flow MRI data with flow-matched flat and through-plane profiles. These alternative profiles are commonly used when 4D-flow MRI is not available, see Fig. 57(a). To estimate the effects of imaging errors in 4D-flow MRI on flow solutions, the 3D three-component inlet velocity profile was scaled by a 25% increase, reflecting the extent of imaging error observed in previous studies. The outlets were modeled using three-element Windkessel models and the mean outlet flow rates for each major branch were obtained from 4D-flow MRI data. It is noteworthy that the measurement uncertainties in 4D-flow MRI were higher in these smaller branches than in the aorta. The study compared velocity, pressure, helicity, and WSS distributions. Helicity was used to identify streamwise vortex structures by quantifying the local orientation of velocity and vorticity vectors. Interestingly, since helical structures in the aorta have a larger scale than the fluid boundary layer, these features can be measured more reliably with 4D-flow MRI than with WSS measurements. Moreover, the study included a proper orthogonal decomposition analysis of the velocity field to assess how each inlet condition affects the coherent structures within the flow and the energy distribution. The results indicated that secondary inlet flows significantly impact the distribution of oscillatory shear and helicity throughout the false lumen. Even the 3D three-component inlet velocity profile showed notable differences in helicity and oscillatory shear when adjusted to account for imaging errors. The locations of the most significant false luminal growth are shown in Fig. 57(b) and marked by the sections *α* and *β* in the 3D energy contribution to the average pressure plot. In the thoracic region, TAWSS was highest and was located immediately distal to the primary entry tear, near section *α*. Minimal variations in TAWSS occured in the remainder of the false lumen. TAWSS was elevated in the +25% case, particularly in the aortic branches due to the increased flow volume. Regions with low TAWSS, which were directly related to false luminal growth in aortic dissections, generally coincided with areas experiencing higher oscillatory shear. There is general consensus that a 3D three-component inlet velocity profile should be used where available, with through-plane profiles serving as the next best option. While a flat inlet velocity profile adequately captured the magnitude and trends of the circumferentially-averaged WSS metrics, the local values of OSI, energy contribution to average pressure, and RRT in the most dilated regions showed substantial differences when using the 3D three-component inlet velocity profile. Increasing the inlet velocity components by 25% to account for the underestimation by 4D-flow MRI affected the average magnitude of the differences in the WSS and helicity metrics. Overall, the quality of inlet velocity conditions in simulations of type B dissection can profoundly influence their clinical relevance.

**Fig. 58.**
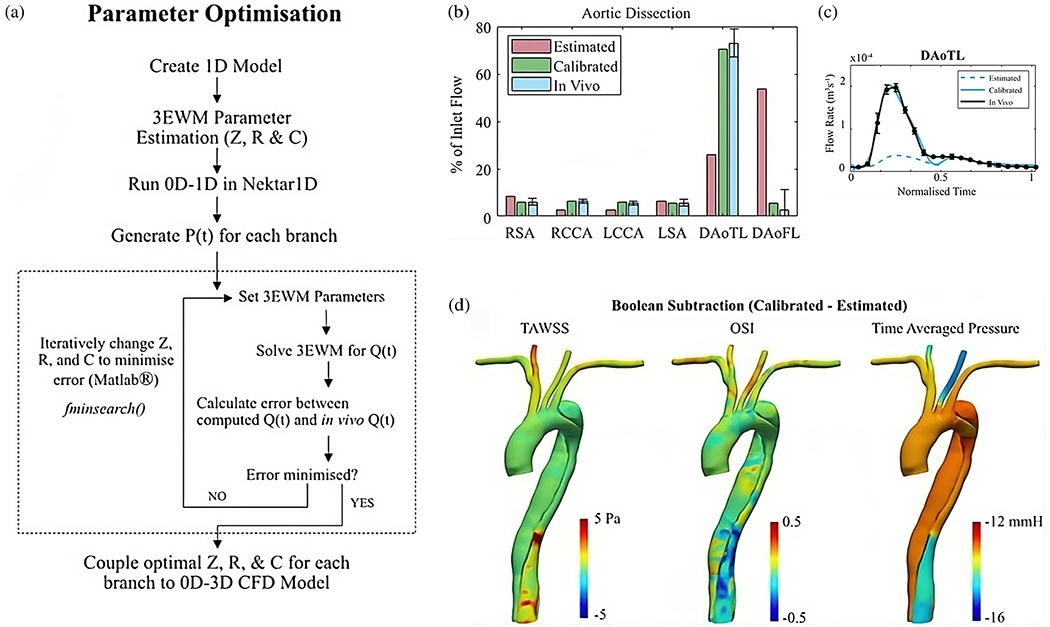
(a) Flowchart illustrating the calibration process for impedance (‘Z’), resistance (‘R’), and compliance (‘C’) within the boundary conditions of the three-element Windkessel model (‘3EWM’). To configure a 0D-3D CFD model, each branch (or outlet) is integrated with a three-element Windkessel model. At the inlet, a flow waveform generated by 4D-flow MRI is converted into a parabolic velocity profile. The equation for the discretized three-element Windkessel model describes the relationship between pressure (‘P’) and flow (‘Q’) at each branch, where ‘n’ represents the current iteration. (b) Blood flow perfusion distribution in the entire 0D–3D CFD model before and after calibration compared with *in vivo* 4D-flow MRI data and (c) a representative flow waveform at the aortic dissection outlet of the true lumen, calculated via the 0D three-element Windkessel model before (dashed colored curves) and after (solid colored curves) calibration compared with *in vivo* 4D-flow MRI data (error bars represent mean standard deviation). (d) Boolean difference (calibrated – estimated) in TAWSS, OSI, and time-averaged pressure within the dissected aorta determined by 0D-3D CFD simulation. Figure is adapted from Black et al. [779] (licensed under CC-BY 4.0).

A final study to summarize is by Black et al. [779], who introduced a novel reduced-order computational framework for the iterative flow-based calibration of three-element Windkessel model parameters. This development has significant implications for the creation of patient-specific boundary conditions, especially in healthy individuals and those with type B dissection. The study utilizes boundary conditions identified from retrospective VENC 4D-flow MRI data at both the inlet and outlet of an incompressible Reynolds-averaged Navier–Stokes approach in conjunction with a standard k-*ω* SST turbulence model. Through a fully-integrated 0D-3D numerical framework, the calibration process aimed to identify a combination of parameters at each aortic outlet that best matches the clinical 4D-flow MRI data, which required an iterative process, see Fig. 58(a). Initial estimates for this process came from 1D modeling, which provided rudimentary pressure and flow waveforms at each outlet of the thoracic aorta; these initial Windkessel parameters then formed the starting point for the iterative calibration process. Remarkably, this automated calibration only required about 3.5 minutes per outlet and yielded physiologically relevant and highly accurate flow waveforms. A comparison between the estimated, calibrated, and *in vivo* data is shown in Fig. 58(b) for the percentage of inlet flow at the outlets and in Fig. 58(c) for the flow rate at a representative outlet. Moreover, Fig. 58(c) illustrates the Boolean difference between the calibrated and estimated hemodynamic parameters. When these calibrated boundary conditions were prescribed, the computed metrics related to near-wall hemodynamics and perfusion distribution were found to be consistent with existing clinical measurements and the literature, confirming the physiological relevance of the model. Therefore, this calibration technique can be applied in clinical settings where outlet flow rates are measured using methods such as 4D-flow MRI or ultrasound. It enables efficient determination of patient-specific boundary conditions for CFD simulations, provided the reduced-order model accurately captures the key flow characteristics.

The presented works and the summarized findings demonstrate that cross-sectional case studies can provide valuable insights into the hemodynamics of various patient cases. For example, they compared cases with stent and without stents as well as cases with varying numbers, locations, and sizes of intimal tears. These comparisons allowed them to observe the significant effect of fenestrations on hemodynamics. Furthermore, case studies facilitated the investigation of the influence of boundary conditions such as inflow and outflow conditions and emphasized their considerable impact on the validation results. Even small changes can lead to substantial deviations in the computational outcome. Overall, the model validation results demonstrated that the results of the *in silico* hemodynamic models agree well with *in vivo* data. Virtual case studies in which patient-specific geometries are virtually altered to examine the effects of certain morphological phenomena are a valuable extension of cross-sectional case studies, particularly when longitudinal data are not available.

### 6.3. Virtual case studies

Based on cross-sectional case studies, invaluable insights into the underlying principles of cardiovascular diseases can be obtained, taking into account different geometric configurations, hemodynamic scenarios, or different problem parameters. In the context of an individual patient, virtual case studies have proven to be an excellent tool for identifying underlying mechanisms. They have demonstrated their applicability in clinical support and treatment planning within personalized medicine. For example, the patient-specific geometry can be modified by adding, occluding, or altering the size of intimal tears. This approach can be used to examine specific geometric features or explore possibilities for surgical interventions, even without longitudinal data.

In this sense, Karmonik et al. [780] studied the influence of entry and exit tear occlusion on hemodynamic parameters using fixed-grid CFD simulations, virtually adapting the geometry to model TEVAR, false lumen thrombosis, or fenestrations. In this particular case, changes in flow patterns and blood pressure were reported. Covering the exit tear resulted in a 300Pa increase in false lumen pressure. In contrast, virtual TEVAR resulted in negligible differences in true-to-false lumen pressure. Additionally, complete removal of the dissection flap reduced the pressure in the combined lumen from an initial value of 2,400 to 800Pa. Despite the limitations of this study due to zero reference pressure applied to various branching vessels, resulting in flow splitting and possibly non-physiological pressure levels, the general observations still transfer to more realistic setups with correct pressure scaling and a deformable dissection flap.

The *in-silico* setup of Chen et al. [767], which was further improved in their follow-up study [781], has physiologically meaningful (instationary) outlet pressures. The latter study compared a four-year follow-up study modeling reduced stroke volume for the treatment of hypertension with multiple virtual endovascular repair variants. Two main findings from this analysis confirmed that (i) treatment of hypertension significantly reduces both the pressure drop between true and false lumina and the WSS and (ii) TEVAR stabilizes the true lumen, which can significantly reduce false lumen perfusion, which in turn leads to stagnant flow in the false lumen. TEVAR can thus trigger thrombosis, but this further depends on the size and number of tears in the dissection flap, with complete closure of all tears resulting in fully stagnant flow, giving the highest probability of false lumen thrombosis and the greatest potential for full aortic remodeling.

Wan Ab Naim et al. [782] investigated the effect of introducing additional fenestrations in a patient-specific model of type B dissection. Flow distribution, pressures in the true and false lumina, and WSS distributions were investigated in three configurations: the original geometry with a single entry and exit tear, and two variants where a fenestration with a diameter of either 10 or 16mm was virtually added to the CTA-based geometry. The laminar flow of an incompressible Newtonian fluid was considered, driven by a flat inflow profile with temporal scaling from the literature. Prescribed flow fractions through the supra-aortic outlets were used to prescribe velocity profiles and pressure scaling was achieved via pulsatile pressure at the distal outlet. The artificially introduced entry tear connecting true and false lumina led to increased fluid exchange between the lumina, effectively reducing the true-to-false lumen pressure difference while only increasing TAWSS locally around the additional fenestration. They argue that reducing pressure in the false lumen, particularly in the distal region, reduces the risk of false lumen dilatation. The TAWSS was found not to differ significantly at the model scale. However, locally increased values could trigger the onset of dissection and should therefore be avoided. Therefore, hemodynamic investigations on patient-specific models might be used to determine optimal stent placement to reduce the true-to-false lumen pressure difference and to minimize the risk for further rupture.

**Fig. 59.**
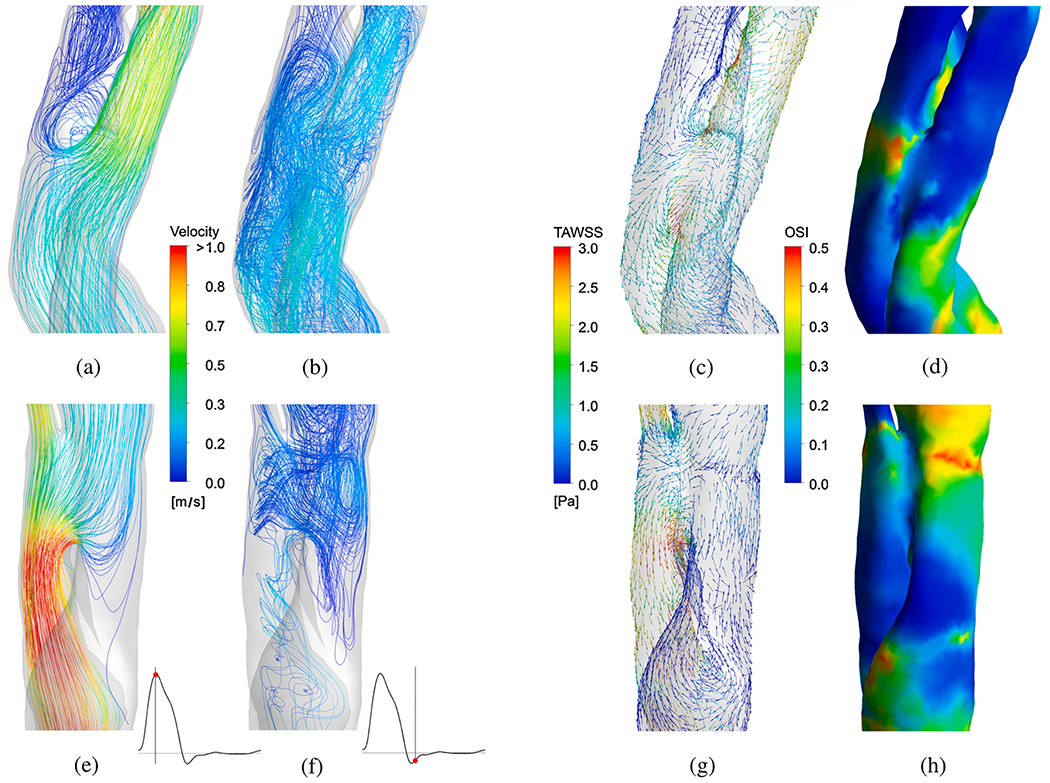
Flow characteristics around the entry (top row) and exit (bottom row) tears during two points of the cardiac cycle: (a, e) streamlines at peak systole, (b, f) streamlines at mid-diastole, (c, g) orientation of TAWSS vectors, and (d, h) OSI related to the preferential flow direction. Figure is adapted from Alimohammadi et al. [765] with permission from Elsevier.

Alimohammadi et al. [765] investigated a case of type B dissection in which a patient-specific geometry was reconstructed from CT images. Blood was modeled as an incompressible Newtonian fluid under laminar flow conditions in a fixed lumen. The main contribution of this work is an algorithm for tuning parameters of three-element Windkessel models to clinical data, which, depending on the number of outlets involved, tremendously facilitates tuning in patient-specific scenarios. The vessel wall and dissection flap were assumed to be fixed, but within this limitation the pressure wave propagation was accurately captured. The flow characteristics in the areas surrounding the entry and exit tears at peak systole and mid-diastole were rigorously analyzed. Fig. 59 shows the streamlines, the TAWSS, and the OSI. The developed methodology was further employed in patient-specific virtual stent implantation [766], comparing single and double stent-graft strategies based on velocity, pressure, energy loss, and WSS distribution. The computational model identified significant differences in the surgical procedures: a double stent-graft strategy further reduced the true-to-false lumen pressure difference and the energy loss was reduced by 40% compared with the single stent-graft variant.

Dillon-Murphy et al. [783] studied the effects of dissection flap morphology, i.e. the number and size of intimal tears or even the complete removal of the dissection flap on the hemodynamics. A detailed model of type B dissection was reconstructed from CTA data, imposing boundary conditions and optimizing Windkessel models based on 2D PC-MRI measurements. As for the numerical treatment, the flow of an incompressible Newtonian fluid was controlled either by a prescribed parabolic inflow profile informed by 2D PC-MRI data or by a lumped parameter model that indirectly enforces a target inflow waveform. Using the latter approach, the authors demonstrated that the required left ventricular stroke work was increased by 14% for the present configuration. The reduced true lumen cross-section in the dissected case resulted in increased velocities in the true lumen and a drastic increase in the TAWSS from 5 to 100dynes/cm^2^ in the entry tear region. The computational model was validated using 4D PC-MRI, which demonstrated good agreement of mean flows. The maximum error observed was 7.6% across three measurement planes, with an *in vivo* flow fraction of 79% compared with an *in silico* flow fraction of 76% in the dissected aorta. This method was also used to identify additional fenestrations connecting the true and false lumina. They demonstrated that neglecting additional fenestrations can lead to severe alterations of the true-to-false lumen pressure difference (18%) and false luminal flow (increased by 200%), which are important indicators for late complications and have a major impact on false lumen thrombosis. Although wall motion was not considered in this study and patient-specific pressure data were not available, the comprehensive analysis presented here has contributed significantly to our understanding of aortic dissection. This research emphasizes the importance of accurately capturing the size and location of entry and exit tears together with possible additional fenestrations.

Shi et al. [784] compared various configurations of type A dissection by virtually placing a dissection flap in an aorta reconstructed from CTA data. Each dissection flap had a single entry and exit tear, while the ratio of the entry-to-exit tear area was set to 1.0 and 2.0, respectively. The location of the tear also varied from the proximal end of the false lumen in the ascending arch to the apex of the arch, resulting in a total of four different configurations. The average pressure and flow data were considered to account for physiological flow conditions and drive the laminar flow of an incompressible Newtonian fluid. They reported that flow rates and TAWSS in the false lumen were higher with a larger exit tear and that the entry tear closer to the aortic root also increased TAWSS and pressure in the false lumen. Additionally, in cases where the proximal end of the false lumen did not have an entry tear, high residence times were observed, potentially triggering thrombosis in the region of stagnant flow in the false lumen. Due to the virtual nature of this study, it was able to determine isolated effects of the tear size and position on hemodynamics. The insights gained from this small virtual cohort appear promising for translation to clinics and patient-specific scenarios. However, it remains unclear to what extent the results of this study hold when further variations in geometry, such as tortuosity, radius, and curvature, or different inflow conditions and pressure levels, are taken into account.

In a similar study, Yu et al. [785] created a patient-specific model of type B dissection. They derived a computational model from CTA data that included an entry tear and three fenestrations. However, they did not distinguish between a dominant distal exit tear and fenestrations. An incompressible, laminar flow of a Newtonian fluid was considered and boundary conditions generated from flow data from the literature. Three additional models were constructed by altering the number, size, and location of tears, namely, (i) occluding two fenestrations, (ii) introducing one additional fenestration, and (iii) increasing the entry tear size compared with the original model. They found that pressure and flow rates were different in the true and false lumina for each of the cases, while occluding the entry tear resulted in stagnant flow in the cut-off portion of the false lumen. Additionally, occluding the original entry tear resulted in flow reversal at the re-entry tears and introduction of additional tears resulted in increased false lumen perfusion. They concluded that CFD modeling may be a useful tool for predicting hemodynamic changes in aortic dissection, providing additional guidance for treatment planning and for non-invasive serial monitoring.

When branch vessels were located near the tears, careful attention to blood supply is crucial. In these cases, multiple overlapping uncovered stents are strategically employed to address the challenge. Dai et al. [786] performed a virtual case study on a CTA-based geometry in which they compared various treatment strategies and evaluated their effectiveness in false lumen thrombosis. Simplified stent geometries were considered in the CFD mesh, with the stents having different mesh densities, resulting in different porosity. Flow rates adopted from the literature were used to drive the flow of an incompressible Newtonian fluid with SST turbulence model. Supra-aortic vessels were neglected and the corresponding flow fraction was subtracted from the inflow. This was a reasonable strategy when focusing on the regions distal to the aortic arch. Notably, the use of multiple overlapping uncovered stents and the coverage of branch vessels complicated the application and calibration of Windkessel models. They reported that multiple overlapping of uncovered stents reduced blood flow to the false lumen and resulted in lower WSS. Furthermore, this strategy expanded the true lumen and also stabilized the false lumen, while keeping the blood flow to the branch vessels intact. The combination of standard covered stent-grafts with multiple overlapping uncovered stents where necessary was found to be the most effective option and resulted in the largest region of stagnant flow. In the present scenario, the blood flow in the latter case was reduced to a small region near the right renal artery, which continued to be supplied. This virtual case study thus highlighted how favorable thrombus formation in the false lumen can be achieved by analyzing the hemodynamics using CFD.

In Xiong et al. [787], CFD was used to investigate the hemodynamic effects of what is known as type I hybrid arch repair in a patient suffering from non-A non-B dissection. This procedure consists of repositioning aortic arch vessels on the healthy ascending aorta in addition to TEVAR. To quantify the effectiveness of this alternative procedure, patient-specific geometries were reconstructed from medical images pre-operatively, one week, one month, and one year after the intervention. In addition, the dissection flap was virtually removed to obtain the pre-dissection geometry. In a longitudinal study, true-to-false lumen pressure difference, WSS, and volumetric flow rates were then compared with normal blood flow. Morphological changes led to the return of hemodynamic indicators to baseline, which indicates the effectiveness of the procedure or remodeling of the aorta. Incompressible flow simulations based on a typical volumetric flow rate and tuned Windkessel models showed that entry tear closure regularized the flow pattern in the true lumen, while the flow field in the false lumen became more complicated and even resulted in retrograde flow during diastole in the proximal false lumen. Of course, covering the entry tear led to a large reduction in the volumetric flow rate in the false lumen, so that the overall flow splits had a strongly positive effect. TAWSS was more evenly distributed in the normal range of 10 to 50 dyne/cm^2^s at the one-year follow-up, with less pronounced peak values. Moreover, the region of abnormal WSS in the true lumen progressively decreased, while the WSS in most areas of the false lumen remained below 4dyne/cm^2^. Elevated pressure and WSS were related to the onset of dissection, whereas low magnitude and large variations of the WSS triggered thrombosis, so the risk of further rupture was considered low. Moreover, the patent false lumen exhibited gradual thrombosis at the one-year follow-up, as suggested by the CFD analysis. Hybrid arch repair resulted in smaller true-to-false lumen pressure differences, such that at the one-year follow-up the pressure between the two lumina was nearly equal. The surgical procedure was therefore verified using CFD analysis, whereby the influence of the specific choice of the inflow profile could have a significant impact on the computational results. The artificial vessels were created very close to the aortic root, so that inlet and leaflet motion and the specific form of inflow profile and temporal scale can strongly influence the flow field. Nevertheless, this work demonstrated how longitudinal case studies on hemodynamics can be used to analyze, compare, and potentially optimize surgical interventions on digital twins.

Li et al. [788] also adopted a similar numerical approach to investigate the effects of intimal tears on hemodynamics in five post-TEVAR aortic dissection cases. Subjects were grouped according to the false lumen dilatation that occurred within the initial post-TEVAR and the first follow-up CTA image. For comparison, additional models were generated based on the first post-TEVAR CTA scans by artificially occluding intimal tears. The vessel wall and dissection flap were assumed to be rigid, and a flat inflow profile along with a single identical flow waveform and Windkessel parameters were adopted from the literature for all models. The individual models, including the first post-TEVAR and corresponding variants that virtually occlude the intimal tears, were then compared based on hemodynamic indicators. The true-to-false lumen pressure difference, the related first point of equal cross-sectional and time-averaged pressures in true and false lumina, the first balance point, as it is known, the flow split between true and false lumina, and the RRT were calculated. They found that the shift in the first balance point was more pronounced in the group with enlarged false lumen than in the stable lumen group and therefore could serve as a predictor in clinical practice.

Li et al. [789] investigated the potential triggering mechanisms underlying aortic dissection and conducted a CFD study on 15 type B dissection cases by virtually repairing them, i.e. removing the dissection flap. These repaired cases were analyzed using CFD and compared with 12 control patients using geometric features, flow helicity, and WSS-based indicators. They considered laminar flow of an incompressible Newtonian fluid driven by the same flow waveform. Windkessel models were used for each of the outlets, fitting the parameters to the patient’s admission blood pressure levels via a tuning algorithm. They reported no significant differences in the geometric properties of the groups, while the healthy control cases showed increased contralateral helical blood flow and helicity. The mean normal transverse WSS was significantly (25%) higher in the virtually repaired type B patient cases, while the locations of primary entry tears matched well with regions of high mean normal transverse WSS. Hence, given limited patient data, CFD analysis could therefore already be a useful tool for early detection of high-risk patients. The strategy used to develop virtually repaired geometries assumed that the pre-dissected cross-section can be restored by removing only the dissection flap without changing the radius. In many cases, such a strategy is useful. However, this procedure required a pre-selection of the dissection cases that corresponded to these assumptions. Therefore, it remains unclear whether the study’s conclusions are applicable to cases in which dissection resulted in a significant increase in the volume of the entire vessel.

Wen et al. [790] conducted a comparative study on healthy, dissected, and virtually repaired aortas to identify hemodynamic parameters effective in predicting type B dissection. They considered four indicators based on the WSS, namely the TAWSS, the OSI, the cross flow index (CFI) [791], and the topological shear variation index (TSVI). TAWSS and OSI signal areas of high and high oscillatory shear stress on the vessel wall, while CFI only captures the direction of the WSS but not its magnitude. The TSVI is higher in regions with large variations in endothelial cell contraction and expansion [792,793]. Another three helicity-based indicators were used, namely the locally normalized helicity (LNH), the averaged helicity, and the absolute intensity of the spiral flow [794,795]. CTA images were obtained and corresponding meshes were created for all 16 patients. The flat inflow profile was scaled according to the literature [768], Windkessel models were chosen based on a previous study [796], and blood was modeled as an incompressible, non-Newtonian fluid. The hemodynamic indices between the groups were compared in the descending aortic arch region, where the intimal tear originated in the dissected cases. Statistical analysis showed that OSI and CFI were significantly increased in patients with aortic dissection and that the average helicity also differed significantly between dissected and healthy patient groups. This study suggests that hemodynamic indicators may be more accurate than anatomical characterization in predicting aortic dissection.

The virtual case studies presented have provided valuable insights into the hemodynamics of patients with aortic dissection. This is achieved by virtually altering the number, location, or size of the intimal tears or by removing the dissection flap to obtain a virtual pre-dissection geometry. This has proven beneficial not only in virtually modeling specific treatment scenarios, but also in gaining a better overall understanding of the complex hemodynamics. Moreover, because pre-dissection geometries are rarely available and estimates of pre-dissection geometry from CT imaging are limited [712], such investigations are invaluable for enhancing our understanding. Longitudinal case studies, discussed below, introduce another level of complexity as they necessitate the creation and simulation of multiple patient-specific geometries over time. Because all geometries are based on patient data, they can yield more reliable results.

### 6.4. Longitudinal case studies

Cross-sectional case studies cannot prove causality or determine the timing of events. However, virtual and longitudinal case studies that address these important aspects can provide valuable insights. In the following section, we will present longitudinal case studies that follow individual patients or cohorts over time to investigate the effects of specific factors on various outcomes. Longitudinal studies can be either retrospective, involving the collection of data about past events, or prospective, starting with a baseline assessment and tracking individuals over time, with *in silico* longitudinal case studies generally being retrospective to date.

In 2011, Karmonik et al. [797] presented pre- and post-treatment CFD simulations of a type B dissection case, investigating changes in WSS and pressure to compare the initial configuration with a completely remodeled one-year follow-up. They emphasized that to achieve accurate results, patient-specific geometries and measured boundary conditions have to be provided. Then, the relevant quantities such as WSS and true-to-false lumen pressure differences could be accurately captured in both scenarios. In a follow-up study by Karmonik et al. [798], the validation of the proposed framework for patient-specific aortic dissection was carried out based on the clinical examination of a 45-year-old patient, including a ten-month follow-up examination. Axial through-plane velocity PC-MRI data from sites in the thoracic and abdominal aorta were compared with the obtained results and showed good correlation of major flow patterns in true and false lumina. A comparison of hemodynamic parameters obtained by CFD of a healthy aorta and a chronic aortic dissection case in [446] demonstrated the serious effects of false lumen dilatation. In the dissected case, a reduction of 77% in WSS, a 69% reduction in pressure, and an increased true-to-false lumen pressure difference were noted, along with a reduction in WSS by a factor of three.

In related work, Cheng et al. [799] investigated the effect of aortic and entry tear morphology on flow characteristics and corresponding clinical outcomes in four patients with acute type B dissection. Blood flow in the true and false lumina varied drastically among the subjects considered. The individual geometries included the aorta from the arch to the iliac bifurcation and excluded all secondary vessels. Based on these studies, significant morphological parameters were identified. According to the results obtained, the maximum height and width of the entry tear, as well as the distance between the peak of the aortic arch and the upper edge of the tear, were important parameters that governed the flow field. The size and location of the tear dominated the flow rate in the false lumen and have a major impact on the progression of false lumen thrombosis or the onset of dissection. These findings suggested that patients can be divided into groups based on CFD results. Furthermore, the prediction of flow rates and the risk for onset of dissection appears promising.

**Fig. 60.**
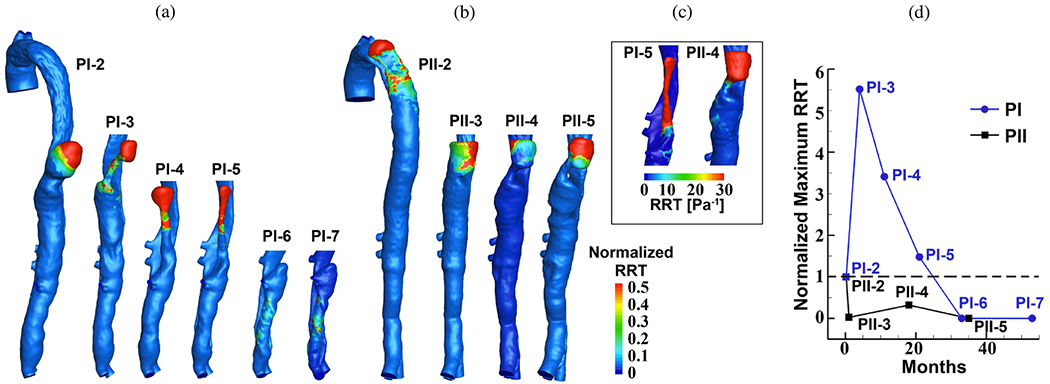
(a, b) Distribution and variation of normalized RRT to its maximum value in two patients with type B dissection post-TEVAR (PI and PII) at multiple follow-ups. (c) The absolute RRT differences at comparable times are similar. (d) Short- to middle-term differences in the normalized maximum RRT of PI-2 and PII-2, indicating significantly elevated values (above one) for several months in PI. This biomarker could therefore be correlated with positive false lumen remodeling. Figure is adapted from Xu et al. [774] with permission from Elsevier.

Xu et al. [774] explored two patient cases with type B dissection treated by TEVAR, denoted as PI and PII and shown in Fig. 60. Only one case showed an enlarged false lumen at follow-up, while the other case remained stable. Patient-specific follow-up models were then developed based on Doppler ultrasound velocimetry and CTA images. Flow rates computed using Doppler ultrasound velocimetry were prescribed assuming flow profiles at the aortic root and supra-aortic vessels. For the remaining outlets, time-dependent mean pressures were adopted from another patient and additionally mapped between the individual stages within the longitudinal case study whenever the computational domain had to be shortened due to missing CTA data. It should be noted that the choice of pressure scaling can have a significant impact on the flow split. Choosing neither patient-specific pressure curves nor resistance-based models with tuned parameters (both of which provide the desired flow splits and pressure values) can lead to unpredictable alterations in the results obtained. However, when comparing the computational results and the Doppler ultrasound velocimetry measurements, a difference of less than 5% in the mean velocities over a cardiac cycle was found at selected points. Therefore, the aforementioned possible consequences of imposing pressure values in different patients did not impact the results excessively within this study. However, in the second case, the false lumen was shortened, causing problems with outflow conditions and potentially drastically affecting the results. Temporal and spatial grid convergence analyses and comparisons with a simulation using the k-*ω* SST turbulence model showed that for an average Reynolds number over a cardiac cycle of about 2,100 and about 2,900 at the ascending aorta assuming laminar flow, a grid with boundary layers and 45 time steps per cardiac cycle resulted in sufficiently small errors compared with the baseline. The flow fields at multiple follow-ups are compared by analyzing the true-to-false lumen pressure differences, RRT, WSS, and OSI. They showed that the true-to-false lumen pressure difference was highly dependent on the size and location of the entry tear, while the largest true-to-false lumen pressure differences correlated with regions of maximum false lumen dilatation. The patient with a patent false lumen still showed a significant true-to-false lumen pressure difference at the final follow-up. Furthermore, TEVAR initially caused an increased true-to-false lumen pressure difference, resulting in dilatation of the false lumen distal to the stent-graft. Thrombus formation in the false lumen was related with TAWSS, OSI, and RRT. The WSS for the patient with patent false lumen remained elevated, while the RRT was higher at the proximal end of the false lumen because the distal ends had additional fenestrations in one case and even an outlet in the second case, as shown in Figs. 60(a) and (b). The RRT distributions differed directly post-TEVAR but are similar at follow-up, leading them to suggest that the difference in RRT at short- to middle-term follow-up could be a potential predictor for false lumen thrombosis, as shown in Fig. 60(d). An increase in RRT after TEVAR signals stagnant flow in the false lumen, a condition that must be maintained for an extended period of time. This was evident from the increasing relative maximum RRT, normalized by the maximum RRT at the first follow-up, required to induce positive false lumen remodeling. In conclusion, this hemodynamic parameter might be clinically applied to predict false lumen thrombosis. This could suggest early re-intervention, long before routinely acquired morphological changes trigger similar measures. However, similar computational studies on larger cohorts are needed to confirm these initial findings and increase the statistical relevance.

**Fig. 61.**
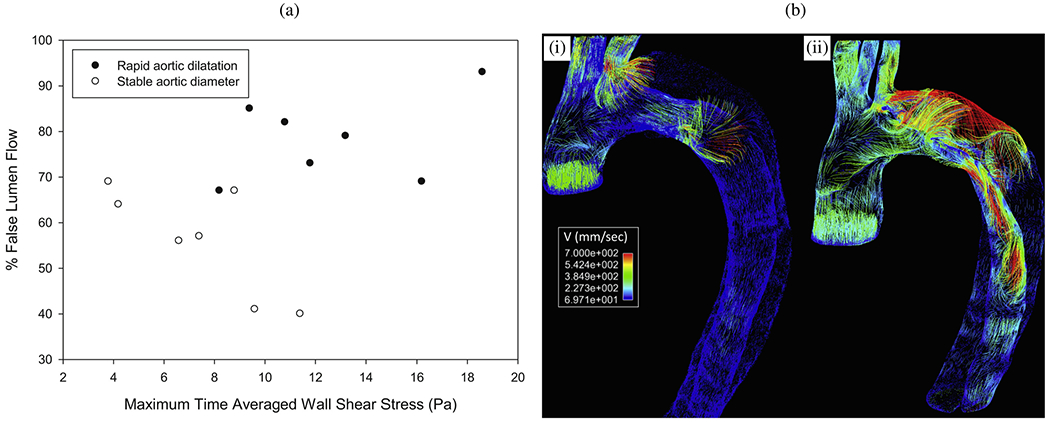
(a) False lumen flow rate and maximum TAWSS for type B dissection cases with stable and rapid growth rates. (b) Typical velocity fields in the intimal tear region: (i) uncomplicated type B dissection and (ii) patient case with rapid growth. Figure is adapted from Shang et al. [763] with permission from Elsevier.

Another longitudinal case study was carried out by Shang et al. [763], who performed CFD-based comparisons of uncomplicated type B dissection with cases featuring subacute or chronic false lumen dilatation. Groups of seven patients each were considered, reconstructing patient-specific geometries from CTA data and computing hemodynamic indicators such as flow rates and TAWSS. They found that patients with rapid false lumen dilatation had growth rates of 5.3±2.7mm/month, while the stable control group had growth rates of 0.2±0.02mm/month. Although neither the initial aortic diameters, false lumen size, nor peak velocities differed between groups, the false lumen flow rate, entry tear size, shear stress, area of high shear stress, and TAWSS (ignoring the dissection flap) were significantly elevated in the group suffering rapid false lumen dilatation. As shown in Fig. 61, patients with aortic dilatation experienced strong recirculation in the intimal tear region and the proximal false lumen. Despite the greatly simplified boundary data, i.e. prototypical inflow conditions from the literature that are equal for each case and zero pressure boundary conditions, they were able to show a remarkably strong link between the hemodynamic quantities and rapid dilatation in type B dissection cases. Because cases may be advanced at the time of data collection, their application for early detection or even prognosis during the acute phase may be limited. Nonetheless, this study also showed the great potential of CFD in personalized medicine, even if only limited data are available.

The case study by Costache et al. [800] reported on the outcomes of a multilayer flow modulator implantation for treating type B dissection, demonstrating its effects on aortic remodeling and associated hemodynamics. Briefly, this device is an aortic stent with multiple layers and varying porosities. Its aim is to reduce volumetric flow rates and promote vessel reconstruction in the false lumen, rather than completely obstructing the false lumen by blocking entry and exit tears along with additional fenestrations. After treatment, the velocity fields showed immediate laminarization of flow in the false lumen, resulting in complete aortic remodeling. In this case, the false lumen gradually disappeared between 12 and 24 months after treatment, and a stable configuration was reached thereafter, as predicted by the CFD analysis. CFD analysis showed decreasing false lumen flow, supporting the argument. The most striking changes were observed in the volumetric flow rate and laminarization in the false lumen. Quantification of these indicators and detailed analysis of hemodynamic indicators such as TAWSS or true-to-false lumen pressure difference would have further supported their conclusions. However, this work is an excellent example of how computational models can be used in medical device design, demonstrating their effectiveness or optimizing their function. In the following years, this work was further extended in subsequent studies aimed at understanding aortic remodeling and hemodynamics both before and after deployment of such multilayer stents [801,802].

The longitudinal case study by Costache et al. [802] investigates the effectiveness of a multilayer flow modulator by virtually analyzing pre-operative, immediate post-operative, and annual follow-up data from 23 patients over a 36-month period. The treated cases were complicated acute, subacute, and chronic type B dissection, and CFD analysis demonstrated effective relaminarization of aortic flow following a previously defined protocol [800]. The presented study, supported by the CFD results, showed that the implanted devices modulate the flow field, leading to laminar flow and a reduction in peak WSSs. This might promote positive aortic remodeling and false lumen thrombosis, while over-stented vessels remain patent due to locally reduced stent porosity. Additionally, no device migration, kinking, or fracture was observed, making this device a particularly viable option. Regarding the contribution of patient-specific CFD modeling, the numerical results were used to support the quantitative measurements through visualization.

In the study of Pirola et al. [803], the focus is on combining patient-specific geometries with a tuning algorithm to obtain patient-specific boundary conditions that are compatible with pre-operative 4D-flow MRI data and pre- and post-operative Doppler-wire pressure measurements acquired from a type B dissection patient undergoing TEVAR. The medical data were then used further for validation, comparing the predicted flow patterns in a stationary lumen, showing good qualitative agreement of the instantaneous velocity streamlines. In addition, the predicted fluid pressure deviated from the measured data by a maximum of 11mmHg (9.7%). Furthermore, it was demonstrated that the presented methodology allowed the derivation of post-operative patient-specific boundary conditions for hemodynamics, which further enhanced the predictability of virtual surgery. The drastic impact of patient-specific boundary conditions on overall system behavior – such as flow splits, shear rates and stresses, pressure distributions, regions of recirculatory or stagnant flow, and the related biomarkers derived from them – makes this contribution highly relevant. Adjusting boundary conditions is a costly process, both in terms of time and computational resources. Therefore, tailored algorithms such as the one presented are crucial for real clinical application.

Xu et al. [804] focused on the prognosis of aortic dissection based on CFD simulations and proposed a functional index based on the difference in luminal pressure as an indicator of the hemodynamic status of type B dissection to predict luminal remodeling after surgical intervention. A total of 51 cases of type B dissection were considered, each of which via an initial pre-TEVAR geometry and three follow-ups (at three month, six month, and 12 month). In terms of computational modeling, the blood was considered to be Newtonian and incompressible, imposing flow ratios determined using Doppler ultrasound and pressure waveforms from the literature. Especially for such large study cohorts, collecting more comprehensive data sets for individual patients is challenging, so the limited data per patient is acceptable. Boundary layers were included in each of the models and a temporal and spatial grid-sensitivity analysis for representative cases revealed that the grid and time step sizes employed were appropriate. The main contribution of this work was statistical investigations using a linear mixed-effects model. This analysis showed that the more distal the first balance point, the better the functional status of aortic dissection after TEVAR repair. Patients with the first balance point still present in the abdominal region showed clinically unsatisfactory results. On the other hand, it was shown that cases in which the first balance point was moved from the dissected region after TEVAR showed improved long-term results, meaning reduced false lumen diameter and true lumen expansion. This study thus showed, using a significant number of cases, that (i) the effectiveness of TEVAR could be quantified using CFD by shifting the first balance point distally and (ii) that the same indicator could be used to predict subsequent false lumen dilatation. The lack of a first balance point indicated stable configurations since the true and the false lumen pressures might be instationary, but the sign of the true-to-false lumen difference does not change over time. This did not lead to a change in the hydrodynamic forces on the dissection flaps, which was the crucial aspect in this context.

Armour et al. [805] investigated the influence of the choice of inflow velocity profile and waveform, targeting two cases of type B dissection, the latter including pre- and post-TEVAR configurations. Because patient-specific 4D-flow MRI is not routinely acquired and the need for immediate surgical intervention may make data acquisition impossible, establishing inlet flow profiles based on the limited data available is crucial. This study compares 3D flow profiles reconstructed from 4D-flow MRI with through-plane profiles and flat inlet velocity profiles with non-patient-specific flow rates, the latter mimicking missing data scenarios. The geometries are derived from CTA images and three-element Windkessel models were applied at the outlets. For the first case, the parameters were tuned based on Doppler-wire pressure readings combined with flow splits calculated from the 4D-flow MRI data. For the second case, however, missing patient-specific data had to be artificially supplemented. For the post-TEVAR configuration of the second case, the left subclavian artery occluded by TEVAR was blocked in the CFD model, redirecting flow. Grid convergence studies were performed based on the velocity and TAWSS in selected planes and the computational model was validated with the 4D-flow MRI data, while the changes in the solution and hemodynamic indicators are reported using the 3D flow profile as a reference. Differences in the TAWSS and velocity fields were most distinct in the ascending arch, which was expected but also of great importance for simulations of type A dissection. In type B dissection, the overall flow patterns were relatively independent of the specific inflow profile chosen. However, discrepancies in TAWSS and the true-to-false lumen pressure difference of up to 6% were observed when changing the inlet condition, especially near the entry tears and additional fenestrations. Regions with low WSS were more affected, especially when the entry tear was near the left subclavian artery. Using non-patient specific waveforms and stroke volume, there was a 30% reduction in WSS and maximum velocity, resulting in a 25% lower flow rate compared with 4D-flow MRI measurements. Furthermore, the chosen waveform had a substantial impact on the predicted pressure. They concluded that 3D, through plane, or flat inlet profiles can be used, resulting in similar flow patterns and TAWSS distributions, while the second yields a better approximation of the true-to-false lumen pressure difference than the flat profile. If no patient-specific inflow data can be obtained, the stroke volume and heart rate, i.e. the volumetric flow rate over the cardiac cycle, must be carefully selected. Due to the changes in TAWSS depending on inflow condition, thrombus formation models that include a TAWSS threshold are also severely affected, so incomplete inflow data can lead to increased uncertainties in predicted thrombus growth. Doppler-wire pressure measurements gave a difference between true and false lumina of 2.3mmHg, while the CFD simulations resulted in 4.6mmHg, most likely due to the assumption of a fixed grid and neglected vessel compliance. At the same time, the flat inflow profiles produced errors of up to 6% and the effects of prescribing non-patient-specific flow rates resulted in errors of 25% in terms of this hemodynamic indicator. Therefore, it is likely that both neglect of vessel compliance and the introduction of artificial inflow ratios can lead to drastic errors in the simulation. This effect is expected to be even more relevant when flow in the ascending aorta is of central interest.

The study presented by Zhu et al. [806] focused on a comparison of geometrical and hemodynamic parameters in a total of 17 cases of type A dissection. Using a false luminal growth rate of 2.9mm/year as the threshold, nine cases were categorized as stable, while the remaining eight cases were categorized as unstable. Each patient’s pathology was assessed with CTA imaging and further processed to allow for CFD analysis. Multiple follow-up CTA image sets were obtained for each patient, allowing the identification of potential factors indicative of dilatation risk based on the false luminal growth rate *in vivo*. The entry tear and possible fenestrations were identified along with the radii of the true and false lumina. Doppler ECG was employed to determine the maximum flow velocity, which was combined with patient-specific heart rates and physiological flow transients to prescribe suitable volumetric flow rates at the aortic root. Three-element Windkessel models were optimized assuming that 21% of the volumetric flow exits through the arch vessels and the flow ratios depend linearly on the outlet surface areas. Despite the limited sample size, this study had significant implications due to the following four conclusions. First, the stable and unstable configurations of type A dissection did not significantly affect either the size of the entry tear or the volume ratio between the true and false lumina. They argue that this is due to the complex geometries and false lumen flow rate and pressure, which are not solely dependent on tear size and volume ratio. Second, the stable cases had a larger number of fenestrations. Third, the stable cases showed a more even flow distribution between true and false lumina, while the true lumen velocity was increased in the unstable cases. Therefore, more even TAWSS distributions were seen in the stable cases, whereas the unstable aortic dissections showed increased heterogeneity and maximal WSS. In all cases of stable and unstable aortic dissections, recirculatory and helical flow patterns as well as strong flow separation were observed. Finally, in conjunction with the lower number of fenestrations, the true-to-false lumen pressure difference increased in the unstable cases, reaching 6.7mmHg, while in the stable cases they were 0.9mmHg. Therefore, the geometrical analysis along with hemodynamic investigations following replacement of the ascending aorta seem promising for predicting late false lumen dilatation.

**Fig. 62.**
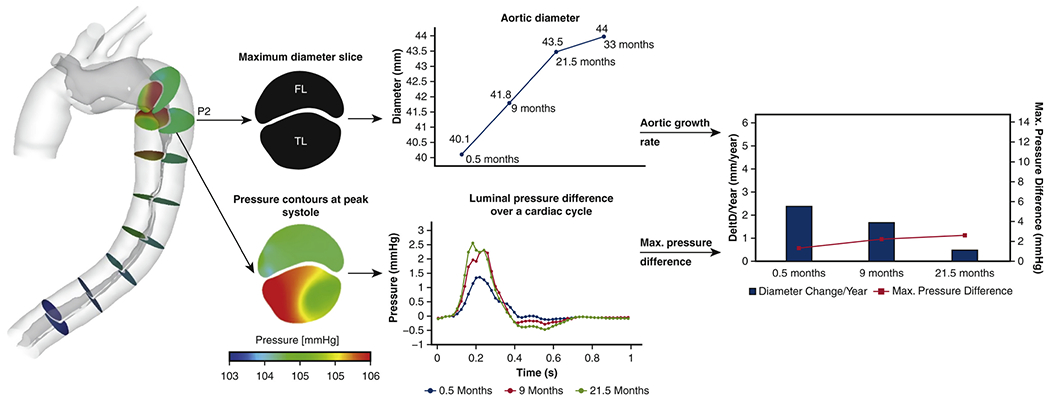
Patient’s aortic diameter changes at the maximum diameter slice, along with true-to-false lumen pressure difference variations during a cardiac cycle. The aortic growth rate (mm/year) is evaluated and compared at each follow-up scan, along with the maximum luminal pressure difference over a cardiac cycle. Both the true and false lumina have outlets where it is difficult to obtain flow data. Imposing boundary conditions inevitably affects the results and causes the pressure difference and flow rates to be influenced by the enforced flow rates or pressures chosen. In clinical practice, such situations are often unavoidable but create a modeling conundrum. The presence of associated true and false lumina outlets or extensive dissection flap fenestration would mitigate this effect. Figure is reprinted from Zhu et al. [807] (licensed under CC-BY 4.0).

In a follow-up work, Zhu et al. [807] then focused on the true-to-false lumen pressure difference as the main hemodynamic indicator for predicting increasing aortic diameters, as shown for a selected patient in Fig. 62. Maintaining the numerical setup unchanged from [806], longitudinal case studies were performed using CFD analysis on four cases after type A dissection repair, tracking the true-to-false lumen pressure difference and maximum aortic diameter in 12 cross-sections along the aorta. The resulting data were then assessed using a linear mixed-effects model to identify the statistical relevance of an increased true-to-false lumen pressure difference. They showed that the true-to-false lumen pressure difference can predict unstable growth (above 2.9mm/year) in the current, relatively small, and heterogeneous patient group. Using a receiver operating characteristic curve, values of >5mmHg true-to-false lumen pressure difference were identified as promoting unstable aortic growth. They noted that the small sample size of only four patients and the highly different medical histories among them – including one patient with mechanical aortic valve replacement, another with Marfan syndrome and hypertension, a third with a bicuspid aortic valve, and a fourth with unstable aortic growth – are limitations of this study. Classifying patients into groups according to their medical history in larger cohorts is required, but the strategy used has high potential for clinical application. As far as computational modeling is concerned, limiting CFD to a fixed grid can have drastic effects on the pressure difference, as a highly mobile dissection flap can significantly reduce the true-to-false lumen pressure difference. In addition, the three-element Windkessel models were not fitted to patient data but were selected based on the approach described in [806]. Specifically, the maximum velocity and heart rate were measured and then combined with an assumed typical flow waveform and a flat inflow profile to obtain a realistic, partially patient-specific inflow condition. This has a direct impact on the absolute pressure values, while the effect on the true-to-false lumen pressure difference may be smaller. The true-to-false lumen pressure difference could actually be influenced by the number and location of the Windkessel outlets relative to the fenestrations and the false lumen geometry. In this present study, the computational domain was shortened so that the false lumen has a Windkessel boundary, as shown in Fig. 62, which leads to a drastic influence of the Windkessel parameters on the false lumen pressure, and hence the true-to-false lumen pressure difference. Consequently, obtaining credible boundary data is particularly difficult. However, such configurations can not be avoided in clinical practice, which motivated their discussion. Despite these limitations, promising results were presented, highlighting that luminal pressure was a particularly relevant hemodynamic indicator that could be used in clinical practice in the future.

Parker et al. [808] investigated partial false lumen thrombosis and quantified the relation between hemodynamic pressure, thrombus morphology, and clinical outcome in type B dissection. CTA data were collected from 69 patients, each correspondingly divided into groups based on thrombus morphology. The true-to-false lumen pressure difference was determined, a survival analysis for adverse aortic events at one year was carried out, and a morphology classification were performed. This study showed that patients with proximal thrombosis had a 10.1mmHg decreased false lumen pressure compared with patients with minimal thrombosis. This reduces the risk of acute complications, resulting in fewer late adverse aortic events within a year. Note again that they observed no differences in WSS, low and oscillatory shear, or OSI between thrombosis groups. They identified proximal false lumen thrombosis at initial presentation as the ‘strongest clinical predictor of both reduced perfusion pressure in the false lumen and favorable short-term and one-year outcomes’. However, it remains unclear whether the proximal thrombosis results from an overall favorable condition, thus being merely a positive side effect, or whether it initiates a cascade of beneficial events.

Armour et al. [809] investigated the role of multiple fenestrations in type B dissection in a longitudinal case study. For this purpose, a controlled swine model was analyzed using CT imaging and 4D-flow MRI as well as computational modeling to assess aortic hemodynamics. In a previous study by Guo et al. [607], as detailed in Section 4.2, a type B dissection swine model with a distal tear 7cm beyond the left subclavian artery was developed. In the current study, a re-intervention was carried out to introduce two additional fenestrations, one of which was nearly occluded. This resulted in three configurations: one with a single natural exit tear, one with a natural exit tear and one additional fenestration, and one with a natural exit tear and two additional fenestrations. Image and flow data were collected before and after the intervention, including multiple follow-ups. 3D inlet profiles were constructed from the 4D-flow MRI data and Windkessel models were fitted to flow rates or Doppler-wire pressure measurements when available. Instationary flow simulations were performed to collect flow fields, reverse flow index, TAWSS, and pressures in the true and false lumina. In the combined study, false luminal growth stabilized with the introduction of additional fenestrations, and false lumen volume decreased by 6% over time. Additional fenestrations altered the flow distribution and increased the flow through the true lumen by 22%. This, of course, altered the observed velocities; TAWSS was reduced at the tears, and the pressure difference between the true and false lumina also decreased. They showed that the introduction of additional fenestrations could be beneficial when the pressure in the false lumen is high and the false lumen was fully perfused. This strategy could be used to stabilize false lumen dilatation, making dissection flap fenestration a key aspect in terms of hemodynamics and control of false lumen dilatation.

Another longitudinal case study using CFD simulation techniques was conducted by Fatma et al. [810]. The hemodynamics of two cases with residual type B dissection following surgical repair of type A dissection were explored. Only one of the two considered patient-specific geometries showed a favorable evolution, potentially allowing the identification of hemodynamic features that lead to further disease progression over time. The aortic wall and dissection flap were assumed to be rigid, blood flow was considered laminar based on the maximum observed Reynolds number and the minimum critical Reynolds number, a generalized Newtonian fluid was considered via the Carreau-Yasuda law, and the Windkessel parameters for regulating flow rates at the outlets were fitted to reproduce the flow rates and pressure as under hypotensive treatment. It should be noted that these parameters were not adjusted to each specific patient’s data. A mesh sensitivity study was also carried out and further assumptions were introduced. First, identical volumetric flow rates from the literature were prescribed for both patients. This is a common obstacle in scenarios where flow data are not available, but can potentially alter the observed hemodynamics, particularly in the cases presented where the radii of the ascending aortas differ by 15%. Second, and more strikingly, the computational domain for the favorable outcome case was shortened by trimming the true and false lumina. If volumetric flow data were available for true and false lumina, one might fit the Windkessel parameters for these outlets as well, but the present study appears to avoid this complication by combining the true and false lumina into a single lumen where relative flow rates and pressure levels are easier to determine. This chosen strategy, which neglected the computational domain at the celiac trunk and combines the true and false lumina, could have a dramatic impact on the results obtained and once again highlights the exceptionally challenging aspects of modeling aortic dissection. Nevertheless, they identified three intimal tears when analyzing the two cases and the respective follow-up examinations. The case with a subsequent unfavorable outcome had larger tear diameters, which increased by 72 and 59%, respectively, during follow-up. The large entry tear was located in the descending aorta. In contrast, the case that resulted in a favorable outcome featured a smaller tear just distal to the implanted Dacron prosthesis that decreased in area by 41% over time. Overall, significant remodeling was observed in both cases, leading to a pronounced volume increase (27%) in the unfavorable case with partial thrombosis, compared with a 19% volume increase in the favorable case. Using CFD, Fatma et al. [810] then identified four possible markers of late complications, namely (i) a sustained high volumetric flow ratio into the patent false lumen above 50%; (ii) a high WSS at the entry tear (>18Pa) and on the false lumen wall (regional >10Pa), caused by the flow jet through the entry tear hitting the false lumen wall and creating vortex structures; (iii) low TAWSS into the distal false lumen (<0.5Pa); and (iv) pronounced helical flow patterns and recirculation. Interestingly, they report that a comparison of the vortex structures in the two cases presented is much more pronounced than the differences in the areas showing combined low TAWSS and high OSI values. This observation motivates further investigation on the vortex structures within the false lumen, as these could also lead to promising hemodynamic markers in aortic dissection.

Osswald et al. [811] investigated the relationships between WSS and the development of new entry tears caused by distal stent-grafts using what is known as the frozen elephant trunk procedure, see Section 2.3.2. It combines replacement of the aortic arch with a prosthesis, while dissection of the descending aorta is treated with a stent-graft. Five patients with diagnosed with dSINE were retrospectively analyzed using CFD both pre- and post-complication. They examined hemodynamic alterations with a focus on WSSs in CTA image-based geometries. To reduce the computational effort, steady-state simulations of the geometries were carried out, assuming a laminar incompressible flow of a Newtonian fluid, a constant inflow taking 0.4m/s as the average flow velocity, and an outlet pressure of zero. This avoids the time-consuming tuning procedures related with Windkessel models and fills in missing data with only a few, but fairly strong assumptions. Despite these assumptions, they were able to detect a significantly increased average WSS within the stent-graft and in its landing zone, while only a small increase was observed distal to the stent-graft. Additionally, the WSS was found to be higher at the convexity of the aorta than at the concavity, which is due to the redirection of flow and the creation of Dean vortices. They concluded that elevated WSSs after stent-graft deployment may contribute to weakening of the aortic wall and potentially trigger aortic rupture. However, enforcing zero pressure outflow conditions could be viewed as an over-simplification since setting stationary outlet pressures (relative to the outlet area or flow splits) is just as simple as assuming zero pressure, resulting in a more realistic flow split but requiring further pre-processing. Nonetheless, this study showed that CFD studies can be employed to identify cases susceptible to developing new entry tears caused by distal stent-graft and that WSSs could be used as an indicator even with these simplifications.

In summary, these findings suggest that CFD simulations of aortic dissection can indeed support clinical decision-making in both cross-sectional and longitudinal case studies as well as in virtual case studies. However, predictions about the stress distribution within the vessel wall and thus the risk of rupture remain unattainable. The flow field can actually be recovered *in silico* and provides important insights into connections between topological features such as location, number, and size of tears, resulting flow split between true and false lumina, pressure gradients between in- and outlets and through the dissection flaps, luminal pressure and false lumen dilatation as well as the effect of flow jets on local pressure. These quantities can further be related to allow virtual comparison of different surgical treatment options. CFD alone can therefore provide valuable insights, but depends heavily on the patient data supplied. Therefore, the clinical application of CFD must combine powerful numerical schemes and parameter fitting methods to recover realistic flow splits between outlets. A second fundamental assumption concerns the elasticity of the tissue. In contrast to the healthy case, where neglecting any deformation in the vessel might be acceptable given the vessel does not experience large motions, in aortic dissection the dissection flap could be generally mobile, at least in the acute and subacute phases. Such effects can only be taken into account by using FSI, which drastically increases the numerical effort. From this perspective, CFD modeling still has its place in aortic dissection due to the reduced number of required parameters, simpler discretization in terms of mesh construction and solver design, and, most importantly, shorter run times, which are crucial in clinical applications. Depending on the specific case, fixed-grid flow simulations can be an adequate tool, while in the case of finite deformations or if the stresses in the tissue are to be examined, the coupling of fluid and structure must be taken into account.

### 6.5. Key findings and publication index

To summarize this section, we present the publication index of hemodynamic *in silico* models in Table 7, with a focus on CFD simulations of aortic dissection using both simplified and patient-specific geometries. Due to the large number of studies available, only a selected range was discussed; however, this table covers an even broader range of studies. Essential information about the modeling approaches and patient cases as well as the objectives of each study is included. Finally, the most important findings are listed conclusively.

- Depending on the pathology in question, modeling via fixed-grid CFD may be possible, while other configurations may require the use of more advanced FSI schemes. For healthy aortas with little radial motion, consideration of the coupled problem may not be essential depending on the aim of the study and available resources.
- Studies have shown a strong correlation between *in silico* models and *in vivo* or *in vitro* data. However, these studies also highlight the sensitivity of the results to the *in silico* model itself. More specifically, CFD simulations require several advanced methods to handle the immense complexity of this application, including: (i) a grid resolving the domain and the solution accurately, (ii) suitable inflow and outflow conditions, and (iii) realistic rheological parameters. Even minor changes in these parameters can lead to significant deviations in the results.
- The characteristic flow jets can cause a local increase in pressure on the outer wall of the false lumen and, depending on the entry tear location, trigger a disturbed, oscillatory flow in the proximal false lumen. The volumetric flow through the false lumen heavily depends on the location and size of the primary entry and exit tears, as well as on additional fenestrations. The same parameters also affect residence times in the false lumen and thus possible false lumen thrombosis. The pressure in the true and the false lumen can be elevated in the arch region and is generally higher than in FSI models when CFD is taken into account, i.e. if the cushioning effect of a deformable vessel and a dissection flap is neglected.
- By virtually adding or occluding fenestrations, the entry tear, or the exit tear, CFD models enable the study of possible implications of stent-grafts or other medical implants for hemodynamics in a patient-specific setting. Specifically, the major influence of tear occlusion on flow in the false lumen was demonstrated and its relation with thrombosis in the false lumen was established.
- WSS concentrations are observed locally in the aortic arch region as the ejected fluid is reoriented and Dean vortices form. The region in which the dissection flap separates from the remaining vessel wall can also be exposed to increased stress, which largely depends on the vicinity to the intimal tear and aortic arch as well as the geometry and location of the intimal tear.
- The true-to-false lumen pressure difference in aortic dissection is highly patient-specific and is influenced by morphological factors such as the number, size, and location of intimal tears, as well as the volume of the false lumen. Typically, the dissection flap maintains a pressure differential of 2 to 3mmHg, but in some cases, it can exceed 5mmHg. This pressure difference can be positive, meaning that the true lumen experiences higher pressure, especially during systole. However, it can also be negative, where the false lumen has higher pressure because it cannot release the buildup pressure as quickly as the true lumen. Moreover, the gradient direction of the pressure difference can also vary along the length of the dissection flap. This dynamic can vary significantly based on the individual’s unique morphology, ultimately influencing the direction of flow within the false lumen and through the respective tears.
- Despite the necessary and sometimes major assumptions, the derivation of hemodynamic parameters now finally allows for the prediction of complications where purely geometric indicators might fail. True-to-false lumen flow splits and pressure differences, WSS-based indicators, and localized stress concentrations are promising examples in this regard. However, it is still unclear how reliably CFD models describe the clinical environment in general. The numerous studies presented highlight their potential for clinical application, even if only routinely acquired medical data are available. One of the biggest remaining problems is the complex pre- and post-processing and simulation turnaround times, which represent a major bottleneck for personalized medicine. This last aspect in particular motivates further research and development, not only in the application and combination of numerical methods but also in the development of the underlying numerical methods themselves.

## 7. *In silico* fluid–structure interaction models

The simulation of aortic dissection and all its essential elements is a particularly multifaceted and challenging task in terms of biomechanical modeling and from the perspectives of mathematical solutions and computer implementation. The basic components are largely the same as in hemodynamic simulations of the cardiovascular system, namely the reconstruction of the computational domain, the selection of appropriate inflow and outflow boundary conditions, and the definition of applicable fluid and tissue models. Incorporating the motion of the flap and the aorta as a whole has been shown to significantly alter the computational results and possibly the conclusions drawn [448,868,869]. When performing FSI simulations, additional complexities emerge, especially in the selection and customization of boundary conditions and constitutive models for the vessel wall to address patient-specific characteristics. In this discourse, the emphasis is on contributions and work that specifically target aortic dissection. Other aspects have been extensively covered and discussed in the literature; see [22,24,870,871] and others.

For various reasons, it is extremely difficult to account for vessel wall compliance by solving an FSI problem. From a mathematical perspective, challenges lie in the parameter combinations that occur in hemodynamic applications. As the density ratio of solid to fluid approaches one, high added-mass effects come into play, hindering the convergence of standard partitioned coupling schemes but also impacting monolithic solution approaches. In addition, incompressible fluid and solid phases have to be considered, further limiting the admissible formulations and solvers for each tissue and blood model. The difficulties encountered in CFD modeling of the circulatory system and numerical treatment are transferred to the FSI case because the FSI problem naturally involves a fluid flow problem. However, in addition to the fluid and solid subproblems, interface tracking or interface capturing techniques must be employed to consider the domain and interface motions. The coupled problem typically involves fluid and solid subproblems, mesh deformation, and strategies for decomposition or level-set updates. It must also ensure effective coupling of the involved fields to achieve temporal stability. Specialized modeling approaches for viscoelastic support, pre-stress in the tissue at the time of image acquisition, and the connection of inlets and outlets significantly increase complexity and computational effort for both the fluid and solid phases. However, compared with fixed-grid CFD simulations, it is not clear to what extent or under what circumstances domain motions or interaction of blood and tissue can be neglected. It has to be evaluated on a case-by-case basis if the additional computational effort, time to solution, and the introduction of additional parameters to adapt to clinical data are well invested.

### 7.1. Simplified studies

Similar to CFD approaches, useing idealized geometries in an FSI setting can enhance the basic understanding of the mechanisms underlying aortic dissection. Especially due to the complexity of FSI models, this may be a good first step toward patient-specific applications. In this overview, the available FSI models based on simplified geometries are briefly presented and their most important findings are summarized below.

In this manner, Qiao et al. [872] investigated the effects of bypassing type B dissection using two alternative virtual bypass strategies: (i) bypass between the ascending aorta and abdominal aorta and (ii) bypass between the left subclavian artery and the abdominal aorta. Due to the choice of parameters, the maximum flap displacement at peak systole in a blind (or patent) lumen model before bypassing was approximately 1.3mm. However, even in this scenario, comparing a rigid wall simulation with the full FSI model revealed significant differences in the observed fluid pressure and velocities, highlighting the potential significance of FSI modeling in the context of aortic dissection. Furthermore, they demonstrated *in silico* that the bypass strategies positively influenced the vessel walls by reducing the mean pressure amplitudes, both in the case of a false lumen with an exit tear and in a virtually created blind lumen.

Their study was subsequently followed by numerous others. Chen et al. [873] investigated blood flow and flap wall motion using a simple approach consisting of a tube with an integrated flap. They identified regions of stress concentration at the leading edge of the flap. Jayendiran et al. [874] developed a simplified composite structure composed of a human aortic segment. The segment was modeled with three aortic layers using both isotropic and anisotropic material models in conjunction with a Dacron graft. This setup made it possible to examine the interaction between fluid and structure. In a related study, Ryzhakov et al. [875] used a monolithic FSI approach to simulate fluid flow in a flexible tube with a parallel channel connected via entry and exit tears formed on the basis of the *in vitro* study of Rudenick et al. [618]. Comparisons between rigid and elastic flaps revealed that the elasticity of the silicon flap had minimal impact on fluid flow. Keramati et al. [876] concentrated on studying blood flow characteristics within an idealized aortic dissection geometry that included 5 and 10mm diameter tears. Their findings suggested that flow resistance was lower and less flutter of the dissection flap occurred when both entry and exit tears were 10mm in diameter, resulting in increased stability. They later compared this model with a reduced-order model [877] and, subsequently, analyzed the sensitivity of hemodynamic quantities to the lumped parameters of the 0D, or reduced-order, model using a Monte Carlo simulation [878]. A rudimentary model, which included a healthy aorta and three distinct types of aortic dissection models, was developed by Saveljica and Filipovic [879] to study the interaction between fluid and solid. Building on previous studies [756], see Section 6.1, the findings by Qiao et al. [880] provided valuable insights into understanding the energy loss mechanisms in aortic conditions, with viscous friction in particular contributing the most to energy loss, followed by deformation of the aortic wall. Aghilinejad et al. [881] adopted immersed boundary and lattice–Boltzmann methods (IB-LBM) to explore the relationship between endograft length and hemodynamic variables within the true and false lumina. The findings revealed a nonlinear trend, indicating a progressively increasing reversal of false lumen flow as endograft length increased. Recently, Kim et al. [882] conducted a study of dynamic obstruction of the true lumen in type B dissection, and Lee et al. [883] examined the effect of cannulation on hemodynamics with two simulated methods: (i) axillary cannulation through the branchiocephalic trunk and (ii) combined axillary and femoral cannulation through the branchiocephalic trunk and the right common iliac artery. The first method resulted in a pressure difference between the lumina, causing the true lumen to collapse. In contrast, the second method maintained similar pressures, preventing the collapse of the true lumen. Additionally, they found that the stiffness of the flap influenced the collapse of the true lumen.

Subsequently, two studies by Chong et al. [884,885] established an FSI model that took thrombus formation into account. To date, this is the only *in silico* model on aortic dissection that combines FSI and thrombus formation. First, Chong et al. [884] developed a simplified geometry of a type B dissection, including an entry and exit tear. They investigated flap dynamics and its correlation with WSS, pressure, and wall stresses. This model was subsequently extended to account for the gradual influence of thrombus formation on the flow field, as described in Section 8.

In summary, simplified models have provided important contributions to modeling FSI in aortic dissection. However, due to recent advances in FSI and the availability of patient-specific models, these are becoming increasingly outdated. However, when incorporating thrombus formation modeling into FSI simulations, simplified models remain the only feasible option to date due to the high computational effort required. Nonetheless, the majority of these studies have made valuable contributions by enhancing our understanding of aortic dissection and encouraging the use of FSI modeling through targeted comparisons.

### 7.2. Patient-specific studies

In contrast to the extensive research on CFD simulations, there are only a limited number of patient-specific FSI models of aortic dissection, largely due to the complexity of the challenges involved. Below is a summary of the basic components and the main findings of these published works.

Alimohammadi et al. [868] published one of the first studies that captured the rheological behavior of blood using a non-Newtonian model, considering the aortic tissue as a 3D continuum in the context of aortic dissection. There, a patient-specific lumen geometry was constructed from CT images and expanded by a fixed margin of 2.5mm to obtain a geometric representation of the vessel wall and a flap thickness of 2.45±0.34mm. For the fluid, the Carreau-Yasuda model was considered along with an SST turbulence model. Data on the inlet boundary were obtained from the literature, while Windkessel models were used to match volumetric flow data and pressure of patients at the outlets obtained from invasive pressure measurements. The constitutive law governing the isotropic, incompressible, hyperelastic response of the tissue was a specific form of generalized power law of the neo–Hookean model [886], and the outlets were not fixed but could expand and contract in the radial direction of the vessel. Furthermore, a uniform external pressure of 52.5mmHg was applied, neglecting the elasticity of the surrounding tissue. Due to the specific geometry and tissue parameters considered, small displacements of the vessel wall and interface were reported, resulting in deviations in the cross-sectional areas of the true and false lumina in the range of ±5%. It is all the more remarkable that when comparing fixed-grid CFD and FSI simulations, clear differences in flow distribution and OSI were found even under these conditions. Additionally, it was observed that standalone CFD computations might suffice when the primary focus is on TAWSS. They argue that critical differences and key features may not be accurately captured when wall and flap motion were neglected in the modeling of aortic dissection, otherwise particularly regions of high OSI combined with low TAWSS were not well captured.

In an attempt to include vessel wall motion at a lower cost, Bonfanti et al. [768] introduced what is known as the moving boundary method (MBM). The displacement of the vessel wall was linearly related to the pressure of the fluid, while the dissection flap remained rigid. For the fluid, a Carreau-Yasuda model was considered along with flow profiles and Windkessel models to account for realistic boundary conditions. The approach was tested on a patient-specific case of type B dissection compared with standard fixed-grid CFD, and an improved match with target patient data in terms of flow rates and pressure waveforms was documented. In [769] the method was then further improved and a deformable dissection flap was also introduced. When comparing FSI, fixed-wall CFD, and the newly introduced method, equal differences in TAWSS were observed, while pressure and flow rates of the MBM were closer to FSI than the fixed-grid CFD approach. Further simplifications were subsequently introduced in [770] using lumped parameter models, as discussed in Section 6. In summary, the aim of these studies was to capture the phase-lag of blood flow and pressure waves due to vessel compliance, which cannot be captured using standard fixed-grid CFD methods. In addition, the parameters in the MBM were tuned using an FSI solution. However, in clinical practice, non-invasive measurements could be employed to perform parameter fitting, which is easier due to the limited number of model parameters compared with complex tissue models that consider hyperelastic, fiber-reinforced 3D continua. Therefore, the main advantage of the developed methods is that they significantly reduce the computational costs, while capturing the relevant effects of vessel motion to some extent, making them increasingly attractive for application in clinical scenarios. For a brief breakdown of potential precision medicine pipelines based on these concepts, see [887].

Qiao et al. [869] presented the first study on aortic dissection considering FSI in combination with two-phase non-Newtonian fluid flow, where red blood cells (the main particulate phase) are suspended in plasma (the continuous phase). A patient-specific geometry was created from CT images, which specified a uniform wall thickness of 2mm. For the boundary conditions of the solid subproblem, a fixed external pressure corresponding to the diastolic pressure in the false lumen [868] was applied. Additionally, the center points of the vessel inlets and outlets were fixed, effectively stabilizing the position of the aortic tree. It should be noted that the influence of the surrounding tissue was not considered. Three-element Windkessel models with parameters from the literature were applied at the outlets and a physiological flow rate was enforced at the inlet. An isotropic, linear elastic material behavior was assumed and the tissue was assigned an increased density of 2,000kg/m^3^ and a Young’s modulus of 2.7MPa. The resulting displacement of the dissection flap in the range of 0.68±0.2mm was found to coincide well with the MRI data from [447]. The observed small wall displacements resulted from the aforementioned supra-physiological stiffness and density, which significantly simplified the numerical treatment. Nonetheless, important conclusions can be drawn from this study, as it accounted for the multiphase nature of blood. The main objective of the study was to compare the results obtained from the FSI and rigid wall models, as well as from single- and two-phase non-Newtonian models, by evaluating OSI and TAWSS, in addition to assessing the deformation of the vessel wall during a cardiac cycle. They demonstrated that the relative differences in WSS between single- and two-phase non-Newtonian models could be up to ±50% during the cardiac cycle, suggesting the potential to improve blood flow modeling in cases of aortic dissection. However, it should be noted that the study did not report absolute differences in WSS. Although the relative differences might seem significant, the absolute values typically fall below 1Pa, suggesting that small absolute differences can result in large relative variations. Consequently, comparing relative values may yield misleading interpretations. According to them, accounting for the complex rheology of blood using multi-phase non-Newtonian models involving individual constituents has dramatic effects on hemodynamic parameters. However, these models typically require detailed patient-specific data, which are often not available. Therefore, single-phase generalized Newtonian models that account for shear-thinning and plug flow effects can be considered a worthwhile compromise between predictive power and complexity. Although this study provided initial insights into the significance of non-Newtonian models, the interaction between larger motions of the dissection flap and the resulting significantly altered flow rates in true and false lumina remains unclear. Similar computational tools that attribute the rheological behavior to single-phase non-Newtonian behavior were then applied in the clinical scenario in Qiao et al. [835]. Herein, pre- and post-TEVAR configurations in acute type B dissection, with and without coverage of the left subclavian artery, were compared in terms of hemodynamic indicators based on TAWSS to quantitatively assess the effectiveness of the surgical procedure.

Whether FSI modeling could provide additional insights into the potential evolution of aortic dissection was investigated by Khannous et al. [888] by comparing the flow behavior within a patient-specific model, both with and without considering FSI. For the analysis, a residual type B dissection was reconstructed from the patient’s CT scans. Blood was modeled as an incompressible fluid with shear-thinning behavior using the Carreau–Yasuda model. Flow rate and unsteady velocity profiles were set at the inlet, and a three-element Windkessel model was used for the outlet. The thickness of the aortic wall was assumed to be constant at 2.5mm, while that of the flap varied around 1.85mm. A linear elastic material model was applied for the aortic wall. The results of the study indicated that the rigid model tends to overestimate the velocity across the domain on average. However, it was observed that the velocities at the entry tear were underestimated. Moreover, a flow jet was detected in the FSI case, which did not appear in the rigid model analysis. This underlines that FSI may provide more comprehensive insights into aortic dissection behavior.

**Fig. 63.**
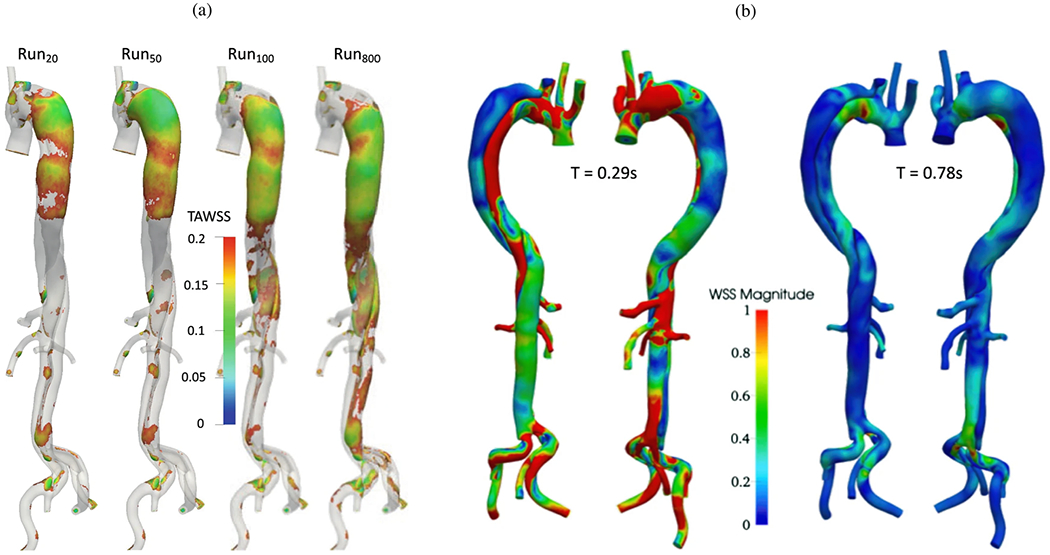
(a) Simulation results show TAWSS distributions obtained with different elastic moduli in the dissection flap. Areas with TAWSS < 0.2Pa are opaque, while the rest are transparent. The region with TAWSS < 0.2Pa expands as the dissection flaps become stiffer. (b) WSS snapshot with an elastic modulus of 20kPa. The left image represents peak systole, the right image depicts end diastole. The WSS magnitude scale is limited to 1Pa (10dyne/cm^2^). Maximum values in the entire domain do not exceed 158dyne/cm^2^. Figure is adapted from Bäumler et al. [448] with permission from Springer Nature.

One of the most notable FSI studies, published by Bäumler et al. [448], involved patient-specific FSI simulations of type B dissection and was acclaimed for its highly realistic approach at the time. Blood was considered as an incompressible Newtonian fluid and the aortic tissue was modeled as an isotropic, hyperelastic and nearly incompressible continuum. Patient-specific 4D-flow MRI data were utilized to prescribe an inflow profile matching the measured volumetric flow rate, and three-element Windkessel models were tuned to account for the downstream vasculature. Furthermore, Robin boundary conditions were considered, see, e.g., [889], to account for external tissue support, with values adopted from the literature to account for elastic support of the aorta. A pre-stressing algorithm [890] was applied to compute the stress present at the time of image acquisition in the solid domain with the exception of the dissection flap, which was assumed to have no stress in the diastolic phase. The anatomical model was developed from high-resolution CTA data, the entry and exit tears as well as multiple fenestrations. The model was further processed manually to achieve a consistent wall and flap thickness of 2mm. This setup enabled investigations of flow rates, true-to-false lumen pressure difference, TAWSS, and geometric quantities such as cross-sectional area or diameter variations. For example, Fig. 63(a) shows the TAWSS, while Fig. 63(b) displays two snapshots of the WSS at peak systole and end-diastole. This contribution highlighted the importance of flap stiffness by varying the elastic modulus of the dissection flap in and around the physiological range in a validated computational environment. Good agreement was found, including remarkably large flap motion within the realistic range of 1.4 to 14.3mm depending on the stiffness of the dissection flap. The combination of an adequate geometric approximation, a monolithic FSI solver including realistic boundary conditions for both fluid and solid sub-problems with patient-specific data leads to one of the most holistic approaches presented in the literature. On the other hand, it remains unclear to what extent the assumption of an initially stress-free dissection flap influences the results achieved or how the inclusion of pre-stress in the dissection flap affects the convergence behavior of the pre-stressing algorithm.

**Fig. 64.**
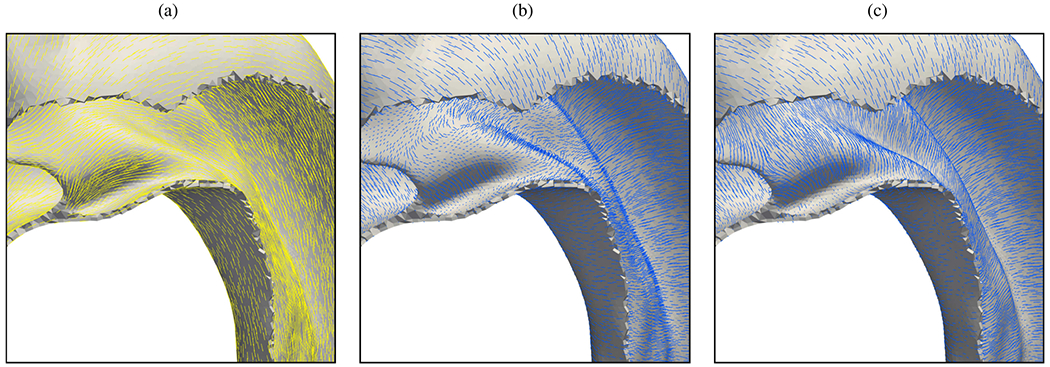
Construction of material orientation basis vectors in a patient-specific aortic dissection model (only aortic arch region shown): (a) axial direction, (b) circumferential direction based on two subsequent Laplace solutions, and (c) circumferential direction as obtained via the approach by Schussnig et al. [892], which uses an extrapolated interface normal. The latter approach delivers better results, especially in the flap region of the aortic dissection. Figure is adapted from Schussnig et al. [892] (licensed under CC-BY 4.0).

The simultaneous analysis of blood flow and tissue within the framework of FSI models is a challenging task not only due to the complexity of the individual fluid and structural models, but above all due to the coupling of these fields. Coupled problems in continuum mechanics occur in various contexts and have their own methods of analysis, mainly distinguishing between monolithic and partitioned approaches. In the vascular context, including aortic dissection, the works by Schussnig et al. [891,892] can be considered key technologies for efficient and stable analysis using partitioned algorithms. They combined (i) Robin transmission conditions, a mixed-type interface condition that simultaneously enforces continuity of velocities and tractions, (ii) semi-implicit FSI schemes that merely iterate the structural displacements and fluid pressure resulting from a consistent split-step fluid solver [893], and (iii) quasi-Newton methods to accelerate the FSI coupling algorithm. This approach proved to be robust and efficient in practical application to a patient-specific case of aortic dissection in [894], taking into account Windkessel models, exterior tissue support, and pre-stress. In terms of constitutive modeling, the shear-thinning behavior of blood was captured via generalized Newtonian models, while the non-symmetric distribution of collagen fibers was captured by the model proposed by Holzapfel et al. [895] with realistic fiber orientations generated by a custom algorithm [896,897], and different tissue parameters in the dissection flap as well as in the medial and adventitial layers of the aorta were taken into account. The results of the algorithm proposed to construct material orientation in aortic dissection geometries are shown in Fig. 64. These works show that partitioned schemes can indeed be applied in a clinical scenario. This is of particular relevance because monolithic coupling was long considered the only reliable FSI coupling strategy in hemodynamics, a viewpoint that is no longer valid today. As [898] shows, partitioned FSI has the potential to outperform monolithic FSI in terms of throughput by several orders of magnitude (factor 100 with parameters in the vascular regime), thereby significantly reducing simulation turnaround times. The adaptation of modern high-performance software for use in a medical context is indeed a promising topic, as it bears great potential to significantly enhance the clinical relevance of both CFD and FSI methods.

**Fig. 65.**
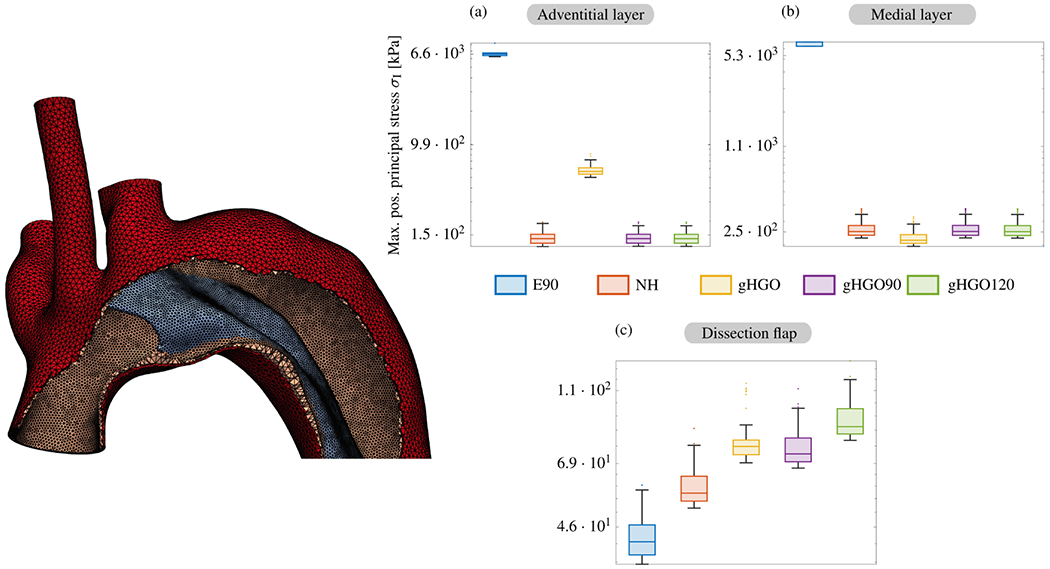
Close-up of the layer-specific finite element mesh in the aortic arch region, showing the cut adventitia (dark red) and media (peach) layers and exposing the dissection flap (light blue) originating at the left subclavian artery: (a) to (c) box plots display the maximum positive principal stress at the time step with the highest value, considering only the top 40% of values and removing the top 1% to exclude outliers. The load distribution between the tissue layers (a) to (c) differs significantly when comparing the E90 with hyperelastic models. The gHGO model with base parameters also differs from the NH, gHGO90, and gHGO120 models in the adventitial layer, indicating different load-bearing behavior. Large differences can be observed in the dissection flap. Figure is adapted from Schussnig et al. [899] (licensed under CC-BY 4.0).

Schussnig et al. [899] then extended the framework previously established by Bäumler et al. [448] while maintaining the original geometry and flow boundary conditions. In this study, they introduced a numerical framework that considered, among other things, a combination of: (i) patient-specific geometry with three layers comprising the combined intima and media, the adventitia, and the dissection flap; (ii) layer-specific tissue models such as the anisotropic constitutive model for the aortic wall layers that takes wall microstructure into account; (iii) a non-Newtonian rheological model; (iv) patient-specific boundary conditions derived from 4D-flow MRI; (v) viscoelastic exterior tissue support; (vi) pre-stressed tissue that counteracts diastolic fluid load; and (vii) prescribed aortic root motion at the inlet. This framework also required the introduction of a local coordinate system, as previously developed and shown in Fig. 64. In summary, the study compared hemodynamic indicators and stress measurements in computational models with varying complexity of vessel tissue material models, from rigid walls to anisotropic hyperelastic materials, making the presented framework one of the most comprehensive for aortic dissection studies to date. The Central new aspects rooted in the detailed analysis of tissue stress measures in a layer-specific and direction-dependent manner, utilizing the material coordinate system embedded within tissue layers. They evaluated and compared dissection flap displacements, flow rates in both true and false lumina, true-to-false lumen pressure differences, and shear stress-based indicators such as TAWSS, OSI, HOLMES, and endothelial cell activation potential. The results closely matched those of previous studies [448]. Most results showed significant differences between rigid wall flow simulations and those using linear elastic and hyperelastic models in an FSI approach. However, some hemodynamic indicators exhibited only small variations. A detailed analysis of stress measurements, as exemplified in Fig. 65, revealed that a significant portion of the pressure load is transferred to the viscoelastic support. In particular, the maximum positive principal stress depends on the tissue-to-support stiffness ratio. In fact, all hyperelastic models with the current parameter set produced mostly similar results. This suggests that existing methods that use relatively stiff viscoelastic support may not adequately replicate the load-bearing behavior *in vivo*. If the viscoelastic support *in vivo* is significantly lower than assumed in the literature, the relative importance of ring forces increases and thus the influence of constitutive modeling, anisotropy, and fiber reinforcement. It is noteworthy that this latter conclusion has received little attention in the past.

Zimmermann et al. [612] then adopted the computational framework by Bäumler et al. [448] and qualitatively and quantitatively compared the hemodynamics in a type B dissection between the FSI analysis and a 3D-printed model, as discussed in Section 4.2 (see Figs. 42(a) to (c)). Both are based on CTA data and assume a dissection flap thickness of 2mm. As previously introduced, the experimental setup allowed tuning of inflow and outflow conditions to achieve physiological inflow waveforms and luminal pressure levels. In the *in silico* model, blood was assumed to be Newtonian and an isotropic neo–Hookean model was fitted to the tensile test results of the polymer used for the 3D printed model. The boundary conditions for the computer simulation were then fitted to the measured *in vitro* data, including the inflow and tuning of the Windkessel parameters. This allowed a comparison of flow rates, changes in cross-sectional area and pressure using 4D-flow MRI, cine 2D MRI, 2D PC-MRI, and pressure catheter measurements at selected points. The flow splits of 78/22 *in silico* and 73%/27% *in vitro* agreed well, but despite adjusting velocities and pressures, both were overestimated in the computational model. The mean pressure in the computational model differed by 15.8%. The expansion of the false luminal area was 11% in the FSI analysis, while it was measured at 5% in the cine 2D MRI, which may be caused by the different mean pressure levels and possible differences in the true-to-false lumen pressure. As already discussed in Section 4.3, discrepancies in the results between *in vitro* experiments and *in silico* models could be due to different resolutions between 4D-flow MRI and 2D PC-MRI. Additionally, higher damping in the 3D printed model, possibly due to underestimated resin stiffness, and neglect of the *in vitro* support from the embedding gel in the FSI simuations may also contribute to these discrepancies. This work highlights two aspects of computational methods for clinical support: (i) FSI modeling can actually be used to predict hemodynamics even in complex pathologies such as aortic dissection, but (ii) fitting all model parameters and obtaining all necessary data is a formidable challenge. Nevertheless, the validation study presented shows that computational biomechanics is indeed a promising tool for application in the cardiovascular system and can provide new insights as long as the model parameters are sufficiently calibrated.

**Fig. 66.**
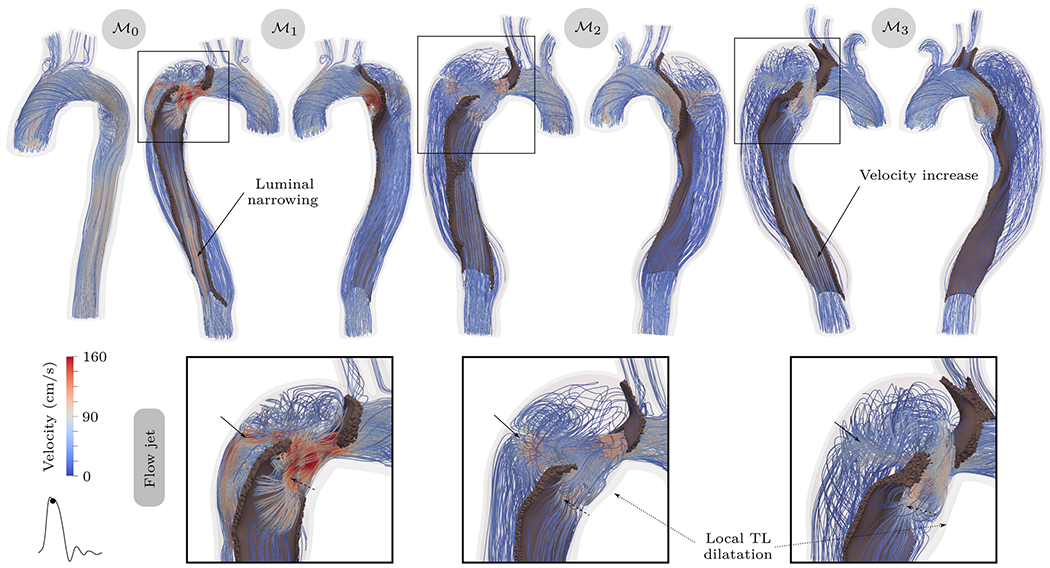
Displayed at peak systole, the flow velocity for the pre-dissection (*ℳ*_0_) and the three post-dissection models *ℳ*_1_ to *ℳ*_3_) highlights the flow jet and the increase in blood velocity in the distal part of the false lumen. Additionally, a close-up view of the region where the flow jet impinges on the outer wall of the false lumen (solid arrow) is provided. This view also emphasizes a localized dilatation in the true lumen (dotted arrow) and the flow separation occurring in the true lumen (dashed arrow). Figure is reprinted from Bäumler et al. [342] with permission from IEEE.

The first longitudinal case study using FSI simulations that captures the evolution of a patient’s aortic dissection, from pre-dissection through the subacute phase to five years after disease onset was recently presented by Bäumler et al. [342]. For this purpose, they applied monolithically coupled FSI models with tissue pre-stress, external tissue support, and an anisotropic tissue model to study potential growth-related hemodynamic markers under realistic conditions. Moreover, the extracted wall geometries differentiate between the tissue layers to allow for layer-specific material properties. Aortic dissection geometries, *in vitro* 4D-flow MRI [612], and the patient’s blood pressure were used to apply *in vivo* boundary conditions at each follow-up. This allowed to study FSI simulation results and anatomical changes, at each available follow-up, which provided the unique opportunity to qualitatively correlate growth-related hemodynamic markers and aortic growth over time to advance our understanding of the evolution and interplay of hemodynamics, biomechanics, and aortic remodeling that contributes to the most common late adverse event in aortic dissections, namely aortic dilatation. Computational results and aortic growth over time were analyzed, revealing a decrease in flow jet velocity at the entry tear as the disease progressed (Fig. 66). This flow jet caused locally increased WSS and blood pressure in the impingement zone on the outer wall of the false lumen, correlating with significant aortic dilatation in these areas. Similar effects were noted in the true lumen distal to the entry tear. Furthermore, the comparable pressure levels in the true and false lumina indicate a hypertensive-like state in the false lumen, particularly during acute phases when the outer wall is abnormally thin. This disruption of homeostasis – characterized by altered WSS, increased pressure, and hypertension – has been associated with changes in mechanosensing and growth and remodeling mechanisms in the aortic wall, highlighting the need for future research in this area.

Expanding on their previous work that neglected vessel wall compliance [806,807], Zhu et al. [900] investigated repaired type A dissection adopting an FSI algorithm and compared the results with pure flow simulations. Two patient-specific models were derived from CTA data, in which the structural domain was generated by uniformly expanding the luminal surface by 1.4mm in the normal direction. The boundary conditions for the fluid flow problem were adopted from their previous study [806] and enforce a flat inflow profile in combination with prototypical flow waveforms fitted to patient data that took into account patient-specific heart rates and maximum velocities. Windkessel models were fitted assuming flow splits at the outlets, with the computational model shortened depending on the case and available imaging data. Therefore, the outflow conditions had to be tuned to achieve the desired volumetric flow rates when exiting the true and false lumina. Although not available, these conditions strongly influence the flow split between the true and false lumina and thus the true-to-false lumen pressure difference. An incompressible Newtonian fluid was considered together with the transitional SST turbulence model. Both the stent and the aortic tissue were considered linear elastic, with Young’s moduli of 7.8 and 1.3MPa, respectively. External tissue support was represented by Rayleigh damping, see, e.g., [901], where volumetric strong damping was used, which should not be confused with conventional spring and dashpot supports as adopted by, e.g., [448,894]. The inclusion of pre-stress was based on the stress state resulting from the diastolic pressure load, which is a reasonable approach.

They reported vessel displacements of about 1mm, which naturally depended on the chosen tissue stiffness. A change in stiffness also led to changes in volumetric flow rates and velocities, as higher true lumen pressure caused displacements of the dissection flaps and expanded the true lumen cross-section. Depending on the specific case and whether the dissection flap has already remodeled or not, these results may be in good agreement with *in vivo* observations. In this study, regions with higher TAWSS are significantly increased when vessel compliance is taken into account, as the relative motion of fluid and solid can potentially contribute to shear in the boundary layers. Furthermore, the turbulence intensity was increased in the presented configurations, but the true-to-false lumen pressure differences were only slightly affected.

**Fig. 67.**
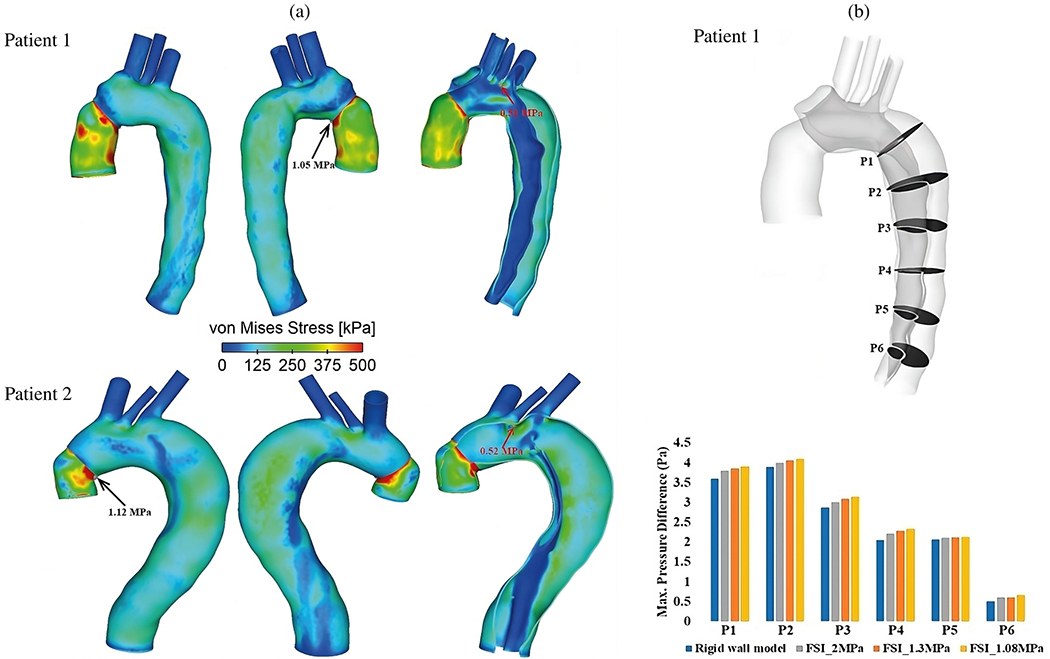
(a) Spatial distribution of von Mises stress for patient 1 and patient 2 in a study by Zhu et al. [900]. Black arrows indicate the highest von Mises stress values at the wall-graft interface, while red arrows highlight isolated areas of elevated von Mises stress in regions not in direct contact with the graft. (b) Quantitative assessment of the pressure differences between the true and false lumina across selected cross-sectional planes of the aorta of patient 1. Pressure differences were analyzed across FSI models that differed in Young’s modulus values. Figure is adapted from Zhu et al. [900] (licensed under CC-BY 4.0).

Depending on the subject of investigation, they also argue that considering FSI in the context of surgically repaired type A dissection does not result in more insights in the clinical setting. In fact, the high costs and the additional model assumptions required do not allow any further conclusions to be drawn than can be obtained from (much faster) CFD computations alone. We would like to emphasize that the issue of data availability is crucial, especially in the patient-specific context. However, as data sets become more available and parameter estimation methods become more popular in biomechanics and medicine, the increased computational and modeling effort is actually justified if simulation turnaround times can be kept reasonable. Tissue properties can then be selected to represent the actual anisotropic behavior, although in this study they had a relatively small impact, apart from changes in TAWSS. However, as shown in Fig. 67(b), wall stiffness also affects the pressure difference between true and false lumina. Even though pressure differences on this scale, i.e. between 0 and 4Pa, are not clinically measurable, they reveal the impact of tissue models and parameters on the results. Such differences can also lead to flap movement and associated risks, as discussed in Section 3.1.

The high stiffness considered corresponds to a remodeled dissection flap, but the external tissue support was not considered via springs and dashpots, but via Rayleigh damping, which can drastically alter overall system behavior, see, e.g., [894,901]. As a result, dissection flap displacements were hindered in multiple ways, so that a vast change in hemodynamics was not to be expected. However, Fig. 67(a) demonstrates tissue stresses in the region of the aortic arch and highlights the typical locations for stress concentrations by plotting the von Mises stress resulting from the deployment of a stent-graft. This highlighted that stent placement induced local stress peaks and thus potential future dissection sites. Since this region was also strongly influenced by inflow and aortic root motion, future studies investigating such scenarios might incorporate the mentioned aspects and anisotropic tissue models to investigate the stress and strain concentrations in more detail.

Wang et al. [902] explored the ability of a biomechanical model to predict risks associated with a non-dilated thoracic aorta in type A dissection. They focused on the correlation between von Mises stress, WSS, and blood flow in a patient with type A dissection. The implemented model included a patient-specific 3D aortic dissection geometry and two aortic layers with constant thickness: intima and adventitia. The anisotropic properties of the aortic wall were modeled using the HGO model. Furthermore, *in vivo*-based physiological time-varying blood velocity profiles from the literature, non-Newtonian rheology (Quemada model), and a turbulence model were adopted. The sizes of the entry and exit tears were derived from the existing literature data. Aortic stress values have been found to be primarily determined by a mixture of blood flow dynamics, wall properties, and arterial tortuosity. The maximum von Mises stress was identified in the region of the aortic arch adjacent to the brachiocephalic artery. Notably, the intimal tear was subjected to less stress compared with the true lumen wall. Moreover, the von Mises stress in the intimal layer exceeded that in the adventitia. However, the study had certain limitations. The lack of a pre-stressing algorithm reduces the significance of the obtained wall stresses. Although the study used patient-specific geometry and *in vivo* boundary conditions – many of which were obtained from the literature from different patients with varying geometries – this may have limited the validity of the results. However, despite these limitations, the use of an anisotropic material model, a non-Newtonian model to describe blood flow, and a turbulence model in an FSI framework represents significant improvements compared with previous works.

In summary, considering the coupling of solid and fluid phases when modeling aortic dissection using FSI can have a tremendous effect on the results obtained in terms of true and false lumina flow rates and true-to-false lumen pressure differences, and derived quantities and clinical markers such as OSI or TAWSS associated with initialization and onset of dissection. Taking vessel compliance into account, these quantities may vary significantly depending on the specific case. By increasing aortic wall stiffness, WSS, and related quantities such as RRT, OSI, and HOLMES undergo potentially large changes. Modeling the tissue allows for studying stresses and strains, which are particularly important in aortic dissection. The dissection flap is dynamically loaded by blood flow, making it subject to significant strains during the cardiac cycle. This dynamic loading may be a key factor in delamination. FSI modeling allows investigations on stress concentrations in the vessel wall due to stent-graft placement or intimal tears of different sizes, locations, and numbers with resulting dissection flap configurations. Likewise, vessel joints or bifurcations, in which there is a relative motion of individual segments, as well as stent-graft ends, where unavoidable misfits lead to a contact pressure being exerted on the vessel from the interior, can lead to localized strains. In all of these cases, the dynamic loading and coupling of solid and fluid can have drastic effects on the stress and strain distribution in the tissue.

Due to the increased complexity and the high computational cost, the numerical treatment and the clinical application derived from it remain a challenge. The need for medical data is even greater compared with fixed-grid CFD approaches, as not only flow rates, pressure measurements, and rheological parameters require parameter tuning. At this stage, critical questions remain about parameter selection for arterial tissue and other relevant models, such as viscoelastic support. Additionally, the extent to which individual constitutive models impact overall system behavior and the best methods for modeling different disease stages are still uncertain. In this regard, uncertainty quantification and sensitivity analysis tools need to be considered to address remaining doubts about the importance of FSI modeling given the high costs involved. In FSI, the relative importance of the subproblems involved and specific modeling techniques as well as the related parameters remain unclear, especially when targeting patient-specific simulations. Therefore, many open questions remain, motivating further research toward modeling aortic dissection using FSI.

### 7.3. Key findings and publication index

To summarize this section, we present the publication index of *in silico* models on FSI in Table 8, with a focus on FSI simulations of aortic dissection using both simplified and patient-specific geometries. Due to the large number of studies available, only a selected range was discussed; however, this table covers an even broader range of studies. Essential information on the modeling approaches, study size, and main objectives of each contribution are summarized. Finally, the most important findings are listed conclusively.

- Overall, there is a favorable correlation between *in vivo* wall deformation and results derived from FSI models; however, the accuracy still depends heavily on boundary conditions and material parameters, the tuning of which requires extensive data sets that are hardly available from today’s clinical practice.
- Aortic dissection configurations with a highly mobile dissection flap require modeling using FSI because the flow field is heavily influenced by tissue deformation. Consequently, alterations in the stiffness of the dissection flap affect both the volumetric flow rate and velocity. If the stress distributions within the tissue are to be investigated throughout the entire cardiac cycle, the dynamic coupling of tissue and blood has to be accounted for.
- Under physiological conditions, pre-stress plays an important role and needs to be rigorously considered, primarily capturing the diastolic pressure acting on the tissue at the time of image acquisition. However, the question of whether the dissection flap is pre-stressed and the magnitude of this effect remains uncertain.
- In addition to the requirements of CFD simulations, namely (i) a grid that resolves the domain and the solution with sufficient accuracy, (ii) suitable inflow and outflow conditions, and (iii) realistic rheological parameters, the computational modeling of aortic dissection using FSI requires (iv) reconstruction of the tissue domain and the pre-stress present in the tissue at time of model construction, (v) appropriate tissue models and related parameters, and (vi) consideration of external (visco-)elastic support.
- In the patient-specific context, it remains unclear whether FSI simulations are required or whether vessel compliance can be neglected. The often tremendously increased runtimes for FSI simulations compared with fixed-grid CFD, the difficult mesh construction and the large number of model parameters continue to make their use in clinical practice difficult. Current literature suggests that the difference in terms of biomarkers may be significant in some cases and negligible in others.
- Without accurately accounting for pre-stress, the anisotropic material behavior of the vessel wall across different tissue layers, and the *in vivo* external tissue support – factors that remain challenging to quantify – the absolute stress magnitudes computed in FSI simulations have only limited value. However, these simulations can still provide useful insights by highlighting regions of stress concentration and indicating the relative stress distribution within the vessel wall.

## 8. *In silico* models on selected topics

In the previous sections, we examined the various *in silico* approaches for aortic dissection, grouped into primary categories such as multiscale tissue modeling, fluid dynamics approaches, and coupled FSI methods. Our objective in this section is to highlight studies with a specific research focus rather than the methodology employed. First, we discuss approaches based on computational solid mechanics (CSM) that investigate wall stress in patient-specific geometries and assess its connection to the tear location. Second, we provide a detailed analysis of stent-graft deployment and migration using an exclusively CSM-based method to study medical implant development and treatment improvement. We then explore thrombus formation models in aortic dissection in both CFD and FSI frameworks, which have the potential to examine, for example, the effects associated with deployed stent-grafts. This section provides a succinct overview of all existing techniques for modeling aortic dissection and highlights the likely impact of these emerging methods on clinical outcomes.

### 8.1. Patient-specific studies on wall mechanics

Several studies specifically targeting aortic dissection are based on CSM, which will be briefly discussed in the following section. In these investigations, patient-specific geometries of individuals affected by aortic dissection were employed to calculate inherent wall stress without incorporating the fluid domain adjacent to the solid domain. Simplified boundary conditions were utilized to account for hemodynamic forces.

In 2004, Beller et al. [910] conducted one of the first *in silico* studies aimed at a better understanding of aortic dissection. They measured the axial displacement of the aortic root in 40 cardiac patients with coronary artery heart diseases (excluding patients with aortic dissection) to investigate whether downward movement of the aortic root during the cardiac cycle could be responsible for the development of the circumferential tear observed in aortic dissection. In addition to the results from imaging, a finite element model of the human aortic root, aortic arch, and supra-aortic vessels was developed, with geometry comparable to a 3D MRI reconstruction. They analyzed the downward movement of the aortic root during the cardiac cycle, along with the resulting torsion and pressure exerted on the aortic wall. In the control model, a luminal pressure of 120mmHg was applied, followed by an additional axial translation of 8.9mm and a twist of 6°. To examine the impact of root motion versus hypertension on aortic wall stress, the analysis was also performed with a luminal pressure of 180mmHg. The results revealed a critical increase in axial stress in the ascending aorta above the sinotubular junction, which may be responsible for the higher occurrence of circumferential intimal tears and aortic dissections at this location. Of note, the right lateral aspect of the ascending aorta, several centimeters above the sinotubular junction, demonstrated increased axial stress with increased axial displacement, pressure, and aortic wall stiffness.

Doyle et al. [911] conducted a study on *in vivo* wall stresses in aortic dissection. Fifty patients and corresponding models were considered. The aim of the study was to estimate the stresses in the tissue more accurately using a patient-specific finite element analysis. To identify significant correlations, geometric parameters were measured and statistical analyses were performed. They revealed that the highest wall stresses were predominantly located in the ascending aorta and the aortic arch. Notably, these regions coincided with areas in which more than 80% of intimal entry tears occur in acute aortic dissection. This research provided valuable insight into the potential factors contributing to the development of aortic dissection and may serve as a basis for future preventive measures. Similar results were reported by Plonek et al. [912]. They studied the aorta both before and after dissection, focusing on pre-dissection wall stress and the location of entry tears. After an initial study with a simplified geometry [913], they summarized two key findings: (i) a strong correlation between high wall stress and the areas where intimal tears occurred, and (ii) the peak wall stress predominantly concentrated in regions of abrupt geometrical changes rather than in the most dilated segments.

Menichini et al. [914] conducted a case study in which they examined two patients. Both patients had undergone TEVAR for type B dissection, with one patient in the chronic phase (patient 1) and the other in the subacute phase (patient 2). Interestingly, a dSINE was observed in patient 1, while the dissected aorta in patient 2 remained stable. Computational analysis revealed that the patient who experienced a dSINE had significantly higher stent-graft tortuosity compared with the patient who experienced this complication, and considerably elevated wall stress in the area next to the dSINE. This case study suggests that increased stent-graft tortuosity may be associated with higher wall stress, potentially leading to complications such as a dSINE in patients undergoing TEVAR for type B dissection.

In a study by Subramaniam et al. [915], they analyzed the distribution of systolic wall stress in two cases of proximal aortic dissection, employing finite element analysis to determine how the stress distribution was affected by the choice of anisotropic material models and aortic root motion. Specifically, they used the Fung-type and the HGO model. In the case of spiral dissection, they discovered that the peak stress correlated with the origin of the tear at the sinotubular junction. However, in another case involving root dissection, the highest stress was found at the distal end of the tear. Interestingly, they found a 9 to 11% increase in predicted tear pressure for root motions of up to 10mm. Comparing the two material models, they noted that predicted tear pressure determined by the HGO model was 8 to 15% lower than the estimate of the Fung-type model. This study indicated that the selection of anisotropic material models and root motion could significantly influence both stress distribution and predicted tear pressure in aortic dissections. However, it should be noted that the stress distribution strongly depends on the selected material parameters, which makes further generalizations difficult. Such statements must therefore be based on carefully tuned tissue parameters.

Hu et al. [916] evaluated the wall stress distribution, particularly at the sites of proximal tears, in patient-specific type B dissection. The study utilized 30 patient-specific CTA images to reconstruct 3D models. The elastic behavior of the aortic wall was described by a third-order hyperelastic Ogden model, where the material parameters were obtained with data from [912]. Furthermore, inner blood pressure and aortic morphology were included in the simulations. Anatomical variables such as aortic angulation, aortic diameters, curvatures, and tortuosity were measured for each patient and compared with the computed wall stress. The results indicated that tears were associated with either locally high or low wall stress regions. Interestingly, the study also identified abnormal aortic angulation as a potentially crucial risk factor that could be monitored to predict aortic dissection.

In conclusion, the previously summarized *in silico* studies have demonstrated that local stress concentrations in patients with aortic dissection, located primarily in the ascending aorta and aortic arch, may be associated with the localization of entry tears [911,912]. Furthermore, these studies suggested that changes in anatomy due to the use of stent-graft deployment could lead to additional stress localization [914]. However, the presented studies appear to be limited due to the small number of patients involved, resulting in restricted geometric variations. Moreover, assessing the applicability of these findings becomes challenging when fluid flow is omitted, and instead, simplified boundary conditions are applied to the exterior or fluid loading is substituted without adequately investigating the implications of such simplifications.

### 8.2. Stent-graft deployment and migration

Simulations of stent-graft deployment have recently become significantly more important, particularly in the treatment of aortic dissection. The FEM including contact formulations is commonly used to replicate the biomechanical interactions between the stent-graft and the aorta during TEVAR procedures. Although virtual stent-graft deployment, possibly in combination with FSI, poses computational challenges, it has attracted increasing interest in recent years. The accuracy and potential of virtual stent-graft simulation models as interventional planning tools have already been demonstrated in the non-dissected aorta, see, e.g., [917–919]. Nonetheless, success in this area remains a challenging endeavor due to the complex anatomical structure of the dissected aorta and the nonlinear contact relationship between the stent-graft and the dissected aorta.

The first known study to assess aortic injuries caused by stent-grafts in aortic dissection was conducted by Ma et al. [920]. They examined the interaction between the aorta and a stent-graft, and identified risk factors for injury using CSM simulations. The study involved a patient with type B dissection who underwent TEVAR treatment with two different types of stent-grafts. The stent wire was modeled as an isotropic superelastic Nitinol, the graft was made from polyethylene terephthalate with a Young’s modulus of 1840MPa, and the aorta was modeled as an isotropic hyperelastic neo–Hookean material with a thickness of 1mm and a Young’s modulus of 5MPa. The TEVAR simulations included compression, bending, and release of the stent-graft. The simulations were performed with or without coverage of the left subclavian artery, and each stent-graft deployment was performed seven times within the landing zone area for statistical analysis. The study found that in all TEVAR models, the maximum aortic stress was exerted on the proximal bare stent caused by the angled contact between the proximal bare stent and the stent-graft.

Stent-graft deployment in a tortuous aorta, such as in aortic zones one to three, which refers to the arch and proximal descending aorta, resulted in significantly increased aortic stress compared with a straight artery. Kinking of the arch and proximal descending aorta resulted in incomplete stent-graft apposition at the concavity, resulting in a so-called bird-beak configuration [917]. The proximal bare stent in the stent-grafts generated higher stress toward the aortic wall than other parts of the stent-graft, and inadequate apposition between the stent-graft and aorta led to a characteristic maximum aortic stress distribution at the convexity. Higher oversizing ratios and the presence of a connecting bar also significantly increased the stress on the aortic wall after stent-graft implantation.

In a follow-up study, Meng et al. [921] then utilized the same patient-specific geometry to examine the biomechanical mechanisms behind new lesions induced by stent-graft implantation. They analyzed the relationship between the radial force and the spring-back force of the stent-graft, especially during virtual implantation with various oversizing ratios between 0 and 15%. The peak wall stress of the aorta was identified at the point where the proximal bare stent interacted with the aortic wall, with its value notably increasing by 62.2%. The simplified computational solid mechanics approach used in these studies did not account for variable blood pressure or viscous stresses, anisotropic material behavior of the aortic wall, variations in wall thicknesses, or outer wall boundary conditions, which might result in misleading and incorrect absolute stress values. However, given the complexity, the chosen approach still seems reasonable and the comparison of the relative values between different stent-grafts provided valuable insights.

**Fig. 68.**
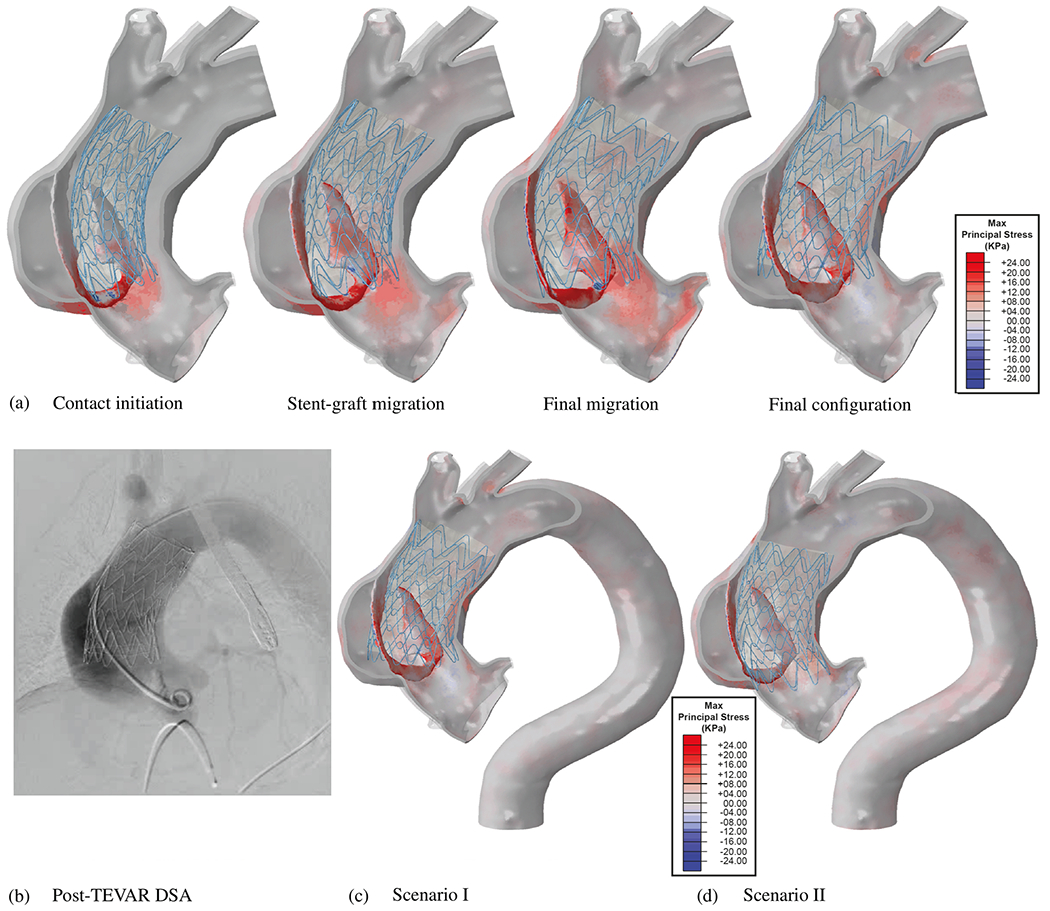
(a) Different stages of virtual stent-graft deployment from left to right: (b) post-interventional digital subtraction angiogram showing stent-graft migration; (c) *in silico* stent-graft model of the actual outcome (scenario I); and (d) landing position following placement of the stent-graft 10mm proximal (scenario II). The final configurations of the stent-grafts in both scenarios are projected onto the post-interventional digital subtraction angiogram. Figure is reprinted form Yuan et al. [922] with permission from Elsevier.

Simultaneously, Yuan et al. [922] developed a framework for a patient-specific virtual stent-graft deployment model using FEM to analyze TEVAR-induced changes under physiological conditions. This framework was later extended by several follow-up studies. Pre-interventional CTA images were acquired from an 80-year-old patient. The model incorporated a uniform wall thickness of 1.5mm and a non-uniform thickness of the dissection flap as extracted from ECG-gated CTA. A second-order hyperelastic Ogden model fitted to circumferential tensile tests of aortic tissue [457] was used to model the aortic wall, while a linear elastic material model was used for the dissection flap. For the simulation, both the proximal and distal ends of the aorta were fixed and constant pressure was applied. The deployed TEVAR device consisted of two components: a metal stent and a fabric. For the quasi-static simulations, a general penalty contact approach was chosen and two different landing positions were simulated. Scenario I corresponded to the position adopted in the actual procedure. To understand the influence of the proximal landing position of the stent-graft on its biomechanical performance, scenario II was simulated in which the landing position was shifted 10mm toward the aortic root. The virtual stent-graft was crimped from its stress-free state onto the diameter of the delivery catheter. Both the proximal landing position and the assumed curvature of the virtual stent-graft were simulated before removing the virtual catheter. The simulation results were validated by comparing the simulated with the actual stent-graft position. Simulation of the real procedure showed that the proximal bare metal stent pushed the flap into the false lumen, leading to further stent-graft migration during the insertion phase. As shown in Fig. 68, an alternative landing position reduced local deformation of the dissection flap and prevented migration of the stent-graft. When deployed in the actual position, a higher maximum principal stress was found on the flap, while the alternative strategy reduced the stress. Note, however, they this study did not take into account the zero-stress configuration of the aortic tissue, the pre-stress of the aorta, pulsatile pressure, or viscous stresses, all of which could influence the final landing position and stent-graft migration. Nevertheless, the applicability in clinical support could be demonstrated using a comparatively inexpensive computational approach.

**Fig. 69.**
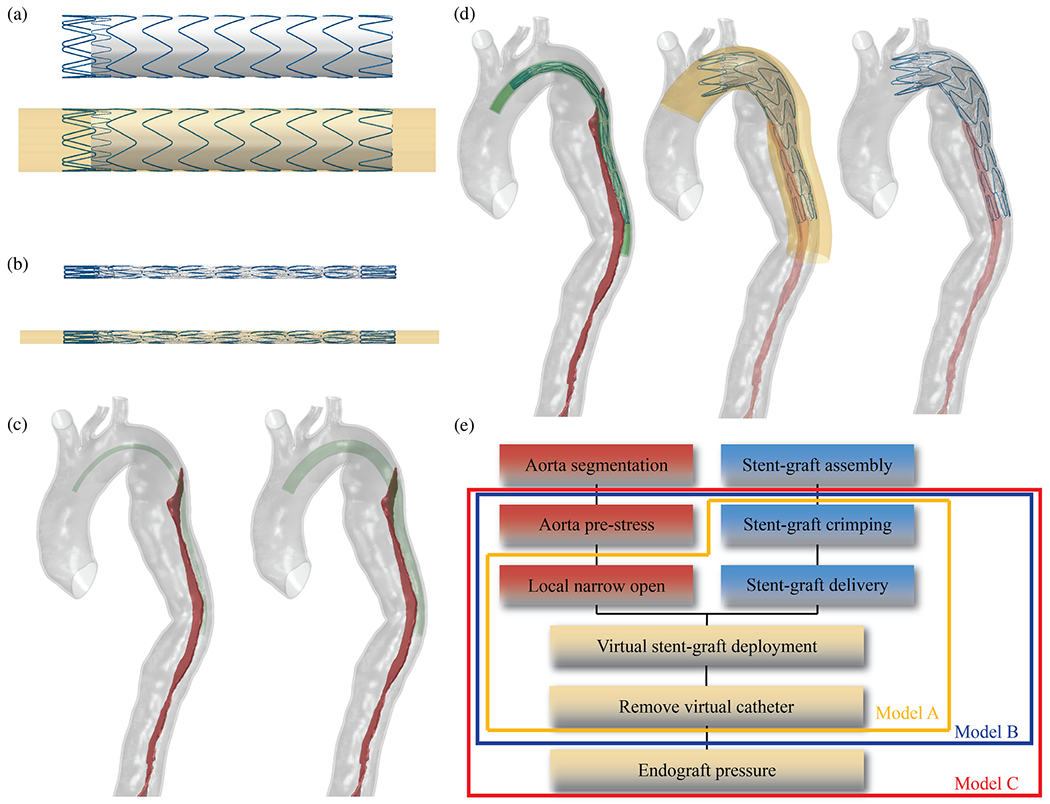
Virtual stent-graft deployment: (a) valiant (Medtronic) stent-graft for TEVAR covered by a virtual sheath; (b) compressing the stent-graft into its crimped state by the virtual sheath; (c) curved tube employed to open the local narrowing in the compressed true lumen; (d) virtual delivery and deployment of the stent-graft at the intended location; and (e) overview of the deployment of stent-grafts in *in silico* with model variants highlighted. Figure is reprinted from Kan et al. [458] (licensed under CC-BY 4.0).

Building on previous work, Kan et al. [458] refined the model by incorporating a pre-stressing algorithm for the aortic wall, as described in [923]. CTA scans were obtained from a 44-year-old patient with type B dissection before TEVAR and three months after the procedure. Similar to the previous study, a uniform thickness of 1.5mm and a dissection flap thickness between 0.6 and 1.6mm were chosen. To determine the material parameters of the dissected aorta, 12 tissue samples were collected from five patients with type B dissection, as briefly summarized in Section 3.2.2. The experimental results were used to calibrate the material parameters of a Yeoh material model. A stent-graft was implanted with a proximal bare metal stent consisting of a Nitinol stent scaffold and a PET fabric graft. The mechanical behavior of the Nitinol stent-graft was reproduced using superelastic material properties, while the PET fabric was simplified by considering it as an isotropic elastic material. For illustration, the TEVAR deployment workflow is depicted in Fig. 69. When the stent-graft was deployed into the true lumen, it successfully sealed the proximal intimal tear. Coverage of the luminal surface by the stent-graft resulted in a change in loading conditions, with blood pressure at the aortic internal surface decreasing from 80 to 0mmHg, and graft internal pressure increasing from 0 to 80mmHg. The simulated post-TEVAR configuration was compared with follow-up CT scans and revealed a strong correlation. The mean deviations were 5.8% in the local open area, which was defined as the enclosed area of the unfolded stent-graft, and 4.6mm in the stent-strut position, which referred to the position of the individual struts of the stent. The deployment of the stent-graft increased the maximum principal stress in the narrowed true lumen but reduced the stress in the entry tear region where false lumen dilatation occurred. Comparisons of simulation results with varying model complexities suggested that the pre-stress of the aortic wall and the blood pressure inside the stent-graft should be included for accurate prediction of the deformation of the deployed stent-graft.

In the follow-up study, Kan et al. [924] applied their numerical framework to a patient with type B dissection who underwent TEVAR during which a tapered self-expandable stent-graft was deployed into the true lumen, sealing the entry tear. Follow-up CTA scans were conducted at three months and 12 months post-TEVAR. The wall and dissection flap thicknesses were assumed to be constant at 1.45mm, with model generation based on [448]. The outer wall was modeled as an isotropic hyperelastic material, while the flap was treated as a linear elastic material, similar to previous studies. Three stent-grafts of varying lengths were included in the virtual deployment simulation. The stent-graft consisted of stainless steel stent struts and a PET fabric graft, both modeled as linear elastic materials. Pre-stress was computed before simulations were carried out and contact between the stent-graft and virtual sheath was modeled using a general contact algorithm via a penalty formulation. The stent-graft was delivered to the target landing position determined by the centerline, as seen in the three-month follow-up scan. The model was able to predict the post-TEVAR stent-graft configuration and wall stress. Comparisons of simulation results for different stent-graft lengths revealed that the maximum stress location varied with stent-graft length. With the short stent-graft, which was actually deployed in the patient, the maximum von Mises stress was observed on the dissection flap at the distal landing zone, where a stent-induced new fenestration was identified at the three-month follow-up. Increasing the stent-graft length caused the maximum von Mises stress to shift away from the distal landing zone, reducing stress values by approximately 17% with the medium-length stent-graft and by 60% with the long stent-graft.

In their third consecutive study, Kan et al. [925] applied their developed computational model to investigate the impact of various stent-graft designs and lengths on wall stress and aortic morphology, particularly in relation to the ‘spring-back’ effect and its connection to dSINEs. Spring-back force measures the tendency of a curved stent-graft to return to its straight configuration [926,927]. They noted that specific design features of stent-graft products, such as the connecting bar, may increase bending stiffness and potentially increase the risk of tears at the dSINE. However, quantifying this effect remains a challenge. For their study, a patient-specific model of type B dissection (pre-TEVAR) was developed, which featured a single entry and exit tear with a uniform wall thickness of 1.45mm in the outer wall of the aorta and the dissection flap. The aortic wall was modeled as a hyperelastic material using a second-order polynomial strain-energy function. Moreover, parametric models were developed for three different stent-graft products, each available in two lengths. These models incorporated various stent and graft materials, strut patterns, and assembly methods, each modeled individually. The study revealed that areas of high stress coincided with regions where tears of the dSINE occurred. Additionally, morphological changes in the true lumen before and after virtual deployment as well as the distribution of the maximum principal stress on the dissection flap were analyzed and compared across five zones for short stent-grafts and seven zones for long stent-grafts. Increased tortuosity was observed at the proximal and distal ends, with reduced tortuosity in the intermediate zones. Of particular note was the third stent-graft model, which consisted of a single helically twisted ring and a tubular expanded synthetic graft, especially in its longer variant. This design effectively minimized the spring-back effect, led to a more uniform expansion of the true lumen, and significantly reduced stress in the distal landing zone. This model demonstrated significant improvements: it reduced the spring-back effect by 65%, achieved a more uniform dilatation of the true lumen by 70%, and lowered peak stress in the distal landing zone by 78%. Overall, these results, with less than 10% stent strut diameter accuracy in validation, underscore the potential of using virtual stent-graft deployment models in surgical planning. Theey also underlined that their modeling approach is recognized as a potential new technique for achieving optimal outcomes in endovascular procedures [126]. To show how the virtual stent-graft deployment model can serve as a pre-procedural planning tool, they proposed a timeline (49 hours) for the management of type B dissection with this *in silico* tool. For further information the reader is referred to [928,929].

In conclusion, all studies discussed simulate the effects of stent-graft deployment, virtual TEVAR procedures, and surgical planning on digital twins, which appear to have enormous potential. In addition, a related study by Zhou et al. [930], which was not discussed in depth but is worth mentioning, focused exclusively on the development of a stent-graft model for deployment in aortic dissection patients. Rapid technological advances combined with increasingly shorter product cycles lead to a substantial demand for reliable *in silico* validation methods that can be executed repeatedly and efficiently. Although the presented approach can still be substantially enhanced, particularly with regard to the refinement of boundary conditions, its integration with technological advances and clinical applications indicates a promising future.

### 8.3. Thrombus formation

In Section 3.4, we discussed the role of thrombus formation in aortic dissection and the relationship between false lumen status and patient mortality, dilatation, and hemodynamics. In brief, thrombus formation is a complex multistage biological process involving many species and biochemical reactions and encompasses the recruitment, activation, and adhesion of platelets to form a blood clot. Therefore, modeling such a process presents significant challenges. Various studies, mainly kinetic-based, have addressed specific stages with a range of assumptions, see [931] for a brief overview. While these studies provide valuable insight into thrombus formation, their focus on the micro or cellular level makes it difficult to apply these methods to an anatomically realistic model in which macro-scale hemodynamics strongly influence the coagulation process. To realistically simulate thrombus formation in combination with the flow field, most aortic dissection models to date are based on hemodynamic approaches, which simplify the complex process and make it applicable to patient-specific models based on the original work of Menichini and Xu [932].

The groundbreaking work of Menichini and Xu [932] introduces a hemodynamics-driven model that predicts thrombus formation in aortic dissection for the first time. This model was originally applied to a simplified aortic dissection geometry and uses shear rates, fluid residence time, and platelet distribution to assess the likelihood for thrombosis and its effect on blood flow. The primary features of the model and the key species transported have been established in a number of previous studies. A computational model for blood flow and thrombosis in patient-specific aortic dissection was first presented in [488] and then validated in [933]. Under physiological flow conditions, they demonstrated how the model utilizes shear stress, fluid residence time, and platelet distribution to predict thrombus formation in a non-moving lumen. Three type B dissection patients were considered in this study, in which predicted thrombus growth was compared with follow-up CT scans. Good agreement was found between the *in vivo* and *in silico* results, with the maximum difference between the predicted and measured false lumen reduction being less that 8%. To reduce the computational cost, accelerated kinetics are introduced into the model, which consequently led to a loss of connection between physical and computational time. Therefore, the need for correlation in time is required to fully bridge the time scales of thrombus formation and fluid flow. Nevertheless, the results were consistent with clinical studies indicating that exit tears and branching vessels are risk factors for incomplete thrombosis. Moreover, the distance between tears was found to be an important factor in determining the likelihood of false lumen thrombosis, which is of high clinical relevance.

**Fig. 70.**
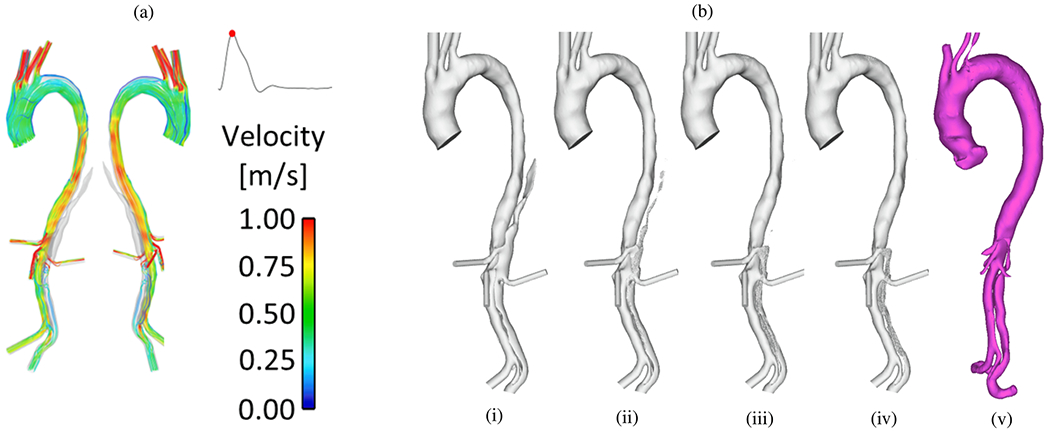
Patient-specific (a) hemodynamics in aortic dissection post-TEVAR with streamlines at peak systole and (b) lumen boundary evolution during modeling of thrombus growth: (i) initial reconstructed geometry, (ii) to (iv) computational approximations, and (v) one-year follow-up showing good agreement between predicted and actual false lumen thrombosis. Figure is reprinted from Armour et al. [934] (licensed under CC-BY 4.0).

The framework was then further utilized by Armour et al. [934] to investigate effects of post-TEVAR morphological features on false lumen thrombosis. Three patient-specific cases were considered where CT image data were employed to create models immediately after surgery and then annually for up to three years. The CFD model including thrombus formation was then applied to predict thrombosed regions based on the initial configurations and compared with the actual outcome at follow-up. For a representative patient case, Fig. 70(a) shows the flow velocity at peak systole, while Fig. 70(b) depicts the evolution of the lumen boundary during the modeling of thrombus growth. The high flow velocities in the branch vessels of the arch are noteworthy, appearing to be higher than in comparable studies; however, this is not further discussed in the study. With this follow-up data, the model was validated once again, yielding satisfactory agreement with discrepancies stemming from virtual occlusion of minor branch vessels only. The main goal of this study was to shed some light on the role of the distance from the entry tear to the first, more distal fenestration in false lumen thrombosis after TEVAR. Artificially altering the reconstructed flow domain, this distance was then adapted, yielding two additional virtual models. Comparing all these configurations, Armour et al. [934] concluded that a greater distance between the entry tear and the first, more distal fenestration led to lower false lumen flow rates, reduced TAWSS, and thus ultimately resulted in increased thrombosis of the false lumen, which was found to be consistent with clinical observations. This virtual case study therefore suggested a TEVAR design that took into account the local false lumen morphology and reduces false lumen perfusion by enlarging the region of zero or slow flow in the proximal false lumen. Consequently, this computational study also supported the current understanding that the introduction of additional fenestrations during stent-graft placement can prevent false lumen thrombosis.

Armour et al. [805] aimed to enhance the understanding of the role of branch vessels in aortic dissection by studying two patients with type B dissection from the ADSORB trial [935]. One patient was post-TEVAR, while the other was pre-TEVAR. Branching vessels have been demonstrated to reduce false lumen thrombosis and decrease the likelihood of false lumen dilatation, as discussed in Section 3.4. Their investigation encompassed all branching vessels in the original model, along with additional fenestrations feeding the false lumen in the modified model. One model included the inferior mesenteric artery, while the other featured intercostal arteries. The results revealed increased flow and flow disturbance around branch vessels, leading to a rise in WSS in local branch areas. Depending on the model, there was a 2 to 10% increase in WSS, with intercostal vessels experiencing higher WSS due to their larger diameters. This, in turn, reduced the likelihood of thrombus formation. Overall, small areas of thrombus formation deviated from follow-up observations, which may be due to non-patient-specific boundary conditions as inlet and outlet values were derived from the literature. Inclusion of branched vessels in the model also resulted in reduced pressure in the false lumen.

Based on these investigations, Armour et al. [931] continued to investigate the effect of false lumen perfused branch vessels. Two patient-specific models were considered including the inferior mesenteric artery or intercostal arteries, which were meshed in a semi-automatic fashion based on CT data due to their small radii of 1.6 and 2.0mm, respectively. In this study, boundary conditions were in part derived from the literature, since medical data were limited. Note here that the volumetric flow rates through the intercostal arteries were fixed at 5% of the inflow according to the literature. Then, a comparison of models considering or neglecting the false lumen perfused branch vessels was performed. Including the minor branch vessels, the authors revealed that the peak systolic flow velocity in the intimal tear increased by 13%, which leads to an increase of the volume-averaged TAWSS in the abdominal false lumen by 25.1%. Simultaneously, the true-to-false lumen pressure difference was not altered significantly. Including the inferior mesenteric artery in one of the cases led to a reduction in abdominal false lumen thrombosis of 15%, reducing the discrepancies between the simulation results and the one-year follow-up. This work thus confirmed anatomical studies on post-TEVAR false lumen thrombosis, which suggested that false lumen perfused side branches inhibit thrombus formation. This finding motivates further investigations on larger study cohorts.

In an initial study, Jafarinia et al. [936] examined the impact of hematocrit values on false lumen thrombosis using a 2D idealized geometry of a type B dissection, including an entry and an exit tear. The considered geometry was parameterized based on literature data, and a non-Newtonian fluid model incorporating hematocrit values was considered. Their findings indicated that elevated hematocrit levels hinder thrombus formation due to rheological factors, suggesting that patients with higher hematocrit values may have a reduced likelihood of experiencing complete false lumen thrombosis. They explicitly underlined a limitation of their study: it only considered a single geometry. Hemodynamics, defined by the interaction of flow and rheology and a primary driver of thrombosis, is significantly influenced by geometry. Thus, a different geometry might yield different results. The same consideration applies to hematocrit levels, or, to put it another way, the concentration of red blood cells. Subsequently, Jafarinia et al. [937] utilized the previously established geometry to explore the impact of morphological parameters on false lumen thrombosis, providing further insight into the factors influencing false lumen thrombosis. In doing so, the major limitation of the previous study, which was its use of only a single geometry, was addressed. With a fully automatic pipeline from geometry generation and CFD simulation including the thrombosis model and evaluation, a global sensitivity analysis of input parameters was performed. This sensitivity analysis included selected morphological parameters such as the size of the tears, the diameter and length of the false lumen, the diameter of the true lumen, and the location of the entry tear. The study also introduced dimensionless morphological parameters to generalize the results. To ensure sufficient representation of the input variability of the chosen geometry, the input sample space of the global sensitivity analysis was set to a dimension of 4,000. The sensitivity analysis revealed that the false lumen diameter and the size and location of intimal tears were the most sensitive parameters influencing false lumen thrombosis. A higher risk of partial thrombosis was observed when the false lumen diameter was larger than the true lumen diameter. Reducing the ratio of entry to exit tear size increased the risk of false lumen patency. These parameters played a dominant role in classifying morphologies into patent, partially thrombosed, and fully thrombosed false lumen. Although simplified morphologies of the dissected aorta were used, the results provide promising insight into the mechanisms underlying false lumen thrombosis in type B dissection. This study therefore highlighted the predictive role of morphological parameters for false lumen thrombosis in type B dissections, which is consistent with clinical observations.

Due to the time-consuming nature of the hemodynamics-driven model by Menichini and Xu [932], Jafarinia et al. [938] sought to simplify the model. They significantly reduced the number of components involved, making thrombus growth predictions 65% more efficient. This proposed model is based on a previous study by Melito et al. [939], who performed a sensitivity analysis of the original model [932], and revealed that not all parameters influenced thrombus formation equally, suggesting model could be simplified. In patients with type B dissection, thrombus formation was predicted with this simplified model in patient-specific geometries obtained one month after TEVAR and compared with the *in vivo* configuration observed three years later. The *in silico* predictions closely matched the actual clinical observations in terms of location and overall thrombosed volume, validating the model. Although the direct link to physical time is lost, as seen in related models, future work could potentially assist in estimating time correlations.

As discussed in Section 7.1, Chong et al. [885] developed the first FSI model that incorporated thrombus formation in a simplified geometry of type B dissection, which was based on the thrombus formation model of Menichini and Xu [932]. Despite knowledge of fluid–solid interaction, reducing flap mobility by increasing the Young’s modulus of the flap slows thrombus growth. In fact, when compared with the rigid model, the predicted thrombus volume is 25% larger using the FSI-thrombosis model with a relatively mobile flap, which highlighted the importance of incorporating thrombus formation in FSI modeling. Moreover, they reported vortices near the tears caused by drastic flap motion, which is an example of hemodynamic behavior only seen when considering FSI.

In the recent research by Wang et al. [940], the significance of low WSS in the initiation and growth of thrombus was explored. In the past, this aspect has been highlighted in many studies, but the consequence of thrombus breakdown due to increased WSS has been widely overlooked. To fill this gap, they proposed a novel WSS-induced thrombus breakdown function, which was integrated into the previously developed hemodynamics-based thrombosis model. The performance of the enhanced model was assessed by quantitative comparison with experimental *in vitro* data on thrombus formation [941] in a backward-facing step geometry. Moreover, a qualitative comparison with *in vivo* data from a type B dissection patient with post-TEVAR follow-up was performed. Remarkably, incorporating the aspect of thrombus breakdown considerably improved the accuracy in predicting thrombus volume. In instances like the backward-facing step geometry, this inclusion revealed that thrombus breakdown hinders its expansion over the step and downstream, thereby enabling a stable thrombus formation at a quicker rate. When compared with the original model that omits thrombus breakdown, the refined model showed a better alignment with experimental measurements in terms of thrombus volume, height, and length. Conclusively, their study underscores that the effect of thrombus breakdown warrants its inclusion in computational thrombosis models.

**Fig. 71.**
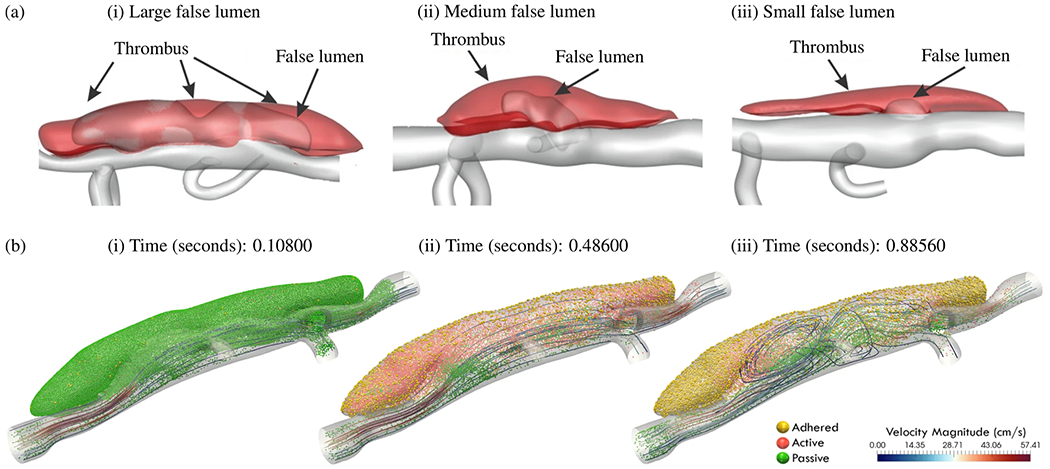
(a) Reconstructed geometries from 3D ultrasound and coherence tomography image stacks show dissections of the suprarenal aorta in *Apoe*^−/−^ mice: (i) large; (ii) medium; and (iii) small false lumen [469]. The red-shaded volume indicates the thrombus, the pink volume represents the false lumen with flowing blood, and the gray represents the patent vessel. Blood flow is directed from the proximal end (right side) to the distal end (left side). (b) Illustration of results showing three stages of particle distribution within the large false lumen at specific moments during the simulation: (i) initial particle arrangement; (ii) intermediate phase, which concludes diastole; and (iii) final phase during peak systole. Selected streamlines are shown, with their colors indicating velocity magnitude. The particle color codes are as follows: passive particles (green); activated or triggered particles (red); and active, adhered particles (yellow). For better visualization, not all particles are presented and the flow direction runs from left to right. Figure is adapted from Yazdani et al. [489] (licensed under CC-BY 4.0).

Despite the computational framework proposed by Menichini [533], and its recent simplification by Jafarinia et al. [938] and further extension to model thrombus breakdown by Wang et al. [940], several other frameworks have been employed to model thrombus formation in aortic dissection. One of which was proposed by Yazdani et al. [489]. They aimed to find out why some false lumina thrombose entirely, while others do so only partially or not at all. Using a data-driven particle-continuum model [942], they examined thrombus formation in a murine model of aortic dissection. This dissection was induced in adult male *Apoe*^−/−^ mice by a standard angiotensin II subcutaneous infusion at a rate of 1, 000ng/kg/min. The 3D geometric model was derived from multimodal imaging that integrated 3D ultrasound with optical coherence tomography. Moreover, the average flow velocities were determined using pulsed wave Doppler and anatomic 3D ultrasound [469]. They analyzed three types of aortic dissection that did not rupture: a large false lumen with minimal thrombus, a moderate false lumen with significant thrombus, and one filled primarily with thrombus (Fig. 71(a)). Their computational model incorporated the two-way coupling of Lagrangian particle transport with blood flow. They studied the motion of platelets within the flow and their adherence to the damaged dissection surface by merging the spectral/hp element method (SEM) [943] with a force coupling method, similar to the previous work of Pivkin et al. [944]. Drawing inspiration from previous research, platelets in their model were perceived to exist in one of three states: passive, triggered, or activated [944], see Fig. 71(b). Both passive and triggered platelets were classified as non-adhesive. However, when a passive platelet interacts with an activated platelet or a damaged luminal boundary, it was triggered and activated after a short delay. This activated state allows the platelets and associated fibrin to grow in size. This approach allowed them to use fewer platelets than were normally observed at physiological concentrations, while still simulating the transformation of the clot in size and shape upon interaction with injured walls or other platelets. Moreover, in their methodology they divided the thrombus formation process into two stages. Initially, a thrombus formed and developed in the false lumen, occurring minutes to hours after dissection. This was followed by the expansion and remodeling phase of the clot, which was primarily driven by fibrin degradation and collagen production, and could last from days to weeks. They focused on the acute phase, in which they thoroughly analyzed the platelet activation, aggregation, and formation of the primary fibrin network. The resulting numerical data emphasized the significance of both geometry and local hemodynamics in determining the acute progression of a thrombus. Even considering the inherent geometrical differences between murine and human dissections, mouse models are invaluable due to their consistency. Their results showed that false lumina with different geometries resulted in greater thrombus formation in areas with lower bulk shear rates. In contrast, regions with high shear or intense vortices were less favorable for the formation of dense thrombi.

Aiming to better understand the role of proteins in the coagulation cascade for thrombosis, Wang et al. [945], in their study, specifically focus on the activation and stabilization of platelets in type B dissection. They introduced a reduced-order fluid-chemical model to simulate the coagulation cascade in a simplified geometry of a two-year aortic dissection and investigate the driving mechanisms of false lumen thrombosis. The coagulation cascade model built on the work of Bodnár and Sequeira [946] and is an extension of the model originally proposed by Anand et al. [947,948]. The model demonstrated a consistently high level of fibrin at the top of the false lumen and in some time-varying areas between the two tears, suggesting a high probability of thrombus formation at these locations. Furthermore, the model showed that the temporal evolution of coagulation factors is strongly influenced by local hemodynamics, with periodic changes in the high disturbance zone aligning with the flow field.

A novel approach to modeling thrombus formation was proposed using a computational model for blood stasis, see the work of Jiang et al. [949]. This model employed the two-fluid principle to monitor the spatial distribution of residual blood over time. The focus of the study was four patients with type B dissection who underwent TEVAR and had persistent false lumina. Two years post-TEVAR, follow-up *in vivo* observations were conducted. In contrast to previous modeling approaches [932], in this model a specific point in time for a fully developed hemodynamic flow field was identified. ‘New blood’ was then defined as the inlet substance that had similar physical properties to the existing ‘old blood’, such as density and viscosity, which corresponds to the classic two-fluid or phase model. The volume of fluid surface tracking technology was used to solve the hemodynamic field with two fluids. This made it possible to track the location and shape of the new and old blood interfaces over time by resolving the volume fraction equation in each computational cell, thereby identifying the location of remaining old blood and the area of blood stasis. Their findings revealed that the remaining false lumen was located below the highest tears and its span was closely associated with the distance between tears. Due to the size and location of the tears, a high probability of thrombosis was suggested if a majority of the blood remained in the false lumen. Importantly, the model-predicted positions and topologies of residual blood in the false lumen showed a strong correlation with the *in vivo* observations of thrombus.

On the basis of the theory of porous media, Gupta and Schanz [950] recently presented an approach to modeling thrombus growth and formation in aortic dissections using a multiphasic framework. This macroscopic continuum mechanical approach provides a tool to describe the complex microstructure of thrombi. This was achieved by defining a representative elementary volume in which individual constituents – solid, liquid, and nutrient-rich phases – are considered to be in a state of ideal disarrangement. Through averaging processes over this representative elementary volume, the microscale information of the overall aggregate and its constituents was homogenized into macro-scale quantities. Generally, the thrombus growth process is driven by chemical, mechanical, and metabolic factors. Due to the multiphasic nature of the thrombus, they presented a triphasic model that integrated a solid phase saturated by fluid, with the fluid itself comprising both liquid and nutrients. Since the effects of blood velocity and nutrients on thrombus growth were well described in the literature, they presented a velocity and nutrient concentration-induced thrombus growth model based on the theory of porous media. After outlining the theoretical framework and its implementation, they applied the developed model to a 2D cross-section of a false lumen derived from patient-specific geometry and used simplified boundary conditions. This model emphasizes that thrombus formation occurred in regions of reduced blood velocities. Material parameters have been proven to play a central role, as several parameter studies have shown. In conclusion, although their findings are promising, further advances are of pressing need, especially with regard to material parameters and boundary conditions, to gain clearer insights into thrombus formation and growth based on this multiphasic approach. Nevertheless, their work represented a novel and intriguing method for modeling thrombus growth and formation in aortic dissection through the theory of porous media.

In summary, several studies over the past decade have modeled thrombus formation using both simplified and patient-specific geometries. Most of these studies employ a hemodynamics-based approach [932], which has demonstrated simulation results that agree well with medical data. However, this method remains computationally demanding. Notably, a recent model reduction study further optimized this shear-driven thrombosis model and significantly improved computational efficiency by reducing computation time by 65% in a representative example [938]. The currently available thrombus formation models exhibit good agreement with medical data, but the computational requirements are typically higher than for fixed-grid CFD approaches.

### 8.4. Key findings and publication index

To summarize this section, we present the publication index of *in silico* models on selected topics in Table 9, with a focus on CSM simulations of arterial tissue stress, the deployment and migration of stent-grafts, and simulations of thrombus formation in aortic dissections. Due to the large number of studies available, only a selected range was discussed; however, this table covers an even broader range of studies. Essential information about the modeling approaches and patient cases as well as the objectives of each study is included. Finally, the most important findings are listed conclusively.

- In patient-specific aortic dissection, localized stress concentrations, particularly in the ascending aorta and aortic arch, may be associated with the locations of intimal tears.
- TEVAR procedures increase wall stress in the narrowed true lumen, but at the same time reduce it in the entry tear area, where false lumen dilatation typically occurs. Moreover, an analysis of varying stent-graft lengths indicated that the location of maximum wall stress shifts with the length of the stent-graft. In general, CSM models are effective in capturing stent-graft positioning in patients undergoing TEVAR.
- Current thrombus formation models can provide good predictions and high accuracy in predicting thrombus formation for patients. The likelihood of thrombus formation is elevated due to factors such as the absence of an exit tear, a larger distance between the entry tear and other fenestrations, a larger false lumen diameter, and smaller intimal tears. However, the interplay between these factors and possibly many more associated with thrombus formation is complex and not yet fully understood.

## 9. Conclusion and future perspectives

### 9.1. Conclusion

The previous sections highlighted the extensive research efforts undertaken to uncover the pathological changes of aortic dissection using various approaches. As demonstrated, well-constructed multiscale material and *in silico* models can serve as versatile tools for bioengineers and clinicians, allowing them to explore unsolved problems. This contributes to a deeper understanding of aortic dissection, particularly with regard to specific phenomena.

**Fig. 72.**
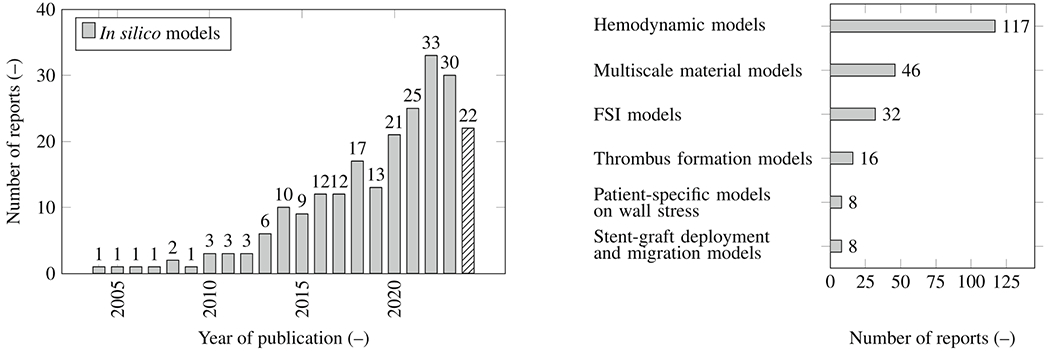
Annual number of reports on multiscale materials and *in silico* models incorporating patient data related to aortic dissection (total 227 reports). The data for the year 2024 (hatched bar) remains incomplete.

In Section 2, following a brief overview of the biomechanics of the normal aorta – including details of anatomy and wall micro-structure – an introduction to aortic dissection was provided. This involved a definition of the disease, commonly used classification systems, a summary of modern imaging techniques and treatment methods for aortic dissection patients, and aortopathies related to aortic dissection. The purpose of this section was to familiarize the reader with the fundamental aspects of aortic dissection. We then presented medical data focusing on the pathological remodeling of aortic dissection, including both the anatomy and the wall microstructure of the aorta in Section 3. This included an examination of factors such as the extent and configuration of the dissection flap and the dilatation and growth of the aorta. Furthermore, the microstructure and mechanical behavior of the diseased aortic wall were described. The section concluded with a discussion of altered hemodynamics in the true and false lumina, including blood flow patterns and local pressure throughout the cardiac cycle, as well as the role of thrombus formation. Subsequently, experimental models for aortic dissection were discussed in Section 4, including *in vivo*, *ex vivo*, and *in vitro* studies on animal, human, and phantom models. These models have played an important role in uncovering biomechanical mechanisms, particularly in understanding the hemodynamics during deterioration of aortic anatomy, microstructural changes, and evaluating the effectiveness of medical implants. Section 5 then focused on multiscale material models for damage and failure. These models were developed to model wall delamination, dissection propagation and flap configuration, the effects of focal areas of medial degeneration, and growth and remodeling in the newly formed tissue layers. The following three sections provided an overview of *in silico* models of hemodynamics (Section 6), FSI (Section 7), and selected topics (Section 8). To the best of the authors’ knowledge, this is an almost complete synthesis of the literature in this area. Each section contains key findings and the publication indices for the available models. To provide readers with a comprehensive overview of publication trends in recent years, the number of reports on multiscale material models on damage and failure and on *in silico* models using patient data on aortic dissection is presented in Fig. 72. The trend underscores a significant increase in the total number of models, especially in the last decade. Particularly in recent years, the number of reports on *in silico* models, particularly those addressing hemodynamics in patient-specific models of aortic dissection, has increased rapidly.

### 9.2. Future perspective

#### 9.2.1. Disease-related modeling aspects

**Fig. 73.**
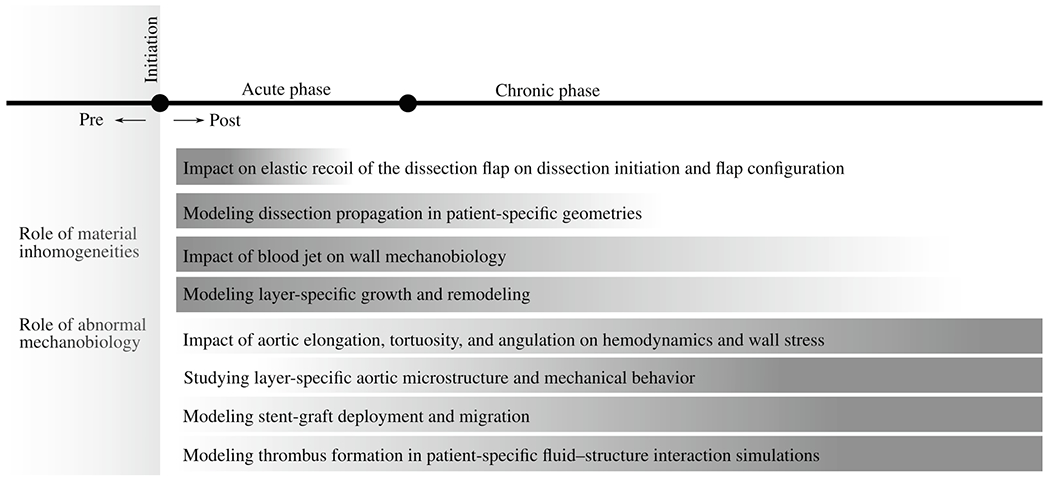
Visualization of future perspectives in disease-related modeling with respect to aortic dissection. The monochromatic coloring scheme illustrates the importance of the specific aspects during the acute, subacute, and chronic phases, and their progression over time. For visualization reasons, the hyperacute and subacute phases are neglected.

Considering future developments, it is clear that there are numerous challenges and unresolved problems in the field of disease-related modeling of aortic dissection, particularly with regard to the key factors that contribute to the onset and progression of aortic dissection. It is noteworthy that these factors often dominate in different stages of aortic dissection, from the acute to the chronic phase, as shown in Fig. 73.

As discussed in previous sections, given the remarkable robustness of a normal, healthy aorta, an abnormal aortic wall is likely a major factor in triggering aortic dissection. Multiscale material models have been developed to improve our understanding of specific pathological changes in aortic dissection. In fact, pathological changes in aortic dissection, as in other diseases, cannot be attributed to a single alteration, but rather are correlated [717]. For example, the accumulation of GAGs can cause swelling pressure that can lead to the degradation of interlamellar, radially-oriented elastic fibers. Consequently, SMCs connected to elastic lamellae by elastic fibers could lose their microstructural integrity, which could lead to apoptosis and dysfunction [49]. Although the exact mechanism is not yet fully understood, it could be crucial to couple and investigate these and similar mechanisms. Against this background, the inclusion of mechanobiology and related pathological changes in mechanical models could also be of great importance, especially since none of the material models presented and discussed so far have taken this aspect into account. In this context, central areas of medial degeneration, in other words local material or geometrical inhomogeneities that arise or develop due to pathological changes, should be further investigated for their possible contribution to the initiation and progression of aortic dissection. It is truly fascinating to study the influence of local GAG accumulation [103] or the degradation of elastic fibers [717] on the material behavior at the macroscopic scale in patient-specific geometries. The raises the question of whether these localized pathological changes may accumulate over time, ultimately and triggering aortic dissection, potentially explaining why certain pathological events, such as the formation of intimal tears, tend to recur in specific region. Moreover, as recently highlighted by Myneni and Rajagopal [114], even the normal, healthy aortic wall exhibit significant inhomogeneities that must be considered. While it may be difficult to obtain this information from the entire aorta through traditional microstructural investigations such as second harmonic generation microscopy, future advances may enable non-invasive acquisition using, e.g., MRI imaging [117,118].

From the initial onset to the acute phase and possibly the chronic phase, aortic dissection typically propagates over time. However, most of this propagation usually occurs early or immediately after the hyperacute phase. The progression of an aortic dissection can vary depending on the specific geometry of the aorta and the location of the intimal tear, with antegrade movement being more common, but retrograde movement also occurring in some cases. In certain cases a pronounced helical pattern may be observed, but in others it is not. Using *in silico* models, these variations can be comprehensively studied, and in the near future, patient-specific geometries could also be examined, initially through the use of CSM models. Different geometries could be explored, such as variations in the branch vessel, tortuosity, or unusual geometrical features. Furthermore, the role of the microstructure, particularly the alignment of collagen fibers along the aorta, can be further investigated.

In addition to axial propagation, the aortic dissection or false lumen also extends circumferentially. The circumferential propagation of the false lumen occurs primarily at the onset of the disease and typically progresses only slowly thereafter. In fact, the circumferential extent of the false lumen varies among patients. We hypothesize that the elasticity of the aortic wall, particularly within the intima and media, plays a pivotal role in circumferential propagation. Localized defects in the aortic wall, such as small initial tears or local separations due to accumulated GAGs, may result in delamination of the aortic wall if the interlamellar strength between layers is compromised. Subsequently, the wall undergoes elastic recoil, releasing the stored elastic energy in the process. The segment of the aortic wall that becomes the dissection flap may tend to return to its stress-free configuration to relieve tension and allow blood flow into the false lumen. Consequently, the elasticity of the aortic wall could determine the circumferential extent of the false lumen, with a small newly formed false lumen in very stiff aortic walls, as in older adults, or the false lumen covers (almost) the entire circumference of the true lumen in very elastic aortic walls, as in young adults. Nevertheless, further research is necessary to evaluate this hypothesis and to fully understand the correlation between aortic wall elasticity and the extent and associated configuration of the dissection flap.

At dissection initiation, one or more intimal tears typically form. With each cardiac cycle, blood is pushed through these tears into the false lumen and flows back. This process creates a flow jet and a pressure difference between the lumina, both depending on the size, location, and number of tears. The role of this flow jet and the pressure difference remains a controversial topic, particularly regarding how both factors evolve from the onset to the chronic phases. While the flow jet is often considered a factor in dissection progression, it is uncertain whether the local increase in pressure can further delaminate the aortic wall. A recent computational study [612] demonstrated that the flow jet induce a local increase in WSS and pressure on the aortic wall. However, there is no definitive evidence to suggest that the flow jet is sufficiently strong to initiate aortic wall delamination. Instead, local wall remodeling may occur as the disease progresses. In contrast, the pressure difference between the lumina can have a significant impact on dissection, particularly the pressure in the false lumen, which can pull the outer wall away from the aorta and promote delamination. In addition, the cyclic pressure difference between the lumina may further aggravate progression of the dissection. Further investigations are required to better understand these phenomena.

Next, we would like to emphasize the importance of considering the growth and remodeling of the dissected aortic wall throughout the progression of the disease from onset to the chronic phase. This aspect has received little attention in the context of aortic dissection, as shown in Section 5.4. Growth and remodeling frameworks have recently received considerable attention in the field of cardiovascular modeling in general [954]. As already mentioned, in aortic dissection, the aortic wall can be divided into three distinct layers, namely the outer wall of the true lumen, the dissection flap, and the outer wall of the false lumen. However, the microstructural composition of these layers varies depending on the individual patient case. In the event of a delamination between the media and adventitia, it may happen that the outer wall of the false lumen consists only of the adventitia, but can also include parts of the media or even the entire media. Other geometrical factors may also play a crucial role, but these remain to be determined. Furthermore, these layers may remodel over the course of disease and during the transition from the acute to the chronic phase. This remodeling may be due to changes in the mechanical stress of the respective tissue layers, which trigger remodeling processes, or due to inflammatory processes resulting from the delamination-induced injury response. In fact, regions of altered, usually increased WSSs are generally associated with remodeling of the aortic wall, leading to altered mechanical behavior, particularly with greater elastin degradation. Ultimately, this often results in local wall dilatation, which increases the risk of rupture. Evidence for this has been shown in both in clinical studies using advanced medical image techniques and in computational studies of patients with aortic aneurysms [422,736,955–957], bicuspid aortic valves [958–960], aortic stenosis [961], or intracranial aneurysms [962–966], among others.

As the disease progresses, the dissection flap stiffens, and the outer wall of the false lumen thickens, as discussed in Section 3.2. Moreover, the outer wall of the false lumen becomes more compliant under the prevailing luminal pressure, leading to dilatation of the false lumen. However, it remains largely unknown how these different regions remodel and what microstructural changes occur. Nevertheless, understanding these changes could potentially enhance patient treatment, as the timing of surgical intervention often depends on remodeling [283–285]. In short, while increased stability could make TEVAR safer, it is important to note that a dissection flap in its most pliable state offers the greatest opportunity for complete remodeling, see Sections 3.1 and 3.2. Growth and remodeling models have been applied to many aspects of vascular disease [237], but there remains an urgent need to consider these effects in dissection.

To date, aortic elongation and its relationship to aortic dissection have not been extensively studied. The connection between an elongated aorta – a condition often associated with aging – and the onset and progression of the disease remains however unclear [967]. This is especially true considering that elongation can also result in pronounced angulation and increased tortuosity. It remains to be determined whether the disease causes aortic elongation or if elongation itself contributes to the onset and progression of the disease, or, in other words, whether it develops during the chronic phase. Computational models can be a versatile tool to study the influence of axial growth on dissection progression. Studies have indicated that the ascending aorta and the aortic arch elongate, affecting not only hemodynamics but also wall stresses. In addition, as highlighted by Poullis et al. [968] and later discussed in [969], aortic elongation can occur even without dilatation. Their study, which was based on a mathematical model, suggested that aortic curvature may be a more critical factor leading to aortic dissection than aortic diameter, blood pressure, and other factors. The greater aortic curvature may explain why normal diameter aortas can dissect and also suggests that the location of the entry tear may be predictable, although this has not yet received much attention. Moreover, with respect to vessel tortuosity, it has been shown to be related to other diseases, e.g., tortuosity is a well-documented challenge in coronary arteries [967,970]. There is strong evidence of a causal relationship between the tortuosity of carotid arteries and stroke [971] or connective tissue disease [972]. In addition, computational studies have been developed to study vessel tortuosity, but there are only a few, see, e.g., [346,973].

For the development of novel material models, access to experimental data from mechanical tests and microstructural examinations on both healthy and diseased tissue is crucial. Although some experimental data are available, they are still insufficient, as briefly summarized in Section 4. In particular, there are insufficient data on the mechanical behavior, dispersion, cross-linking, and alignment of fibrous constituents in the different tissue layers: outer wall of the true lumen, dissection flap, and outer wall of the false lumen. As discussed, these regions undergo remodeling as the disease progresses. Therefore, it is crucial to understand and characterize the changes in individual tissue layers that accompany the disease phases from the acute phase to the chronic phase. However, it may be challenging to collect representative samples for specific phases because the parameters are different for each patient and it may be difficult to obtain a sufficient number of donor samples. Animal models could be of great use here and provide valuable insights into the progression of the disease. Therefore, it might be more appropriate to compare the normal aorta with the different tissue layers in the chronic phase when the aortic wall has remodeled and is mostly stable. Additionally, conducting experiments at various locations along the aorta could be valuable because the aortic wall near the aortic arch differs from more distal areas, causing dissections to often occur at this location, as discussed in Section 3.2. In the future, such information could be obtained using MRI imaging [117,118]. In fact, it would be advantageous to derive mechanical and microstructural data from experiments on diseased tissue and integrate this information into advanced material models. Moreover, novel advanced experimental methods can improve the characterization of aortic mechanical properties in health and disease [974]. Full-field optical measurements [470], including digital image correlation (DIC), digital volume correlation (DVC), and the combined use of optical coherence tomography with digital volume correlation (OCT-DVC), to visualize and quantify intramural strain distributions under similar conditions to those in aortic dissection, can significantly enhance our understanding of combined mechanics and microstructure.

In the chronic phase, significant false lumen dilatation often occurs, which increases the risk of rupture. Therefore, stent-grafts are frequently deployed in complicated patient cases. Not only do stent-grafts change the local stress distribution, stent-grafts that are oversized, undersized, or incorrectly placed can also migrate over time. Recent studies have shown that stent-grafts can even affect the microstructure of the aortic wall due to the substantial local pressures, leading to aortic wall remodeling [975,976]. Furthermore, as outlined in two subsequent studies by Suh and his colleagues [977,978], graft-to-arch angulation increased due to cardiac pulsation and respiration, likely resulting in additional local stress concentrations caused by the deployed stent-graft in the aortic wall, an aspect not typically considered. This is an important area with high potential. Newly designed stent-grafts can be integrated into *in silico* platforms, allowing various conditions to be tested without the need for costly animal and human trials typically required for regulatory approval. Therefore, this particular area is considered a promising application of computational biomechanics with potentially great impact on the clinical practice of patients with aortic dissection.

Finally, we would like to address the role of thrombus formation in aortic dissections, which predominantly occurs in the chronic phase. The currently available models examine thrombus formation in the false lumen exclusively from a fluid dynamics perspective – in particular, they examine the effect of thrombus on blood flow. Typically, these models are validated by individual follow-up cases. However, they only capture part of the overall picture because the important mechanical role of the thrombus in aortic dissection is largely overlooked. Consideration of thrombus formation within the FSI modeling in patient-specific cases is crucial for further validation of these models. This examination could provide valuable insights across various patient profiles, particularly with regard to the use of stent-grafts. Although deployment should theoretically enhance thrombus formation, movement of the flap could potentially impact thrombosis. As mentioned previously, the formation of a thrombus could seal the false lumen, thereby contributing to the patient’s recovery. This situation often leads to wall remodeling, a process that requires further investigation due to its complexity. From a mechanical perspective, the intraluminal thrombus appears to play a protective role by limiting biomechanical wall stress due to increased wall thickness [979]. However, this also creates a highly proteolytic and oxidative environment. In the aorta, where blood pressure is high, radial convection pushes the proteolytic and oxidative components of the intraluminal thrombus into the wall, potentially contributing to progressive dilation and rupture risk. The combination of low velocities and oscillatory flow within the aneurysm leads to flow stagnation and promotes deposition of fibrinogen and circulating cellular elements such as leukocytes, platelets, and red blood cells in the intraluminal thrombus. Enzymes and other components released by these aggregated cells are transported outward to the wall, contributing to extracellular matrix degradation [980,981]. Conversely, if diffusion from the lumen into the wall is hindered by a thrombus, important questions arise about the nutrient supply to the wall. This scenario could lead to possible hypoxia, similar to that observed in aortic aneurysms [566]. To study these effects, the application of multiscale material models and the inclusion of mechanobiology could prove useful. When considering patient-specific aspects, the exclusive use of solid mechanics or multiphase approaches can provide insightful information. For example, the bio-chemo-mechanical impact of intraluminal thrombus deposition on arterial tissue growth and remodeling was investigated using the constraint mixture theory in aortic aneurysms, as reported by Virag et al. [982]. Readers are also referred to the related studies mentioned therein. A multiphasic approach to aortic dissection based on the theory of porous media [950] considers both solid and fluid phases. This approach has already been discussed in Section 8.3. However, their work mainly focuses on the theoretical framework and demonstrates limited applicability. Because these approaches are based on CSM models, they do not explicitly consider blood flow, which significantly reduces complexity. However, for some research questions, considering blood flow can be extremely useful.

#### 9.2.2. Numerical challenges and clinical applications

Computational modeling and simulation can be utilized in medicine to simulate and enhance understanding of pathological processes, assist in the diagnosis and treatment of patients, support decision-making in complex clinical cases, and evaluate the safety and efficacy of medical products, commonly known as ‘*in silico* medicine’ [983,984]. Additionally, the term ‘*in silico* trial’ was coined to describe the use of computer modeling and simulations to assess the safety and efficacy of various medical products, including drugs, medical devices, and diagnostic tools. Typically, these are obtained through controlled experiments carried out *in vitro*, *ex vivo* or *in vivo* on animals or humans, with multiple clinical trials being carried out with increasingly larger numbers of participants [985–990]. *In silico* trials aim to reduce, refine, or replace these experiments. For this purpose, digital twins are being developed [991,992] – a digital replica or virtual model of a patient that faithfully mirrors reality in real-time – and the creation of synthetic data and virtual cohorts [993–996] are of great importance, with a recent publication presenting the first synthetic aortic dissection surface meshes containing two flow channels [997]. These models provide predictive insights and also leverage machine learning techniques and reasoning to aid decision making. In recent years there has been great interest in such predictive *in silico* models, which are emerging as new methodologies for the development and regulatory evaluation of medical products, as evidenced by the numerous published studies and initiatives [984].

In general, there is broad consensus on the potential advantages of *in silico* models. In the United States, the first guidelines for conducting and reporting verification and validation [998] and for assessing the credibility of *in silico* models used in medical device submissions [999], have been published by the American Society of Mechanical Engineers (ASME) and the US Food and Drug Administration (FDA). There are also initial initiatives in Europe that advocate and promote the use of *in silico* modeling to close the gap between biomedical research, industry, and regulatory bodies. These initiatives play a crucial role in shaping policy and regulatory approaches to *in silico* methods in healthcare and highlight the value of computer modeling and simulation in reducing the time, costs, and risks associated with the development and regulatory approval of new devices. Despite consensus on the potential benefits and the existence of initial methodological frameworks, there is still a lack of general acceptance and guidance for researchers, manufacturers, or notified bodies on how to use these approaches to generate evidence. Such evidence is necessary to obtain market authorization, known as certification or qualification, from competent authorities. The first step in certifying a new methodology is to precisely define the so-called ‘Context of Use’ [998]. It is a clear and concise description of the specific conditions and purposes for which a particular methodology, technology or product is intended to be used. In general, each new certification must be granted for each specific Context of Use to determine the required level of credibility. A comprehensive definition of all possible Contexts of Use for all *in silico* trial methodologies is still lacking, representing a challenge that must be overcome [984,987].

While these problems are discussed in the literature, this review article highlighted the existing models and numerical approaches specifically applied to disease-related aspects of aortic dissection. In particular, it addressed the inherent complexity of aortic dissection and the numerous numerical challenges that must be overcome to utilize such models for patient care. Therefore, we focused on the numerical challenges associated with modeling aortic dissection, particularly when incorporating patient-specific medical data, and their potential for clinical application. Although the disease-related modeling aspects discussed previously are specific to aortic dissection, the numerical aspects examined in the following are not only applicable to modeling of aortic dissection but also relevant to related biomedical applications. Nevertheless, it is crucial to recognize the current challenges from a numerical perspective and to raise clinicians’ awareness of the current limitations in modeling and simulation of aortic dissection. This is particularly crucial because clinicians and engineers generally do not speak a common language and consistent terminology is not always established [1000]. Therefore, knowledge transfer can be challenging and lead to mutual issues going undetected.

There are numerous computational models in the literature that simulate blood flow or FSI in dissected aortas. Many of these presented models incorporated patient-specific data to reconstruct 3D geometry or obtained realistic boundary conditions, but most were still based on simplified constitutive relations such as hyperelastic solids or Newtonian fluids. There is currently no clear agreement on the importance and concrete influence of the involved (sub-)models and the simplifications made: while some studies concentrated on increasingly advanced fluid models for blood flow, others focus on boundary conditions, geometric aspects, or tissue models. Sensitivity analyses may help to identify and characterize a hierarchy of influencing factors when modeling and simulating flow phenomena in aortic dissection, also in the context of FSI and coupled tissue models. Moreover, modeling of the essential aspects of thrombus formation in aortic dissection remains rare, and detailed comparisons of available approaches are scarce in the literature due to the almost overwhelming computational demand despite advanced numerical tools. Although there were some comparisons in terms of model assumptions, combining multiple subjects into a single computational framework, such as FSI simulations with thrombus growth, remains a challenge. It is also worth noting that the integration of initial tissue rupture, FSI, and patient-specific geometries in one framework is still beyond the current state of the art. This remains a challenging topic given the inherent numerical complexity and the difficulties in transferring traditional high-performance numerical methods to clinically relevant scenarios.

When generating spatial discretizations, i.e. computational grids for FSI or CSM simulations in patient-specific cases, vessel wall thickness is an essential component because it is directly related to the calculated wall stress. However, this aspect has received only limited attention, typically assuming constant thickness, see, e.g., [448,612,868,869,894], and many others. One of the challenges in determining wall thickness is that conventional CT and other imaging techniques available for clinical use are often unable to extract this information, despite being the primary source of medical data for computational models. Not only can the wall thickness of different regions vary, it also undergoes remodeling and may become thicker as the disease progresses, as previously discussed. Wall thickness also varies locally and the overall aortic wall thickness of the aorta decreases from the proximal to the distal part [52]. Nevertheless, the inherent complexity of creating patient-specific models with varying wall thicknesses represents a significant challenge, especially given the geometry of aortic dissection, which is characterized by multiple lumina and a dissection flap. Currently, no computational model of aortic dissection exists that does not assume a uniform distribution of wall thickness. However, recently a model has been established that has a different wall thickness for different layers: the outer wall of the true lumen, the dissection flap, and the outer wall of the false lumen [342]. This simplification leads to substantial uncertainty with *in silico* models, which must be taken into account when evaluating their reliability.

The selection of appropriate rheological or turbulence models is a topic frequently discussed in the literature when conducting fluid flow simulations. The choice depends heavily on the specific topology and existing flow conditions. A recent example demonstrates the influence of the choice of rheological model on computed WSS values in aortic hemodynamics [1001]. In particular, the uncertainty of the rheological parameters was considered and how unavoidable errors in the model parameters lead to errors in the observed quantities. The suitability and differences between laminar and turbulent flow as well as rheological modeling were discussed on a case-by-case basis, see, e.g., [759,869,936], which precluded a general statement at this point. Typically, the computationally expensive options are excluded because the computational effort in patient-specific scenarios already reaches the limits of modern computing systems.

The influence of surrounding structures is often underestimated, but can have a significant influence on the predictability of computational results. The aorta is connected to other organs and supported by tissue, as described in Section 2.1, but this is typically ignored or integrated by exterior pressure, as in [868,869], embedding elastic continuum, or condensed zero-dimensional models with uniform or spatially varying parameters, see, e.g., [448,889,892,1002], among others. From a mechanical point of view, it is then modeled as a system of springs and dashpots acting on the exterior of the vessel. Additionally, pressure in the abdominal cavity is poorly understood, let alone described as a function of the respiratory cycle, or considered to vary spatially. In fact, locally different resistances and their dependence on time could have an enormous effect on the observed stresses and the deformation state in the dissected aorta. As a result, the tissue response can change significantly, if used improperly. Therefore, one cannot simply match the measured *in vivo* displacements with tissue stiffness without taking external tissue support into account. Studies have also identified heterogeneous circumferential strains *in vivo* [1003], suggesting that certain regions of the aorta may exhibit restricted expansion, while others demonstrate greater flexibility. This demonstrates that the surrounding tissue is not homogeneous; it varies both along the length and around the circumference of the aorta. Therefore, a uniform external tissue support does not accurately represent the *in vivo* conditions.

Similar challenges exist in translating the stresses present at the time of imaging into patient-specific geometries, where one may distinguish between approaches that recover a stress-free configuration [1004,1005] or integrate pre-stress into the balance equations [890,1006,1007]. The loads occurring during the diastolic phase, commonly referred to as pre-stress, should be included in the computational model, which makes the FSI approach more complex and often requires significantly more numerical effort. Without strict consideration of pre-stress, using more advanced tissue models may not produce more realistic results compared with linear elasticity.

When creating patient-specific models of aortic dissection, spatial discretization using finite element or finite volume grids plays a critical role in the subsequent approximation of the solid or fluid phases, or both. Especially when the dissection exhibits an intricate topology, for example, when the false lumen includes not only the aorta but also several aortic branches, it becomes increasingly difficult to create a computational grid with sufficient quality and good approximation to the real geometry. Undesirably small finite elements or unfavorable aspect ratios are possible consequences if the complex geometries are even suitable for automatic mesh generation. This can affect approximation quality and simulation time in multiple ways: (i) poor finite element quality reduces the solution quality and performance of the linear solver; (ii) under normal stability conditions, small finite elements may require more time steps; and (iii) finite elements that are too small can introduce a large number of undesirable degrees of freedom. Particularly if a layer-specific model is desired, the existing thin tissue layers make mesh construction even more difficult. Semi-automatic algorithms that combine manual segmentation and unstructured meshing are usually used for mesh construction, see, e.g., [1008,1009]. Alternatively, structured meshing approaches can be utilized [1010–1012]. For example, Bošnjak et al. [1013] introduced a fully automated approach for generating structured higher-order hexahedral meshes of healthy blood vessels, which was recently adapted for application to dissected geometries [1014]. This method converts CT images into high-quality finite element grids of arbitrary order including boundary layers. The spatial resolution can be adjusted as coarsely as desired, which significantly shortens computing time. However, an extension to the solid domain is not yet available.

In the context of the finite element mesh, it is necessary to use a local coordinate system, for example, for each finite element, to take into account the anisotropic material properties of the aortic wall. This is crucial for describing the alignment of collagen fibers, which, as previously mentioned, serve as primary load-bearing constituents in the aortic wall. Within the framework of a local coordinate system, changing fiber alignment during tissue remodeling can be characterized. However, the presence of two lumina and the dissection flap in between pose challenges that require consideration of rule-based methodologies [1015], methods based solely on structured meshing, or hybrid approaches that additionally incorporate wall normals [892]. Furthermore, when calculating a local coordinate system, due to the discontinuity of the intimal tears in the flap, there may be unphysical local fiber orientations that must be taken into account.

Another limitation of currently published studies is that they are not applicable to the general population, as most studies focus on a single patient-specific case, mainly due to the still significant numerical effort involved in patient-specific simulations. Closely related to model selection and the associated complexity is simulation processing time, which ranges from seconds when reconstructing pressures from a specific velocity field in a specific geometry, to hours or days when performing patient-specific simulations with complex constitutive models and accounting for FSI. In order to increase clinical relevance, these times must be significantly reduced. In this context, high-performance tools are the key technology to enable parametric studies on virtual cohorts, virtual surgery, digital twins, and the construction of large data sets, thus creating a solid basis for machine-learning methods. Only when a clinically relevant runtime is reached can computational models be effectively used in clinical practice, which motivates the development of tailored numerical tools. The practitioner is then no longer limited to simplified model assumptions, such as simple material models, neglect of vessel compliance, or even the consideration of 1D wireframe models, which enormously limit the predictive power of the simulation.

**Fig. 74.**
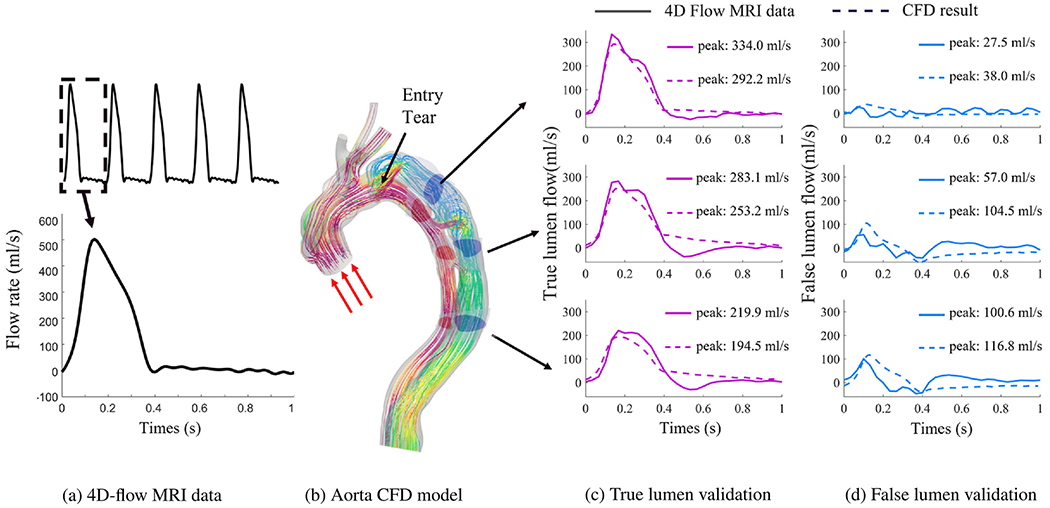
Comparison and validation of 4D-flow MRI data and CFD simulations in a 74-year-old male patient with residual type B dissection. (a) Pulsatile flow waveform across cardiac cycles acquired by 4D-flow MRI. (b) CFD model with emphasis on visualizing flow patterns through streamlines and location of entry tears. (c, d) Flow data derived from specific locations within the true and false lumina, indicated by arrows. Dotted curves represent CFD simulation results, while solid curves represent 4D-flow MRI results. Overall, the correlation between the two results is stronger for the true lumen, with the deviations being more pronounced in early diastole. The highest observed peak flow differences are approximately 12.5 and 83% for the true and false lumina, respectively. Figure is reprinted from Wang et al. [29] with permission from Elsevier.

The majority of calculation models are often insufficiently validated or not validated at all. Nevertheless, validation of the combined modeling assumptions for highly complex coupled multiphysics or multiscale models remains a central topic. In particular, Sections 7 to 9 illustrate various *in silico* models that have undergone validation, often using 2D or 4D PC-MRI data as ground-truth for flow simulations. The reader interested in further information is referred to Wang et al. [29], who provided a concise overview of validation results by comparing computational results with *in vivo* data from 4D-flow MRI in relation to aortic dissection models. A representative validation result is presented in Fig. 74. At this point, the importance of the inflow and outflow boundary conditions must be pointed out again. In order to achieve sound model validation, high-quality *in vivo* data for inflow and outflow must be considered. While 2D and 4D PC-MRI are most commonly used to determine inlet boundary conditions at the beginning of the ascending aorta or just above the arch, they are also typically used for outlet conditions [805]. However, because PC-MRI cannot provide the traditionally required pressure data, studies often rely on data from the literature. When PC-MRI data are available at the outlet, they can provide flow rates for fine-tuning individual Windkessel parameters [772], see, e.g., [448,773,803,820]. The clinical value of these models could be substantially enhanced by placing more emphasis on validation. Unvalidated models significantly reduce the predictive value of time-consuming simulations.

Conversely, in solid-domain validation, it is often difficult to validate the wall and flap motions obtained derived from the *in vivo* displacement, which is typically obtained from cine MRI. In some cases, displacements of 8mm were reported [448], while in other cases only ~1mm were detected [447,869,872,900]. Depending on the selection of material parameters, external tissue support, or pre-stress, some simulations reflect relatively large displacements, while others reflect smaller displacements. There is little emphasis on relating these measurements to the actual stage of tissue remodeling. For instance, in chronic aortic dissection, small displacements of the dissection flap are often measured *in vivo*, rendering a stiffer material or CFD on a fixed grid applicable. Conversely, in an acute aortic dissection, the tissue may not yet be remodeled and elastic materials therefore produce results closer to the deformations *in vivo*. Therefore, the choice of the material model that reflects the inherent microstructure in the current state can be crucial. To date, the anisotropic material behavior of the aortic wall, especially the dissected aortic wall, has usually not been taken into account in patient-specific studies.

Moreover, incorporating MRI into patient-specific CFD or FSI simulations is still an evolving field for which no standardized method is currently available to ensure a seamless transition to clinical use [29]. Notably, artificial intelligence, including deep learning techniques, holds immense potential to facilitate the acquisition and segmentation of MRI [1016] and the pre-processing of CFD, including 3D reconstruction and meshing, as well as prediction of CFD results [1017]. Custom geometric models could be automatically created from medical images [1018,1019], paving the way for automatic mesh creation and quality evaluation [1020–1022], as well as simulation setups that includ boundary condition settings and post-processing [1023].

**Fig. 75.**
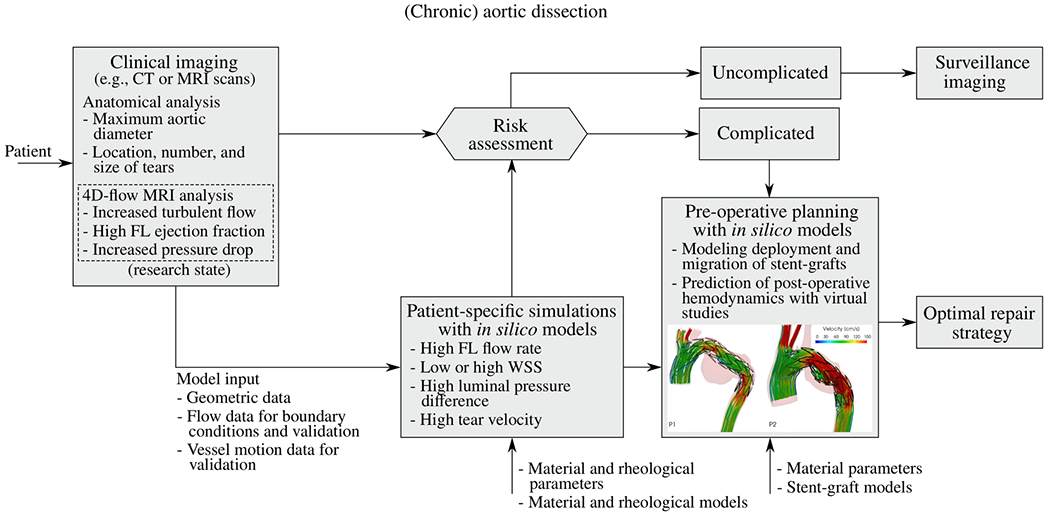
Possible workflow for personalized risk assessment and pre-operative planning supported by (validated) *in silico* models, based on Zhu et al. [1024] and Wang et al. [29]. The figure showing virtual stent-graft deployment and prediction for postoperative hemodymamics is adapted from Romarowski et al. [1025] with permission from Elsevier.

Once these techniques are fully validated across a large patient cohort, image-based CFD simulations could assist physicians in identifying patients at high risk of late complications and enable personalized pre-operative planning that may help prevent post-operative complications such as stent-graft migration. A possible workflow, presented in Fig. 75, could provide clinicians with additional quantitative data and support them in diagnosis and risk stratification. However, since acute aortic dissection requires immediate intervention and decision-making, this work could be particularly applicable to cases of chronic aortic dissection where clinicians have more time for evaluation. In these cases, *in silico* models, whether during the risk assessment phase or in pre-operative planning, could prove invaluable for future personalized medicine. This is particularly important since patient management has so far been primarily driven by clinical factors and specific morphological parameters, as discussed throughout Section 3. While clinical guidelines undoubtedly add value at the population level, implementing patient-specific strategies could potentially lead to more optimal care.

As we integrate mathematical modeling and subsequent numerical simulations into clinical practice, we are confronted with a plethora of possible options, ranging from real-time 1D pipe networks to patient-specific FSI that require substantial computational resources. These decisions obviously affect the quality of our predictions. In traditional engineering science, the materials and related parameters may be known or even thoroughly tested. However, such comprehensive parameter studies are not available for the human body because the data are limited and expensive. Consequently, larger margins must be incorporated, further limiting the often simplistic assumptions of uniform thickness and stiffness of the tissue, uniform exterior support of the vessel, or parabolic or flat inflow profiles in combination with a population-mean volumetric flow rate. The development of complex models has to be accompanied by the capture of the variance of the model parameters involved and a non-invasive parameter estimation in the patient-specific scenario. A balance must be found between the richness of the model, the associated numerical effort and data availability. Therefore, the development and scalable implementation of efficient, optimized, and accurate reduced-order models, as well as the resulting accuracy and reliability of these models, have become a focus of modeling and simulation in recent years [1026]. Due to the approximation error, reduced-order models usually do not have the same qualitative properties and accuracy as full-order models. Therefore, finding a compromise between model size and approximation is an essential task. Predictive power in practical scenarios can only be truly improved if the overall accuracy is sufficiently high.

### 9.3. Closure

In summary, many key questions remain unanswered and numerous obstacles persist in developing accurate, reliable, and fast multiscale, multiphysics, and data-informed *in silico* models of aortic dissection, including the complexity of the pathological microstructure and the multiconstituent nature of blood and the challenges of numerical simulation of patient-specific models with FSI or CFD tools. Of course, there is no one-size-fits-all model, and each model must be tailored to the specific research question, with appropriate assumptions, simplifications, and the necessary verification of the same. To facilitate progress and accelerate the accumulation of knowledge, it could be beneficial to group research activities in the above directions. This approach would be advantageous from both a computational engineering and a medical or clinical perspective, as it would yield new medical data that would serve as the basis for material and computational models. Crucial to the success of future developments is the collaboration between engineers and clinicians, which is currently often lacking. As a community, we bear the responsibility of promoting knowledge transfer by incorporating current advances and novel technologies from all involved fields. This approach aims to enhance predictive accuracy in clinical settings, improve and accelerate medical device design, facilitate virtual surgeries, and conduct studies on virtual cohorts in clinical practice, ultimately advancing personalized medicine and healthcare technology.

## Figures and Tables

**Table 1:** A synopsis of the strengths and limitations of imaging modalities and their variants for clinical use and research is provided, where performance is indicated as high (+), medium (°), or low (−) (CT: Computed tomography; CTA: Computed tomography angiograhy; ECG: Electrocardiogram; PCCT: Photon-counting CT; MRI: Magnetic resonance imaging; PC: Phase-contrast; VENC: Velocity encoding; US: Ultrasound; TTE: Transthoracic ECG; TEE: Transesophageal ECG).

Parameter	Angiography	CT	CTA	ECG-gated CTA	4D CTA	PCCT	MRI	Cine MRI	MRA	2D PC-MRI	4D PC-MRI / 4D-flow MRI	(Dual) VENC 4D PC-MRI	Doppler US	TTE	TEE	Intravascular US
Portable													**✓**	**✓**	**✓**	
Invasive	**✓**														**✓**	**✓**
Radiation		**✓**	**✓**	**✓**	**✓**	**✓**										
Flow sensitive										**✓**	**✓**	**✓**	**✓**			
3D data set		**✓**	**✓**	**✓**	**✓**	**✓**					**✓**	**✓**				
Contrast-based	**✓**		**✓**	**✓**	**✓**	**✓**			**✓**							
Availability	−	+	+	+	+	°	°	°	°	−	−	−	+	+	°	°
Speed of acquisition	−	+	+	+	+	+	−	−	−	−	−	−	°	°	°	−
Spatial resolution	−	+	+	+	+	+	°	°	°	°	°	°	+	+	+	+
Temporal resolution	−	−	−	−	−	−	+	+	+	+	+	+	+	+	+	+

**Table 2:** Some of the many mutations that predispose to thoracic aortopathy, including dissection. Those at the top have definitively been shown to be disease causing, while those at the bottom have strong to moderate associations. Note the three classes of mutations – those affecting ECM, those affecting the mechanosensitive cytokine TGF-*β* and its signaling, and those affecting SMC actomyosin activity. See Pinard et al. [190] and Creamer et al. [191] for more detailed discussion (SM: smooth muscle; cGMP: cyclic guanosine monophosphate).

Discovery	Report	Gene	Affected protein	Effect
Definite association				
1988	[192–195]	*COL3A1*	Collagen III	Reduced ECM (collagen III) integrity
1991	[196]	*FBN1*	Fibrillin-1	Reduced ECM (elastic fiber) integrity
2005	[197]	*TGFBR1, TGFBR2*	TGF-*β* receptors	Altered TGF-*β* gene products
2006	[198,199]	*MYH11*	SM-myosin heavy chain	Reduced SMC actomyosin activity
2007	[200]	*ACTA2*	SM-alpha actin	Reduced SMC actomyosin activity
2010	[201]	*MYLK*	Myosin light chain kinase	Reduced SMC actomyosin activity
2011	[202]	*SMAD3*	TGF-*β* signaling molecule	Altered TGF-*β* gene products
2012	[203,204]	*TGFB2*	TGF-*β*2 ligand	Altered TGF-*β* gene products
Strong to modest association				
2011	[205]	*SMAD4*	TGF-*β* signaling molecule	Altered TGF-*β* gene products
2011	[206,207]	*FLNA*	Filamin A	Cytoskeleton/mechanotransduction
2013	[208]	*PRKG1*	cGMP-dependent kinase	Reduced SMC actomyosin activity
2015	[209]	*TGFB3*	TGF-*β*3 ligand	Altered TGF-*β* gene products
2016	[210,211]	*LOX*	Lysyl oxidase	Reduced ECM (cross-linking) integrity
2019	[212]	*SMAD4*	TGF-*β* signaling molecule	Altered TGF-*β* gene products

**Table 3:** Publication index of reports on mechanical experiments and microstructural investigations on tissues extracted from aortic dissection patients, ordered by year of publication (TL: true lumen; FL: false lumen).

Publication	Year	No. of donors	Age	Type	Objective(s)
Yamada et al. [452]	2015	Human ascending (9)	52–85	Acute type A	Histology (collagen, elastin); uniaxial tests
Babu et al. [453]	2015	Human ascending (16)	7-71	Type A	Biaxial tests
Sommer et al. [454]	2016	Human ascending (3) & descending (1)	43–73	n.a.	Shear tests (in-plane, out-of-plane)
Ahuja et al. [455]	2018	Porcine descending (1)^a^	n.a.	n.a.	Biaxial tests; layer-specific (outer wall of the TL, dissection flap, outer wall of the FL)
Manopoulos et al. [456]	2018	Human ascending (12)	35–77	Acute type A	Histology; uniaxial tests; layer-specific (outer wall of the TL, outer wall of the FL)
Deplano et al. [457]	2019	Human ascending (3)	53–57	Acute type A	Histology (collagen, elastin); biaxial tests; layer-specific (outer wall of the TL, dissection flap, outer wall of the FL)
Amabili et al. [250]	2020	Human descending (1)	73	Chronic type A	Uniaxial tests (quasi-static, dynamic); layer-specific (outer wall of the TL, dissection flap, outer wall of the FL)
Rolf-Pissarczyk et al. [450]	2021	Human ascending (1)	51	n.a.	Uniaxial tests; layer-specific (media, adventitia)
Kan et al. [458]	2021	Human (5)	n.a.	Type B	Uniaxial tests
Panpho et al. [449]	2022	Human descending & ascending (14)	37–68	Chronic	Histology (elastin); biochemical analysis (elastin, collagen, GAGs); custom-indentation test; layer-specific (outer wall of the TL, dissection flap, outer wall of the FL)
Li et al. [459]	2022	Human (35)	64–38	Type A	Uniaxial tests; layer-specific (dissection flap)
Guo et al. [460]	2024	Human (13)	41–61	Type A	Biaxial tests; histology (collagen, elastin); layer-specific (outer wall of the TL, dissection flap, outer wall of the FL)

a Artificially created aortic dissection (see Table 5)

**Table 4:** Publication index of reports on hemodynamics in aortic dissection patients assessed with medical imaging, ordered by year of publication (TL: true lumen; FL: false lumen; DSA: digital subtraction angiography; CS: compressed-sensing; PVM: phase velocity mapping).

Report	Year	Flow imaging method(s)	No. of patients	Type	Objective(s)
Mohri et al. [501]	1985	pulsed Doppler ECG	1	Type A	Hemodynamics in patient (flow pattern)
Bogren et al. [502]	1988	MRI PVM^b^	1	Type B	Hemodynamics in patient (flow rate)
Mitchell et al. [503]	1988	MRI PVM^b^	1	Type B	Hemodynamics in patient (flow rate)
Mohr-Kahaly et al. [504]	1989	2D, pulsed, color-coded Doppler ECG	18	Type A & B	Hemodynamics in patients (flow pattern)
Chang et al. [499]	1991	cine VENC MRI	6	Type B	Hemodynamics in patients (average flow velocity, flow rate)
Inoue et al. [512]	1996	PC-MRI	6	Type B	Hemodynamics in patients (flow pattern, peak average velocity)
Inoue et al. [513]	2000	cine VENC MRI	21	Type A^a^, A, & B	Hemodynamics in patients (flow volume, flow pattern)
Strotzer et al. [505]	2000	MRI PVM	14	Type A^a^ & B	Hemodynamics in patients (peak flow velocity, antegrade/retrograde flow rate)
Amano et al. [521]	2011	cine 3D MRI PVM	16	Type A & B	Hemodynamics in patients (flow pattern, flow velcity)
Müller-Eschner et al. [522]	2011	VENC 4D-flow MRI	1	Type B	Hemodynamics in patient (flow velocity)
Clough et al. [158]	2012	VENC 2D PC-MRI & VENC 4D-flow MRI	12	Type B	Comparison of hemodynamics in patients (stroke volume, flow velocity, antegrade/retrograde flow rate, helical flow) with growth rates
Markl et al. [506]	2004	VENC 4D-flow MRI	1	Type A^a^	Hemodynamics in patient (helical flow, flow pattern, flow velocity, antegrade/retrograde flow rate)
François et al. [159]	2013	VENC 4D-flow MRI	12	Type A/B	Comparison of hemodynamics in patients with/without intervention (flow pattern, peak flow rate, flow velocity, retrograde/antegrade flow rate)
Sherrah et al. [507]	2017	multiVENC 4D-flow MRI	10	Type A^a^	Hemodynamics in patients (flow rate, flow velocity, pulsatility index)
Rudenick et al. [516]	2017	VENC PC-MRI	33	Type A^a^ & B	Hemodynamics in patients (flow velocity, flow profile in FL at diaphragm level)
Liu et al. [508]	2018	VENC 4D-flow MRI	16	Type A & B	Hemodynamics in patients with diverse characteristics (flow velocity, peak flow, regurgitant fraction in TL/FL)
Guo et al. [523]	2018	VENC 4D-flow MRI	1	Type B	Hemodynamics in patient (flow velocity, flow pattern)
Allen et al. [509]	2019	VENC 4D-flow MRI	19	Type B	Hemodynamics in patients (flow velocity); detection of tear
Takei et al. [510]	2019	4D-flow MRI	1	Type B	Comparison of hemodynamics in patient with pre-/post TEVAR (flow rate, flow velocity)
Burris et al. [514]	2019	4D-flow MRI	12	Type A^a^ & B	Comparison of hemodynamics in patients (FL ejection fraction, peak flow velocity) with growth rates
Burris et al. [161]	2020	VENC 4D-flow MRI	18	Type A^a^ & B	Comparison of hemodynamics in patients (peak flow velocity, FL ejection fraction) with growth rates
Jarvis et al. [162]	2020	VENC 4D-flow MRI	20	Type A^a^ & B	Hemodynamics in patients (antegrade/retrograde flow rate, stasis, kinetic energy)
Marlevi et al. [517]	2021	VENC 4D-flow MRI	12	Type A^a^ & B	Comparison of hemodynamics in patients (FL ejection fraction, max. systolic deceleration rate, FL relative pressure) with growth rates
Chen et al. [524]	2021	VENC 4D-flow MRI	32	Type A^a^ & B	Comparison of hemodynamics in patients (regurgitant fraction in TL/FL) with/without TEVAR in descending aorta
Fang et al. [525]	2021	DSA with syngo iFlow	51	Type B	Impact of TEVAR in descending aorta on renal blood flow (pre-/post-TEVAR)
Takahashi et al. [526]	2021	VENC 4D-flow MRI	33	Type A^a^ & B	Comparison of hemodynamics in patients (gross flow, average flow velocity, regurgitant fraction in TL/FL, helical flow) with growth rates
Evangelista et al. [320]	2022	VENC 4D-flow MRI	131	Type A^a^ & B	Impact of hemodynamics in patients (flow velocity, antegrade/retrograde flow rate) on grow rates/outcome
Chu et al. [519]	2022	VENC 4D-flow MRI	51	Type A^a^ & B	Comparison of hemodynamics in patients (kinetic energy, stasis, peak flow velocity, antegrade/retrograde flow rate) with growth rates
Cosset et al. [511]	2022	VENC 4D-flow MRI	7	Type B	Comparison of hemodynamics in patients (pre-/post-TEVAR) (antegrade/retrograde flow rate, statis, helical flow)
Urmeneta Ulloa et al. [527]	2022	VENC 4D-flow MRI	3	Type A^a^, A, & B	Comparison of hemodynamics in patients (with/without endograft in descending aorta) (flow velocity)
Ruiz-Muñoz et al. [528]	2022	VENC 4D-flow MRI	54	Type A^a^ & B	Comparison of hemodynamics in patients (helical flow, pulse wave velocity, WSS) with growth rates
Liu et al. [529]	2023	4D-flow MRI	1	Type B	Hemodynamics in patient (flow velocity, flow rate)
Kilinc et al. [530]	2023	Accelerated VENC 4D-flow MRI	12	Type B	Hemodynamics in patients (kinetic energy, peak flow velocity, antegrade/retrograde flow rate, stasis); comparison between GRAPPA- and CS-accelerated 4D-flow MRI
Ruiz-Muñoz et al. [520]	2024	VENC 4D-flow MRI	68	Type A^a^ & B	Hemodynamic in patients with patent/partially thrombosed FL in descending aorta (inflow, helical flow, WSS, kinetic energy, flow acceleration, stasis)
Bellala et al. [531]	2024	VENC 4D-flow MRI	15	Type B	Comparison of hemodynamics in patients (regurgitation fraction in FL, energy loss) with growth rates

a Repaired type A with residual type B dissection;

b Based on [532]

**Table 5:** Publication index of experimental *in vivo*, *ex vivo*, and *in vitro* models related to aortic dissection, ordered by publication year (FL: false lumen).

Report	Year	No. of models	Model type	Main objective(s)
Aortic dissection replication				
Blanton et al. [571]	1959	Dog (26)	*In vivo*	Replication of aortic dissection
Trent et al. [582]	1990	Dog (12)	*In vivo*	Efficacy of endovascular implants
Moon et al. [583]	1993	Dog (12)	*In vivo*	Efficacy of endovascular implants
Kato et al. [584]	1994	Dog (14)	*In vivo*	Efficacy of endovascular implants
Kato et al. [585]	1995	Dog (10)	*In vivo*	Efficacy of endovascular implants
Marty-Ané et al. [586]	1995	Dog (14)	*In vivo*	Efficacy of endovascular implants
Razavil et al. [572]	1998	Pig (15)	*In vivo*	Efficacy of endovascular implants
Morales et al. [580]	1998	Dog (7)	*In vivo*	Effectiveness of fenestration
Zannoli et al. [589]	2007	Silicon (1)	*In vitro*	Efficacy of medical device (intravascular passive counterpulsating damper)
Cui et al. [575]	2009	Dog (12)	*In vivo*	Replication of aortic dissection
Tang et al. [576]	2010	Dog (20)	*In vivo*	Replication of aortic dissection
Fujii et al. [573]	2000	Pig (12)	*In vivo*	Replication of aortic dissection
Terai et al. [574]	2005	Dog (15)	*In vivo*	Replication of aortic dissection
Okuno et al. [577]	2012	Pig (14)	*In vivo*	Replication of aortic dissection
Faure et al. [587]	2015	Human (15)	*Ex vivo*	Efficacy of endovascular implants
El Batti et al. [581]	2018	Sheep (17)	*In vivo*	Effectiveness of fenestration
Wu et al. [578]	2021	3D printed materials (4)	*In vitro*	Replication of aortic dissection with 3D printed materials
Liu et al. [579]	2023	Dog (18)	*Ex vivo*	Efficacy of endovascular implants
Mohl et al. [588]	2024	Transparent flexible resin (1)	*In vitro*	Efficacy of endovascular implants
Dissection propagation with a single entry tear				
Halliday & Robertson [591]	1946	Human (n.a.)	*Ex vivo*	Impact of blood pressure on dissection propagation with needle-based experiments
Robertson & Viner Smith [592]	1948	Human (42)	*Ex vivo*	Impact of blood pressure on dissection propagation with needle-based experiments
Hirst & Johns [593]	1962	Human (63) & Dog (2)	*Ex vivo*	Impact of blood pressure on dissection propagation with needle-based experiments
Prokop et al. [601]	1970	Tygon – rubber cement (1) & Dog (15)	*In vitro* & *ex vivo*	Impact of pulsating blood pressure on dissection propagation
Carney et al. [602]	1975	Dog (30)	*In vivo*	Impact of rate of pressure change on dissection propagation
Roach et al. [432,595,596,600]	1987–99	Dog (36)/Pig (34)/Pig (17)/Pig (48)	*Ex vivo*	Impact of rate of pressure change on dissection propagation with needle-based experiments
Senoo et al. [603]	1989	Dog (49)	*In vivo*	Impact of intimal tear (location, size, depth) on propagation pressure
Mitsui al. [604]	1994	Dog (99)	*Ex vivo*	Impact of intimal tear (depth) on propagation pressure
Tam et al. [605]	1998	Pig (20)	*In vitro*	Impact of intimal tear (location, depth) on propagation pressure
Peelukhana et al. [606]	2017	Pig (36)	*Ex vivo*	Impact of intimal tear (location, size, depth) on hemodynamics/flap motion
Guo et al. [607]	2019	Pig (12)	*In vivo*	Impact of intimal tear (location, depth) on hemodynamics/dissection propagation
Brunet et al. [609]	2023	Rabbit (11)	*Ex vivo*	Visualization of intimal tear/propagation/flap configuration with CT imaging/histology; impact of blood pressure on propagation
Wang et al. [639]	2023	Pig (70)	*Ex vivo*	Impact of intimal tear (location, size, depth) on propagation pressure
Effects of intimal tears on hemodynamics and dissection flap				
Iwai et al. [614]	1991	Styrene–butadiene–styrene rubber (3)	*In vitro*	Impact of intimal tear (location) on hemodynamics/flap motion
Chung et al. [615,616]	2000	Bicycle tire inner tube – PTFE graft (1) & PTFE (1)	*In vitro*	Impact of intimal tear (location, size) on hemodynamics/flap motion
Tsai et al. [610]	2008	Polydimethylsiloxane (1)	*In vitro*	Impact of intimal tears (location, size) on hemodynamics
Dziodzio et al. [267]	2011	Pig (26)	*Ex vivo*	Impact of intimal tears (location) on dissection propagation
Qing et al. [617]	2012	Pig (15)	*Ex vivo*	Impact of intimal tear (location) on hemodynamics/flap motion
Rudenick et al. [618]	2013	Latex (8)	*In vitro*	Impact of intimal tear (location, size) on hemodynamics
Faure et al. [619]	2014	Human (20)	*Ex vivo*	Impact of intimal tear (location) on dissection propagation
Veger et al. [620]	2015	Pig (2)	*Ex vivo*	Impact of branch arteries on false lumen dilatation
Birjiniuk et al. [624–626,640]	2015–20	Silicone (1)/Silicone (2)/Silicone (3)/Silicone (4)	*In vitro*	Impact of intimal tear (location) on hemodynamics/flap motion
Veger et al. [621]	2017	Pig (6)	*Ex vivo*	Impact of tear (location) on hemodynamics
Marconi et al. [627]	2017	Transparent rigid resin/silicone elastomer (10)	*In vitro*	Impact of intimal tear (location, size) on hemodynamics
Ahuja et al. [455,613]	2018	Pig (5) & (5)	*Ex vivo*	Impact of balloon catheter in parallel channel on radial pressure; calibration to layer-specific material behavior
Canchi et al. [628]	2018	Pig (10)	*Ex vivo*	Impact of intimal tear (location, size) on hemodynamics/flap motion
Moore et al. [590]	2019	PLA (2)	*In vitro*	Development of biomaterials for vascular embolic treatment
Salameh et al. [629]	2019	Plexiglass (8)	*In vitro*	Impact of intimal tear (location, size) on hemodynamics
De Beaufort et al. [160]	2019	Pig (13) & Human (14)	*Ex vivo*	Comparison of hemodynamics with *In vivo* data (4D-flow MRI, 2D PC-MRI)
Zadrazil et al. [630]	2020	PFA (4)	*In vitro*	Impact of intimal tear (location, size) on hemodynamics
Bonfanti et al. [635–637]	2020–23	TuskXC2700T (1)	*In vitro*	Impact of intimal tear on hemodynamics
Veger et al. [622]	2020	Pig (3)	*Ex vivo*	Impact of outer wall elasticity on FL dilatation
Veger et al. [623]	2021	Pig (2)	*Ex vivo*	Impact of heart rate on hemodynamics; validation to *in vivo* data (4D-flow MRI)
Chi et al. [608]	2022	Silicone (2)	*In vitro*	Impact of intimal tear (size)/layer adhesion on dissection propagation
Liang et al. [611]	2022	Pig (24)	*Ex vivo*	Impact of intimal tear (location) on hemodynamics/flap motion
Chen et al. [631]	2022	Silicone (2)	*In vitro*	Impact of heart rate/flow rate on hemodynamics with healthy and dissection models
Zimmermann et al. [612]	2023	Photopolymer resin coating film (3)	*In vitro*	Impact of intimal tear (size) on hemodynamics
Aghilinejad et al. [638]	2023	Polyvinyl alcohol coated with latex (4)	*In vitro*	Development of fully automated patient-specific aortic dissection model fabrication

**Table 6:** Publication index of multiscale material models on damage and failure related to aortic dissection, ordered by year of publication (FL: false lumen).

Report	Year	Method(s): Numerical	Damage and failure	Solver	Main objective(s)
		
Delamination of the wall					
Gasser & Holzapfel [648,650]	2005/06	PUFEM	–	FEAP	Modeling balloon angioplasty in aortic dissection; validation to peeling tests
Rajagopal et al. [11]	2007	FEM	Constrained mixture model	–	–
Ferrara & Pandolfi [653,654]	2008/10	FEM	Cohesive zone model	Custom code	Validation to peeling tests
Pal et al. [656]	2014	FEM	Cohesive zone model	Custom code	Validation to peeling tests; impact of radially-directed collagen fibers on delamination
Shah et al. [659]	2014	FEM	Individual fiber failure	Custom code	Validation to uniaxial and biaxial extension
Leng et al. [661]	2015	FEM	Cohesive zone model	Abaqus^a^	Validation to atherosclerotic plaque delamination tests
Witzenburg et al. [660]	2016	FEM	Individual fiber failure	Custom code	Validation to uniaxial extension, biaxial extension, peeling, and lab shear tests
Thunes et al. [663,665]	2016/18	FEM	Individual fiber failure	Custom code	Modeling medial lamellar unit with microstructural-based model
Noble et al. [666]	2017	FEM	Cohesive zone model	MARC	Validation of peeling tests to model catheter-induced dissection
Leng et al. [662]	2018	FEM	Cohesive zone model	Abaqus^a^	Validation of atherosclerotic plaque delamination tests
Brunet et al. [461]	2019	FEM	Cohesive zone model	Abaqus & Custom code	Validation to tensile rupture tests with medial strip
Yu et al. [671]	2020	FEM	Individual fiber failure	Abaqus^a^	Validation of peeling tests; role of radially-directed collagen fibers in avalanche-like delamination behavior
Wang et al. [674]	2020	FEM	Individual fiber failure	Abaqus^a^	Validation of peeling test; role of radially-directed elastic/collagen fibers in oscillating delamination
Dissection propagation and flap configuration					
Wang et al. [675]	2015	FEM	Cohesive zone model	FEAP	Impact of fiber-reinforced tissue on tear propagation with 2D medial strip
Gültekin et al. [681–683]	2016–19	FEM	Phase-field approach	FEAP	Impact of pressure on tear propagation in symmetric 3D tube
Wang et al. [677,679]	2017/18	XFEM	–	Abaqus	Impact of residual stress/tear length/pressure/fiber alignment on flap configuration/propagation direction in symmetric 2D tube
Ahuja et al. [613]	2018	FEM	–	Abaqus	Validation of flap configuration to *in vitro* experiments
Mousavi et al. [684]	2018	FEM	Continuum damage theory & constrained mixture theory	Abaqus^a^	Impact of pressure on location of initiation/direction of dissection propagation in aortic aneurysms
Li et al. [704]	2019	FEM	Incremental deformation model	FEAP	Impact of residual stress/axial stretch/fiber alignment/pressure on FL volume with 2D medial layer
Rolf-Pissarczyk et al. [450,685]	2021	FEM	Exclusion of degraded fibers	FEAP & Abaqus^a^	Modeling degradation of radially-directed elastic fibers in dissection model
Brunet et al. [691]	2021	XFEM	–	Abaqus^a^	Impact of tear geometry/pressure/axial stretch/material parameters on tear propagation in symmetric 3D tube
Ban et al. [693,696,697]	2021/22	FEM	Phase-field approach	FEniCS	Modeling needle experiments to uncover microstructural-based tear propensities in descending/abdominal aorta
FitzGibbon et al. [701,703]	2021/22	FEM	Cohesive zone model	Abaqus	Impact of pressure on tear propagation with symmetric 3D tube; development of novel cohesive zone model
Zhang et al. [705]	2022	FEM	–	Abaqus^a^	Impact of residual stress on flap configuration with 3D dissection segment
Han et al. [708]	2023	FEM	Cohesive zone model	Abaqus^a^	Impact of tear geometry/residual stress/luminal pressure difference on tear propagation in symmetric 3D tube
Yeerella et al. [741]	2024	FEM	Fracture mechanics theory (energy release rate)	Abaqus^a^ & Semi-analytic	Impact of material parameters/tear depth/ wall thickness/pressure on propagation in symmetric 2D tube
Gheysen et al. [709]	2024	FEM	–	Abaqus^a^	Impact of material parameters/flap thickness on stress/deformation
Focal areas of medial degeneration					
Roccabianca et al. [79,700]	2014	FEM	–	FEBio	Impact of pooled GAGs in media on wall stress/stress concentrations with 3D medial strip
Ahmadzadeh et al. [719,722]	2018/19	SPH	Continuum damage theory	FEBio & Custom code	Impact of microstructural changes in media on wall stress; modeling growth and coalescence of pooled GAGs
Liu et al. [724]	2022	FEM	Material volume fraction	Abaqus^a^	Impact of swelling pressure of pooled GAGs in media on stress in elastic fiber struts
Ranftl et al. [717]	2022	FEM	Exclusion of degraded fibers	FEAP	Stochastic modeling of degradation of radially-directed elastic fibers with 3D medial strip
Soleimani et al. [728]	2023	FEM	Phase-field approach	AceGen & Ansys	Modeling tearing (rupture) in the artery wall following microinjuries in vasa vasorum
Growth and remodeling					
Zhang et al. [734]	2022	FEM	Homogenized constrained mixture theory	Open-Source^b^	Modeling FL dilatation with growth and remodeling framework
Gheysen et al. [714]	2024	FEM	Homogenized constrained mixture theory	Abaqus^a^	Comparison between dilatation/layer thickness growth rates obtained with different inflammatory patterns/clinical observations

a Standard;

b [742,743]

**Table 7:** Publication index of *in silico* hemodynamic models related to aortic dissection, ordered by publication year (FL: false lumen; Lam.: laminar; Turb.: turbulent).

Report	Year	Solver	Flow assumption	No. of patients	Type	Main objective(s)
Simplified studies						
Guan et al. [750]	2009	Ansys	Lam.	–	Type B	Comparison of hemodynamics in simplified models with/without bypassing treatment (from left subclavian artery to abdominal aorta)
Fan et al. [749]	2010	Ansys^a^	Lam.	–	–	Impact of intimal tear (location, size)/luminal area ratio on hemodynamics in simplified models
Chitsaz et al. [751]	2012	OpenFOAM	Lam.	–	–	Impact of intimal tears (number) on tearing force in longitudinal direction/(d*p*/d*t*) in symmetric 2D tubes
Tang et al. [752]	2012	Ansys^a^	Turb.	–	Type B	Impact of intimal tear (number, location)/FL diameter on force acting on the wall in simplified dissection models
Rudenick et al. [516,747,748]	2015/17	Matlab	n.a.	–	–	Impact of wall stiffness/intimal tear (size, location) on hemodynamics with lumped-parameter model; validation to *in vitro* experiments
Ben Ahmed et al. [753]	2016	CRIMSON	Lam.	–	–	Impact of intimal tear (number, size, location)/vessel curvature on hemodynamics in simplified models
Zadrazil et al. [630]	2020	Ansys^a^	Turb.	–	Type B	Validation of hemodynamics to *in vitro* experiments
Zorilla et al. [754]	2020	Kratos^d^	n.a.	–	Type B	Validation of hemodynamics to *in vitro* experiments
Li et al. [757,758]	2021	Ansys^a^	Lam.	–	Type B	Morphological parameters
Qiao et al. [756]	2022	Ansys	Lam.	–	Type B	Impact of stent-graft introducer sheath during TEVAR on hemodynamics in simplified aneurysm/coarctation/dissection models
Peng et al. [755]	2022	Ansys^a^	Lam.	–	–	Impact of morphological parameters on hemodynamics in simplified healthy model
Cross-sectional case studies						
Karmonik et al. [812]	2008	Ansys^a^	n.a.	3	Multiple	Comparison of hemodynamics in patients with mobile thrombus in aortic arch/acute type II/abdominal aortic aneurysm repair
Cheng et al. [759]	2010	Ansys^b^	Turb.	1	Type B	Hemodynamic simulations with hybrid SST k-∈/k-*ω* turbulence model
Chen et al. [767]	2013	CFD-ACE+	Turb.	1	Type B	Hemodynamic simulations with SST k-*ω* turbulence model
Cheng et al. [760]	2014	Ansys^b^	Turb.	1	Type B	Validation of hemodynamics to *in vivo* data
D’Ancona et al. [813]	2014	n.a.	n.a.	4	Type B	Hemodynamics in patients
Djorovic et al. [762]	2015	PAK-F	Lam.	2	Type A	Hemodynamics in patients
Cheng et al. [761]	2015	Ansys^b^	Turb.	8	Type B	Comparison of hemodynamics/morphology in patients with medical treatment/TEVAR
Wan Ab Naim et al. [814]	2016	Ansys^a^	Lam.	1	Type B	Impact of intimal tear (number) on hemodynamics in patients
Osswald et al. [815]	2017	Star-CCM+	Lam.	20	Type B	Comparison of hemodynamics in patients with/without subsequent retrograde type A dissection
Long Ko et al. [816]	2017	Ansys^b^	Turb.	3	Type B	Hemodynamic simulations with SST k-∈ turbulence model
Iida et al. [817]	2017	n.a.	n.a.	1	Type B	Hemodynamics in patient with ulcer-like projection
Chi et al. [818]	2017	Ansys^a^	Turb.	7	Type A	Hemodynamics in patients (pre-dissection)
Bonfanti et al. [770]	2019	Ansys^b^	Lam.	1	Type B	Development of algorithm for calibration of flow boundary conditions
Peng et al. [819]	2019	Ansys^a^	n.a.	1	–	Hemodynamics in patient with interrupted aortic arch (implications for dissection)
Bonfanti et al. [635]	2020	Ansys^b^	Turb.	1	Type B	Validation to *in vitro* experiments/*in vivo* data
Tomasi et al. [820]	2020	Ansys	Turb.	2	Type B	Comparison between CFD and reduced-order models in healthy/dissection patients
Li et al. [821]	2020	Ansys^a^	n.a.	120	Type A	Comparison of hemodynamics in patients with different proximal-to-distal tear size ratios
Qiao et al. [822]	2020	Ansys	Lam.	2	Type B	Hemodynamics in patients with TEVAR/*in situ* double fenestrations of left carotid and subclavian artery post-operation
Marrocco-Trischitta et al. [823]	2021	LifeV	n.a.	5	–	Hemodynamics in patients with type I/II/II arch (implications for dissection)
Li et al. [824]	2021	CFD-ACE+	Lam.	2	Type B	Impact of inlet velocity profiles on validation to *in vivo* data in healthy/dissection patients
Hohri et al. [825]	2021	Ansys	Turb.	6	–	Comparison of hemodynamics in healthy/pre-dissection patients
Abazari et al. [764]	2021	Ansys^b^	Lam.	1	Type B	Impact of beta-blocker medication on hemodynamics
Armour et al. [805]	2021	Ansys^b^	Lam.	2	Type B	Impact of inlet velocity profile on hemodynamics on validation to *in vivo* data
Armour et al. [773]	2022	Ansys^b^	Lam.	5	Type B	Validation of hemodynamics to *in vivo* data
Wen et al. [826]	2022	Ansys^a^	n.a.	30	Healthy	Impact of arch type I/II/III on hemodynamics (implications for dissection)
Kimura et al. [775]	2022	scFLOW	n.a.	19	Type B & non-A non-B	Comparison of hemodynamics/morphology in type B/non-A non-B patients
Takeda et al. [827]	2022	OpenFOAM	Turb.	11	Type A & B	Comparison of hemodynamic simulations with SST k-∈ turbulence model in healthy/type A/type B patients
Wang et al. [772]	2022	OpenFOAM	Lam.	1	Type A	Validation of hemodynamics to *in vivo* data post-operation with residual dissection
Moretti et al. [776]	2023	OpenFOAM	Turb.	4	Multiple	Comparison of hemodynamics in healthy patient and patients with patent/partially thrombosed/completely thrombosed FL
Stokes et al. [777]	2023	Ansys^b^	Turb.	1	Type B	Impact of different *in vivo* data for inlet conditions on hemodynamics in aneurysmal dissection
Black et al. [779]	2023	Ansys^a^	Turb.	1	Type B	Development of reduced-order framework to calibrate Windkessel model parameters with *in vivo* data in healthy/dissection patients
Chatpattanasiri et al. [637]	2023	Ansys^b^	Turb.	1	Type B	Validation of hemodynamics in reduced-order model to *in vitro* data
Tsai et al. [828]	2024	n.a.	n.a.	1	Type A	Hemodynamics in patient with iatrogenic aortic dissection
Messou et al. [829]	2024	SimVascular	n.a.	3	Type B	Comparison of hemodynamics in patients with FL thrombosis, a large fenestration, and the orbital orientation of the FL
Wang et al. [830]	2024	Ansys^b^	Lam.	5	Type B	Impact of different *in vivo* data (only ECG, ECG with stroke volume, 4D-flow MRI) for inlet conditions on hemodynamics
Zorilla & Soudah [831]	2024	Kratos^d^	n.a.	4	Type B	Development of two-step segmentation procedure for thin-walled flap; hemodynamics in patients
Wang et al. [832]	2024	Ansys^b^	n.a.	5	Type B	Development of novel framework to tune inlet boundary conditions to *in vivo* data in dissection patients (post-TEVAR)
Virtual case studies						
Karmonik et al. [780]	2011	Ansys^a^	n.a.	1	Type B	Impact of virtually occluded tears/removed flap on hemodynamics
Chen et al. [781]	2013	CFD-ACE+	Lam.	1	Type B	Comparison of hemodynamics in patient by virtually occluding different tears (pre-/post-medical treatment)
Alimohammadi et al. [765]	2014	Ansys^b^	Lam.	1	Type B	Impact of virtually occluded tears on hemodynamics in patients with coarctation
Alimohammadi et al. [766]	2014	Ansys^b^	Lam.	1	Type B	Impact of virtually occluded tears on hemodynamics in patients
Wan Ab Naim et al. [782]	2014	Ansys^a^	Lam.	1	Type B	Impact of virtually added tears on hemodynamics
Dillon-Murphy et al. [783]	2016	CRIMSON	n.a.	1	Type B	Comparison of virtually added tears/occluded tears/removed flap on hemodynamics
Shi et al. [784]	2016	Ansys^a^	Lam.	4	Type A	Impact of virtually altered tears (size, location, number) on hemodynamics
Yu et al. [785]	2016	Ansys^a^	n.a.	4	Type B	Impact of virtually altered tears (size, number) on hemodynamics
Rikhtegar Nezami et al. [833]	2018	Ansys^b^	n.a.	1	Type B	Impact of multilayer flow modulator on end-organ perfusion (pre-/post-operation and virtual pre-dissection)
Jiang et al. [834]	2019	Ansys^a^	Lam.	1	Type B	Comparison of hemodynamics by virtually occluding different branch arteries (post-TEVAR)
Qiao et al. [835]	2019	Ansys	Lam.	1	Type B	Comparison of hemodynamics between occluded (pre-TEVAR)/virtually added left subclavian artery (post-TEVAR)
Dai et al. [786]	2020	Ansys^2^	Turb.	1	Type B	Comparison of hemodynamics in patient with multiple overlapping uncovered stents by virtually occluded/partially occluded tears
Qiu et al. [836]	2020	Ansys^a^	Lam.	1	Type B	Comparison of hemodynamics post-TEVAR with virtual pre-TEVAR/virtually removing graft material
Xiong et al. [787]	2020	Ansys^a^	n.a.	1	Non-A non-B	Comparison of hemodynamics pre-/post-hybrid arch repair, and virtually removing flap
Polanczyk et al. [837]	2021	Ansys^a^	Lam.	12	Type B	Comparison of hemodynamics/hematocrit value pre-/post-TEVAR, and by virtually removed flap
Sengupta et al. [838]	2022	Ansys^b^	Turb.	2	Type A	Comparison of hemodynamics post-TEVAR (double-branched endograft in aortic arch) by virtually altering tunnel branch diameters
Li et al. [788]	2022	Ansys^b^	n.a.	5	Type B	Comparison of hemodynamics in pre-TEVAR/post-TEVAR/post-TEVAR with virtually occluded tear patients
Li et al. [839]	2022	Ansys^a^	n.a.	2	Type B	Comparison of hemodynamics in different bypass strategies (left subclavian artery) of healthy patients and patients with/without dissection
Stokes et al. [840]	2023	Ansys^b^	Turb.	1	Type B	Validation of hemodynamics with minor/major branch arteries and virtually occluded minor branch vessels to *in vivo* data
Li et al. [789]	2023	Ansys^a^	Lam.	27	Type B	Comparison of hemodynamics between in healthy/dissection patients and virtually removed flap
Motoki et al. [841]	2023	Ansys^b^	Turb.	2	Type A	Comparison of hemodynamics by virtually altered tears (size, location,number) (post-operation)
Wen et al. [790]	2023	Ansys^a^	n.a.	16	Type B	Comparison of hemodynamics/morphology in healthy/dissection/virtual pre-dissection patients
Zhu et al. [842]	2023	Ansys^b^	Turb.	1	Type A	Comparison of hemodynamics in patients with frozen elephant trunk procedure (virtual) (pre-/post-operation); validation to *in vivo* data
Chen et al. [843]	2023	n.a.	n.a.	1	Type B	Comparison of hemodynamics in double/single (virtual) FL abdominal aortic dissection aneurysm, and saccular abdominal aortic aneurysm
Liu et al. [844]	2024	Ansys^a^	n.a.	1	Type B	Comparison of hemodynamics by virtually altering tears (number) (post-dissection) and virtual pre-dissection
Girardi et al. [845]	2024	Ansys^a^	Turb.	1	Type B	Comparison of hemodynamics in three different grafting strategies (pre-/post-operation)
Longitudinal case studies						
Tse et al. [846]	2011	ADINA	Lam.	1	Type B	Hemodynamics in patients with aortic valve/proximal ascending aortic replacement (pre-/post-operation)
Karmonik et al. [797]	2011	Ansys^a^	n.a.	1	Type B	Hemodynamics in patients (pre-/post-TEVAR)
Karmonik et al. [446,798]	2012/13	Ansys^a^	n.a.	1	Type B	Comparison of hemodynamics in healthy patients and in patients with FL dilatation
Cheng et al. [799]	2013	Ansys^b^	Turb.	5	Type B	Comparison of hemodynamics/tear/FL diameter in uncomplicated/complicated cases
Mori et al. [847]	2013	Ansys^a^	Lam.	2	Type B	Hemodynamics in patient with ulcer-like projections in completely thrombosed FL
Rinaudo et al. [848]	2014	Ansys^a^	Lam.	25	Type B	Impact of tear/luminal area/hemodynamics in patients on outcome
Shang et al. [763]	2015	Abaqus/CFD	n.a.	14	Type B	Comparison of hemodynamics/growth rates in patients with stable/rapidly growing FL diameters
Stefanov et al. [849]	2017	Ansys^b^	Lam.	12	Type B	Comparison of morphology in follow-up and impact of streamliner multilayer flow modulator on hemodynamics
Xu et al. [774]	2017	CFD-ACE+	Turb.	2	Type B	Comparison of hemodynamics/tear in patients with stable FL/FL dilatation (post-TEVAR)
Xu et al. [850]	2018	LifeV	Lam.	1	Type B	Comparison of hemodynamics/morphology in patients
Wan Ab Naim et al. [851]	2018	Ansys^a^	Turb.	5	Type B	Comparison of hemodynamics/morphology in patients with completely/partially thrombosed FL (post-TEVAR)
Costache et al. [800]	2018	Ansys	n.a	1	Type B	Comparison of hemodynamics in patients with multilayer flow modulator (pre-/post-operation)
Pirola et al. [803]	2019	Ansys^b^	n.a.	1	Type B	Validation of hemodynamics to *in vivo* data (pre-/post-TEVAR)
Athanasiou et al. [852]	2019	Ansys^b^	n.a.	2	Type B	Comparison of hemodynamics in patients with multilayer flow modulator (pre-/post-operation)
Costache et al. [801]	2020	Ansys^a^	n.a.	16	Type B	Impact of multilayer flow modulator on aortic remodeling/hemodynamics (pre-/post-operation)
Xu et al. [804]	2021	CFD-ACE+	n.a.	51	Type B	Comparison of hemodynamics/morphology in patients (pre-/post-TEVAR)
Armour et al. [805]	2021	Ansys^b^	Lam.	2	Type B	Impact of inflow profile on hemodynamics (pre-/post-TEVAR); validation to *in vivo* data
Zhu et al. [806]	2021	Ansys^b^	Turb.	17	Type A	Impact of hemodynamics/morphology on FL dilatation in patients (post-operation)
Costache et al. [802]	2021	Ansys	n.a.	23	Type B	Comparison of hemodynamics/morphology in patient with multilayer flow modulator (pre-/post-operation)
Wang et al. [853]	2021	Ansys^a^	Lam.	15	Type A	Impact of ascending aortic diameter/hemodynamics in dissection/healthy patients on outcome
Shad et al. [854]	2022	SimVascular	n.a.	9	Type A	Impact of hemodynamics/morphology on FL dilatation in patient (post-operation)
He et al. [855]	2022	Ansys^a^	n.a.	53	Type B	Impact of hemodynamics in patients pre-/post-residual dissection and in patients with closed FL (post-TEVAR)
Liu et al. [856]	2022	Ansys^a^	Lam.	2	Type B	Impact of inlet/outlet boundary conditions on hemodynamics pre-/post-TEVAR with/without involvement of the abdominal aorta; validation to *in vivo* data
Zhu et al. [807]	2022	Ansys^b^	Turb.	4	Type A	Impact of hemodynamics/morphology on FL dilatation in patients (post-operation)
Fatma et al. [810]	2022	Ansys^a^	Lam.	2	Type A	Comparison of hemodynamics in patient with/without FL dilatation (post-operation)
Parker et al. [808]	2022	n.a.	n.a.	69	Type B	Impact of FL thrombosis on FL pressure/complications
Armour et al. [809]	2022	Ansys^b^	Lam.	1 (Pig)	Type B	Impact of tears (number) on hemodynamics/FL dilatation
Zhang et al. [857]	2022	CFD-ACE+	n.a.	1	Type B	Comparison of hemodynamics in patient with modular single-branched stent-graft with aberrant right subclavian artery (pre-/post-operation)
Wang et al. [858]	2023	n.a.	n.a.	1	Type B	Hemodynamics in patient with residual dissection (pre-/post-TEVAR)
Osswald et al. [811]	2023	Star CCM+	Lam.	5	Type B	Role of hemodynamics in patient with frozen elephant trunk procedure in dSINE (pre-/post-operation)
Yoon et al. [859]	2023	n.a.	n.a.	16	Type B	Impact of specific stent-graft on dSINE (post-TEVAR)
Pan et al. [860]	2023	FOAMExtend	n.a.	1	Type B	Validation of hemodynamics to *in vivo* data (pre-/post-TEVAR)
Bäumler et al. [861]	2023	SimVascular	n.a.	4 (Mouse)	Type B	Impact of FL thrombus formation on hemodynamics in mice models with angiotensin II-induced dissection
Wang et al. [862]	2023	Ansys^b^	n.a.	4	Type B	Comparison of hemodynamics with/without dSINE (post-TEVAR)
Jiang et al. [863]	2023	Ansys^a^	Lam.	6	Type A	Impact of physician-modified endograft in patient with complicated dissection on outcome (pre-/post-operation)
Ritter et al. [864]	2024	Star CCM+	Lam.	5	Type A, B, & non-A non-B	Impact of stent-assisted balloon-induced intimal disruption and relamination technique on hemodynamics (pre-/post-operation)
Kimura et al. [865]	2024	n.a.	n.a.	1	Type B	Comparison of hemodynamics in patient with mesenteric artery malperfusion due to dynamic obstruction (pre-/post-TEVAR)
Forneris et al. [866]	2024	Ansys^a^	Lam.	22	Type B	Comparison of hemodynamics/aortic dilatation in complicated/uncomplicated cases
Mei et al. [867]	2024	Ansys^b^	n.a.	55	–	Impact of hemodynamics/morphology on FL dilatation in patient with superior mesenteric artery dissection

a Fluent;

b CFX;

c Mechanics;

d Multiphysics;

e Explicit;

f Standard

**Table 8:** Publication index of *in silico* models of FSI related to aortic dissection, ordered by publication year (TL: true lumen; Lam.: laminar; Turb.: turbulent; Iso.: isotropic; Aniso.: anisotropic).

Report	Year	Method(s)	Solver	Flow assumption	Wall model	No. of patients	Type	Main objective(s)
Simplified studies								
Qiao et al. [872]	2015	FSI	Ansys	Lam.	Iso.	–	Type B	Comparison between two bypass strategies (from ascending to abdominal aorta) in simplified models
Chen et al. [873]	2016	FSI	Abaqus	Turb.	Iso.	–	–	Validation of simplified model (tubular segment with flap) to *in vitro* data
Jayendiran et al. [874]	2018	FSI	Abaqus	Turb.	Aniso.	–	–	Comparison between different material models in simplified models (tubular segment with Dacron graft)
Ryzhakov et al. [875]	2019	FSI	Kratos^d^	Lam.	Iso.	–	–	Validation of simplified dissection model to *in vitro* data
Keramati et al. [876–878]	2020–24	FSI	COMSOL	Lam.	Iso.	–	–	Impact of entry/exit tears in simplified model (tubular segment with flap); comparison between FSI and reduced-order model; sensitivity analysis
Chong et al. [884]	2020	FSI	COMSOL^c^	Lam.	Iso.	–	Type B	Impact of flap motion on hemodynamics in simplified models; comparison between CFD and FSI models
Saveljica & Filipovic [879]	2021	FSI	PAK-SF	Lam.	Iso.	–	Type A & B	Comparison of hemodynamics/wall stress in simplified healthy/dissection models
Aghilinejad et al. [881]	2022	IB-LBM	n.a.	Lam.	Iso.	–	–	Impact of stent-graft length on hemodynamics in simplified models (tubular segment with flap/tears)
Qiao et al. [880,903]	2022/23	FSI	Ansys	Turb.	Iso.	–	Type B	Comparison of hemodynamics/heat transfer in simplified healthy/aneurysmatic/coarctation/dissection models
Lee et al. [883]	2023	FSI	Ansys^c^	Turb.	Iso.	–	Type B	Impact of different cannulation methods in simplified models
Kim et al. [882]	2023	FSI	n.a.	n.a.	n.a.	–	Type B	Impact of hemodynamics/mechanics on dynamic obstruction of TL with simplified models
Patient-specific studies								
Alimohammadi et al. [868]	2015	FSI	Ansys	Turb.	Iso.	1	Type B	Comparison between CFD and FSI models; validation of flap motion to *in vivo* data (literature)
Malvindi et al. [904]	2016	FSI	Abaqus^f^ & Ansys^a^	n.a.	n.a.	1	–	Modeling hemodynamics/wall stress pre-dissection with ascending aortic aneurysm
Bonfanti et al. [768]	2017	MBM	Ansys^b^	Lam.	Iso.	1	Type B	Validation of hemodynamics to *in vivo* data
Bonfanti et al. [769]	2018	FSI & MBM	Ansys^b^	Turb.	Iso.	1	Type B	Comparison between MBM, FSI, and CFD models
Qiao et al. [869]	2019	FSI	Ansys	Lam.	Iso.	1	Type B	Comparison between FSI and CFD models with two-phase non-Newtonian blood
Khannous et al. [888]	2020	FSI	Ansys	Lam.	Iso.	1	Type B	Modeling residual aortic dissection
Bäumler et al. [448]	2020	FSI	SimVascular	Lam.	Iso.	1	Type B	Impact of flap motion/stiffness on hemodynamics; validation of hemodynamics to *in vivo* data
Deng et al. [905]	2021	FSI	ADINA	Lam.	Iso.	1	Type A	Impact of different cannulation methods on hemodynamics/wall stress
Zhu et al. [900]	2022	FSI	Ansys^b,c^	Turb.	Iso.	2	Type A	Impact of wall stiffness on wall stress post-graft replacement; comparison between CFD and FSI models
Qiao et al. [906]	2022	FSI	Ansys	Turb.	Iso	6	Type B	Hemodynamics/wall stress in patients with distal dSINE (post-TEVAR follow-ups)
Schussnig [894]	2023	FSI	Abaqus^f^	Lam.	Aniso.	1	Type B	Development of local fiber orientation algorithm for layer-specific dissection models
Wang et al. [902]	2023	FSI	Ansys	Turb.	Aniso.	1	Type A	Hemodynamics in dissection patient
Zimmermann et al. [612]	2023	FSI	SimVascular	Lam.	Iso.	1	Type B	Validation of hemodynamics to *in vitro* data; impact of tear size on hemodynamics/flow jet
Luan et al. [907]	2023	FSI	Ansys	Lam.	Iso.	23	Type B	Comparison of morphology/wall stress in patients with dSINE (pre-dSINE and pre-/post-operation follow-ups)
Deplano et al. [908]	2024	FSI	Ansys^b,c^	Lam.	Iso.	1	Type A	Comparison between FSI and CFD (with/without rigid flap) models in patient with residual dissection (post-operation follow-up)
Girardin et al. [909]	2024	MBM	Ansys^b^	Lam.	Iso.	1	Type B	Impact of wall stiffness/graft length/graft stiffness on hemodynamics post-operation
Schussnig et al. [899]	2024	FSI	Deal.II	Turb.	Aniso.	1	Type B	Impact of material models/non-Newtonian flow on hemodynamics/wall stress
Bäumler et al. [342]	2024	FSI	SimVascular	Lam.	Aniso.	1	Type B	Hemodynamics/wall stress in patient (pre-/post-dissection follow-ups); impact of flow jet on wall remodeling

a Fluent;

b CFX;

c Mechanics;

d Multiphysics;

e Explicit;

f Standard

**Table 9:** Publication index of patient-specific *in silico* models on selected topics relating to aortic dissection, more precisely CSM models on wall mechanics, deployment and migration of stent-grafts, and thrombus formation, ordered by year of publication (Lam.: laminar; Iso.: isotropic; Aniso.: anisotropic).

Report	Year	Method(s)	Solver	Flow assumption	Wall model	No. of patients	Type	Main objective(s)
Patient-specific studies on wall mechanics								
Beller et al. [910]	2004	CSM	Ansys^c^	–	Iso.	–	–	Impact of aortic root motion on wall stress in simplified healthy aorta (implications for dissection)
Doyle et al. [911]	2013	CSM	Abaqus^f^	–	Iso.	50	–	Impact of aortic geometry on wall stress in healthy aorta (implications for dissection)
Plonek et al. [913]	2017	CSM	Ansys^c^	–	Iso.	–	–	Impact of blood pressure/axial stretch on wall stress in simplified aortic aneurysm (implications for dissection)
Plonek et al. [912]	2018	CSM	Ansys^c^	–	Iso.	4	Type A	Comparison between wall stress pre-dissection and intimal tear (location) post-dissection
Menichini et al. [914]	2018	CSM	Ansys^c^	–	Iso.	2	Type B	Comparison between wall stress post-TEVAR and formation of dSINEs
Houben et al. [338]	2020	CSM	CRIMSON	–	Iso.	3	–	Comparison between wall stress pre-dissection and focal area of growth
Subramaniam et al. [915]	2021	CSM	FEBio	–	Aniso.	2	Type A	Impact of aortic root motion/material models on wall stress
Hu et al. [916]	2021	CSM	Abaqus^f^	–	Iso.	30	Type B	Comparison between morphological parameters and wall stress
Stent-graft deployment and migration								
Ma et al. [920]	2018	CSM	Abaqus^e^	–	Iso.	1	Type B	Impact of stent-graft with/without connecting bar on wall stress
Zhou et al. [930]	2019	CSM	Abaqus	–	–	–	–	Development of stent-graft for TEVAR; validation to *in vitro* experiments
Meng et al. [921]	2020	CSM	Abaqus^e^	–	Iso.	1	Type B	Impact of stent-graft oversizing ratio on wall stress
Yuan et al. [922]	2020	CSM	Abaqus^e^	–	Iso.	1	Type A	Impact of stent-graft landing zone on wall stress/stent-graft migration
Kan et al. [924]	2021	CSM	Abaqus^e^	–	Iso.	1	Type B	Validation of stent-graft deployment and migration framework to *in vivo* data (post-TEVAR follow-ups)
Kan et al. [458]	2021	CSM	Abaqus^e^	–	Iso.	1	Type B	Impact of stent-graft length on wall stress
Kan et al. [925]	2024	CSM	Abaqus^e^	–	Iso.	1	Type B	Impact of stent-graft length/model on wall stress/stent-graft migration
Li et al. [951]	2024	CSM	Abaqus^e^	–	Iso.	2	Type B	Comparison between wall stress in patients with/without distal stent-graft induced new tears
Thrombus formation								
Menichini & Xu [932]	2016	CFD	Ansys^b^	Lam.	–	–	Type B	Development of hemodynamics-based thrombus model; application to simplified models
Menichini et al. [488]	2016	CFD	Ansys^b^	Lam.	–	14	Type B	Validation to *in vivo* data (follow-ups)
Menichini et al. [933]	2017	CFD	Ansys^b^	Lam.	–	3	Type B	Validation to *in vivo* data (post-TEVAR follow-ups)
Yazdani et al. [489]	2018	SEM	n.a.	n.a.	–	3 (Mouse)	Type B	Development of data-driven particle-continuum thrombosis model; validation to *in vivo* data (follow-ups)
Armour et al. [934]	2020	CFD	Ansys^b^	Lam.	–	3	Type B	Impact of stent-graft end-to-distal tear distance on thrombus formation; validation to *in vivo* data (post-TEVAR follow-ups)
Jafarinia et al. [936,937]	2020/23	CFD	OpenFOAM	Lam.	–	–	Type B	Impact of blood rheology/morphological parameters on thrombus formation in simplified models
Wang et al. [945]	2021	CFD	OpenFOAM	Lam.	–	–	Type B	Development of fluid-chemical thrombus model; application to simplified models
Jiang et al. [949]	2022	CFD	Ansys^a^	Lam.	–	4	Type B	Development of two-fluid blood stasis thrombosis model; validation to *in vivo* data (post-TEVAR follow-ups)
Chong et al. [885]	2022	FSI	COMSOL^d^	Lam.	Iso.	–	Type B	Comparison of thrombus formation between simplified CFD and FSI models
Armour et al. [931]	2022	CFD	Ansys ^b^	Lam.	–	2	Type B	Impact of false lumen perfused side branches on thrombus formation in pre-/post-TEVAR patients
Jafarinia et al. [938]	2022	CFD	Ansys^b^	Lam.	–	1	Type B	Development of simplified hemodynamics-based thrombosis model; validation to *in vivo* data (post-TEVAR follow-ups)
Wang et al. [952]	2023	CFD	OpenFOAM	Lam.	–	1	Type B	Development of multiconstituent thrombosis model; validation to experiments/*in vivo* data (post-TEVAR follow-ups)
Wang et al. [940]	2023	CFD	Ansys^b^	Lam.	–	1	Type B	Development of WSS-induced thrombus breakdown model; validation to *in vivo* data (post-TEVAR follow-ups)
Gupta & Schanz [950]	2023	CSM	PANDAS	–	–	–	Type B	Development of multiphasic approach for thrombus modeling based on theory of porous media
Pei et al. [953]	2024	CFD	Ansys^a^	Lam.	–	8	Coronary artery	Impact of tear direction on hemodynamics in iatrogenic coronary artery dissection

a Fluent;

b CFX;

c Mechanics;

d Multiphysics;

e Explicit;

f Standard

## Data Availability

No data was used for the research described in the article.
